# Global Molecular Analyses of Methane Metabolism in Methanotrophic Alphaproteobacterium, *Methylosinus trichosporium* OB3b. Part I: Transcriptomic Study

**DOI:** 10.3389/fmicb.2013.00040

**Published:** 2013-04-03

**Authors:** Janet B. Matsen, Song Yang, Lisa Y. Stein, David Beck, Marina G. Kalyuzhnaya

**Affiliations:** ^1^Department of Chemical Engineering, University of WashingtonSeattle, WA, USA; ^2^Department of Biological Sciences, University of AlbertaEdmonton, AB, Canada; ^3^eScience Institute, University of WashingtonSeattle, WA, USA; ^4^Department of Microbiology, University of WashingtonSeattle, WA, USA

**Keywords:** methanotrophic proteobacteria, serine cycle, ethylmalonyl*-*CoA pathway in methanotrophs, TCA, gene expression

## Abstract

Methane utilizing bacteria (methanotrophs) are important in both environmental and biotechnological applications, due to their ability to convert methane to multicarbon compounds. However, systems-level studies of methane metabolism have not been carried out in methanotrophs. In this work we have integrated genomic and transcriptomic information to provide an overview of central metabolic pathways for methane utilization in *Methylosinus trichosporium* OB3b, a model alphaproteobacterial methanotroph. Particulate methane monooxygenase, PQQ-dependent methanol dehydrogenase, the H_4_MPT-pathway, and NAD-dependent formate dehydrogenase are involved in methane oxidation to CO_2_. All genes essential for operation of the serine cycle, the ethylmalonyl-CoA (EMC) pathway, and the citric acid (TCA) cycle were expressed. PEP-pyruvate-oxaloacetate interconversions may have a function in regulation and balancing carbon between the serine cycle and the EMC pathway. A set of transaminases may contribute to carbon partitioning between the pathways. Metabolic pathways for acquisition and/or assimilation of nitrogen and iron are discussed.

## Introduction

Aerobic methanotrophic bacteria (methanotrophs) are a highly specialized group of microbes utilizing methane as a sole source of carbon and energy (Hanson and Hanson, 1996; Murrell and Jetten, 2009). As the recognition of methane’s impact on global climate change increases, a multitude of research activities have been directed toward understanding the natural mechanisms for reducing methane emissions, including consumption by methanotrophs. The number of described microbial species capable of methane oxidation has recently expanded dramatically. A number of novel methanotrophic phyla have been isolated and described in the past few years, including new members of the Alpha- and Gammaproteobacteria, and Verrucomicrobia (Trotsenko and Murrell, 2008; Chistoserdova et al., 2009; Murrell and Jetten, 2009). Several genomes of methanotrophic bacteria have been sequenced opening new dimensions in characterization of methane metabolism (Ward et al., 2004; Dunfield et al., 2007; Hou et al., 2008; Chen et al., 2010; Stein et al., 2010, 2011; Dam et al., 2012b). Initial genome-based reconstructions of methane metabolism in methanotrophic proteobacteria and Verrucomicrobia have been performed (Ward et al., 2004; Kelly et al., 2005; Hou et al., 2008; Khadem et al., 2011).

*Methylosinus trichosporium* OB3b, an obligate alphaproteobacterial methanotroph, has served as a model system for years (first described in Whittenbury et al., 1970). Research on both fundamental and biotechnological aspects of methanotrophy in *M. trichosporium* OB3b has been carried out with applications involving cometabolism of contaminants (Oldenhuis et al., 1991; EPA, 1993; Fitch et al., 1996), epoxidation of propene (Hou et al., 1979), and synthesis of polyhydroxybutyrate (PHB) (Williams, 1988; Doronina et al., 2008). *M. trichosporium* OB3b possesses two systems for methane oxidation, a particulate methane monooxygenase (pMMO), expressed under high biomass/copper ratios, and a soluble methane monooxygenase (sMMO) which is expressed at low copper conditions (Hakemian and Rosenzweig, 2007; Semrau et al., 2010). It has been shown that the strain is capable of fixing nitrogen (Oakley and Murrell, 1988; Auman et al., 2001). Although significant progress has been made in the understanding of primary methane and methanol oxidation pathways in this model bacterium, little work has been carried out on carbon assimilation by *M. trichosporium* OB3b. The reconstruction of the core metabolic pathways for alphaproteobacterial methanotrophs has been primarily based on a restricted set of enzymatic studies and extrapolations relying on similarity to non-methane utilizing methylotrophs (Lawrence and Quayle, 1970; Strom et al., 1974). A draft genome of *M. trichosporium* OB3b has recently been generated (Stein et al., 2010). This genetic blueprint provides an essential background for revisiting the established model of methanotrophy in Alphaproteobacteria using modern system-level approaches. For this research, we integrated heterogeneous multi-scale genomic, transcriptomic, and metabolomic data to redefine the metabolic framework of C_1_-utilization in *M. trichosporium* OB3b grown in batch culture under copper, oxygen, and iron sufficiency on methane and nitrate as the sources of carbon and nitrogen, respectively. In this part of our work we present transcriptomic-based analysis of the methanotrophic metabolic network. Metabolomic and ^13^C-labeling studies are presented in a follow-up paper (Yang et al., 2013).

## Results and Discussion

### Gene expression studies

Gene expression studies were carried out with *M. trichosporium* OB3b cultures grown on methane at N (10 mM), Cu (9 μM), and Fe (9 μM) sufficiency conditions. The maximum specific growth rate of *M. trichosporium* OB3b in shake flasks during the exponential growth phase was μ = 0.038 ± 0.004 h^−1^. The methane consumption rate during the period of maximum growth rate was 8.95 mmol of CH_4_h^−1^ L culture^−1^ (OD_600_ = 1).

All experiments were performed with at least two biological replicates. RNA samples were prepared as described in the Section “Materials and Methods.” Illumina sequencing for two biological replicates (BR1 and BR2) returned 28 and 29 million 36-bp reads. The Burrows–Wheeler Aligner (BWA, Li and Durbin, 2009) aligned 98% of the reads to the *M. trichosporium* OB3b genome annotated by MaGE^1^ using the default parameters for small genomes. Reads per kilobase of coding sequence per million (reads) mapped (RPKM) (Mortazavi et al., 2008) was calculated to compare gene expression within and across replicates, and no further normalization (other than RPKM) was applied. The samples were in good agreement with each other, with per gene coding sequence RPKM correlations of 0.959 and 0.989 for the Pearson and Spearman correlations, respectively. In total, 4,762 of 4,812 ORFs (CDS, tRNA, and rRNA predicted from the draft genome) were detected. Based on relative expression, genes (omitting rRNAs) could be grouped into six major expression categories (Table 1): *very high* (RPKM ≥ 15,000), *high* (RPKM ≥ 1,500), *moderate* (1,500 > RPKM ≥ 500), *modest* (500 > RPKM ≥ 250), *low* (250 > RPKM ≥ 150) *very low* (150 > RPKM ≥ 15), and *not expressed* (RPKM < 15). The majority of genes fell into *low/very low expression* categories (74%). About 14% of genes displayed *moderate/modest* expression and only a small fraction of the genome showed very *high/high expression* (2.7%).

**Table 1:** **Classification of gene expression level based on replicate averaged RPKMs**.

Description of expression level	RPKM range	% of ORFs	Number of ORFs
Very high	>15,000	0.23	11
High	1,500–15,000	2.49	120
Moderate	500–1,500	5.30	255
Modest	250–500	8.61	414
Low	50–250	40.41	1,944
Very low	15–50	23.70	1,140
Not expressed	<15	19.27	927

In order to determine whether the draft genome of the strain is missing some functional genes, we performed *de novo* assembly of the transcriptome. Using this approach, a total of 173 genes that are not present in the genome sequence, but have homologs in the non-redundant database were detected. Among those are key subunits of succinate dehydrogenase (*sdhABCD*), 2-oxoglutarate dehydrogenase (E2), and nitric oxide reductase (*norB*) (Table S1 in Supplementary Material). The *de novo* transcriptome assembly provides additional information for highly expressed genes and it was used for verification of some metabolic functions that were predicted by enzymatic studies but were not detected in the draft genome assembly (see below).

**Figure 1:**
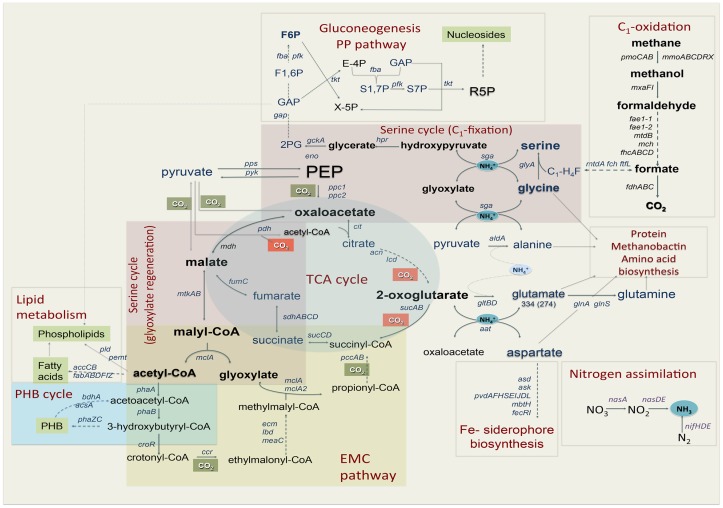
**Central metabolism of *Methylosinus trichosporium* OB3b grown on methane as sole source of energy and carbon as deduced from the genome sequences and transcriptomic studies**. Font size of the gene name indicates the expression level.

In addition, the reads obtained from RNA-seq were aligned to the reference genome in order to identify transcription boundaries and transcription start sites for the most highly expressed genes, including the *pmoCAB* operons, *mxaFJGI* operon, *fae1*, *pqqA*, and key genes of the serine cycle (Table S2 in Supplementary Material, see description below). Gene expression data were used to reconstruct central metabolic pathways in *M. trichosporium* OB3b (Table 2; Figure 1; Table S2 in Supplementary Material). Core functions are described below.

**Table 2:** **Gene expression profile in methane-grown cells of *M. trichosporium* OB3b**.

Gene ID	Predicted function	Gene	Replicate 1	Replicate 2
**METHANE AND METHANOL OXIDATION**				
METTOv1_1270003	Particulate methane monooxygenase subunit C	*pmoC*	123026	127241
METTOv1_1270002	Particulate methane monooxygenase subunit A	*pmoA*	37102	31813
METTOv1_1270001	Particulate methane monooxygenase subunit B	*pmoB*	27371	22917
METTOv1_310040	Particulate methane monooxygenase subunit C2	*pmoC2*	532	492
METTOv1_50081	Soluble methane monooxygenase alpha subunit	*mmoX*	9	8
METTOv1_50082	Soluble methane monooxygenase beta subunit	*mmoY*	13	9
METTOv1_50084	Soluble methane monooxygenase gamma subunit	*mmoZ*	20	19
METTOv1_240014	PQQ-dependent methanol dehydrogenase	*mxaF*	15313	13760
METTOv1_240011	PQQ-dependent methanol dehydrogenase	*mxaI*	24552	28474
METTOv1_240012	Cytochrome *c* class I	*mxaG*	5712	6117
METTOv1_240013	Extracellular solute-binding protein family 3	*mxaJ*	1942	1838
METTOv1_240001	Putative methanol utilization control sensor protein	*mxaY*	36	41
METTOv1_240002	Putative two-component response regulator	*mxaB*	303	317
METTOv1_240003	MxaH protein, involved in methanol oxidation	*mxaH*	399	391
METTOv1_240004	MxaD protein, involved in methanol oxidation	*mxaD*	1137	1077
METTOv1_240005	von Willebrand factor type A, involved in methanol oxidation	*mxaL*	191	201
METTOv1_240006	Protein of unknown function, involved in methanol oxidation	*mxaK*	124	132
METTOv1_240007	von Willebrand factor type A, involved in methanol oxidation	*mxaC*	144	141
METTOv1_240008	MxaA protein, involved in methanol oxidation	*mxaA*	137	127
METTOv1_240009	MxaS protein, involved in methanol oxidation	*mxaS*	202	167
METTOv1_240010	ATPase, involved in methanol oxidation	*mxaR*	563	538
METTOv1_110056	Coenzyme PQQ biosynthesis protein A	*pqqA*	11857	13927
METTOv1_160001	Coenzyme PQQ biosynthesis protein E	*pqqE*	166	161
METTOv1_160002	Coenzyme PQQ biosynthesis protein PqqC/D	*pqqC/D*	372	344
METTOv1_160003	Coenzyme PQQ biosynthesis protein B	*pqqB*	306	313
METTOv1_20046	Coenzyme PQQ biosynthesis protein F	*pqqF*	183	185
METTOv1_20047	Coenzyme PQQ biosynthesis protein G	*pqqG*	157	142
METTOv1_610028	Aldehyde dehydrogenase	*aldh*	37	37
METTOv1_290006	Aldehyde oxidase	*aor*	45	38
METTOv1_100046	Aldehyde dehydrogenase	*aldh-F7*	7	9
**FORMALDEHYDE OXIDATION**				
METTOv1_40010	Methenyltetrahydromethanopterin cyclohydrolase	*mch*	393	312
METTOv1_40011	Tetrahydromethanopterin-linked C1 transfer pathway protein. Orf5	*orf5*	128	111
METTOv1_40012	Tetrahydromethanopterin-linked C1 transfer pathway protein, Orf7	*orf7*	73	72
METTOv1_40013	Formaldehyde activating enzyme	*fae1*	24353	24787
METTOv1_40014	Formaldehyde activating enzyme	*fae1-2*	4024	3676
METTOv1_840013	Formaldehyde activating enzyme homolog	*fae2*	535	581
METTOv1_40015	Tetrahydromethanopterin-linked C1 transfer pathway protein	*orf17*	38	45
METTOv1_110058	Tetrahydromethanopterin formyltransferase, subunit C	*fhcC*	535	453
METTOv1_110059	Tetrahydromethanopterin formyltransferase, subunit D	*fhcD*	496	470
METTOv1_110060	Tetrahydromethanopterin formyltransferase, subunit A	*fhcA*	591	546
METTOv1_110061	Tetrahydromethanopterin formyltransferase, subunit B	*fhcB*	620	570
METTOv1_560001	Tetrahydromethanopterin -linked C1 transfer pathway protein	*orf9*	172	167
METTOv1_560002	Methylenetetrahydrofolate dehydrogenase (NAD)	*mtdB*	688	607
METTOv1_440045	Ribofuranosylaminobenzene 5′-phosphate synthase	*mptG*	94	80
**FORMATE OXIDATION**				
METTOv1_630016	Transcriptional regulator, LysR family	*fdsR*	52	39
METTOv1_630017	NAD-linked formate dehydrogenase, subunit G	*fdsG*	672	608
METTOv1_630018	NAD-linked formate dehydrogenase, subunit B	*fdsB*	585	531
METTOv1_630019	NAD-linked formate dehydrogenase, subunit A	*fdsA*	593	554
METTOv1_370001	Formate dehydrogenase family accessory protein	*fdsC*	210	199
METTOv1_370002	NAD-linked formate dehydrogenase, subunit D	*fdsD*	368	312
METTOv1_220028	NAD-linked formate dehydrogenase, subunit A	*fdhA2*	9	7
**C1-ASSIMILATION:SERINE CYCLE**				
METTOv1_130002	Phosphoenolpyruvate carboxylase	*ppc2*	104	89
METTOv1_400011	Glycerate kinase	*gckA*	229	211
METTOv1_400012	Conserved protein of unknown function	*orf1*	626	708
METTOv1_400013	Malyl-CoA lyase/beta-methylmalyl-CoA lyase	*mclA*	1713	1615
METTOv1_400014	Phosphoenolpyruvate carboxylase	*ppc1*	141	139
METTOv1_400015	Malate thiokinase, small subunit	*mtkB*	516	485
METTOv1_400016	Malate thiokinase, large subunit	*mtkA*	534	455
METTOv1_400017	Methenyltetrahydrofolate cyclohydrolase	*fch*	355	281
METTOv1_400018	NADP-dependent methylenetetrahydrofolate dehydrogenase	*mtdA*	281	243
METTOv1_400019	2-Hydroxyacid dehydrogenase NAD-binding	*hprA*	375	348
METTOv1_400020	Serine-glyoxylate transaminase	*sga*	1840	1969
METTOv1_400021	Formate-tetrahydrofolate ligase	*ftfL*	448	412
METTOv1_670019	Serine hydroxymethyltransferase	*glyA*	1342	1197
METTOv1_20135	Enolase	*eno*	432	408
**C1-ASSIMILATION:EMP PATHWAY AND PHB CYCLE**				
METTOv1_100079	Acetyl-CoA acetyltransferase	*phaA*	597	561
METTOv1_100080	Acetoacetyl-CoA reductase	*phaB*	1160	1060
METTOv1_50006	Crotonase	*croR*	235	275
METTOv1_110068	Crotonyl-CoA reductase	*ccr*	577	523
METTOv1_60013	Ethylmalonyl-CoA mutase	*ecm*	187	162
METTOv1_510010	Methylsuccinyl-CoA dehydrogenase	*ibd*	309	295
METTOv1_110043	Mesaconyl-CoA hydratase	*meaC*	341	317
METTOv1_30129	Methylmalonyl-CoA epimerase	*epm*	428	394
METTOv1_220010	Malyl-CoA lyase/beta-Methylmalyl-CoA lyase	*mclA2*	137	135
METTOv1_200020	Acetyl/propionyl-CoA carboxylase	*ppcA*	353	310
METTOv1_220035	Propionyl-CoA carboxylase	*ppcB*	472	455
METTOv1_50067	Methylmalonyl-CoA mutase, large subunit	*mcmA*	201	188
METTOv1_10062	Methylmalonyl-CoA mutase small subunit B	*mcmB*	144	144
METTOv1_270063	3-Hydroxybutyrate dehydrogenase	*bdhA*	235	232
METTOv1_130047	Poly-beta-hydroxybutyrate polymerase	*phaC*	30	30
METTOv1_200042	Acetoacetate decarboxylase	*aad*	123	114
METTOv1_200022	Acetoacetyl-coenzyme A synthetase	*aas*	116	114
METTOv1_630008	Polyhydroxyalkanoate depolymerase	*phaZ*	237	263
**C1-ASSIMILATION: TCA CYCLE**				
METTOv1_360040	Malate dehydrogenase	*mdh*	539	473
METTOv1_360041	Succinyl-CoA synthetase, beta subunit	*sucC*	660	631
METTOv1_510003	Succinyl-CoA synthetase, alpha subunit	*sucD*	1198	1135
METTOv1_510002	2-Oxoglutarate dehydrogenase E1	*sucA*	236	237
METTOv1_370050	2-Oxoglutarate dehydrogenase E2	*sucB*	191	181
METTOv1_80046	Succinate:ubiquinone oxidoreductase	*sdhB*	327	348
METTOv1_80046	Succinate:ubiquinone oxidoreductase	*sdhA*	311	299
METTOv1_80051	Succinate:ubiquinone oxidoreductase, cytochrome b556 subunit	*sdhC*	318	329
METTOv1_40061	Fumarate hydratase	*fum*	196	185
METTOv1_1080004	2-Oxoacid ferredoxin oxidoreductase	*ofr*	79	77
**INTERMEDIARY METABOLISM AND ANAPLEROTIC CO_2_-FIXATION**				
METTOv1_70038	Phosphoenolpyruvate synthase	*pps*	28	26
METTOv1_120036	Pyruvate carboxylase	*pcx*	145	140
METTOv1_830002	Acetyl-coenzyme A carboxylase subunit beta	*accD*	246	228
METTOv1_380021	Acetyl-CoA carboxylase subunit alpha	*accA*	211	204
METTOv1_130018	Acetyl-CoA carboxylase, biotin carboxyl carrier protein	*accB*	307	276
METTOv1_150014	Pyruvate kinase	*pyk1*	245	224
METTOv1_340039	Pyruvate dehydrogenase (acetyl-transferring) E1	*pdhA*	181	175
METTOv1_340041	Pyruvate dehydrogenase subunit beta	*pdhB*	160	158
METTOv1_340042	Pyruvate dehydrogenase	*pdhC*	101	106
METTOv1_350050	Pyruvate phosphate dikinase	*pdk*	50	52
METTOv1_80025	Malic enzyme	*mae*	107	106
METTOv1_680013	Phosphoglycerate mutase	*gpmA*	136	135
METTOv1_100061	Phosphoglycerate mutase (modular protein)	*pgm*	101	99
METTOv1_10180	Phosphoglycerate mutase (modular protein)	*pgm*	72	65
METTOv1_280049	Phosphoglycerate kinase	*pgk*	199	208
METTOv1_280047	Glyceraldehyde-3-phosphate dehydrogenase	*gpd*	391	407
METTOv1_620016	Ribokinase	*rik*	140	144
METTOv1_620017	Phosphoribulokinase	*prk*	173	164
METTOv1_620018	Transketolase	*tkl*	147	123
METTOv1_620019	Fructose-bisphosphate aldolase, class II	*fba*	427	461
METTOv1_220030	6-Phosphofructokinase	*pfk*	243	239
METTOv1_200031	Fructose 1,6-bisphosphatase II	*glp*	68	56
METTOv1_550029	Glucose-6-phosphate isomerase	*pgi*	88	84
METTOv1_620022	Ribulose-phosphate 3-epimerase	*rpe*	51	53
**NITROGEN, Cu, Fe METABOLISM**				
METTOv1_310019	Nitrate transporter component	*nrtA*	151	154
METTOv1_310020	Nitrite reductase (NAD(P)H), large subunit	*nasB*	1762	1562
METTOv1_310021	Nitrite reductase (NAD(P)H), small subunit	*nasD*	726	610
METTOv1_310022	Nitrate reductase, large subunit	*nasA*	646	597
METTOv1_130049	Ammonium transporter	*amtB*	2029	1766
METTOv1_300058	Glutamate synthase large subunit (NADPH/GOGAT)	*gltB*	199	183
METTOv1_300033	Glutamate synthase small subunit (NADPH/GOGAT)	*gltD*	431	401
METTOv1_190023	Glutamate dehydrogenase	*gdh*	2	2
METTOv1_200046	Glutamate-ammonia ligase	*glnS*	1413	1375
METTOv1_200047	Nitrogen regulatory protein P-II	*glnK*	2400	2277
METTOv1_200048	Glutamine synthetase, type I	*glnA*	2452	2321
METTOv1_280018	Alanine dehydrogenase	*aldA*	29	36
METTOv1_80043	Phosphoserine aminotransferase	*serC*	415	364
METTOv1_560023	Cytochrome *c*′-alpha	*cycA*	336	370
METTOv1_230076	Putative oxygenase		6	8
METTOv1_230077	Hydroxylamine reductase	*hcp*	21	16
METTOv1_230078	Putative transcriptional regulator	*nsrR*	38	35
METTOv1_730005	Putative FecR iron sensor protein	*fecR*	48	58
METTOv1_730006	Putative TonB-dependent receptor protein	*tonB*	382	383
METTOv1_CDS4222756D	Methanobactin precursor	*Mb*	1439	2177
METTOv1_730007	Putative lyase		306	384
METTOv1_730008	Conserved protein of unknown function	*hp*	170	175
METTOv1_730009	Conserved protein of unknown function	*hp*	116	143
METTOv1_660011	l-Ornithine 5-monooxygenase	*pvdA1*	1410	1450
METTOv1_760004	Putative hydroxy-l-ornithine formylase	*pvdF*	2255	2676
METTOv1_760006	l-Ornithine 5-monooxygenase	*pvdA2*	979	1116
METTOv1_760007	Diaminobutyrate-2-oxoglutarate aminotransferase	*pvdH*	720	770
METTOv1_760008	Sigma-24 (FecI-like)	*pvdS*	1260	1522
METTOv1_760009	Putative pyoverdine ABC export system, permease	*pvdE*	532	500
METTOv1_760010	TonB-dependent siderophore receptor	*fpvA*	2197	2174
METTOv1_760011	FecR-like protein	*fecR*	330	321
METTOv1_760012	FecI-family sigma factor	*fecI*	922	951
METTOv1_870003	Ferribactin synthase	*pvdL*	433	441
METTOv1_870004	Pyoverdine biosynthesis regulatory protein-TauD/TfdA family protein		932	1039
METTOv1_870005	Pyoverdine synthetase, thioesterase component	*pvdG*	1542	1473
METTOv1_870006	Integral components of bacterial non-ribosomal peptide synthetases	*MbtH*	4176	5481
METTOv1_1220001	Putative pyoverdine sidechain peptide synthetase IV, d-Asp-l-Ser component	*pvdI/J*	480	564
METTOv1_1220002	Putative non-ribosomal peptide synthase	*pvdJ/D*	379	396

*Values represent reads per kilobase of coding sequence per million (reads) mapped (RPKM)*.

### C_1_-oxidation: Methane-to-methanol

It has been previously demonstrated that *M. trichosporium* OB3b possesses two types of methane oxidation enzymes: pMMO and sMMO. The expression of the enzymes is determined by copper availability; sMMO is dominant in copper-limited environments while pMMO dominates under copper sufficiency (Hakemian and Rosenzweig, 2007; Semrau et al., 2010). Structures of both enzymes are available (Elango et al., 1997; Hakemian et al., 2008). In this study, *M. trichosporium* OB3b was grown at a copper concentration that has been shown to be sufficient to suppress the expression of sMMO (Park et al., 1991; Phelps et al., 1992; Nielsen et al., 1997; Lloyd et al., 1999; Murrell et al., 2000). Indeed, virtually no expression of the sMMO gene cluster (*mmoXYBZC*) was observed. In contrast, the *pmoCAB* genes were the most highly expressed in the transcriptome, representing about 14% of all reads mapped to the coding regions (Table 2). It has previously been shown that PMMO in *M. trichosporium* OB3b is encoded by two copies of the *pmoCAB* operon that appear to be identical (Gilbert et al., 2000). The current genome assembly failed to resolve these closely related duplicated regions. The *pmoCAB* genes were found within one relatively short contig, which includes 320 bp upstream from *pmoC*, and about 66 bp downstream from *pmoB*. It is possible that in the genome assembly, the *pmo* contig represents only those parts of the duplicated regions that are highly similar. Thus, it was not possible to determine relative expression of the two operons with the transcriptomic data.

Previous attempts to identify transcriptional starts of the *pmoCAB* operons in *M. trichosporium* OB3b using a conventional primer extension approach were not successful (Gilbert et al., 2000). The RNA-seq data were used for identification of transcriptional starts for the *pmoCAB* operons. Because the published METTOv1 genome did not contain a complete *pmoCAB* cluster, a separate alignment run was performed using a previously published sequence as the scaffold (Holmes et al., 1995). For this sequence, two possible transcriptional start sites were identified. It is not known whether these reflect the same start sites of both operons, different start sites for each, or expression of only one operon with two start sites. The position −274nt (A) from the translational start of the *pmoC* gene was predicted as the most prominent start of transcription of the operon (Figure S1 in Supplementary Material). Putative σ^70^-like -10 and -35 regions could be identified upstream of the predicted start (Table S3 in Supplementary Material). The structure of the putative promoter region from *M. trichosporium* OB3b shows significant similarity to a *pmoCAB* promoter region previously identified in *Methylocystis* sp. M (Gilbert et al., 2000). Another potential transcriptional start is at position −324 from the translational start of the *pmoC* gene. It should be noted that the region between the two predicted start sites was also covered with relatively high count (region between −324 and −274nt with respect to the translational start of *pmoC*). No putative promoter sequences were found upstream of position 324.

The genome predicts an additional copy of the *pmoC* gene by itself (*pmoC2, METTOv1_310040*), which can be distinguished from the other *pmoC* genes in the transcriptomics data due to sequence divergence. It has previously been demonstrated that additional copies of *pmoC* are essential for methanotrophic growth in other strains (Stolyar et al., 1999; Dam et al., 2012a). It has also been shown that the homologous *amoC* (additional lone copy of amoC in ammonia-oxidizing bacterium *Nitrosomonas europaea*) plays role in cell recovery from ammonium starvation (Berube and Stahl, 2012). However the functional role of PmoC is not known. The relative expression of *pmoC2* was approximately 450-fold less than the expression of the two *pmoC* genes from the *pmo-*operons (Table 2). Low relative expression of the *pmoC* homolog may suggest a role in regulation or sensing rather than catalytic activity.

### C_1_-oxidation: Methanol-to-formaldehyde

The product of methane oxidation (i.e., methanol) is converted to formaldehyde by a PQQ-dependent methanol dehydrogenase (MDH) (Anthony, 1982, 2002; Yamada et al., 1991; Anthony et al., 1994). The enzyme has been previously purified from *M. trichosporium* OB3b and well characterized (Yamada et al., 1991). MDH is a hetero-tetrameric enzyme encoded by *mxaF* and *mxaI*. The activity of the enzyme *in vivo* requires cytochrome c_L_ (*mxaG*) and a number of chaperones, regulators, and enzymes, including genes required for Ca^2+^ insertion (Anthony et al., 1994; Anthony, 2002). Most of the genes essential for this methanol conversion step in *M. trichosporium* OB3b are organized in one large operon in an order similar to that found in other methylotrophs (Figure S2A in Supplementary Material). The first four genes of the operon (*mxaFJGI*), encoding the two subunits of the MDH, the associated cytochrome, and a gene of unknown function (*mxaJ*) were detected at relatively high RPKM counts. The relative expression of genes downstream from *mxaI*, including those for chaperones, regulators, and Ca^2+^ insertion functions drops by 10- to 50-fold (Table 2). The overall mapping pattern of the *mxa-*cluster is as follows: *mxaFJGI* (highly expressed)*, mxaD* (moderate expression), *mxaRSACKL, mxaB* (low expression), and *mxaY* (very low expression*)*. It remains to be elucidated if the *mxaRSACKL* transcripts arise from the same start as *mxaF* and are attenuated by some transcriptional or post-transcriptional mechanism, or whether separate, lower expression promoter(s) is/are present. Orientations and/or the expression patterns of *mxaD*, *mxaB*, and *mxaY*, suggest that they are not part of the major *mxaF-*operon (Figure S2A in Supplementary Material) and most likely have independent regulatory/promoter regions. According to RNA-seq mapping data, a putative transcriptional start of the *mxaFJGI* operon is predicted at position −164 from the predicted translational start (Figure S3 in Supplementary Material). Just upstream from the predicted transcriptional start, putative σ^70^-like −10 and −35 sequences were identified (Table S3 in Supplementary Material).

The genome of *M. trichosporium* OB3b contains the following three homologs of the large subunit of the MDH: *xoxF1, xoxF2*, and *xoxF3*. Relative expression of all *xoxF-*homologs is very low. The most highly expressed *xox*-homolog (*xoxF1*) showed only about 2% of the *mxaF* expression. The function of the *xox*-gene products has not been studied in *M. trichosporium* OB3b. In the non-methanotrophic methylotroph *Methylobacterium extorquens* AM1, it has been shown that *xoxF* may display methanol-oxidizing activity (Schmidt et al., 2010), and can contribute to the complex regulation of *mxa*-genes (Skovran et al., 2011). Furthermore, there are suggestions that *xoxF* may play a role in formaldehyde oxidation (Wilson et al., 2008). The low expression of all *xoxF-*homologs in *M. trichosporium* OB3b compared to *mxaFI* or H_4_MTP-linked pathway genes suggests that *xox*-genes may have no or a minor contribution to methanol oxidation in *M. trichosporium* OB3b under the tested growth conditions. However, our data do not rule out the possibility that one or more of the *xoxF* gene products are involved in regulation, either of methanol or formaldehyde oxidation.

**Figure 2:**
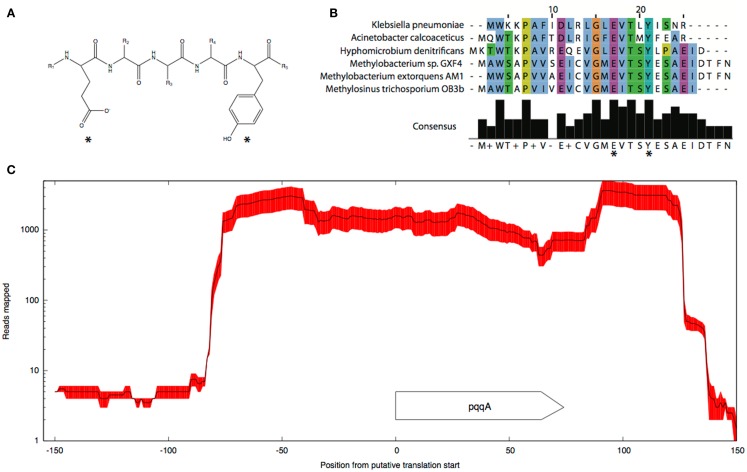
**Predicted structure (A) and alignment of the putative *pqqA* peptide (B) from *M. trichosporium* OB3b and *pqqA* peptides from *Methylobacterium* sp. GXF4, *M. extorquens* AM1, *Hyphomicrobium denitrificans* ATCC51888, *K. pneumoniae*, and *A. calcoaceticus***. **(C)** Mapping.

Pyrroloquinoline quinone (PQQ) biosynthesis is another function essential for operation of the primary methanol oxidation system (Toyama et al., 1997; Anthony, 2002). A total of six *pqq* genes appear to be present in the *M. trichosporium* OB3b genome in two clusters: *pqqBCDE* and *pqqFG*. Moderate expression of both clusters was observed (Table 2). No gene for the small PQQ precursor (PqqA) is predicted in the current version of the genome. Our manual review of the sequences revealed a fragment within the METTOv1_110055 – METTOv1_110057 gene locus (positions 1424678 – 1424755 of current version of the genome) with high sequence identity [83% nucleic acid (NA) identity and 96% amino acid (AA) similarity] to the *pqqA* sequence from *Methylobacterium* spp (Figures 2A,B). Transcript mapping data indicated that only the *pqqA*-like region of the locus is highly expressed (Figure 2C). The relative expression of the putative PQQ precursor gene is comparable to the high expression of the *mxaFI* genes. The rest of the genes involved in PQQ biosynthesis showed modest to low expression (Figure S2B in Supplementary Material).

### C_1_-oxidation: Formaldehyde-to-formate

Previous enzymatic studies predict three possible pathways for formaldehyde oxidation: (1) direct oxidation through dye-linked heme-containing formaldehyde dehydrogenase (Patel et al., 1980), (2) H_4_folate-, and (3) H_4_MTP-mediated C_1_ transfers (Vorholt et al., 1999; Doronina et al., 2008). Contrary to enzymatic studies, BLAST searches of the draft genome of *M. trichosporium* OB3b did not reveal any obvious system that could be attributed to heme-containing formaldehyde oxidation. Three broad-specificity aldehyde-detoxification systems, including two NAD-dependent aldehyde dehydrogenases (Aldh-F7 METTOv1_100046 and Aldh, METTOv1_610028) and one aldehyde oxidase (*Aor*, METTOv1_290006) were predicted in the genome. Two of them, *aldh* and *aor* show low expression (Table 2), while *aldh-F7* was barely detected in the transcriptome. None of the putative genes identified by *de novo* transcriptome assembly could be readily attributed to any dye-linked aldehyde dehydrogenases (Schwartz et al., 2004). Thus, even if the enzyme is present in the genome, its expression during growth of the strain on methane must be low.

For years it has been assumed that methylene H_4_F is formed as a result of the spontaneous (non-enzymatic) condensation of formaldehyde with H_4_F (Large and Quayle, 1963). It has recently been demonstrated that formate, rather than formaldehyde serves as an entry substrate for assimilation in serine cycle methylotrophs (Crowther et al., 2008). With this metabolic arrangement, the H_4_Folate C_1_ transfer could be considered as a part of the assimilatory network that converts formate into methylene H_4_F. In *M. trichosporium* OB3b genes encoding all three steps of the H_4_Folate pathway converting methylene-H_4_F to formate (formyl-H_4_F ligase, *ftfL*, methenyl-H_4_F cyclohydrolase, *fch*, and methylene-H_4_F dehydrogenase *mtdA*) were co-localized and co-transcribed with genes encoding the serine cycle enzymes (Figure S4 in Supplementary Material). While the assimilatory function of the pathway is more apparent, it is still possible that key enzymes of the pathway contribute to formaldehyde oxidation in *M. trichosporium* OB3b.

The tetrahydromethanopterin (H_4_MTP) pathway was proposed to be the key pathway for formaldehyde oxidation/detoxification in alphaproteobacterial methylotrophs (Chistoserdova et al., 1998). It was speculated that this pathway contributes to formaldehyde oxidation in methane utilizing proteobacteria (Vorholt et al., 1999). Nineteen genes encoding enzymes and genes for tetrahydromethanopterin biosynthesis were identified in the *M. trichosporium* OB3b genome. These genes were not clustered together in the genome, but formed five different gene islands: (1) *mch-orf5-orf7-fae1/1-fae1/2-orf17* (Figure S2C in Supplementary Material); (2) *orf19-orf20*-*afpA*-*orf21-orf22*; (3) *fhcCDAB*; (4) *orf9-mtdB*; and (5) *pcbD-mptG*. No homologs of o*rfY, dmrA*, and *pabB* genes, which are commonly associated with tetrahydromethanopterin biosynthesis in methylotrophs (Caccamo et al., 2004; Rasche et al., 2004; Chistoserdova et al., 2005; Kalyuzhnaya et al., 2005), were found in the genome or detected in transcriptome.

Formaldehyde activating enzyme (FAE) is the first enzyme of the H_4_MTP-pathway, which has been shown to catalyze the condensation of formaldehyde and H_4_MPT to form methylene-H_4_MPT (Vorholt et al., 2000). The draft genome of *M. trichosporium* OB3b predicts three homologs of Fae; two of them (*fae1/1* and *fae1/2*) share a high degree of identity (NA 82.2%) and are co-localized in the genome (Figure S2C in Supplementary Material). Though both *fae* genes are clustered with four other genes involved in the H_4_MTP-pathway, they are expressed in dramatically different patterns. The abundance of *fae1-1* transcripts was almost 40-fold higher than the abundance of any other gene in the cluster, except *fae1-2* (Table 2; Figure S2C in Supplementary Material). The relative abundance of the second homolog (*fae1-2*) was one fifth of that observed for *fae1-1*, and *fae1-2* was the second highest expressed gene in the pathway. Mapping data indicate that *fae1-1* and *fae1-2* are most likely co-transcribed (Figure S5 in Supplementary Material). RNA-Seq mapping data suggest two putative transcriptional starts at the positions −215 (with σ^70^-like −10 and −35 sequences upstream) and –105 (with a conserved “AATGGTTG” sequence in the −35 region) upstream from the *fae1-1* translational start (Figure S5 in Supplementary Material; Table S3 in Supplementary Material).

The third homolog of Fae (*fae2*) demonstrates moderate expression. The rest of the genes encoding key enzymes of the pathway (*mtdB*, methylene-H_4_MPT dehydrogenase; *mch*, methenyl-H_4_MPT cyclohydrolase; *fhcABCD*, formyltransferase/hydrolase) have a similarly moderate expression. The relative abundance of genes encoding key enzymes of the pathway were 5- to 10-fold higher than those involved in cofactor biosynthesis. Overall, transcriptomic data indicate that the H_4_MTP-pathway serves as the key pathway for formaldehyde oxidation in *M. trichosporium* OB3b.

### Formate oxidation

Formate is oxidized to CO_2_ by a NAD-dependent formate dehydrogenase in most, if not all, methanotrophs (Anthony, 1982). It has been suggested that most of the reducing power required for methane metabolism is produced by formaldehyde oxidation to formate and then to CO_2_ (Hanson and Hanson, 1996). It has been speculated that in microbes with a functional serine pathway, formate serves as a key branch point between assimilation and catabolism (Chistoserdova, 2011). NAD-dependent formate dehydrogenase from *M. trichosporium* OB3b has been purified and characterized (Jollie and Lipscomb, 1991). The enzyme is composed of four subunit types and contained flavin, iron, and molybdenum (Jollie and Lipscomb, 1991). The genome of *M. trichosporium* OB3b predicts one NAD-dependent molybdenum-containing formate dehydrogenase encoded by *fdsABGCD* and an additional single copy of the alpha subunit (*fdhA)*. The two genes *fdsA* and *fdhA* share 81% identity. Only one of them, *fdsA* as well as the rest of the *fds* cluster genes were expressed in the transcriptome (Table 2).

### C_1_-assimilation: Serine cycle

It has been previously suggested that the serine cycle is the major pathway for C_1_-assimilation in *M. trichosporium* OB3b (Strom et al., 1974). All genetic elements essential for operation of the cycle were predicted in the genome and cluster together (Stein et al., 2010). However, genes of the pathway show deferent levels of expression (Table 2). While *sga*, *glyA*, and *mclA* have high expression levels, the rest of the genes involved in the pathway show modest expression (Table 2; Figure S4 in Supplementary Material). In addition to the serine-glyoxylate aminotransferase (*sga*), a key aminotransferase in the central metabolism of serine cycle microbes, moderate levels of expression were observed for two other aminotransferases, phosphoserine aminotransferase, and aspartate aminotransferase (Table 2).

**Figure 3:**
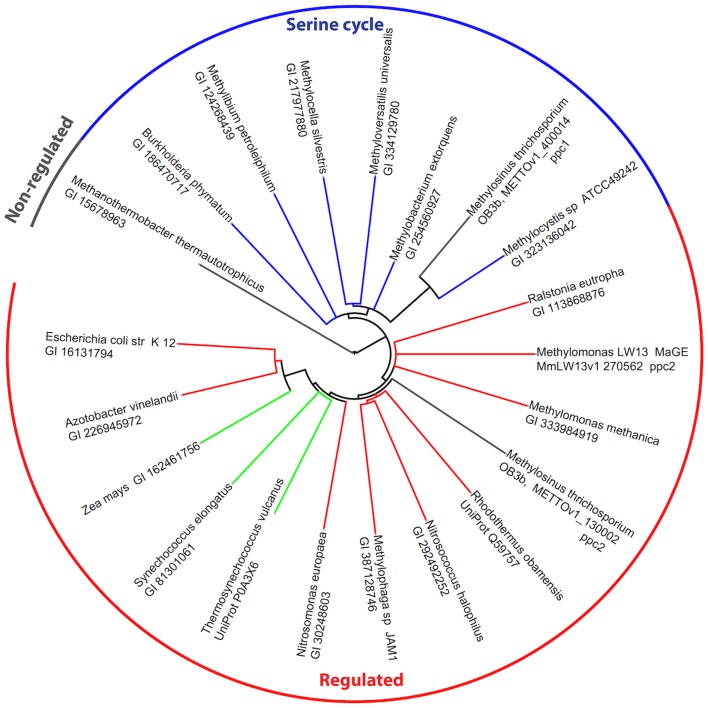
**Phylogenetic tree of phosphoenolpyruvate carboxylases**. Sequence identifiers follow the source organism label. Sequences were aligned with MUSCLE v3.8.31 (Edgar, 2004) and the tree created with ClustalW2 2.0.12 (Larkin et al., 2007) and rendered with iTOL (Letunic and Bork, 2007).

The genome of *M. trichosporium* OB3b encodes two copies of phosphoenolpyruvate (PEP) carboxylase (*ppc1* and *ppc2*). The two enzymes are only distantly related to each other and share 33% identity at the amino acid level. One of them, Ppc1, clusters with PEP carboxylases usually found in bacteria possessing the serine cycle for C_1_-assimilation (Figure 3). Serine cycle Ppcs belong to a “*non-regulated*” group of PEP carboxylases (Anthony, 1982). The activity of these enzymes is not controlled by intermediates of the TCA cycle or glycolysis/gluconeogenesis (Newaz and Hersh, 1975). The second homolog of Ppc (*ppc2*) clusters with anaplerotic “*regulated*” PEP carboxylases, which are controlled by a variety of metabolic effectors (Takahashi et al., 1993; Kai et al., 2003). Both *ppc1* and *ppc2* transcripts demonstrate comparable levels of abundance in this study (Table 2). The sequences of the two genes were further investigated in an attempt to better understand the rationale for the enzymatic redundancy at the PEP to oxaloacetate conversion step. We used multiple sequence alignments of Ppc1, Ppc*2*, and other characterized PEP carboxylases and homology models (not shown) built from tensed and relaxed state crystal structures (Matsumura et al., 2002) to investigate the predicted allosteric regulation sites of these two enzymes. The alignment shows that only the catalytic elements, such as PEP-binding-site residues, are conserved in both proteins (Ppc1 and Ppc2, Figure S6 in Supplementary Material). However, sequence features required for the allosteric regulation of the enzyme activity show several structural differences. The majority of the characterized bacterial PEP carboxylases are activated by acetyl-CoA, FBP, long-chain fatty acids, and pGp. Inhibition occurs in the presence of aspartate and l-malate. In the case of Ppc1 from *M. trichosporium* OB3b, two of the four highly conserved polar amino acids that bind allosteric inhibitors (e.g., aspartate, malate) were hydrophobic: L805 and A912 (K and N in *E. coli* respectively) suggesting alternate inhibitors or a lack of sensitivity to l-malate and aspartate. The activator-binding residues were conserved except for a R159 instead of K. By contrast, Ppc2 was well conserved relative to the well characterized PEP carboxylases and for those with structures, only minor rearrangements of the monomeric interfaces were predicted. It is tempting to speculate that the presence of two functionally identical but differently regulated enzymatic systems in *M. trichosporium* OB3b evolved as a way to control flux through PEP-oxaloacetate in response to levels of the serine cycle and EMC pathway intermediates. The flux is never completely blocked, due to the insensitivity of Ppc1 to the metabolic state of the cell. Increases in the intracellular levels of acetyl-CoA, aspartate, or malate (as a result of saturation of the downstream EMC pathway and the TCA cycle) can reduce the flux through the PEP-oxaloacetate presumably twofold via allosteric inhibition and lack of activation of Ppc2 activity. In this case, C_1_-carbon assimilated via the serine cycle is re-directed to gluconeogenesis or converted into pyruvate.

Regeneration of glyoxylate is an essential part of the serine cycle (Anthony, 1982, 2011; Peyraud et al., 2009). Like other obligate methanotrophic bacteria, *M. trichosporium* OB3b lacks isocitrate lyase, a key enzyme of the glyoxylate shunt (Trotsenko and Murrell, 2008). Homologs of enzymes involved in the ethylmalonyl-CoA (EMC) pathway, an alternative route for glyoxylate regeneration (Peyraud et al., 2009), were identified in the draft genomes of *M. trichosporium* OB3b and another obligate methanotroph *Methylocystis* sp. (Stein et al., 2010, 2011). However, a functional EMC pathway has not yet been demonstrated in methanotrophs. Furthermore, the recent investigation of PHB-metabolism in *Methylocysctis parvus* OBBP, an alphaproteobacterial methanotroph, suggested that this metabolic module can not supply C_2_ (glyoxylate) units for biosynthesis (Pieja et al., 2011). As the initial steps of the EMC pathway are shared with PHB biosynthesis [acetyl-CoA acetyltransferase (*phaA*) and acetoacetyl-CoA reductase (*phaB*), see Figure 1] in context with the data presented by Pieja et al. (2011) call into question the operation of the EMC pathway in methanotrophs.

We found that genes encoding the initial steps of the PHB-synthesis (*phaA/phaB)* show moderate levels of expression. As could be expected for cells in early-mid exponential growth, the expression of the gene encoding PHB synthase (*phaC*) was low. The data are consistent with previous observations of high activity of PhaA and PhaB and low activity of PHB synthase (PhaC) in exponentially grown cells of *M. trichosporium* OB3b (Williams, 1988; Doronina et al., 2008). The PHB-degradation pathway genes, including 3-hydroxybutyrate dehydrogenase, acetoacetate decarboxylase, and acetoacetyl-coenzymeA synthetase, show modest expression levels (Table 2).

All homologs of the EMC pathway enzymes were expressed in *M. trichosporium* OB3b during growth on methane (Table 2). Several putative acetyl/propionyl-CoA carboxylases are predicted in the genome, however only METTOv1_200020 (putative *ppcA*) and METTOv1_220035 (putative *ppcB*) were expressed. Furthermore, the PpcAB and crotonyl-CoA reductase (*ccr*) genes display the highest level of expression among all CO_2_-fixing enzymes in the *M. trichosporium* OB3b transcriptome. Thus, the transcriptional profile of *M. trichosporium* OB3b indicates the methanotroph may possess an active EMC pathway.

### C_1_-assimilation: TCA cycle and anaplerotic CO_2_-fixation

All previous enzymatic studies predict that the tricarboxylic acid cycle (TCA cycle) in alphaproteobacterial methanotrophs is complete (Trotsenko and Murrell, 2008). However, the functional role of this metabolic pathway in methanotrophs is not fully understood. It has been suggested that the main role of the TCA cycle in the methanotrophs is carbon assimilation rather than energy production, due to low enzyme activity and lack of pyruvate dehydrogenase (Trotsenko, 1976; Anthony, 1982; Shishkina and Trotsenko, 1982; Trotsenko and Murrell, 2008). However, labeling studies on acetate and pyruvate utilization have predicted the presence of a catabolic TCA cycle in type II methanotrophs (Wadzink and Ribbons, 1975; reviewed in Higgins et al., 1981). *In silico* genome analysis indicates that *M. trichosporium* OB3b contains predicted genes for all key enzymes of the TCA cycle and pyruvate dehydrogenase (*pdh*). All of these genes were expressed (Table 2). These steps of the TCA cycle are shared between the EMC pathway and the serine cycle (Figure 1). *De novo* transcriptome assembly indicated that *M. trichosporium* OB3b possesses an additional homolog of succinate:ubiquinone oxidoreductase (*sucABCD*, Table S1 in Supplementary Material). Genes encoding the succinate:ubiquinone oxidoreductase and succinyl-CoA synthase (*sucCD*) are among the most highly expressed TCA cycle functions. The reductive branch of the pathway (including genes for citrate synthase, aconitase, isocitrate dehydrogenase, and 2-ketoglutarate dehydrogenase) displays moderate-to-low expression. Low expression of *pdh* genes is consistent with the previous enzymatic studies that show low/no activity of pyruvate dehydrogenase (Trotsenko, 1976).

It has been shown that the CO_2_-fixation potential is maximal during early stages of logarithmic growth (Park et al., 1991, 1992). However, data on carboxylation system(s) in *M. trichosporium* OB3b are controversial. Most previous enzymatic studies predict that the PEP carboxylase (Ppc), a key enzyme of the serine cycle, is the major entry point for CO_2_ in alphaproteobacterial methanotrophs (Shishkina and Trotsenko, 1982). On the other hand Naguib (1979) has shown that *M. trichosporium* OB3b possesses different carboxylation systems, including membrane bound and cytoplasmic enzymes. It could be predicted that the EMC pathway also contributes to CO_2_ assimilation. *In silico* analysis of the genome sequence also revealed that in addition to the CO_2_-fixing functions described above, genes for NAD(P)-dependent malic enzyme (*mae*), acetyl-CoA carboxylase (*accABD*), phosphoribosyl aminoimidazole carboxylase, pyruvate carboxylase (*pcx*), and a putative 2-oxoacid ferredoxin oxidoreductase are all present. All of these genes were expressed (Table 2).

### Glycolysis/gluconeogenesis and pentose-phosphate pathways

The absence of enzymatic activity for the initial steps of the gluconeogenic pathway including pyruvate-PEP or oxaloacetate-PEP conversions was one of the most common explanations for the inability of alphaproteobacterial methanotrophic bacteria to grow on poly-carbon compounds such as pyruvate or acetate (Patel et al., 1979; Shishkina and Trotsenko, 1982). No homolog of PEP-carboxykinase was found in the *M. trichosporium* OB3b genome. However, contrary to expectations based on enzymatic inferences, a set of pyruvate-acetyl-CoA, pyruvate-PEP, and pyruvate-malate interconversions could be predicted from the genome annotation. Homologs of PEP synthase (*pps*), pyruvate kinase (*pyk*1 and *pyk*2), and pyruvate phosphate dikinase were detected. The relative abundances of *pyk1*, *pps*, and *pdk* transcripts were low, but a second pyruvate kinase (*pyk*2) displayed modest expression (Table 2).

During growth on C_1_ compounds (methane or methanol), gluconeogenesis starts with conversion of 2-phosphoglycerate into 3-phosphoglycerate by phosphoglycerate mutase (*pgm*). This metabolic step represents the main branch point of the serine cycle. Four homologs of *pgm* were identified in the *M. trichosporium* OB3b genome. Three of them (METTOv1_10180, METTOv1_100061, and METTOv1_680013) were expressed at tested conditions. Homologs of the genes for the rest of the enzymes in the pathway were detected in the genome, mostly in single copies. All gene transcripts were observed in the RNA-Seq data (Table 2).

The genome analysis suggests that the pentose-phosphate pathway (PPP) is incomplete in *M. trichosporium* OB3b. Glucose-6-phosphate dehydrogenase, gluconolactonase and phosphogluconate dehydrogenase (oxidative PPP), and transaldolase or sedoheptulose bisphosphatase (non-oxidative PPP), are missing in the genome. In addition, no homologs of the genes were detected among *de novo* assembled transcripts. The lack of the oxidative branch of the PPP is consistent with previous enzymatic data and the inability of alphaproteobacterial methanotrophs to utilize sugars. However if the non-oxidative PPP operates as a route for generation of ribose-5-phosphate for the synthesis of nucleotides, an unknown enzyme must be involved in the sedoheptulose-phosphate interconversion. One possible system is a pyrophosphate-dependent phosphofructokinase (Pfk). It has been shown that Pfks from methanotrophic bacteria have surprisingly high affinity for sedoheptulose phosphate, and can catalyze the conversion of sedoheptulose-1,7-bisphosphate to sedoheptulose-7-phosphate (Reshetnikov et al., 2008; Rozova et al., 2012). It is possible that Pfk contributes to sedoheptulose-1,7-bisphosphate conversion in *M. trichosporium* OB3b.

### Lipid metabolism

Methane oxidation via particulate methane monooxygenase is linked to formation of extensive intracellular membranes. It has been shown that lipid/biomass content of *M. trichosporium* OB3b cells grown on methane is 9.2% and that phospholipids represent a significant fraction of membrane lipids (83.4%) (Weaver et al., 1975; Guckert et al., 1991). Concurrent with previous observation, genes essential for biosynthesis of major fatty acids (stearic, oleic, and palmitoleic acids) and phospholipids (including phosphotidylcholine, phosphatidylglycerol, phosphatidylserine, and phosphatidylethanolamine) show moderate level of expression (Table S2 in Supplementary Material).

### Nitrogen, copper, and iron metabolism

The pathways of nitrogen assimilation have been studied in a number of obligate methanotrophic species including *M. trichosporium* OB3b. Methanotrophs are able to grow with ammonia, nitrate, and molecular nitrogen as N-sources (Whittenbury et al., 1970; Shishkina and Trotsenko, 1979; Murrell and Dalton, 1983a,b; Chu and Alvarez-Cohen, 1998; Kim and Graham, 2001). No activities of alanine or glutamate dehydrogenases were detected in cell extracts of *M. trichosporium* OB3b grown on any source of nitrogen (Shishkina and Trotsenko, 1979; Murrell and Dalton, 1983a,b). It has been concluded that ammonia was assimilated exclusively via the glutamine synthetase/glutamate synthase pathway (Murrell and Dalton, 1983b).

In this study, cells of *M. trichosporium* OB3b were grown using nitrate as the N-source. Despite the presence of an exogenous source of nitrogen, some (very low) expression of the nitrogenase gene cluster was observed. Relative expression of nitrogenase structural genes (*nifHDK*) was about four to five times higher than expression of the chaperone and cofactor biosynthesis genes (Table S2 in Supplementary Material). High expression of genes involved in assimilatory nitrate reduction, including the nitrate transporter (*nrtA*), nitrate reductase (*nasA*), and nitrite reductase (*nasDE*) was detected. Interestingly, moderate expression of a putative ammonium transporter (METTOv1_130049) was detected, although a gene cluster with putative involvement in hydroxylamine detoxification (METTOv1_230076-78), which should only be needed under conditions of high ammonium concentration, showed low expression (Table 2). The gene encoding cytochrome *c*′-alpha (METTOv1_560023), a protein implicated in NOx detoxification, was moderately expressed. Homologs of alanine dehydrogenase (METTOv1_280018) and glutamate dehydrogenase (METTOv1_190023) were identified; however, neither gene was expressed (Table 2). High expression of glutamate synthase (both NADH and Fd-dependent), glutamate-ammonia ligase (METTOv1_200046), and Type I glutamine synthetase (METTOv1_200048) was observed. Based on these transcriptomic studies and genome analysis, the only pathway for alanine biosynthesis is transamination of pyruvate. The most likely enzymatic system for this conversion is the serine-glyoxylate aminotransferase (Sga), which is known to catalyze serine-pyruvate transamination (Liepman and Olsen, 2001). It has been shown that alanine may serve as alternative substrate for SGA in methylotrophs (Karsten et al., 2001).

Copper is an important microelement in the physiology of methanotrophic bacteria possessing pMMO (Anthony, 1982). Methanotrophic bacteria synthetize methanobactin (Mb), a copper-chelating compound (Kim et al., 2004; Balasubramanian and Rosenzweig, 2008; Semrau et al., 2010). It has been suggested that Mb provides copper for the regulation and activity of methane oxidation machinery in methanotrophs (Balasubramanian et al., 2010; Semrau et al., 2010). It has been shown that Mb is a peptide-derived molecule. A gene encoding the Mb precursor in *M. trichosporium* OB3b has recently been identified (Krentz et al., 2010). We found that the Mb-gene is among the top 5% most abundant transcripts despite the fact that the culture in our experiments was grown with sufficient Cu (Table 2). It is not known how Mb is synthesized or cleaved, however it has been suggested that genes downstream of *mb* are involved (Krentz et al., 2010). These genes were also expressed, but the expression level was low.

Iron is another essential metal in C_1_-metabolism. It has been previously observed that *M. trichosporium* OB3b can produce a Fe-chelating compound (Yoon et al., 2010); however the siderophore structure and pathways for its biosynthesis remain to be discovered. The production of a fluorescent compound was observed on plates at tested growth conditions (data not shown). Our transcriptomic studies revealed relatively high expression of genes homologous to those involved in pyoverdine (*pvd*) I biosynthesis, excretion, uptake, and regulation (Table 2), making this a possibility for a siderophore. All four essential non-ribosomal peptide synthetases (*pvdLIJD*) were identified. Unfortunately, the *pvd* genes are represented in fragments in the current genome assembly, making it impossible to predict the order of the amino acids in the peptide product.

## Conclusion

In this work we performed genomic- and transcriptomic-based reconstruction of the central metabolic pathways in *Methylosinus trichosporium* OB3b grown on methane as a sole source of carbon and energy. The overview of the methane metabolism is summarized in Figure 1. While some metabolic functions correlate well with previous enzymatic and genetic studies, several novel functions were detected and characterized. The major outcomes of our work are listed below:

1. Exceptionally high expression of pMMO in comparison to other central pathway functions (such as methanol or formaldehyde oxidation) implies a relatively low turn over at the first step of methane conversion.
2. We propose that *M. trichosporium* OB3b uses the EMC variant of the serine cycle for carbon assimilation. In addition to carbon fixing reactions of the EMC-serine cycle, a number of carboxylation reactions are predicted. The role of CO_2_ fixation during methanotrophic growth has further been explored by Yang et al. (2013).
3. The diversity of predicted reactions at the PEP-pyruvate-oxaloacetate node suggests that metabolic interconversions may play an important role in the distribution of carbon flux between the serine cycle, EMC pathway, and TCA cycle. In *M. trichosporium* OB3b the PEP-oxaloacetate conversion is predicted to be performed by two enzymatic systems under different metabolic control. Increases in the intracellular pools of malate, aspartate, and acetyl-CoA could activate flow of C_1_-derived carbon into gluconeogenesis and/or pyruvate. Our results indicate that multiple PEP-pyruvate conversion reactions may be taking place in the strain during growth on methane as a way to regenerate energy and to provide pyruvate for biosynthesis. Due to the lack of PEP-carboxykinase, the PEP synthesis from C_4_ compounds is also possible and could be achieved via decarboxylation of malate (MalE). Two reactions are predicted for PEP synthesis from pyruvate, however both of them seem to be of minor importance during growth on methane.
4. A number of transamination reactions contribute to carbon partitioning and nitrogen assimilation. It has been predicted that the growth of majority of alphaproteobacterial methylotrophic bacteria is NAD(P)H-limited due to the high NADH-requirements for formaldehyde assimilation via serine cycle (Anthony, 1978). Biosynthesis of key amino acids (such as alanine, glutamate and aspartate) via transamination seems to be a rational metabolic compensation to NAD(P)H-limitation.
5. While copper acquisition is quite well characterized in *M. trichosporium* OB3b, relatively little is known about iron uptake systems. Transcriptomic data provide initial evidence for siderophore production in this methanotroph.

## Materials and Methods

### Strain and cultivation conditions

*Methylosinus trichosporium* strain OB3b was kindly provided by Dr. Lisa Stein. The culture was grown in 250 mL glass bottles on modified NMS medium (Whittenbury et al., 1970) containing (per liter of distilled water): 1 g·KNO_3_, 1 g·MgSO_4_·7H_2_O, 0.134 g·CaCl_2_·2H_2_O, 0.25 g·KH_2_-PO, 0.7 g·Na_2_HPO_4_·12H_2_O, and 2 mL of trace elements solution. The trace elements solution contained 0.5 g·Na_2_-EDTA, 1.0 g·FeSO_4_·7H_2_O, 0.75 g·Fe-EDTA, 0.8 g·ZnSO_4_·7H_2_O, 0.005 g·MnCl_2_·4H_2_O, 0.03 g·H_3_BO_3_, 0.05 g·CoCl_2_·6H_2_O, 0.4 g·Cu-EDTA, 0.6 g·CuCl_2_·2H_2_O, 0.002 g·NiCl_2_·6H_2_O, and 0.05 g·Na_2_MoO_4_·2H_2_O. The bottles were sealed with rubber stoppers and aluminum caps, then 50 mL of methane was added to the 200 mL headspace. Bottles were shaken at 250 RPM at 30°C for 1–4 days.

### Growth parameters and methane consumption rate measurements

Methane consumption rates and cell density (OD_600_) were measured in triplicate as cultures grew. Methane measurements were made on a Shimadzu Gas Chromatograph GC-14A, using the FID detection with helium as the carrier gas. Concentrations were deduced from standard curves. OD_600_ was measured on a Beckman DU^®^ 640B spectrophotometer in plastic 1.5 mL cuvettes with a 1 cm path length.

### RNA-seq

Two replicate cultures were grown to mid exponential phase (OD_600_ 0.29 ± 0.01) for approximately 24 h, then collected by pouring 45 mL of culture into 50 mL tubes containing 5 mL of *stop solution* comprised of 5% water-equilibrated phenol, pH 6.6 (Sigma; St. Louis, MO, USA), and 95% ethanol (200 Proof; Deacon Labs, Inc., King of Prussia, PA, USA). The cells were collected by centrifugation at 4,300 × *g* at 4°C for 10 min. The resultant pellet was re-suspended in 0.75 mL of extraction buffer [2.5% CTAB (Sigma; St. Louis, MO, USA), 0.7 M NaCl, and 0.075 M pH 7.6 phosphate buffer] and transferred to a 2 mL sterilized screw-cap tube containing 0.75 mL of phenol:chloroform:isoamylic alcohol with a volume ratio of 25:24:1 (Ambion^®^; Austin, TX, USA), 0.5 g of 0.1 mm silica beads (Biospec products; Bartlesville, OK, USA), 0.2% SDS (Ambion^®^; Austin, TX, USA), and 0.2% lauryl sarkosine (Sigma; St. Louis, MO, USA). The mixtures were homogenized in a bead beater (Mini-Beadbeater; Biospec Products; Bartlesville, OK, USA) for 2 min (75% of the maximum power). The resulting slurry was centrifuged for 5 min at 4°C and 20,800 × *g*. The aqueous layer was transferred to a fresh tube containing 0.75 mL of chloroform:isoamylic alcohol with a volumetric ratio of 24:1 (Sigma; St. Louis, MO, USA) and centrifuged again for 5 min at 4°C and 20,800 × *g* to remove dissolved phenol. The aqueous phase was transferred to a new tube. MgCl_2_ (final concentration 3 mM), sodium acetate (10 mM, pH 5.5), and 0.8 mL icecold isopropanol were added. Nucleic acids were transferred to −80°C for overnight precipitation. Precipitated samples were centrifuged for 45 min at 4°C and 14,000 RPM (20,800 × *g*), washed with 0.5 mL of 75% ethanol (made from 200 proof; Deacon Labs, Inc., King of Prussia, PA, USA), and dried for 15 min at room temperature.

An RNeasy Mini Kit (Qiagen©; Venlo, Netherlands) with two types of DNA digestions was used to isolate the mRNA. Initially, the DNA/RNA pellet was re-suspended in 80 mL of a DNase I (RNase-free) mixture (Ambion^®^; Austin, TX, USA) and incubated for 30 min at 37°C. Then, the samples were purified on RNeasy Mini Kit columns as described in the *RNA cleanup* section of the manual, including the optional on-column DNAse digestion. The MICROBExpress™ (Ambion^®^; Austin, TX, USA) kit was applied to each sample to reduce the rRNA concentration and increase the mRNA sequencing depth.

The sample quality was monitored with three techniques: (1) by electrophoresis in TAE buffer in 1% agarose gels (2) using an Agilent 2100 Bioanalyzer with Agilent RNA 6000 Nano-kit as suggested by the manufacturer, and (3) by real-time reverse-transcriptase PCR (RT-RT PCR) with 16S rRNA (27F/536R) and *pmoA-*specific (Auman et al., 2001) primers.

### Transcript sequencing, alignment, and mapping

Enriched RNA samples (i.e., two biological replicates) were submitted to the University of Washington’s High-Throughput Sequencing Solutions Center on dry ice for single-read Illumina^®^ sequencing (Department of Genome Sciences, University of Washington^2^). The replicates were aligned to the reference genome using BWA under default parameters (Li and Durbin, 2009). The “METTOv1” genome sequence was downloaded from MaGE (Vallenet et al., 2006). The single large pseudo scaffold distributed by MaGE was split into 187 separate contigs at each stretch of *N* bases. In addition, chimeric contigs from the assembly and low quality gene calls were removed (Table S5 in Supplementary Material). The summary of RNA-seq (Illumina) reads can be found in Table S4 in Supplementary Material. The METTOv1 genome did not have a complete *pmoCAB* cluster suitable for alignment. In order to include *pmoCAB*, a separate alignment run was performed with the previously published sequence of this gene cluster (Holmes et al., 1995). After the alignment with BWA, SAM tools was used to generate a *pileup* file that was loaded into a MySQL database for normalization from Reads Per Kilobase of gene per Million mapped reads (RPKM) to coding sequences (Mortazavi et al., 2008).

### Transcription site mapping and transcriptome based gene assembly

The reads mapped at each base position in the genomic scaffolds generated from the *pileup* were manually examined to identify putative transcription starts and stops. Briefly, reads mapped per base data for the two replicates were plotted on a log scale. The boundary of a rapid transition from near zero reads mapped to 10, 100 RPKM or more that was upstream of a gene start was designated a transcription start. Stops were similarly identified as a rapid transition to low numbers of reads mapped downstream of a gene termination codon.

Given the fragmented nature of the *M. trichosporium* OB3b genome, we performed *de novo* assembly of the RNA-Seq reads, in an attempt to identify transcripts whose genomic sequences were incomplete. The assembly was performed with Velvet 1.2.06 (Zerbino and Birney, 2008) and Oases 0.2.08 (Schulz et al., 2012). The oases pipeline tool distributed with Oases was used to survey assemblies across the range of odd k-mers from 17 to 35 where the minimum fragment length was set to 100 bp. The final merged assembly from the pipeline tool was stripped down to the highest confidence transcript for each locus with confidence ties resolved by taking the longest sequence. The high confidence assembled transcripts were aligned to the *M. trichosporium* OB3b scaffolds using BLASTn. Transcripts without significant matches were aligned with BLASTx to the protein non-redundant database, as retrieved on January 13, 2012.

## Conflict of Interest Statement

The authors declare that the research was conducted in the absence of any commercial or financial relationships that could be construed as a potential conflict of interest.

## Supplementary Material

The Supplementary Material for this article can be found online at http://www.frontiersin.org/Microbiological_Chemistry/10.3389/fmicb.2013.00040/abstract

Supplementary Figure S1 **RNA-Seq reads mapped per base relative to start of pmo-operon**. Left, the average number of reads mapped to the region −330 to −270 upstream from the start of *pmoC* for two biological replicates. The sequence at each base is shown above the bars. Right, the range (shown in red) and mean of two biological replicates (shown as black line) for the number of reads mapped per base for the coding and downstream region of the *pmo*-operon.

Supplementary Figure S2 **Genetic organization and relative expression (RPKM) of the mxa gene cluster (A), the pqq gene cluster (B) and cluster of genes encoding reactions of the H_4_MPT-linked C1 transfer pathway (C)**.

Supplementary Figure S3 **RNA-Seq reads mapped per base relative to start of *mxaF* ORF**. Left, the average number of reads mapped to the region −200 to −140 upstream from the start of *mxaF* for two biological replicates. The sequence at each base is shown above the bars. Right, the range (shown in red) and mean of two biological replicates (shown as black line) for the number of reads mapped per base for the coding and downstream region of the *mxaF* cluster.

Supplementary Figure S4 **RNA-Seq reads mapped per base relative to start of serine cycle gene operon**. The log10 average number of reads mapped at each base from biological replicates one and two is shown. In **(A)**, the upstream location spanning −250 to −190 from putative start of ftfL. **(B)** The expression over the entire operon. Note the several drop to near zero upstream indicating the operon is not co-transcribed. **(C)** The −230 to −170 region upstream from sga. Bases from the +strand are shown across the top of the figure. **(D)** The −270 to −210 region upstream of *mclA*.

Supplementary Figure S5 **RNA-Seq reads mapped per base relative to start of *fae1-1***. Left, the average number of reads mapped to the region −235 to −185 upstream from the start of *fae1-1* gene for two biological replicates. The sequence at each base is shown above the bars. Right, the range (shown in red) and mean of two biological replicates (shown as black line) for the number of reads mapped per base for the coding and downstream region of the *fae1-1* and *fae1-2* genes.

Supplementary Figure S6 **Primary structure alignment for phosphoenolpyruvate carboxylases (Ppc1 and Ppc2) from *M. trichosporium* OB3b and Ppc-homologs**.

Supplementary Table S1 **Transcripts detected by de novo assembly RNA-Seq data**.

Supplementary Table S2 **Gene expression profile in methane-grown cells of *M. trichosporium* OB3b**. Values represent reads per kilobase of coding sequence per million (reads) mapped (RPKM).

Supplementary Table S3 **Summary of putative transcription site mapping (Note that the published sequences need references added)**.

Supplementary Table S4 **Summary of RNA-seq (Illumina) reads**.

Supplementary Table S5 **Genes removed from reference scaffold before alignment**.

## Footnotes

^1^https://www.genoscope.cns.fr/agc/microscope/mage

^2^http://www.htseq.org/
