# Supplementary figures and images for: Global Molecular Analyses of Methane Metabolism in Methanotrophic Alphaproteobacterium, Methylosinus trichosporium OB3b. Part I: Transcriptomic Study
Source: Front Microbiol. 2013 Apr 3;4:40. doi: 10.3389/fmicb.2013.00040 (PMC3615186; doi:10.3389/fmicb.2013.00040)

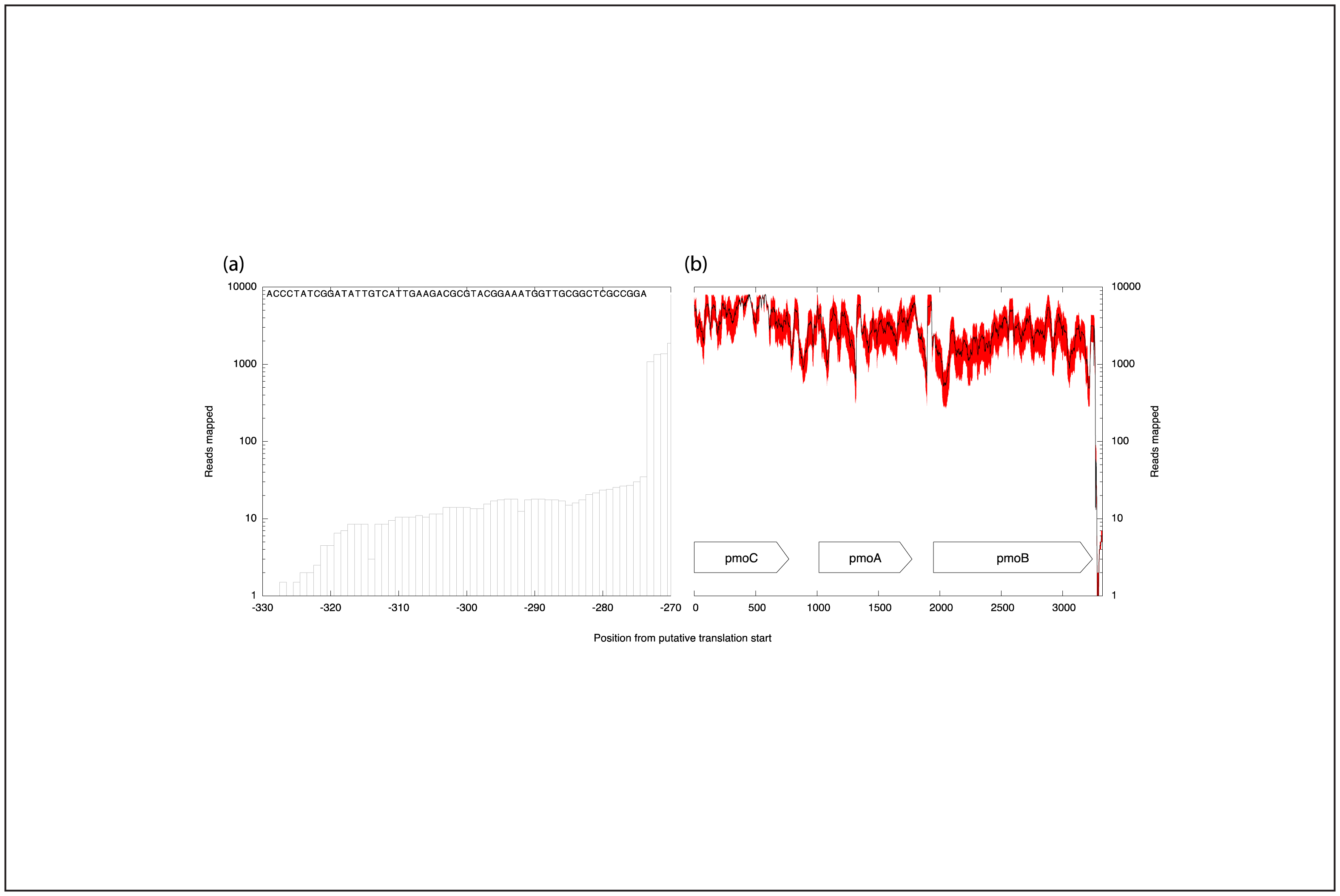

Supplement: Supplementary Figure S1 — RNA-Seq reads mapped per base relative to start of pmo-operon. Left, the average number of reads mapped to the region −330 to −270 upstream from the start of pmoC for two biological replicates. The sequence at each base is shown above the bars. Right, the range (shown in red) and mean of two biological replicates (shown as black line) for the number of reads mapped per base for the coding and downstream region of the pmo-operon. [file Figure_1.TIF]

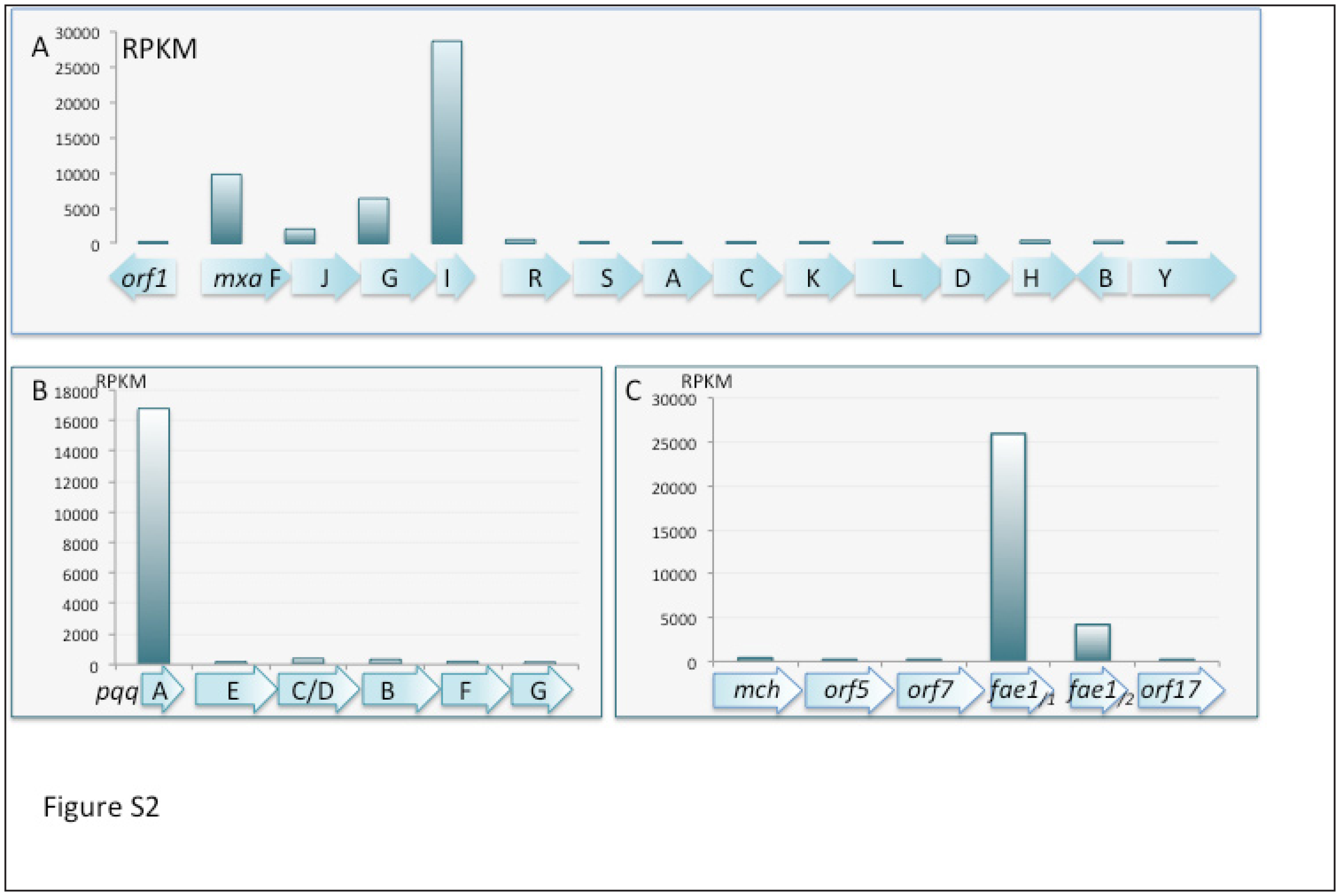

Supplement: Supplementary Figure S2 — Genetic organization and relative expression (RPKM) of the mxa gene cluster (A), the pqq gene cluster (B) and cluster of genes encoding reactions of the H4MPT-linked C1 transfer pathway (C). [file Figure_2.TIF]

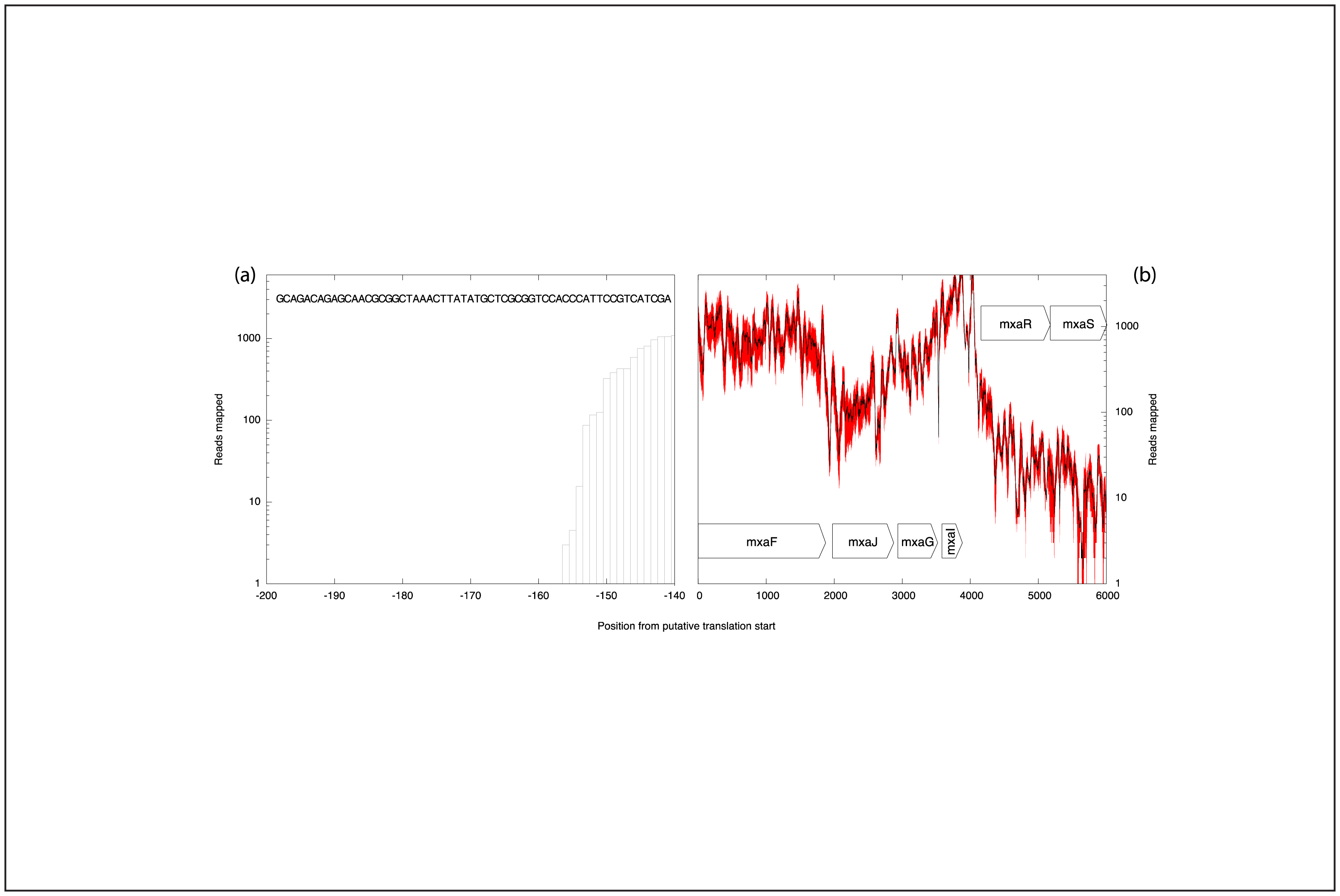

Supplement: Supplementary Figure S3 — RNA-Seq reads mapped per base relative to start of mxaF ORF. Left, the average number of reads mapped to the region −200 to −140 upstream from the start of mxaF for two biological replicates. The sequence at each base is shown above the bars. Right, the range (shown in red) and mean of two biological replicates (shown as black line) for the number of reads mapped per base for the coding and downstream region of the mxaF cluster. [file Figure_3.TIF]

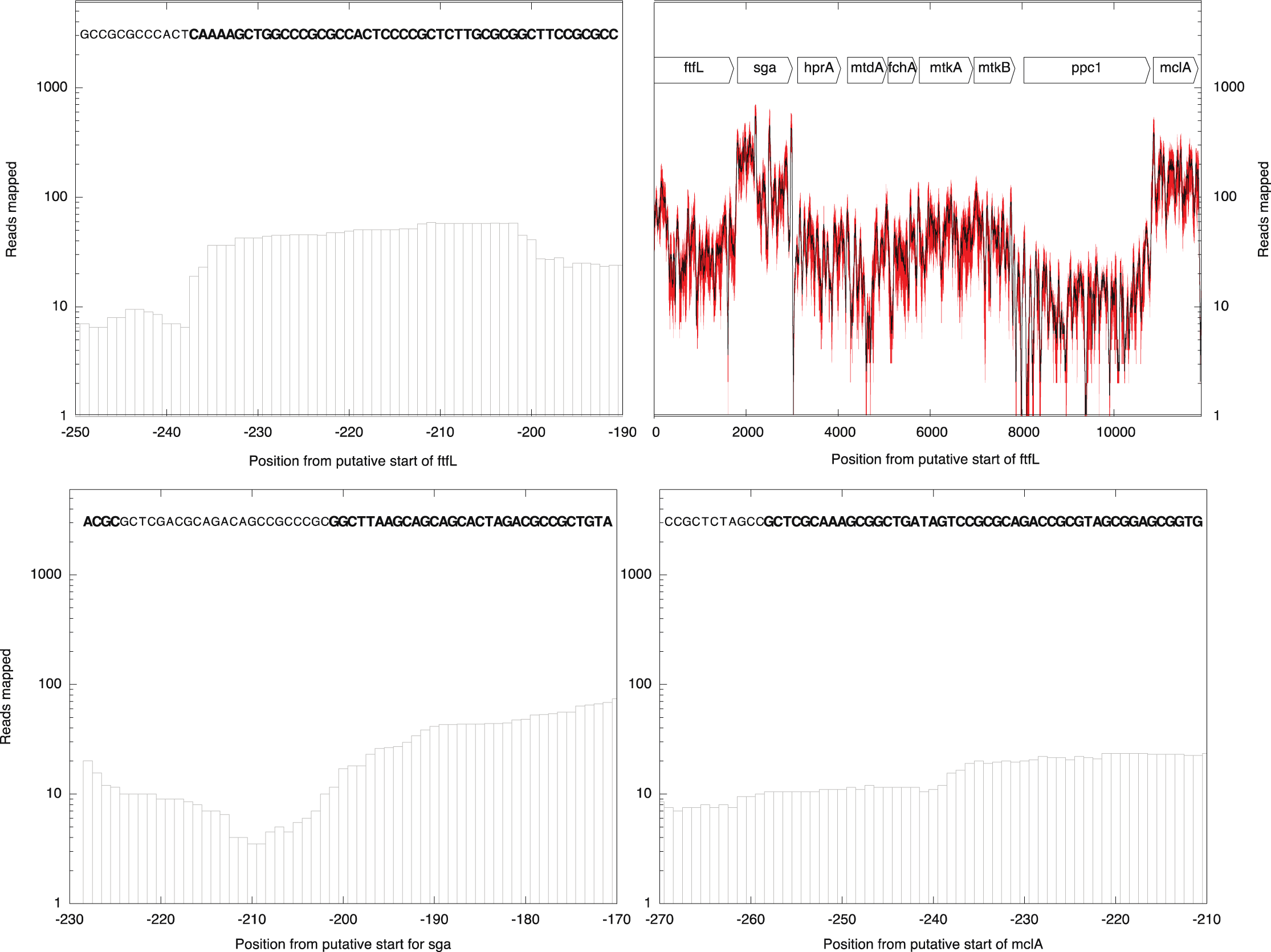

Supplement: Supplementary Figure S4 — RNA-Seq reads mapped per base relative to start of serine cycle gene operon. The log10 average number of reads mapped at each base from biological replicates one and two is shown. In (A), the upstream location spanning −250 to −190 from putative start of ftfL. (B) The expression over the entire operon. Note the several drop to near zero upstream indicating the operon is not co-transcribed. (C) The −230 to −170 region upstream from sga. Bases from the +strand are shown across the top of the figure. (D) The −270 to −210 region upstream of mclA. [file Figure_4.TIF]

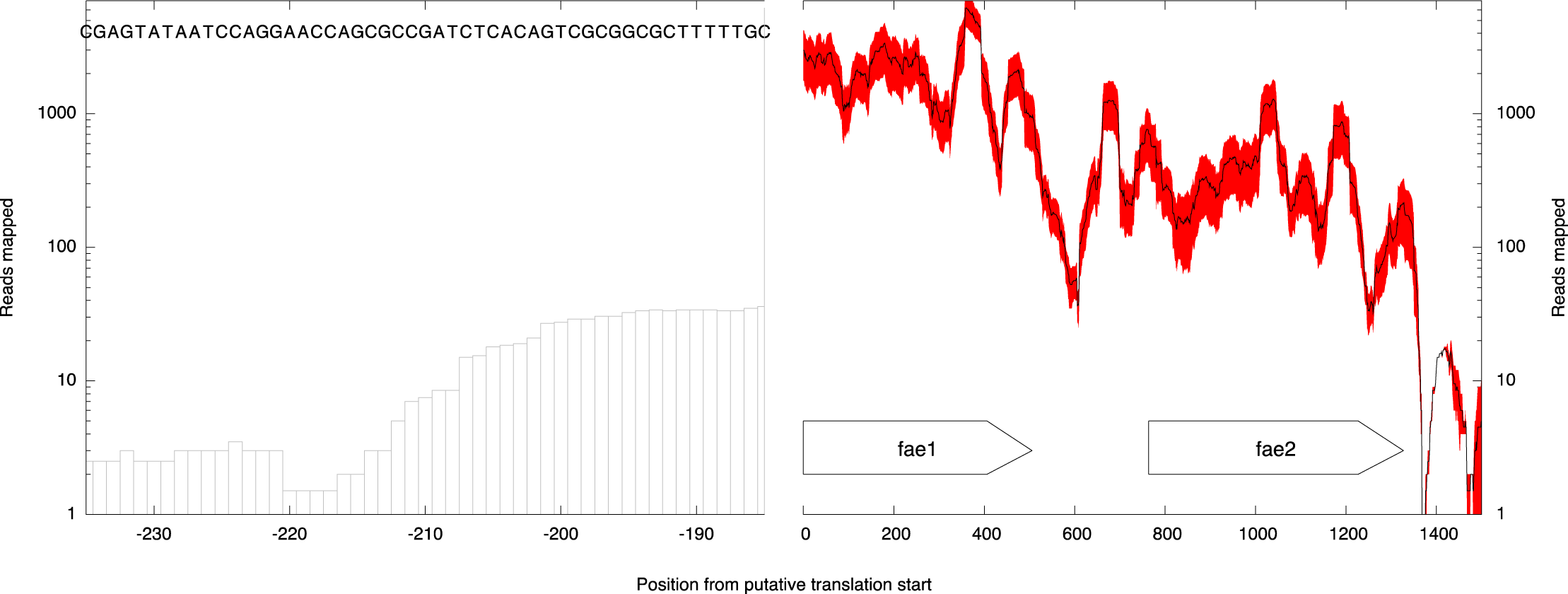

Supplement: Supplementary Figure S5 — RNA-Seq reads mapped per base relative to start of fae1-1. Left, the average number of reads mapped to the region −235 to −185 upstream from the start of fae1-1 gene for two biological replicates. The sequence at each base is shown above the bars. Right, the range (shown in red) and mean of two biological replicates (shown as black line) for the number of reads mapped per base for the coding and downstream region of the fae1-1 and fae1-2 genes. [file Figure_5.TIF]

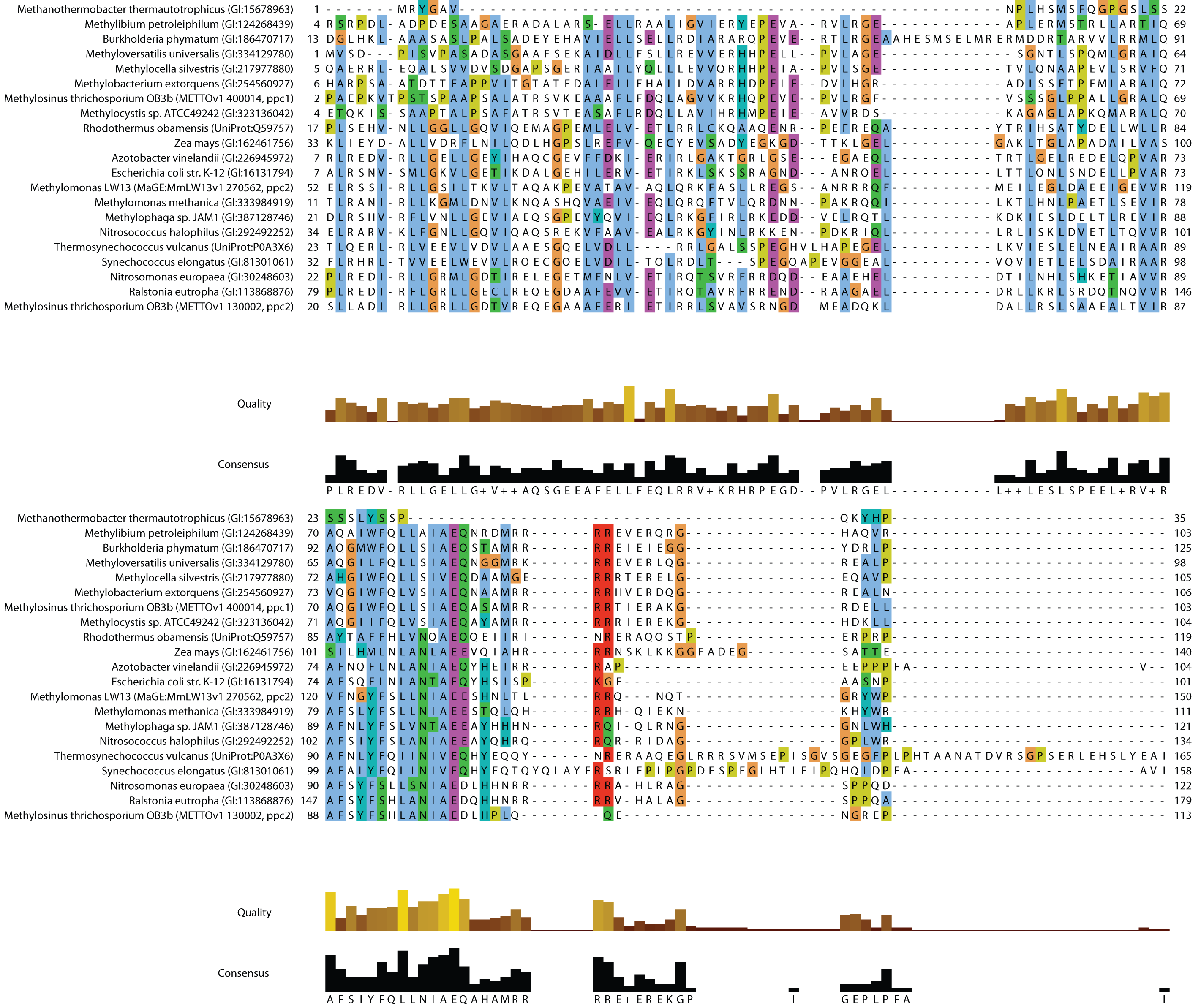

Supplement: Supplementary Figure S6 — Primary structure alignment for phosphoenolpyruvate carboxylases (Ppc1 and Ppc2) from M. trichosporium OB3b and Ppc-homologs. [file Figure_6.TIF]
