# Supplementary material for: Global Molecular Analyses of Methane Metabolism in Methanotrophic Alphaproteobacterium, Methylosinus trichosporium OB3b. Part I: Transcriptomic Study
Source: Front Microbiol. 2013 Apr 3;4:40. doi: 10.3389/fmicb.2013.00040 (PMC3615186; doi:10.3389/fmicb.2013.00040)
Supplement: Supplementary Table S1 — Transcripts detected by de novo assembly RNA-Seq data. [file Data_Sheet_1.PDF]

Table S1. Transcripts detected by *de novo* assembly RNA-Seq data

ID	Non-redundant	Description	Organism	Non-redundant length	Fraction of nr covered by alignment	Alignment % identity	Alignment e-value
8	319794846	hypothetical protein Varpa_4205	Variovorax paradoxus EPS	90	0.89	63	1.00E-23
48	323139488	SEC-C motif domain protein	Methylocystis sp. ATCC 49242	169	0.20	55	0.0004
48	323139488	SEC-C motif domain protein	Methylocystis sp. ATCC 49242	169	0.41	63	4.00E-18
90	307110660	hypothetical protein CHLNCDRAFT_50435	Chlorella variabilis	1535	0.05	24	4.5
90	307110660	hypothetical protein CHLNCDRAFT_50435	Chlorella variabilis	1535	0.05	24	4.6
120	309365749	hypothetical protein CBG_01089	Caenorhabditis briggsae AF16	812	0.12	32	0.7
121	310821854	hypothetical protein STAUR_4605	Stigmatella aurantiaca DW4/3-1	728	0.08	38	3.5
125	197103309	hypothetical protein PHZ_p0169	Phenylobacterium zucineum HLK1	223	0.55	60	8.00E-34
142	78213547	glycosyltransferase	Synechococcus sp. CC9605	1003	0.31	31	5.00E-14
142	325116388	tRNA uridine 5-carboxymethylaminomethyl modification enzyme GidA, related	Neospora caninum Liverpool	819	0.21	31	0.088
171	323136696	hypothetical protein Met49242DRAFT_1164	Methylocystis sp. ATCC 49242	92	0.64	53	6.00E-06
266	338532669	chloride channel	Myxococcus fulvus HW-1	628	0.72	63	3.00E-138
300	323136327	Patatin	Methylocystis sp. ATCC 49242	284	0.44	60	3.00E-38
303	227819692	transposase Y4ZB	Sinorhizobium fredii NGR234	493	0.35	42	8.00E-24
308	323449050	hypothetical protein	Aureococcus	1445	0.48	25	3.00E-33

		AURANDRAFT_66784	anophagefferens				
308	323449050	hypothetical protein AURANDRAFT_66784	Aureococcus anophagefferens	1445	0.48	25	3.00E-33
308	323449050	hypothetical protein AURANDRAFT_66784	Aureococcus anophagefferens	1445	0.48	25	3.00E-33
314	341615616	two-component response regulator	Citromicrobium sp. JLT1363	257	0.15	59	0.32
339	149918868	site-specific recombinase, phage integrase family protein	Plesiocystis pacifica SIR-1	120	0.53	36	7.9
360	46153	endo-glucanase	Ruminococcus flavefaciens	680	0.09	38	4.6
370	167901659	hypothetical protein BpseN_05228	Burkholderia pseudomallei NCTC 13177	474	0.32	29	4.9
381	203288753	BdrQ-like protein	Borrelia duttonii Ly	239	0.67	30	4.00E-05
401	323457221	hypothetical protein AURANDRAFT_70536	Aureococcus anophagefferens	1999	0.06	33	2.1
431	221486147	conserved hypothetical protein	Toxoplasma gondii GT1	702	0.16	30	5.9
469	16519794	transposase number 3 of uncharacterized insertion sequence	Sinorhizobium fredii NGR234	511	0.31	62	7.00E-51
518	323135994	2-oxoglutarate dehydrogenase, E2 subunit, dihydrolipoamide succinyltransferase	Methylocystis sp. ATCC 49242	410	0.10	68	0.0002
523	332821691	PREDICTED: hypothetical protein LOC100609280	Pan troglodytes	329	0.43	28	0.92
542	299133059	Pseudomurein-binding repeat protein	Afipia sp. 1NLS2	686	0.28	96	2.00E-102
542	299133059	Pseudomurein-binding repeat protein	Afipia sp. 1NLS2	686	0.17	94	3.00E-59
569	118340377	putative granule bound starch synthase	Sorghum bicolor	608	0.08	45	5.9
570	316932709	RNA-binding S4 domain-containing protein	Rhodopseudomonas palustris DX-1	717	0.06	41	4.7

570	325116236	putative retinitis pigmentosa GTPase regulator	Neospora caninum Liverpool	1413	0.04	43	0.41
582	255714473	KLTH0E00704p	Lachancea thermotolerans	1389	0.03	42	2.6
712	25029285	hypothetical protein CE2729	Corynebacterium efficiens YS-314	251	0.26	43	0.0003
721	88812339	succinate dehydrogenase catalytic subunit	Nitrococcus mobilis Nb-231	259	0.75	69	4.00E-72
737	222630362	hypothetical protein OsJ_17292	Oryza sativa Japonica Group	377	0.13	40	2
737	115462351	Os05g0171300	Oryza sativa Japonica Group	415	0.12	40	2
783	119174408	predicted protein	Coccidioides immitis RS	363	0.13	41	7.8
787	121710406	Ras GTPase activating protein, putative	Aspergillus clavatus NRRL 1	1671	0.04	35	5.9
788	323135945	molybdenum cofactor biosynthesis protein A	Methylocystis sp. ATCC 49242	346	0.09	87	1.00E-06
809	186510389	octicosapeptide/Phox/Bem1p domain-containing protein kinase	Arabidopsis thaliana	1117	0.09	30	2.7
810	83312534	hypothetical protein amb3435	Magnetospirillum magneticum AMB-1	86	0.38	61	0.029
810	83312534	hypothetical protein amb3435	Magnetospirillum magneticum AMB-1	86	0.42	58	0.006
823	154312862	hypothetical protein BC1G_05132	Botryotinia fuckeliana B05.10	315	0.13	44	7.9
829	304394601	conserved hypothetical protein	Ahrensia sp. R2A130	55	0.73	53	0.049
833	9631033	conotoxin-like protein	Lymantria dispar MNPV	92	0.68	38	7.9
844	298241184	conserved hypothetical protein	Ktedonobacter racemifer DSM 44963	204	0.16	55	2.1
895	198283398	XRE family transcriptional regulator	Acidithiobacillus ferrooxidans ATCC 53993	152	0.93	32	0.0004
909	315500368	transcriptional regulator, mucr	Asticcacaulis excentricus	141	1.03	57	5.00E-36

		family	CB 48				
929	23500301	IS66 family orf3	Brucella suis 1330	523	0.45	60	1.00E-70
929	288962634	transposase	Azospirillum sp. B510	420	0.29	63	6.00E-35
932	73980988	PREDICTED: similar to ALMS1	Canis familiaris	4146	0.01	42	7.9
933	307108552	expressed protein	Chlorella variabilis	838	0.18	27	0.41
954	299133061	DNA modification methyltransferase-related protein	Afipia sp. 1NLS2	917	0.16	94	8.00E-62
958	271965707	hypothetical protein Sros_4262	Streptosporangium roseum DSM 43021	238	0.20	40	6
1074	323139155	replication protein C	Methylocystis sp. ATCC 49242	444	0.60	68	1.00E-94
1083	254502301	hypothetical protein SADFL11_2339	Labrenzia alexandrii DFL-11	993	0.12	83	3.00E-43
1097	85708226	putative DNA methylase	Erythrobacter sp. NAP1	909	0.66	76	0
1128	323139416	protein of unknown function DUF188	Methylocystis sp. ATCC 49242	152	0.34	85	9.00E-17
1128	323139416	protein of unknown function DUF188	Methylocystis sp. ATCC 49242	152	0.50	87	8.00E-18
1171	323139522	hypothetical protein Met49242DRAFT_3956	Methylocystis sp. ATCC 49242	444	0.60	91	2.00E-138
1200	299133058	Putative helicase A859L	Afipia sp. 1NLS2	408	0.48	81	1.00E-89
1250	224107413	PREDICTED: hypothetical protein, partial	Taeniopygia guttata	369	0.22	33	4.6
1259	225156592	conserved hypothetical protein	Opitutaceae bacterium TAV2	275	0.17	35	3.5
1290	298293273	succinate dehydrogenase, hydrophobic membrane anchor protein	Starkeya novella DSM 506	136	0.84	54	1.00E-24
1292	194761572	GF15723	Drosophila ananassae	514	0.14	37	4.5
1302	316934586	hypothetical protein Rpdx1_3258	Rhodopseudomonas palustris DX-1	253	0.27	72	1.00E-18

1310	154495963	hypothetical protein BACCAP_00246	Bacteroides capillosus ATCC 29799	166	0.23	49	4.5
1324	323137236	DNA gyrase, B subunit	Methylocystis sp. ATCC 49242	810	0.07	81	1.00E-15
1352	195442410	GK17748	Drosophila willistoni	421	0.10	45	5.9
1357	323137126	glycosyl transferase group 1	Methylocystis sp. ATCC 49242	408	0.14	70	2.00E-14
1369	323136028	major facilitator superfamily MFS_1	Methylocystis sp. ATCC 49242	528	0.15	65	1.00E-20
1374	90420316	phosphomethylpyrimidine kinase	Aurantimonas manganoxydans SI85-9A1	317	0.06	75	5.9
1436	238023318	hypothetical protein bglu_2p0290	Burkholderia glumae BGR1	476	0.29	36	5.00E-13
1498	330966470	hypothetical protein PSYAC_17830	Pseudomonas syringae pv. actinidiae str. M302091	437	0.10	47	0.0009
1615	338820076	hypothetical protein Agau_P200245	Agrobacterium tumefaciens F2	783	0.11	47	6.00E-14
1691	332560244	Mg chelatase-related protein	Rhodobacter sphaeroides WS8N	512	0.11	38	4.5
1734	323137126	glycosyl transferase group 1	Methylocystis sp. ATCC 49242	408	0.24	77	9.00E-39
1787	299133059	Pseudomurein-binding repeat protein	Afipia sp. 1NLS2	686	0.13	99	9.00E-39
1787	299133059	Pseudomurein-binding repeat protein	Afipia sp. 1NLS2	686	0.09	98	9.00E-25
1793	323137126	glycosyl transferase group 1	Methylocystis sp. ATCC 49242	408	0.08	88	6.00E-08
1793	323137126	glycosyl transferase group 1	Methylocystis sp. ATCC 49242	408	0.08	88	6.00E-08
1836	150378510	tyrosine-protein phosphatase non-receptor type 22	Danio rerio	887	0.05	34	7.6
1856	17232905	hypothetical protein alr8532	Nostoc sp. PCC 7120	304	0.25	48	4.00E-11

1922	296534676	conserved hypothetical protein	Roseomonas cervicalis ATCC 49957	206	0.30	46	0.41
1947	163857359	LysR family transcriptional regulator	Bordetella petrii DSM 12804	295	0.15	68	7.00E-09
1953	332708113	penicillin-binding protein, family 1A	Lyngbya majuscula 3L	819	0.08	34	0.91
1961	323139158	integrase domain protein SAM domain protein	Methylocystis sp. ATCC 49242	347	0.10	74	2.00E-08
1970	154245910	succinate dehydrogenase cytochrome b556 subunit	Xanthobacter autotrophicus Py2	133	0.95	57	3.00E-32
1970	110635572	succinate dehydrogenase iron-sulfur subunit	Mesorhizobium sp. BNC1	259	0.25	88	2.00E-26
1976	197103308	hypothetical protein PHZ_p0168	Phenylobacterium zucineum HLK1	323	0.14	45	0.017
2036	291449786	LOW QUALITY PROTEIN: FscRII	Streptomyces albus J1074	880	0.11	36	3.5
2044	194733871	antirestriction protein	Salmonella enterica subsp. enterica serovar Schwarzengrund str. CVM19633	321	0.17	49	6.00E-05
2048	256377452	AraC family transcriptional regulator	Actinosynnema mirum DSM 43827	309	0.12	61	0.029
2048	256377452	AraC family transcriptional regulator	Actinosynnema mirum DSM 43827	309	0.12	61	0.029
2082	302539530	polyketide synthase type I	Streptomyces sp. C	1616	0.03	43	0.71
2087	299133058	Putative helicase A859L	Afipia sp. 1NLS2	408	0.11	82	5.00E-13
2108	159489659	predicted protein	Chlamydomonas reinhardtii	439	0.08	51	7.8
2149	296534676	conserved hypothetical protein	Roseomonas cervicalis ATCC 49957	206	0.32	38	4.6
2151	300865706	conserved hypothetical protein	Oscillatoria sp. PCC 6506	138	0.26	42	7.8
2167	209544899	hypothetical protein Gdia_2780	Gluconacetobacter diazotrophicus PAl 5	397	0.09	97	5.00E-05

2176	323138303	peptidase U62 modulator of DNA gyrase	Methylocystis sp. ATCC 49242	514	0.28	33	0.0002
2236	220922655	hypothetical protein Mnod_2696	Methylobacterium nodulans ORS 2060	173	0.23	75	6.00E-08
2392	316934586	hypothetical protein Rpdx1_3258	Rhodopseudomonas palustris DX-1	253	0.08	84	2.1
2503	323139732	putative transposase	Methylocystis sp. ATCC 49242	150	0.11	88	1.2
2606	159044846	hypothetical protein Dshi_2303	Dinoroseobacter shibae DFL 12	432	0.08	57	0.71
2614	258515430	hypothetical protein Dtox_2211	Desulfotomaculum acetoxidans DSM 771	377	0.10	74	3.00E-08
2641	125524722	hypothetical protein OsI_00707	Oryza sativa Indica Group	532	0.08	44	2.7
2690	222871727	predicted protein	Populus trichocarpa	398	0.10	46	6
2750	323136026	secretion protein HlyD family protein	Methylocystis sp. ATCC 49242	397	0.15	81	7.00E-20
2832	329889268	phosphotransferase domain-containing protein	Brevundimonas diminuta ATCC 11568	1279	0.03	54	0.01
2854	338778752	glutathione S-transferase	Achromobacter xylosoxidans AXX-A	201	0.22	73	5.00E-10
2901	323139705	hypothetical protein Met49242DRAFT_4133	Methylocystis sp. ATCC 49242	253	0.31	48	1.00E-11
2903	28897417	hypothetical protein VP0643	Vibrio parahaemolyticus RIMD 2210633	599	0.05	45	10
2975	323137126	glycosyl transferase group 1	Methylocystis sp. ATCC 49242	408	0.09	89	3.00E-11
2983	297620720	nitric oxide reductase, subunit B	Waddlia chondrophila WSU 86-1044	763	0.04	85	6.00E-10
3047	340030375	helix-turn-helix domain-containing protein	Paracoccus sp. TRP	81	0.64	60	6.00E-08
3093	302134922	hypothetical protein PsyrptN_26257	Pseudomonas syringae pv. tomato NCPPB 1108	988	0.04	53	0.92

3111	329889268	phosphotransferase domain-containing protein	Brevundimonas diminuta ATCC 11568	1279	0.03	51	0.022
3112	323139523	hypothetical protein Met49242DRAFT_3957	Methylocystis sp. ATCC 49242	482	0.09	88	7.00E-06
3123	297292023	PREDICTED: solute carrier family 22 member 3-like	Macaca mulatta	715	0.06	44	7.8
3128	334195582	putative osmotically inducible lipoprotein b1 transmembrane (osmB)	Ralstonia solanacearum Po82	296	0.17	45	3.5
3130	254500001	hypothetical protein SADFL11_39	Labrenzia alexandrii DFL-11	356	0.20	54	5.00E-12
3130	254500001	hypothetical protein SADFL11_39	Labrenzia alexandrii DFL-11	356	0.19	56	1.00E-11
3162	301780690	PREDICTED: lysine-specific demethylase 4D-like	Ailuropoda melanoleuca	487	0.08	49	7.8
3169	296123721	hypothetical protein Plim_3487	Planctomyces limnophilus DSM 3776	438	0.07	47	7.8
3190	149918280	RNA methyltransferase	Plesiocystis pacifica SIR-1	485	0.12	40	4.6
3190	149918280	RNA methyltransferase	Plesiocystis pacifica SIR-1	485	0.12	40	4.6
3191	91200065	conserved hypothetical protein	Candidatus Kuenenia stuttgartiensis	221	0.24	46	0.11
3192	302813543	hypothetical protein SELMODRAFT_427133	Selaginella moellendorffii	270	0.15	48	0.7
3236	333027859	putative ADP-ribosylation/Crystallin J1	Streptomyces sp. Tu6071	893	0.06	44	7.6
3258	110347006	hypothetical protein Meso_4194	Mesorhizobium sp. BNC1	366	0.16	62	4.00E-12
3315	288963127	transposase	Azospirillum sp. B510	267	0.25	67	2.00E-16
3340	3059133	transposase IS1355	Methylobacterium extorquens DM4	179	0.22	87	4.00E-12
3398	83592164	CRISPR-associated helicase Cas3 family protein	Rhodospirillum rubrum ATCC 11170	752	0.05	69	5.00E-05
3602	323137054	hypothetical protein	Methylocystis sp. ATCC	73	0.59	77	4.00E-09

		Met49242DRAFT_1521	49242				
3602	197103644	RNA polymerase sigma 54 subunit, RpoN	Phenylobacterium zucineum HLK1	499	0.11	44	2.7
3605	218667529	hypothetical protein AFE_2089	Acidithiobacillus ferrooxidans ATCC 23270	253	0.42	29	4.5
3656	121596846	RNA polymerase sigma factor	Burkholderia mallei SAVP1	231	0.26	51	3.00E-08
3677	299133058	Putative helicase A859L	Afipia sp. 1NLS2	408	0.27	85	2.00E-28
3742	90424677	plasmid maintenance system killer protein	Rhodopseudomonas palustris BisB18	93	0.31	41	1.4
3786	229828371	hypothetical protein GCWU000342_00430	Shuttleworthia satelles DSM 14600	298	0.15	40	7.9
3786	229828371	hypothetical protein GCWU000342_00430	Shuttleworthia satelles DSM 14600	298	0.15	40	7.7
3891	220922092	NnrS family protein	Methylobacterium nodulans ORS 2060	392	0.09	68	2.00E-05
3901	221487851	conserved hypothetical protein	Toxoplasma gondii GT1	3229	0.02	39	4.5
3981	85711049	Predicted metal-dependent amidohydrolase with the TIM-barrel fold	Idiomarina baltica OS145	558	0.23	26	3.5
3981	89067955	type I secretion target repeat protein	Oceanicola granulosus HTCC2516	818	0.17	28	4.2
4000	323136327	Patatin	Methylocystis sp. ATCC 49242	284	0.44	61	3.00E-37
4020	328541621	hypothetical protein SL003B_p0053	Polymorphum gilvum SL003B-26A1	435	0.43	58	2.00E-56
4035	108763646	putative lipoprotein	Myxococcus xanthus DK 1622	447	0.13	35	7.8
4187	288957870	transposase	Azospirillum sp. B510	460	0.09	43	5.9
4195	335035794	hypothetical protein AGRO_3125	Agrobacterium sp. ATCC 31749	323	0.09	62	0.55

4205	90424421	transposase IS3/IS911	Rhodopseudomonas palustris BisB18	66	0.38	68	1.6
4305	329928833	hypothetical protein HMPREF9412_3689	Paenibacillus sp. HGF5	694	0.05	94	1.00E-10
4311	167621675	IS66 Orf2 family protein	Caulobacter sp. K31	117	0.35	59	3.00E-05
4338	209886756	hypothetical protein OCAR_7650	Oligotropha carboxidovorans OM5	362	0.10	64	2.00E-05
4381	284989640	hypothetical protein Gobs_1064	Geodermatophilus obscurus DSM 43160	285	0.11	59	2.7
4492	13475179	hypothetical protein mlr6199	Mesorhizobium loti MAFF303099	240	0.18	72	1.00E-09
4538	238061000	5'-nucleotidase domain-containing protein	Micromonospora sp. ATCC 39149	616	0.13	35	7.4
4646	302522431	LOW QUALITY PROTEIN: ABC transporter ATP-binding protein	Streptomyces sp. SPB78	564	0.15	30	4.5
4866	299133061	DNA modification methyltransferase-related protein	Afipia sp. 1NLS2	917	0.14	90	3.00E-58
4903	299741957	endoprotease	Coprinopsis cinerea okayama7#130	613	0.07	44	4.5
4908	323139523	hypothetical protein Met49242DRAFT_3957	Methylocystis sp. ATCC 49242	482	0.08	97	2.00E-14
4992	323135620	hypothetical protein Met49242DRAFT_0090	Methylocystis sp. ATCC 49242	153	0.23	91	2.00E-11
5003	299133061	DNA modification methyltransferase-related protein	Afipia sp. 1NLS2	917	0.04	95	6.00E-14
5069	316934586	hypothetical protein Rpdx1_3258	Rhodopseudomonas palustris DX-1	253	0.39	88	1.00E-44
5103	312883142	hypothetical protein VIBC2010_07634	Vibrio caribbenthicus ATCC BAA-2122	138	0.85	38	1.00E-14
5192	16125845	sulfate ABC transporter ATP-binding protein	Caulobacter crescentus CB15	359	0.12	55	0.007

5192	86739180	ABC transporter-like protein	Frankia sp. CcI3	385	0.13	51	0.017
5210	323139955	transposase IS204/IS1001/IS1096/IS1165 family protein	Methylocystis sp. ATCC 49242	549	0.05	54	4.7
5248	170743671	putative transposase	Methylobacterium sp. 4-46	373	0.08	71	0.001
5420	323139641	protein of unknown function DUF1403	Methylocystis sp. ATCC 49242	345	0.10	61	0.9

Table S2. Gene expression profile in methane-grown cells of M. trichosporium OB3b. Values represent reads per kilobase of coding sequence per million (reads) mapped (RPKM).

GENE ID/LOCUS TAG	PUTATIVE FUNCTION	BIOLOGICAL REPLICATE 1 (RPKM)	BIOLOGICAL REPLICATE 2 (RPKM)
METTOv1_1180001	Protein of unknown function	444193	502003
METTOv1_1180006	Conserved protein of unknown function	114962	124378
METTOv1_1270004	Methane monooxygenase subunit PmoC	66689	67703
METTOv1_1270003	Protein of unknown function	56337	59538
METTOv1_1270002	Methane monooxygenase subunit PmoA	37102	31813
METTOv1_1270001	Methane monooxygenase subunit PmoB	27371	22917
METTOv1_720011	Exported protein of unknown function	25925	34114
METTOv1_240011	Methanol dehydrogenase beta subunit, MxaI	24552	28474
METTOv1_40013	Formaldehyde-activating enzyme, Fae	24353	24787
METTOv1_80041	Protein of unknown function	15629	17876
METTOv1_10085	Putative Flp/Fap pilin component (modular protein)	15210	17950
METTOv1_220005	Conserved protein of unknown function	13342	12873
METTOv1_100043	Flavodoxin FldA	12939	14717
METTOv1_10086	Putative Flp/Fap pilin component (modular protein)	12500	14508
METTOv1_pqqA	PqqA	11857	13927
METTOv1_350034	Exported protein of unknown function	10937	11059
METTOv1_10178	Conserved protein of unknown function	10911	11543
METTOv1_240014	PQQ-dependent methanol dehydrogenase, MxaF	9742	8367
METTOv1_360031	10 kDa chaperonin	8505	8095
METTOv1_50029	Conserved exported protein of unknown function	7799	8459
METTOv1_210021	Putative 31 kDa outer-membrane immunogenic protein precursor	7755	7718
METTOv1_50046	Antibiotic biosynthesis monooxygenase	7561	8400
METTOv1_220036	Protein of unknown function	6126	7735
METTOv1_380025	cold-shock DNA-binding domain protein	6056	7763
METTOv1_360030	60 kDa chaperonin	6040	5426
METTOv1_240012	Cytochrome c class I	5712	6117
METTOv1_240015	Protein of unknown function	5571	5393
METTOv1_250008	Protein of unknown function DUF465	5160	7729
METTOv1_370032	Protein of unknown function	5110	7733
METTOv1_310036	H+transporting two-sector ATPase C subunit	4898	4882
METTOv1_10101	50S ribosomal protein L7/L12	4728	4670
METTOv1_670020	Conserved protein of unknown function	4636	5951
METTOv1_90056	Putative outer-membrane immunogenic protein precursor	4497	4098
METTOv1_870006	Conserved protein of unknown function, putative MbtH-like protein	4176	5481
METTOv1_40014	Formaldehyde-activating enzyme, Fae	4024	3676
METTOv1_20142	50S ribosomal protein L34	3876	3678
METTOv1_1180005	Protein of unknown function	3865	4053
METTOv1_10102	50S ribosomal protein L10	3846	3875
METTOv1_50044	TonB-dependent heme/hemoglobin receptor family protein	3483	3631
METTOv1_60112	Protein of unknown function	3418	4716
METTOv1_680015	Cytochrome c class I	3331	3695
METTOv1_140005	Cold-shock DNA-binding domain protein	3308	3283
METTOv1_1590001	Elongation factor Tu (fragment)	3168	2796
METTOv1_150018	Conserved membrane protein of unknown function	3133	3356
METTOv1_140012	Protein of unknown function	3097	4163

METTOv1_270053	Putative outer membrane TonB-dependent receptor; putative receptor for iron transport	3058	2845
METTOv1_390020	Protein of unknown function	3058	3677
METTOv1_10003	Conserved exported protein of unknown function	3045	3088
METTOv1_60110	Flagellar hook protein FlgE	2996	3024
METTOv1_50060	Conserved exported protein of unknown function	2980	3047
METTOv1_50043	Protein of unknown function	2975	3223
METTOv1_340043	TonB-dependent siderophore receptor	2962	2659
METTOv1_10054	protein of unknown function	2885	3524
METTOv1_40026	protein of unknown function DUF88	2850	3035
METTOv1_80110	30S ribosomal protein S12	2744	2578
METTOv1_440010	ribosomal protein L29	2742	2676
METTOv1_130050	nitrogen regulatory protein P-II	2650	2735
METTOv1_710010	conserved protein of unknown function	2639	2582
METTOv1_740011	30S ribosomal protein S15	2582	2802
METTOv1_10009	Acyl carrier protein	2534	3554
METTOv1_840014	Histone family protein DNA-binding protein (fragment)	2484	2644
METTOv1_200048	Glutamine synthetase, type I	2452	2321
METTOv1_200047	Nitrogen regulatory protein P-II	2400	2277
METTOv1_440018	50S ribosomal protein L18	2297	2157
METTOv1_760004	conserved protein of unknown function	2255	2676
METTOv1_210044	exported protein of unknown function	2247	2538
METTOv1_440001	30S ribosomal protein S10	2232	2089
METTOv1_440004	Ribosomal protein L25/L23	2232	2042
METTOv1_760010	TonB-dependent receptor	2197	2174
METTOv1_250051	50S ribosomal protein L35	2195	2579
METTOv1_50068	30S ribosomal protein S13	2173	2167
METTOv1_440020	50S ribosomal protein L30	2157	1946
METTOv1_350038	50S ribosomal protein L33	2134	3246
METTOv1_60031	protein of unknown function	2120	3016
METTOv1_510015	30S ribosomal protein S1	2113	1995
METTOv1_440011	30S ribosomal protein S17	2053	2020
METTOv1_10124	30S ribosomal protein S6	2031	2505
METTOv1_130049	Ammonium transporter	2029	1766
METTOv1_430003	50S ribosomal protein L28	2025	1976
METTOv1_60030	Exported protein of unknown function	2023	2047
METTOv1_440006	30S ribosomal protein S19	2020	1922
METTOv1_20092	protein of unknown function	2013	2471
METTOv1_30061	protein of unknown function	1998	2438
METTOv1_680005	30S ribosomal protein S20	1966	1972
METTOv1_600007	Flagellin domain protein	1948	1994
METTOv1_240013	Extracellular solute-binding protein family 3	1942	1838
METTOv1_440009	50S ribosomal protein L16	1941	1692
METTOv1_50071	50S ribosomal protein L17	1893	1987
METTOv1_280037	glutaredoxin-like protein	1889	2282
METTOv1_440008	30S ribosomal protein S3	1880	1812
METTOv1_440017	50S ribosomal protein L6	1865	1673
METTOv1_180065	ATP synthase subunit alpha	1847	1622
METTOv1_400020	Serine-glyoxylate transaminase, Sga	1840	1969
METTOv1_360023	Exported protein of unknown function	1800	1818
METTOv1_440014	ribosomal protein L5	1790	1620
METTOv1_10173	30S ribosomal protein S4	1789	1786
METTOv1_420032	Glutathione peroxidase	1789	2445

METTOv1_220052	Bacterioferritin	1787	1970
METTOv1_240028	OmpW family protein	1767	1765
METTOv1_310020	Nitrite reductase (NAD(P)H), large subunit	1762	1562
METTOv1_440013	50S ribosomal protein L24	1750	1504
METTOv1_370004	Ferric uptake regulator, Fur family	1745	1828
METTOv1_310023	Major facilitator superfamily MFS_1	1743	1712
METTOv1_60068	Protein of unknown function UPF0005	1722	1670
METTOv1_400013	Malyl-CoA lyase/beta-methylmalyl-CoA lyase	1713	1615
METTOv1_50038	50S ribosomal protein L21	1691	1716
METTOv1_100044	protein of unknown function	1682	1492
METTOv1_140057	Nucleoside diphosphate kinase	1682	1500
METTOv1_440007	50S ribosomal protein L22	1674	1777
METTOv1_50037	50S ribosomal protein L27	1664	1794
METTOv1_710011	protein of unknown function	1655	1686
METTOv1_80112	Elongation factor G	1651	1691
METTOv1_10125	30S ribosomal protein S18	1647	1498
METTOv1_240035	50S ribosomal protein L25	1645	1617
METTOv1_340026	50S ribosomal protein L9	1638	1655
METTOv1_250050	50S ribosomal protein L20	1621	1595
METTOv1_80111	30S ribosomal protein S7	1618	1626
METTOv1_590017	50S ribosomal protein L11	1611	1524
METTOv1_440016	30S ribosomal protein S8	1602	1412
METTOv1_440005	50S ribosomal protein L2	1599	1392
METTOv1_10051	protein of unknown function	1598	2118
METTOv1_440012	50S ribosomal protein L14	1597	1473
METTOv1_410012	conserved exported protein of unknown function	1591	1734
METTOv1_80113	protein of unknown function	1582	1483
METTOv1_20091	globin	1580	1678
METTOv1_1710001	protein of unknown function	1577	1592
METTOv1_150057	50S ribosomal protein L31	1566	1767
METTOv1_140043	Outer membrane protein	1563	1450
METTOv1_870005	Thioesterase	1542	1473
METTOv1_300010	protein of unknown function	1538	1348
METTOv1_740013	conserved protein of unknown function	1528	1631
METTOv1_780012	nucleotide sugar dehydrogenase	1517	1399
METTOv1_440021	50S ribosomal protein L15	1512	1252
METTOv1_180063	F0F1 ATP synthase subunit beta	1478	1280
METTOv1_440015	30S ribosomal protein S14	1470	1254
METTOv1_60038	ribosomal protein L13	1461	1314
METTOv1_380017	Inorganic diphosphatase	1459	1471
METTOv1_CDS42227	methanobactin precursor	1439	2177
METTOv1_340044	conserved membrane protein of unknown function	1424	1254
METTOv1_200046	Glutamate--ammonia ligase	1413	1375
METTOv1_60075	Alkyl hydroperoxide reductase subunit C (Peroxiredoxin) (Thioredoxin peroxidase) (Alkyl hydroperoxide reductase protein C22) (SC	1411	1460
METTOv1_440019	30S ribosomal protein S5	1411	1175
METTOv1_660011	L-ornithine 5-monooxygenase (L-ornithine N5-oxygenase)	1410	1450
METTOv1_310035	H+transporting two-sector ATPase B/B' subunit	1405	1281
METTOv1_50069	30S ribosomal protein S11	1391	1375
METTOv1_40095	protein of unknown function	1387	1583
METTOv1_180064	F0F1 ATP synthase subunit gamma	1380	1236
METTOv1_200029	exported protein of unknown function	1375	1398

METTOv1_670002	phasin	1371	1276
METTOv1_310031	translation initiation factor IF-3	1368	1564
METTOv1_440003	50S ribosomal protein L4	1362	1174
METTOv1_310034	H+transporting two-sector ATPase B/B' subunit	1358	1362
METTOv1_180066	ATP synthase subunit delta	1355	1304
METTOv1_670019	serine hydroxymethyltransferase	1355	1209
METTOv1_50045	protein of unknown function	1352	1360
METTOv1_400031	transcriptional regulator, MucR family	1347	1461
METTOv1_60105	flagellar basal body rod modification protein	1346	1844
METTOv1_1440001	ATP-dependent metalloprotease FtsH (fragment)	1342	1308
METTOv1_60091	conserved exported protein of unknown function	1316	1398
METTOv1_220011	hemimethylated DNA binding protein	1301	1489
METTOv1_60092	flagellin domain protein	1299	1246
METTOv1_310045	phosphoribosylaminoimidazole-succinocarboxamide synthase	1289	1379
METTOv1_150031	exported protein of unknown function	1272	1162
METTOv1_760008	Sigma-24 (FecI-like) (modular protein)	1260	1522
METTOv1_100063	protein of unknown function	1259	1239
METTOv1_590016	50S ribosomal protein L1	1257	1187
METTOv1_280036	protein of unknown function	1252	1359
METTOv1_160008	putative outer-membrane immunogenic protein precursor	1248	1096
METTOv1_270004	heat shock protein Hsp20	1243	1377
METTOv1_310017	putative Alpha amylase, catalytic subdomain	1242	1235
METTOv1_550007	cytochrome c oxidase, subunit II	1241	1186
METTOv1_970002	30S ribosomal protein S21	1236	1515
METTOv1_10053	protein of unknown function	1226	1255
METTOv1_510003	succinyl-CoA synthetase, alpha subunit	1198	1135
METTOv1_310037	F0F1 ATP synthase subunit A	1197	1135
METTOv1_1110003	Electron transfer flavoprotein alpha/beta-subunit	1192	1075
METTOv1_60037	30S ribosomal protein S9	1189	1026
METTOv1_50070	DNA-directed RNA polymerase subunit alpha	1180	1066
METTOv1_550012	cytochrome c oxidase subunit III	1179	1219
METTOv1_80070	50S ribosomal protein L32	1170	1279
METTOv1_180031	outer membrane protein assembly complex, YaeT protein	1164	1083
METTOv1_550008	cytochrome c oxidase, subunit I	1164	1012
METTOv1_100080	acetoacetyl-CoA reductase	1160	1060
METTOv1_590019	preprotein translocase, SecE subunit	1159	1071
METTOv1_220033	DNA-binding protein HU-beta (NS1) (HU-1)	1155	1046
METTOv1_1340001	ATP-dependent metalloprotease FtsH (fragment)	1139	1260
METTOv1_240004	MxaD protein, putative	1137	1077
METTOv1_80082	50S ribosomal protein L19	1133	1068
METTOv1_1510001	Amino acid adenylation domain protein (fragment)	1121	1172
METTOv1_90054	30S ribosomal protein S2	1105	994
METTOv1_70096	protein of unknown function	1104	891
METTOv1_310038	conserved protein of unknown function	1100	1071
METTOv1_200019	Endopeptidase Clp (modular protein)	1098	1231
METTOv1_50033	PpiC-type peptidyl-prolyl cis-trans isomerase	1090	1137
METTOv1_380039	Integration host factor subunit beta	1080	1053
METTOv1_510021	exported protein of unknown function	1071	1054
METTOv1_10061	protein of unknown function	1067	1274
METTOv1_130056	methionine adenosyltransferase 1 (AdoMet synthetase)	1059	1050
METTOv1_220024	conserved exported protein of unknown function	1052	1076
METTOv1_440002	50S ribosomal protein L3	1045	948
METTOv1_220032	ATP-dependent protease La	1031	1022

METTOv1_480030	iojap-like protein (fragment)	1029	1161
METTOv1_200065	ATP-dependent Clp protease adaptor protein ClpS	1019	1311
METTOv1_180062	ATP synthase epsilon chain	1017	989
METTOv1_760003	Acetylpolyamine aminohydrolase	1014	933
METTOv1_140030	peptidyl-prolyl cis-trans isomerase cyclophilin type	1013	1033
METTOv1_10002	conserved protein of unknown function; putative high-affinity Fe2+/Pb2+ permease	1010	949
METTOv1_140073	Superoxide dismutase	1008	1147
METTOv1_210005	Chaperone protein dnaK	1005	941
METTOv1_80045	exported protein of unknown function	979	995
METTOv1_760006	L-ornithine 5-monooxygenase (L-ornithine N5-oxygenase)	979	1116
METTOv1_140080	conserved exported protein of unknown function	971	903
METTOv1_140029	peptidyl-prolyl cis-trans isomerase cyclophilin type	969	982
METTOv1_60106	flagellar biosynthesis repressor FlbT	962	1141
METTOv1_110052	protein of unknown function	943	1268
METTOv1_870004	conserved protein of unknown function, putative SyrP-like protein	932	1039
METTOv1_600021	4Fe-4S ferredoxin iron-sulfur binding domain protein	931	928
METTOv1_280038	BolA family protein	924	1040
METTOv1_760012	DNA-directed RNA polymerase specialized sigma subunit	922	951
METTOv1_20085	conserved exported protein of unknown function	913	1017
METTOv1_20147	Chemotaxis protein cheY	911	947
METTOv1_370028	conserved protein of unknown function	910	1074
METTOv1_90055	Elongation factor Ts	904	930
METTOv1_410026	flagellar hook-basal body complex protein FliE	879	876
METTOv1_30113	NADH-quinone oxidoreductase subunit B	877	830
METTOv1_380031	protein of unknown function DUF1153	877	850
METTOv1_1020008	ETC complex I subunit conserved region	877	788
METTOv1_140067	rare lipoprotein A	874	796
METTOv1_220012	conserved protein of unknown function; putative signal peptide	870	874
METTOv1_370033	conserved protein of unknown function	867	1184
METTOv1_20145	two component transcriptional regulator, winged helix family	866	991
METTOv1_80036	putative outer membrane receptor; putative iron transport (COG1629)	862	823
METTOv1_210051	30S ribosomal protein S16	861	884
METTOv1_140036	transcriptional regulator, TraR/DksA family	860	914
METTOv1_200023	exported protein of unknown function	859	997
METTOv1_610026	septum site-determining protein MinD	857	839
METTOv1_310039	conserved exported protein of unknown function	851	880
METTOv1_40064	protein of unknown function	828	920
METTOv1_60130	carboxyl-terminal protease	826	915
METTOv1_380035	protein of unknown function	822	878
METTOv1_60104	flagellar biosynthesis protein FliQ	820	972
METTOv1_530029	Porin opacity type	820	754
METTOv1_1020006	cold-shock DNA-binding domain protein	814	1017
METTOv1_10008	3-oxoacyl-(acyl-carrier-protein) synthase 2	812	751
METTOv1_90053	exported protein of unknown function	812	824
METTOv1_250025	protein of unknown function	803	767
METTOv1_770010	protein of unknown function	803	831
METTOv1_60107	flagellar biosynthesis regulatory protein FlaF	796	952
METTOv1_440022	preprotein translocase, SecY subunit	796	794
METTOv1_30115	NADH (or F420H2) dehydrogenase, subunit C	792	869
METTOv1_60109	flagellar hook-associated protein FlgK	784	776

METTOv1_380024	Acyl carrier protein	784	740
METTOv1_180036	Ribosome-recycling factor	769	698
METTOv1_240031	protein of unknown function DUF1321	756	804
METTOv1_360024	putative TonB family protein	751	716
METTOv1_200014	protein of unknown function	750	785
METTOv1_850012	Secretion protein HlyD	737	778
METTOv1_60108	Flagellar hook-associated 3 family protein	736	825
METTOv1_10033	protein of unknown function	734	712
METTOv1_140001	Protein hfq	726	893
METTOv1_310021	Rieske (2Fe-2S) domain protein	726	610
METTOv1_930013	conserved exported protein of unknown function	726	605
METTOv1_760007	Diaminobutyrate--2-oxoglutarate aminotransferase	720	770
METTOv1_610025	Cell division topological specificity factor	717	707
METTOv1_530022	UPF0133 protein Msil_0275	715	666
METTOv1_50016	Protein-export protein secB	712	691
METTOv1_570018	dTDP-glucose 4,6-dehydratase	708	681
METTOv1_130057	transcriptional regulator, XRE family	706	734
METTOv1_20141	ribonuclease P protein component	705	649
METTOv1_760005	protein of unknown function	704	800
METTOv1_600023	transcriptional regulator, CarD family	701	700
METTOv1_310046	exported protein of unknown function	700	724
METTOv1_530024	DSBA oxidoreductase	697	721
METTOv1_460013	exported protein of unknown function	693	594
METTOv1_560002	Methylenetetrahydrofolate dehydrogenase (NADP(+))	688	607
METTOv1_150056	response regulator receiver protein	686	684
METTOv1_270056	putative biopolymer transport protein (fragment)	686	568
METTOv1_360027	conserved exported protein of unknown function	685	647
METTOv1_190005	Ketol-acid reductoisomerase	684	653
METTOv1_200064	ATP-dependent Clp protease, ATP-binding subunit clpA	682	680
METTOv1_150017	exported protein of unknown function	681	611
METTOv1_160051	ferredoxin	681	651
METTOv1_670001	protein of unknown function	680	581
METTOv1_380002	response regulator receiver protein	679	706
METTOv1_1110002	Electron transfer flavoprotein alpha subunit	677	692
METTOv1_10099	DNA-directed RNA polymerase subunit beta'	676	689
METTOv1_280043	conserved exported protein of unknown function	674	705
METTOv1_30112	NADH-ubiquinone/plastoquinone oxidoreductase chain 3	673	725
METTOv1_630017	NADH dehydrogenase (ubiquinone) 24 kDa subunit	672	608
METTOv1_50042	periplasmic binding protein	668	653
METTOv1_310004	cold-shock DNA-binding domain protein	668	822
METTOv1_160009	Outer membrane protein	667	625
METTOv1_220031	ATP-dependent Clp protease ATP-binding subunit clpX	667	663
METTOv1_200018	Trigger factor	663	616
METTOv1_360025	Biopolymer transport protein ExbD/TolR	661	635
METTOv1_360041	succinyl-CoA synthetase, beta subunit	660	631
METTOv1_310025	protein of unknown function	657	726
METTOv1_210007	phospholipid N-methyltransferase	656	643
METTOv1_410028	flagellar basal body rod protein FlgB	652	762
METTOv1_800017	transcriptional regulator, AraC family	652	635
METTOv1_210064	import inner membrane translocase subunit Tim44	650	672
METTOv1_410025	flagellar basal body rod protein FlgG	648	686
METTOv1_310022	nitrate reductase, large subunit	646	597
METTOv1_500002	FecI like protein (fragment)	645	647

METTOv1_70077	putative periplasmic ligand-binding sensor protein	638	588
METTOv1_310048	protein of unknown function DUF1476	636	679
METTOv1_540018	CreA family protein	635	615
METTOv1_10206	thioredoxin	631	740
METTOv1_100064	Lytic transglycosylase catalytic (fragment)	630	675
METTOv1_350006	protein of unknown function DUF336	626	637
METTOv1_400012	conserved protein of unknown function	626	708
METTOv1_440025	protein of unknown function	626	471
METTOv1_300045	protease Do	623	579
METTOv1_820006	Protein grpE	622	636
METTOv1_200013	conserved exported protein of unknown function	621	672
METTOv1_540017	conserved exported protein of unknown function	621	615
METTOv1_110061	tungsten-containing formylmethanofuran dehydrogenase, subunit B	620	570
METTOv1_40025	DNA-directed RNA polymerase subunit omega	619	603
METTOv1_90076	RNA polymerase, sigma 32 subunit, RpoH	612	579
METTOv1_150002	Cytochrome c551 peroxidase	607	514
METTOv1_10133	hopanoid biosynthesis associated radical SAM protein HpnH	606	604
METTOv1_250021	conserved protein of unknown function	603	565
METTOv1_70080	Sec-independent protein translocase protein tatA/E homolog	600	622
METTOv1_1120001	cell division protein FtsZ (fragment)	598	504
METTOv1_100079	acetyl-CoA acetyltransferase	597	561
METTOv1_60097	putative two component transcriptional regulator, winged helix family	595	622
METTOv1_630019	formate dehydrogenase, alpha subunit	593	554
METTOv1_110060	formylmethanofuran dehydrogenase subunit A	591	546
METTOv1_590006	conserved exported protein of unknown function	591	561
METTOv1_240041	S-adenosyl-L-homocysteine hydrolase	590	584
METTOv1_520018	conserved protein of unknown function	590	542
METTOv1_130016	conserved protein of unknown function; putative signal peptide, putative outer membrane protein	588	504
METTOv1_630018	NADH dehydrogenase (quinone)	585	531
METTOv1_520002	protein of unknown function DUF1044	584	780
METTOv1_740014	conserved protein of unknown function	584	703
METTOv1_270057	TonB system transport protein ExbD	583	552
METTOv1_410027	flagellar basal body rod protein FlgC	582	642
METTOv1_40099	GcrA cell cycle regulator	579	560
METTOv1_110068	Crotonyl-CoA reductase	577	523
METTOv1_410009	Partition protein (modular protein)	577	579
METTOv1_360044	putative Protein-export membrane protein, secG-like	565	541
METTOv1_60035	Prevent-host-death family protein	564	539
METTOv1_240010	ATPase associated with various cellular activities AAA_3	563	538
METTOv1_340045	conserved exported protein of unknown function	563	490
METTOv1_30051	exported protein of unknown function	559	566
METTOv1_30116	NADH dehydrogenase I, D subunit	556	530
METTOv1_280004	protein of unknown function	555	484
METTOv1_160052	FAD-dependent pyridine nucleotide-disulphide oxidoreductase	551	535
METTOv1_350003	citrate synthase I	551	531
METTOv1_120046	putative methyl-accepting chemotaxis protein (MCP) (modular protein)	550	482
METTOv1_20064	integration host factor alpha-subunit (IHF-alpha) (modular protein)	549	518
METTOv1_700001	Translation initiation factor IF-2 (fragment)	549	535

METTOv1_10015	cold-shock DNA-binding domain protein	547	509
METTOv1_30114	protein of unknown function	545	624
METTOv1_80057	translation initiation factor IF-1	545	524
METTOv1_140082	putative similar to copper export proteins	545	610
METTOv1_370049	protein of unknown function DUF1150	544	584
METTOv1_30123	NADH-quinone oxidoreductase subunit K	542	481
METTOv1_550031	transposase (fragment)	542	524
METTOv1_360040	Malate dehydrogenase	539	473
METTOv1_10100	DNA-directed RNA polymerase subunit beta	537	543
METTOv1_1130004	membrane-bound proton-translocating pyrophosphatase	537	464
METTOv1_210025	elongation factor P	536	508
METTOv1_110058	formylmethanofuran dehydrogenase subunit C	535	453
METTOv1_840013	formaldehyde-activating enzyme	535	581
METTOv1_400016	Malate thiokinase, large subunit	534	455
METTOv1_590018	NusG antitermination factor	534	451
METTOv1_310040	PmoC	532	492
METTOv1_760009	putative Pyoverdine ABC export system, permease/ATP-binding protein	532	500
METTOv1_80031	protein of unknown function	527	530
METTOv1_360045	exported protein of unknown function	527	487
METTOv1_310018	putative two-component response regulator:antitermination factor NasT	523	505
METTOv1_130071	RNA polymerase sigma factor RpoD	522	542
METTOv1_1620001	Integrase catalytic region	522	560
METTOv1_30117	NADH-quinone oxidoreductase chain E	520	484
METTOv1_1280002	Non-ribosomal peptide synthase:Amino acid adenylation (fragment)	518	614
METTOv1_140083	conserved exported protein of unknown function	517	542
METTOv1_400015	malate thiokinase, small subunit	516	485
METTOv1_80094	transcription elongation factor GreA	515	463
METTOv1_360022	exported protein of unknown function	515	475
METTOv1_10196	exported protein of unknown function	514	532
METTOv1_20049	cobalt chelatase, pCobS small subunit	511	529
METTOv1_100069	outer membrane assembly lipoprotein YfiO	511	487
METTOv1_140031	Phosphopantetheine adenylyltransferase	511	452
METTOv1_540009	conserved protein of unknown function; putative outer membrane protein	511	539
METTOv1_360026	MotA/TolQ/ExbB proton channel	508	424
METTOv1_160015	protein of unknown function	507	480
METTOv1_440023	Adenylate kinase	503	483
METTOv1_60098	conserved exported protein of unknown function	502	615
METTOv1_410007	Porin	500	484
METTOv1_80033	Carbonic anhydrase 2 (Carbonate dehydratase 2)	497	553
METTOv1_110059	formylmethanofuran--tetrahydromethanopterin formyltransferase	496	470
METTOv1_30043	protein of unknown function	491	732
METTOv1_110041	protein of unknown function YGGT	487	464
METTOv1_280052	protein of unknown function	486	431
METTOv1_390001	conserved exported protein of unknown function	486	516
METTOv1_870001	Amino acid adenylation (fragment)	484	481
METTOv1_50014	import inner membrane translocase subunit Tim44	482	509
METTOv1_350033	single-strand binding protein	481	472
METTOv1_650018	ubiquinol-cytochrome c reductase, iron-sulfur subunit	480	391

METTOv1_720018	Translation initiation factor IF-2 (fragment)	480	454
METTOv1_1220001	putative Non-ribosomal peptide synthase:Amino acid adenylation (modular protein) (fragment)	480	564
METTOv1_760002	putative aculeacin a acylase transmembrane protein	479	485
METTOv1_360042	TPR repeat-containing protein	478	457
METTOv1_570019	dTDP-4-dehydrorhamnose 3,5-epimerase	476	470
METTOv1_600008	protein of unknown function	476	477
METTOv1_30118	NADH dehydrogenase I subunit F	475	415
METTOv1_650017	fragment of Cytochrome b/c1 (part 1)	474	429
METTOv1_220035	Propionyl-CoA carboxylase	472	455
METTOv1_190016	conserved exported protein of unknown function	470	449
METTOv1_40021	signal peptidase I	468	498
METTOv1_640006	polyhydroxyalkonate synthesis repressor, PhaR	468	524
METTOv1_160061	conserved protein of unknown function	467	461
METTOv1_40034	exported protein of unknown function	466	435
METTOv1_590030	Cell division protein ftsZ (fragment)	465	407
METTOv1_300011	Protein of unknown function DUF1790	462	452
METTOv1_410033	MotA/TolQ/ExbB proton channel	461	547
METTOv1_490005	Putative chemotaxis protein CheY	461	478
METTOv1_210069	transcriptional regulator, MarR family	458	440
METTOv1_10165	conserved protein of unknown function	456	423
METTOv1_270015	Glycosyl transferase, WecB/TagA/CpsF family (fragment)	455	458
METTOv1_410005	heat shock protein 90	454	446
METTOv1_600011	NADH:ubiquinone oxidoreductase 17.2 kD subunit	453	429
METTOv1_270054	conserved protein of unknown function; putative prolyl 4-hydroxylase, iron-regulated	452	472
METTOv1_650016	fragment of Cytochrome b/c1 (part 2)	451	403
METTOv1_1110001	3-hydroxybutyryl-CoA dehydrogenase	451	448
METTOv1_200034	conserved protein of unknown function	450	469
METTOv1_1480001	fragment of putative transposase (part 2)	450	553
METTOv1_740012	Polyribonucleotide nucleotidyltransferase	449	400
METTOv1_400021	Formate-tetrahydrofolate ligase	448	412
METTOv1_410031	flagellar motor switch protein FliN	448	520
METTOv1_670008	conserved membrane protein of unknown function	447	459
METTOv1_430011	exported protein of unknown function	446	404
METTOv1_190015	Glyoxylate reductase	445	452
METTOv1_360020	exported protein of unknown function	445	444
METTOv1_50030	protein of unknown function	444	415
METTOv1_50047	putative Diguanylate cyclase	444	409
METTOv1_650001	Polysaccharide export protein	442	416
METTOv1_80048	conserved protein of unknown function	439	453
METTOv1_630002	conserved exported protein of unknown function	435	427
METTOv1_870003	Amino acid adenylation domain protein	433	441
METTOv1_20135	Enolase	432	408
METTOv1_150015	conserved protein of unknown function	432	494
METTOv1_300033	glutamate synthase subunit beta	431	401
METTOv1_140021	protein of unknown function	429	472
METTOv1_570004	ErfK/YbiS/YcfS/YnhG family protein	429	397
METTOv1_690020	FecI-like protein	429	399
METTOv1_30129	methylmalonyl-CoA epimerase	428	394
METTOv1_620019	fructose-bisphosphate aldolase, class II, Calvin cycle subtype	427	461
METTOv1_60100	protein of unknown function	426	408
METTOv1_40059	conserved protein of unknown function	423	364

METTOv1_700002	protein of unknown function DUF448	423	388
METTOv1_340005	conserved protein of unknown function	422	370
METTOv1_280033	Redoxin domain protein	421	354
METTOv1_160055	protein of unknown function	420	413
METTOv1_520005	exported protein of unknown function	420	388
METTOv1_840009	protein of unknown function	420	467
METTOv1_150058	protein of unknown function DUF1465	419	489
METTOv1_440028	conserved protein of unknown function	418	366
METTOv1_50108	PTS system fructose subfamily IIA component	416	400
METTOv1_70027	Integrase family protein	416	413
METTOv1_970001	TPR repeat-containing protein	416	402
METTOv1_80043	phosphoserine aminotransferase	415	364
METTOv1_10064	ErfK/YbiS/YcfS/YnhG family protein	413	397
METTOv1_460012	putative Cation efflux system protein cusB precursor	413	368
METTOv1_840007	3-hydroxydecanoyl-(acyl carrier protein) dehydratase	413	347
METTOv1_80037	protein of unknown function	412	376
METTOv1_430027	conserved membrane protein of unknown function	412	381
METTOv1_720017	protein of unknown function	412	500
METTOv1_10079	putative pilus assembly protein cpaE	410	349
METTOv1_130012	protein of unknown function	410	653
METTOv1_10080	Pilus (Caulobacter type) biogenesis lipoprotein CpaD	406	436
METTOv1_130017	3-dehydroquinate dehydratase	402	408
METTOv1_30121	NADH-quinone oxidoreductase subunit I	400	374
METTOv1_160060	acyl-CoA dehydrogenase	400	382
METTOv1_270055	conserved protein of unknown function; putative prolyl 4-hydroxylase, iron-regulated	400	392
METTOv1_10195	short-chain dehydrogenase/reductase SDR	399	378
METTOv1_240003	conserved protein of unknown function	399	391
METTOv1_1090007	protein of unknown function	399	367
METTOv1_50099	alpha/beta hydrolase fold protein	398	394
METTOv1_360047	3-isopropylmalate dehydrogenase	398	366
METTOv1_70087	Peptidase M23	397	384
METTOv1_100077	conserved protein of unknown function	395	489
METTOv1_190006	putative Predicted ester cyclase	395	344
METTOv1_360034	Di-heme cytochrome c peroxidase	395	381
METTOv1_1420001	transposase (fragment)	395	472
METTOv1_930005	conserved exported protein of unknown function	394	423
METTOv1_40010	N(5),N(10)-methenyltetrahydromethanopterin cyclohydrolase	393	312
METTOv1_70063	conserved protein of unknown function	391	379
METTOv1_280047	glyceraldehyde-3-phosphate dehydrogenase, type I	391	407
METTOv1_40051	3-isopropylmalate dehydratase small subunit	389	349
METTOv1_350023	conserved protein of unknown function	389	399
METTOv1_400030	ErfK/YbiS/YcfS/YnhG family protein	389	349
METTOv1_20012	1-deoxy-D-xylulose-5-phosphate synthase	388	382
METTOv1_120045	putative methyl-accepting chemotaxis protein	387	310
METTOv1_80104	exported protein of unknown function	385	402
METTOv1_550010	conserved protein of unknown function	384	466
METTOv1_140011	putative RhsD protein	383	467
METTOv1_60120	response regulator receiver protein	382	365
METTOv1_90065	conserved protein of unknown function; putative Ser protein kinase	382	424
METTOv1_120050	protein of unknown function	382	445
METTOv1_730006	putative TonB-dependent receptor protein	382	383

METTOv1_100062	protein of unknown function	381	335
METTOv1_870002	Polyketide synthase type I	381	399
METTOv1_1220002	protein of unknown function	379	396
METTOv1_400019	D-isomer specific 2-hydroxyacid dehydrogenase NAD-binding	375	348
METTOv1_480011	exported protein of unknown function	375	390
METTOv1_480033	UPF0082 protein Msil_2305	375	308
METTOv1_1310001	arthrofactin synthetase/syringopeptin synthetase C-related non-ribosomal peptide synthetase (fragment)	373	402
METTOv1_160002	pyrroloquinoline quinone biosynthesis protein PqqC	372	344
METTOv1_390004	protein of unknown function	372	391
METTOv1_40108	GTP-binding protein TypA	370	337
METTOv1_410037	protein of unknown function	370	368
METTOv1_310050	protein of unknown function	368	370
METTOv1_370002	putative formate dehydrogenase delta subunit	368	312
METTOv1_190048	Prevent-host-death protein	366	401
METTOv1_1250003	Monooxygenase, FAD-binding	363	407
METTOv1_270058	TonB family protein	361	349
METTOv1_20103	nitrogen fixation protein FixT	360	423
METTOv1_30081	protein of unknown function	360	457
METTOv1_50107	PTS system phosphocarrier protein HPr	360	334
METTOv1_160059	protein of unknown function	360	316
METTOv1_240047	thiamine biosynthesis protein ThiS	359	375
METTOv1_260039	protein of unknown function	359	486
METTOv1_490020	FecI-like protein	359	346
METTOv1_250024	protein of unknown function	358	318
METTOv1_300032	branched-chain amino acid aminotransferase	358	321
METTOv1_50041	transport system permease protein	357	330
METTOv1_10084	Putative type IV prepilin peptidase, cpaA	356	315
METTOv1_410013	Polyprenyl synthetase	356	401
METTOv1_220039	conserved protein of unknown function	355	342
METTOv1_370027	protein of unknown function	355	469
METTOv1_400017	Methenyltetrahydrofolate cyclohydrolase	355	281
METTOv1_610003	conserved protein of unknown function	355	348
METTOv1_200020	Carbamoyl-phosphate synthase L chain ATP-binding	353	310
METTOv1_200056	conserved protein of unknown function	353	462
METTOv1_410017	oxygen-independent coproporphyrinogen III oxidase	353	383
METTOv1_140056	conserved protein of unknown function	352	345
METTOv1_30124	proton-translocating NADH-quinone oxidoreductase, chain L	351	294
METTOv1_200025	putative restriction endonuclease or methylase	351	452
METTOv1_410024	flagella basal body P-ring formation protein FlgA	351	324
METTOv1_420011	Fe-S metabolism associated SufE	351	401
METTOv1_520024	NADH dehydrogenase (ubiquinone)	351	353
METTOv1_550011	cytochrome c oxidase assembly protein CtaG/Cox11	351	346
METTOv1_130013	protein of unknown function	350	461
METTOv1_40106	conserved protein of unknown function	349	383
METTOv1_430028	protein of unknown function	349	306
METTOv1_130022	ErfK/YbiS/YcfS/YnhG family protein	347	329
METTOv1_250022	protein of unknown function	347	440
METTOv1_1030004	protein of unknown function	347	513
METTOv1_310007	conserved protein of unknown function	346	352
METTOv1_30157	conserved protein of unknown function	345	314
METTOv1_80071	conserved protein of unknown function	345	336
METTOv1_420029	protein of unknown function	344	355

METTOv1_570021	glucose-1-phosphate thymidylyltransferase	344	334
METTOv1_850013	putative macrolide ABC transporter, fusion of ATP-binding (N-terminal) and membrane (C-terminal) domains (fragment)	344	317
METTOv1_10027	protein of unknown function	343	358
METTOv1_30057	protein of unknown function	342	327
METTOv1_110043	MaoC like domain protein	341	317
METTOv1_150023	Aspartyl/glutamyl-tRNA(Asn/Gln) amidotransferase subunit C	341	305
METTOv1_380018	GCN5-related N-acetyltransferase	341	354
METTOv1_840006	Beta-ketoacyl synthase	341	301
METTOv1_310008	cold-shock DNA-binding domain protein	340	377
METTOv1_190027	Orn/DAP/Arg decarboxylase 2	339	335
METTOv1_20032	DNA methylase N-4/N-6 domain protein	337	333
METTOv1_390009	exported protein of unknown function	337	327
METTOv1_490023	protein of unknown function	337	325
METTOv1_30125	proton-translocating NADH-quinone oxidoreductase, chain M	336	332
METTOv1_50076	exported protein of unknown function	334	310
METTOv1_70026	protein of unknown function	334	311
METTOv1_310033	protein of unknown function	334	360
METTOv1_740001	mannose-1-phosphate guanylyltransferase/mannose-6-phosphate isomerase	334	333
METTOv1_300013	transcriptional regulator, TetR family	332	328
METTOv1_350037	exported protein of unknown function	332	333
METTOv1_30138	protein of unknown function	330	244
METTOv1_80046	succinate dehydrogenase and fumarate reductase iron-sulfur protein	330	352
METTOv1_150076	conserved protein of unknown function	330	369
METTOv1_370010	regulatory protein, FmdB family	330	334
METTOv1_520014	protein of unknown function	330	483
METTOv1_760011	FecR protein	330	321
METTOv1_310044	5-aminolevulinate synthase	329	334
METTOv1_380038	protein of unknown function	329	266
METTOv1_180032	membrane-associated zinc metalloprotease	328	308
METTOv1_180052	exported protein of unknown function	328	315
METTOv1_570003	histone deacetylase superfamily	328	304
METTOv1_50040	ABC transporter related	327	304
METTOv1_120047	Chemotaxis protein cheW	327	291
METTOv1_140066	Serine-type D-Ala-D-Ala carboxypeptidase	326	304
METTOv1_160053	O-methyltransferase family 3	326	256
METTOv1_70072	protein of unknown function	325	330
METTOv1_640023	conserved protein of unknown function	325	287
METTOv1_160070	protein-export membrane protein SecF	324	336
METTOv1_160071	protein-export membrane protein SecD	324	286
METTOv1_20140	Inner membrane protein oxaA	323	320
METTOv1_10069	protein of unknown function	321	435
METTOv1_70081	Sec-independent protein translocase protein tatB homolog	321	279
METTOv1_80051	succinate dehydrogenase, cytochrome b556 subunit	321	333
METTOv1_240032	conserved exported protein of unknown function	321	268
METTOv1_270014	Glycosyl transferase, WecB/TagA/CpsF family protein	321	320
METTOv1_260038	Metal dependent phosphohydrolase	320	406
METTOv1_20148	CheW protein (fragment)	319	348
METTOv1_70073	conserved protein of unknown function	319	272
METTOv1_90038	exported protein of unknown function	319	343
METTOv1_130019	acetyl-CoA carboxylase, biotin carboxylase	319	305

METTOv1_150032	protein of unknown function DUF1013	319	308
METTOv1_170062	protein of unknown function	319	400
METTOv1_30122	NADH-ubiquinone/plastoquinone oxidoreductase chain 6	318	292
METTOv1_200030	conserved exported protein of unknown function	318	330
METTOv1_670012	Rhodanese domain protein	318	348
METTOv1_350026	protein of unknown function	316	258
METTOv1_20067	glutaredoxin 3	315	279
METTOv1_280048	conserved protein of unknown function	315	321
METTOv1_80049	succinate dehydrogenase flavoprotein subunit	314	302
METTOv1_570027	protein of unknown function	314	308
METTOv1_700003	NusA antitermination factor	314	296
METTOv1_810014	exported protein of unknown function	314	285
METTOv1_1050003	Replication protein b	314	399
METTOv1_1030005	YD repeat protein (modular protein)	313	360
METTOv1_440030	protein-L-isoaspartate(D-aspartate) O-methyltransferase	312	319
METTOv1_520027	protein of unknown function	312	294
METTOv1_300009	ErfK/YbiS/YcfS/YnhG family protein	311	274
METTOv1_380041	protein of unknown function	311	269
METTOv1_510010	Isobutyryl-CoA dehydrogenase	309	295
METTOv1_20150	protein of unknown function	308	282
METTOv1_110042	protein of unknown function DUF167	308	252
METTOv1_410036	conserved protein of unknown function	308	271
METTOv1_40055	putative sigma (54) modulation protein	307	301
METTOv1_50039	conserved protein of unknown function	307	309
METTOv1_90031	putative Arsenate reductase (nitrogenase-associated protein)	307	318
METTOv1_30064	Glutathione S-transferase domain protein	306	259
METTOv1_30120	NADH dehydrogenase subunit H	306	273
METTOv1_40056	PTS IIA-like nitrogen-regulatory protein PtsN	306	309
METTOv1_80083	protein of unknown function	306	240
METTOv1_100074	aspartate-semialdehyde dehydrogenase	306	308
METTOv1_110076	putative Colicin V production protein, cvpA-like (dedE protein) (Pur regulon 18 kDa protein)	306	301
METTOv1_120048	Chemotaxis protein cheA	306	313
METTOv1_140050	FeS assembly SUF system protein	306	347
METTOv1_160003	coenzyme PQQ biosynthesis protein B	306	313
METTOv1_730007	conserved protein of unknown function, putative lyase	306	384
METTOv1_110053	Di-haem cytochrome c peroxidase family	305	293
METTOv1_440037	quinolinate synthetase complex, A subunit	305	308
METTOv1_560023	cytochrome c prime	305	349
METTOv1_170076	ABC transporter related	304	413
METTOv1_220029	Protein translocase subunit secA	304	298
METTOv1_440033	cysteine synthase A	304	298
METTOv1_240002	putative two-component response regulator, with HTH luxR family	303	317
METTOv1_1070003	conserved protein of unknown function	303	498
METTOv1_90057	Transcriptional regulator, TetR family	302	310
METTOv1_410021	Ribonuclease PH	300	281
METTOv1_870008	protein of unknown function	300	254
METTOv1_1190002	Phage-related protein	300	358
METTOv1_100045	conserved protein of unknown function	299	288
METTOv1_140077	aminotransferase class I and II	299	301
METTOv1_10081	putative Pilus assembly protein cpaC; putative signal peptide	298	304
METTOv1_930010	ATP-dependent protease peptidase subunit	298	301

METTOv1_20097	4Fe-4S ferredoxin iron-sulfur binding domain protein	297	390
METTOv1_280042	protein of unknown function	297	306
METTOv1_700010	protein of unknown function	297	274
METTOv1_30087	fragment of conserved protein of unknown function (part 1)	296	286
METTOv1_40096	NAD(P)(+) transhydrogenase (AB-specific)	296	289
METTOv1_80090	putative transcription regulator protein	296	343
METTOv1_180006	membrane protein of unknown function	296	479
METTOv1_240030	conserved protein of unknown function	296	233
METTOv1_40107	Radical SAM domain protein	295	262
METTOv1_200016	protein of unknown function	295	294
METTOv1_230015	conserved protein of unknown function	295	343
METTOv1_120037	protein of unknown function	294	311
METTOv1_140044	putative alpha/beta hydrolase domain protein	294	258
METTOv1_570007	Methyl-accepting chemotaxis sensory transducer with Pas/Pac sensor	294	292
METTOv1_810002	Protein apaG	294	337
METTOv1_20076	protein of unknown function	293	312
METTOv1_30119	NADH dehydrogenase subunit G	293	252
METTOv1_570005	protein of unknown function	293	284
METTOv1_140052	Iron-sulfur cluster assembly protein	292	273
METTOv1_390019	Serine-type D-Ala-D-Ala carboxypeptidase	292	291
METTOv1_1090005	exported protein of unknown function	292	273
METTOv1_10063	exported protein of unknown function	291	306
METTOv1_120044	Chemotaxis protein cheW	291	246
METTOv1_130010	Leucyl aminopeptidase	291	290
METTOv1_20050	heat shock protein DnaJ domain protein	289	283
METTOv1_340046	PepSY-associated TM helix	288	282
METTOv1_160017	protein of unknown function	287	232
METTOv1_200044	protein of unknown function	287	300
METTOv1_520021	iron-sulfur cluster assembly accessory protein	287	274
METTOv1_840015	protein of unknown function	287	317
METTOv1_810015	exported protein of unknown function	286	219
METTOv1_140047	FeS assembly ATPase SufC	285	285
METTOv1_1690001	transposase (fragment)	285	332
METTOv1_80028	conserved protein of unknown function	283	288
METTOv1_700009	recA protein	283	313
METTOv1_720015	Ribosome-binding factor A	283	267
METTOv1_1050004	protein of unknown function	283	338
METTOv1_80034	protein of unknown function	282	281
METTOv1_90034	Phenylalanyl-tRNA synthetase alpha chain	282	242
METTOv1_380001	response regulator receiver modulated diguanylate cyclase	281	272
METTOv1_400018	Bifunctional protein mdtA [Includes: NADP-dependent methylenetetrahydromethanopterin dehydrogenase ; Methylenetetrahydrofolate d	281	243
METTOv1_30044	protein of unknown function	280	492
METTOv1_250028	conserved protein of unknown function	280	273
METTOv1_100052	conserved protein of unknown function; putative glutathione synthetase ATP-binding domain-like	279	292
METTOv1_340049	Membrane protein involved in aromatic hydrocarbon degradation	279	286
METTOv1_40086	2-isopropylmalate synthase	278	282
METTOv1_130018	acetyl-CoA carboxylase, biotin carboxyl carrier protein	278	259
METTOv1_310024	protein serine/threonine phosphatase	278	278

METTOv1_380016	conserved protein of unknown function	278	348
METTOv1_550009	Protoheme IX farnesyltransferase	278	258
METTOv1_250020	protein of unknown function	277	280
METTOv1_370012	2,3,4,5-tetrahydropyridine-2,6-dicarboxylate N-succinyltransferase	277	266
METTOv1_80050	putative Succinate dehydrogenase hydrophobic membrane anchor subunit	276	310
METTOv1_10010	3-oxoacyl-(acyl-carrier-protein) reductase	275	235
METTOv1_10188	GSCFA domain protein	275	266
METTOv1_160026	conserved protein of unknown function	275	324
METTOv1_10024	conserved protein of unknown function	274	259
METTOv1_10082	Putative pilus assembly protein cpaB	274	267
METTOv1_10087	conserved exported protein of unknown function	274	273
METTOv1_550006	Invasion associated locus B family protein (fragment)	274	236
METTOv1_90062	Argininosuccinate synthase	273	262
METTOv1_60048	protein of unknown function	272	274
METTOv1_80052	Transcriptional regulatory protein	272	303
METTOv1_90035	protein of unknown function	272	235
METTOv1_160028	peptidoglycan-associated lipoprotein	272	283
METTOv1_200058	protein of unknown function	272	357
METTOv1_370009	ATPase (fragment)	272	312
METTOv1_1410004	protein of unknown function	272	391
METTOv1_60129	heme exporter protein CcmC	271	268
METTOv1_110065	O-acetylhomoserine/O-acetylserine sulfhydrylase	271	242
METTOv1_550016	exported protein of unknown function	271	273
METTOv1_1570001	Macrolide export ATP-binding/permease protein macB 2 (fragment)	270	294
METTOv1_90029	putative cytochrome C oxidase assembly factor transmembrane protein	269	246
METTOv1_160072	preprotein translocase, YajC subunit (modular protein)	269	246
METTOv1_210002	protein of unknown function	269	193
METTOv1_220058	protein of unknown function DUF1025	269	303
METTOv1_420031	two component, sigma54 specific, transcriptional regulator, Fis family	269	265
METTOv1_10214	major facilitator superfamily MFS_1	268	235
METTOv1_80038	conserved protein of unknown function	268	294
METTOv1_420035	Homospermidine synthase	268	258
METTOv1_690010	aspartate/glutamate/uridylate kinase	268	222
METTOv1_700004	Ribosome maturation factor rimP	268	276
METTOv1_300046	HflC protein	267	246
METTOv1_120038	protein of unknown function	266	350
METTOv1_520009	protein of unknown function	266	318
METTOv1_60096	protein of unknown function	265	275
METTOv1_180035	undecaprenyl diphosphate synthase	265	252
METTOv1_10137	protein of unknown function	264	251
METTOv1_60022	Phosphoribosyl-AMP cyclohydrolase	264	237
METTOv1_80081	Glycoside hydrolase family 25	264	236
METTOv1_190076	putative ABC transporter ATP-binding protein	263	256
METTOv1_1030002	protein of unknown function	263	342
METTOv1_30130	protein of unknown function DUF1467	262	191
METTOv1_160066	response regulator receiver protein	262	237
METTOv1_10088	conserved protein of unknown function	261	271
METTOv1_40029	dihydrodipicolinate synthase	261	233

METTOv1_250041	dTDP-glucose 4-6-dehydratase	261	262
METTOv1_280056	protein of unknown function	261	234
METTOv1_780002	conserved exported protein of unknown function	261	242
METTOv1_10184	Cobalt-zinc-cadmium resistance protein czcB	260	253
METTOv1_40092	ribonuclease, Rne/Rng family	260	237
METTOv1_240054	conserved protein of unknown function	260	297
METTOv1_60021	GTP cyclohydrolase I	259	233
METTOv1_430005	conserved exported protein of unknown function	259	277
METTOv1_690009	aspartate/glutamate/uridylate kinase	259	274
METTOv1_1020005	aminotransferase class-III	259	224
METTOv1_280030	GatB/YqeY	258	272
METTOv1_510006	conserved protein of unknown function	258	283
METTOv1_1560001	conserved protein of unknown function	258	289
METTOv1_180071	adenine phosphoribosyltransferase	257	286
METTOv1_740002	protein of unknown function	257	251
METTOv1_410010	Prokaryotic transcription elongation factor, GreA/GreB, C-terminal domain protein	256	197
METTOv1_120049	response regulator receiver protein	255	254
METTOv1_160058	conserved protein of unknown function	255	224
METTOv1_380032	protein of unknown function	255	227
METTOv1_180038	General secretion pathway protein G	254	285
METTOv1_640007	exported protein of unknown function	254	230
METTOv1_650002	putative CDP-alcohol phosphatidyltransferase	254	268
METTOv1_1380001	protein of unknown function	254	276
METTOv1_10001	heat shock protein HSP20	253	252
METTOv1_10078	type II secretion system protein E	253	238
METTOv1_80055	protein of unknown function	253	239
METTOv1_230044	protein of unknown function	253	271
METTOv1_930004	lytic murein transglycosylase	253	246
METTOv1_100053	protein of unknown function	252	278
METTOv1_60099	protein of unknown function	251	322
METTOv1_90074	conserved membrane protein of unknown function	251	225
METTOv1_370005	UTP-glucose-1-phosphate uridylyltransferase	251	242
METTOv1_30105	methionine aminopeptidase, type I	250	232
METTOv1_320061	conserved protein of unknown function	250	292
METTOv1_380009	tryptophanyl-tRNA synthetase	250	256
METTOv1_880002	putative Subtilisin	250	221
METTOv1_80091	protein of unknown function	249	283
METTOv1_190054	protein of unknown function	249	282
METTOv1_140051	Glyoxalase/bleomycin resistance protein/dioxygenase	248	246
METTOv1_180059	Ferredoxin	248	286
METTOv1_40083	ErfK/YbiS/YcfS/YnhG family protein	247	215
METTOv1_110051	putative 31 kDa outer-membrane immunogenic protein	247	236
METTOv1_150050	protein of unknown function	247	316
METTOv1_470041	transposase	247	212
METTOv1_600004	OmpA/MotB domain protein	247	271
METTOv1_950008	transposase	247	198
METTOv1_970007	conserved protein of unknown function	247	233
METTOv1_10041	Flp/Fap pilin component	246	331
METTOv1_20093	LRV FeS4 cluster domain protein (fragment)	246	219
METTOv1_60134	DNA polymerase III subunit beta	246	246
METTOv1_60135	Prevent-host-death family protein	246	225
METTOv1_80047	putative PilT protein domain protein	246	289

METTOv1_570020	dTDP-4-dehydrorhamnose reductase	246	231
METTOv1_80109	protein of unknown function	245	284
METTOv1_140062	protein of unknown function	245	356
METTOv1_150014	pyruvate kinase	245	224
METTOv1_360043	CTP synthase	245	232
METTOv1_980011	transcriptional regulator, XRE family	245	250
METTOv1_10066	PilT protein domain protein	244	243
METTOv1_190013	putative ferric uptake regulation protein, FUR family	244	227
METTOv1_240022	putative cytochrome c protein	244	192
METTOv1_260057	transposase (fragment)	244	242
METTOv1_60101	protein of unknown function	243	287
METTOv1_220030	6-phosphofructokinase	243	239
METTOv1_250049	conserved protein of unknown function	243	351
METTOv1_340016	conserved exported protein of unknown function	243	220
METTOv1_350024	conserved exported protein of unknown function	243	283
METTOv1_80027	putative transcriptional regulator	242	296
METTOv1_300047	HflK protein	242	218
METTOv1_440046	transcriptional coactivator/pterin dehydratase	242	278
METTOv1_1050005	putative replication protein A	242	295
METTOv1_20013	exported protein of unknown function	241	239
METTOv1_30126	proton-translocating NADH-quinone oxidoreductase, chain N	241	210
METTOv1_140046	FeS assembly protein SufB	241	255
METTOv1_220059	threonyl-tRNA synthetase	241	245
METTOv1_180051	protein of unknown function	240	230
METTOv1_350040	conserved membrane protein of unknown function	240	185
METTOv1_30152	protein of unknown function	239	240
METTOv1_80015	putative Cytochrome c class I	239	183
METTOv1_1020009	protein of unknown function	239	404
METTOv1_10187	conserved protein of unknown function	238	228
METTOv1_80032	Sulfate transporter family protein	238	245
METTOv1_540002	protein of unknown function	238	234
METTOv1_110077	protein of unknown function	237	285
METTOv1_630008	polyhydroxyalkanoate depolymerase, intracellular	237	263
METTOv1_240063	conserved protein of unknown function	236	302
METTOv1_380040	signal peptide peptidase SppA, 36K type	236	226
METTOv1_450015	conserved protein of unknown function	236	298
METTOv1_510002	2-oxoglutarate dehydrogenase E1 component	236	237
METTOv1_520001	transcriptional regulator, XRE family	236	296
METTOv1_50006	putative MaoC-like dehydratase	235	275
METTOv1_210047	Predicted transcriptional regulators containing the CopG/Arc/MetJ DNA-binding domain	235	204
METTOv1_220017	Orotate phosphoribosyltransferase	235	280
METTOv1_270063	3-hydroxybutyrate dehydrogenase	235	232
METTOv1_280025	protein of unknown function	235	317
METTOv1_1120002	cell division protein FtsA	235	209
METTOv1_70008	protein of unknown function	234	344
METTOv1_220004	Response regulator (modular protein)	234	248
METTOv1_310030	conserved exported protein of unknown function	234	205
METTOv1_430007	putative Peptidase C14, caspase catalytic subunit p20	234	232
METTOv1_740015	methionine gamma-lyase	234	239
METTOv1_370038	Protein required for attachment to host cells-like	233	258
METTOv1_650003	putative Diguanylate cyclase	233	209
METTOv1_70069	conserved exported protein of unknown function	232	246

METTOv1_420039	Twin-arginine translocation (Tat)	232	255
METTOv1_60103	flagellar biosynthesis protein FlhA	231	251
METTOv1_180060	conserved protein of unknown function	231	242
METTOv1_310047	protein of unknown function	231	275
METTOv1_410023	flagellar basal body P-ring protein	230	214
METTOv1_1090006	5-formyltetrahydrofolate cyclo-ligase	230	208
METTOv1_80035	putative transcriptional repressor of nickel transport, nickel-responsive (nikR)	229	195
METTOv1_120043	Chemotaxis protein methyltransferase	229	224
METTOv1_280034	Hydrolase protein	229	192
METTOv1_400011	glycerate kinase	229	211
METTOv1_640005	putative diguanylate cyclase (GGDEF)/phosphodiesterase (EAL)	229	224
METTOv1_840005	enoyl-(acyl carrier protein) reductase	229	218
METTOv1_40028	SsrA-binding protein	228	231
METTOv1_630001	membrane protein of unknown function	228	224
METTOv1_10159	protein of unknown function	227	247
METTOv1_30082	protein of unknown function	227	240
METTOv1_90048	protein of unknown function	227	231
METTOv1_670015	conserved protein of unknown function	227	240
METTOv1_1190001	Predicted transcriptional regulator	227	250
METTOv1_10140	ABC transporter related	226	216
METTOv1_440027	protein of unknown function	226	192
METTOv1_530012	protein TolQ	226	193
METTOv1_200059	conserved exported protein of unknown function	225	247
METTOv1_250026	protein of unknown function	225	221
METTOv1_820009	protease Do	225	169
METTOv1_960006	Rv0623 family protein transcription factor	225	216
METTOv1_10136	protein of unknown function	224	184
METTOv1_130003	protein of unknown function	224	293
METTOv1_190012	phosphate ABC transporter, periplasmic phosphate-binding protein	224	221
METTOv1_310053	protein of unknown function	224	339
METTOv1_470005	protein of unknown function	224	292
METTOv1_570006	conserved exported protein of unknown function	224	194
METTOv1_830002	Acetyl-coenzyme A carboxylase carboxyl transferase subunit beta	224	215
METTOv1_1130002	NusB antitermination factor	224	219
METTOv1_10065	Prevent-host-death family protein	223	215
METTOv1_50010	protein of unknown function	223	222
METTOv1_220057	3-isopropylmalate dehydratase large subunit	223	197
METTOv1_230036	protein of unknown function	223	246
METTOv1_340018	DNA gyrase, A subunit	223	223
METTOv1_360036	YCII-related	223	215
METTOv1_600010	protein of unknown function	223	204
METTOv1_20098	HesB/YadR/YfhF-family protein	222	266
METTOv1_280061	protein of unknown function	222	233
METTOv1_420020	conserved protein of unknown function	222	243
METTOv1_640001	phospholipase/Carboxylesterase	222	213
METTOv1_1430001	transposase	222	219
METTOv1_180029	beta-hydroxyacyl-(acyl-carrier-protein) dehydratase FabZ	221	181
METTOv1_10067	arginyl-tRNA-protein transferase	220	200
METTOv1_180037	conserved exported protein of unknown function	220	218
METTOv1_300048	exported protein of unknown function	220	193

METTOv1_510009	conserved protein of unknown function	220	178
METTOv1_1260001	ATP-dependent metalloprotease FtsH (fragment)	220	203
METTOv1_20155	exported protein of unknown function	219	195
METTOv1_390018	heat shock protein DnaJ domain protein	219	240
METTOv1_410034	flagellar basal body rod protein	219	212
METTOv1_110082	Phosphate acyltransferase	218	190
METTOv1_200043	protein of unknown function	218	209
METTOv1_670009	protein of unknown function	218	320
METTOv1_1230005	conserved exported protein of unknown function	218	222
METTOv1_10134	hopanoid-associated phosphorylase	217	218
METTOv1_20041	LexA repressor	217	203
METTOv1_40081	peptide methionine sulfoxide reductase	217	185
METTOv1_200060	protein of unknown function	217	245
METTOv1_440038	conserved protein of unknown function	217	243
METTOv1_460014	protein of unknown function	217	266
METTOv1_40058	two-component response regulator	216	225
METTOv1_200067	DEAD/DEAH box helicase domain protein	216	215
METTOv1_360003	Peptide deformylase	216	227
METTOv1_470004	protein of unknown function	216	227
METTOv1_20055	conserved protein of unknown function	215	190
METTOv1_210006	chaperone protein DnaJ	215	216
METTOv1_310002	protein of unknown function	215	171
METTOv1_410030	flagellar motor switch protein FliG	215	207
METTOv1_90050	conserved protein of unknown function	214	250
METTOv1_550024	Putative metalloprotease Msil_2895	214	215
METTOv1_10171	Thioredoxin	213	197
METTOv1_40132	chaperone DnaJ domain protein	213	227
METTOv1_310016	Sulfate transporter family protein	213	196
METTOv1_410001	ATP-dependent chaperone ClpB	213	182
METTOv1_700013	UspA	213	256
METTOv1_40027	Uracil-DNA glycosylase superfamily (fragment)	212	189
METTOv1_50022	transcription termination factor Rho	212	203
METTOv1_80029	conserved protein of unknown function	212	219
METTOv1_90036	protein of unknown function	212	202
METTOv1_280008	putative nitrogen regulatory protein (NtrP-like); putative virulence associated protein (vap)	212	250
METTOv1_420018	conserved protein of unknown function	212	281
METTOv1_1120003	cell division protein FtsQ	212	184
METTOv1_10060	Beta-ketoacyl synthase	211	211
METTOv1_20042	protein of unknown function	211	145
METTOv1_60088	NUDIX hydrolase	211	203
METTOv1_60115	protein of unknown function	211	188
METTOv1_110075	amidophosphoribosyltransferase	211	195
METTOv1_180003	Histidinol dehydrogenase	211	204
METTOv1_210066	PpiC-type peptidyl-prolyl cis-trans isomerase	211	200
METTOv1_740005	outer membrane lipoprotein carrier protein LolA (modular protein)	211	236
METTOv1_20104	protein of unknown function	210	257
METTOv1_90091	protein of unknown function	210	180
METTOv1_280026	protein of unknown function	210	330
METTOv1_350053	conserved protein of unknown function	210	207
METTOv1_370001	formate dehydrogenase family accessory protein FdhD	210	199
METTOv1_410003	protein of unknown function	210	163

METTOv1_20051	BolA family protein	209	199
METTOv1_150022	Glutamyl-tRNA(Gln) amidotransferase subunit A	209	182
METTOv1_350017	Polysaccharide export protein	209	199
METTOv1_70091	Septum formation initiator	208	230
METTOv1_180057	Sulfite/ferredoxin-nitrite reductase	208	166
METTOv1_400003	cobalamin synthesis protein P47K	208	198
METTOv1_10048	UPF0301 protein Msil_1255	207	192
METTOv1_40060	RNA polymerase, sigma-24 subunit, ECF subfamily	207	211
METTOv1_90071	Flavoprotein	207	209
METTOv1_380023	exported protein of unknown function	207	224
METTOv1_390033	conserved protein of unknown function	207	195
METTOv1_430023	Methyl-accepting chemotaxis protein McpB	207	166
METTOv1_760014	Integrase, catalytic region (fragment)	207	254
METTOv1_930009	ATP-dependent hsl protease ATP-binding subunit hslU	207	190
METTOv1_10007	aminodeoxychorismate lyase	206	208
METTOv1_10050	transposase	206	201
METTOv1_10121	putative RND efflux membrane fusion protein	206	174
METTOv1_60123	protein of unknown function	206	179
METTOv1_100004	putative Zn-ribbon-containing protein involved in phosphonate metabolism, PhnA-like protein.	206	243
METTOv1_410020	Ham1 family protein	206	202
METTOv1_500001	FecR protein (fragment)	206	196
METTOv1_1110006	conserved protein of unknown function	206	181
METTOv1_140049	cysteine desulfurase	205	202
METTOv1_280023	protein of unknown function	205	242
METTOv1_370047	protease Do	205	181
METTOv1_40053	protein of unknown function	204	231
METTOv1_140075	conserved protein of unknown function	204	202
METTOv1_250036	ribose-phosphate pyrophosphokinase	204	210
METTOv1_540003	putative prophage regulatory protein (modular protein)	204	210
METTOv1_90089	putative acyl-CoA thiolase	203	176
METTOv1_110084	SmpA/OmlA domain protein	203	225
METTOv1_880003	putative Subtilisin	203	193
METTOv1_80040	Sel1 domain protein repeat-containing protein	202	203
METTOv1_240009	protein of unknown function	202	167
METTOv1_50067	methylmalonyl-CoA mutase, large subunit	201	188
METTOv1_280022	3'-5' exonuclease	201	205
METTOv1_20107	protein of unknown function	200	222
METTOv1_130008	membrane protein of unknown function	200	188
METTOv1_170084	protein of unknown function	200	227
METTOv1_190034	Tail Collar domain protein	200	181
METTOv1_240029	conserved protein of unknown function	200	193
METTOv1_390023	conserved exported protein of unknown function	200	228
METTOv1_20186	protein of unknown function	199	182
METTOv1_50111	two component transcriptional regulator, winged helix family	199	234
METTOv1_280049	Phosphoglycerate kinase	199	208
METTOv1_300058	Glutamate synthase (ferredoxin)	199	183
METTOv1_1410003	protein of unknown function	199	319
METTOv1_370007	phosphoglucomutase/phosphomannomutase alpha/beta/alpha domain II	198	147
METTOv1_510007	putative ErfK/YbiS/YcfS/YnhG protein family	198	164
METTOv1_620013	ubiquinone/menaquinone biosynthesis methyltransferase	198	189
METTOv1_680003	chromosomal replication initiation protein	198	177

METTOv1_1040004	protein of unknown function	198	240
METTOv1_10076	putative pilus assembly protein; putative membrane protein	197	241
METTOv1_70076	protein of unknown function	197	186
METTOv1_80024	conserved protein of unknown function	197	186
METTOv1_340010	protein of unknown function	197	356
METTOv1_420033	glutathione peroxidase	197	180
METTOv1_1030001	conserved protein of unknown function	197	279
METTOv1_40061	fumarate hydratase, class II	196	185
METTOv1_390031	nitrogen metabolism transcriptional regulator, NtrC, Fis Family	196	177
METTOv1_410006	conserved protein of unknown function	196	151
METTOv1_410016	exported protein of unknown function	196	172
METTOv1_740006	Alcohol dehydrogenase zinc-binding domain protein	196	198
METTOv1_110088	CDP-diacylglycerol/glycerol-3-phosphate 3-phosphatidyltransferase	195	195
METTOv1_160027	conserved protein of unknown function	195	261
METTOv1_160073	conserved exported protein of unknown function	195	210
METTOv1_280060	fatty acid desaturase	195	195
METTOv1_280062	8-amino-7-oxononanoate synthase	195	202
METTOv1_280068	penicillin-binding protein, 1A family	195	180
METTOv1_300037	conserved protein of unknown function	195	217
METTOv1_10039	exported protein of unknown function	194	224
METTOv1_60124	conserved exported protein of unknown function	194	185
METTOv1_60131	Peptidase M23	194	193
METTOv1_90067	putative SpoVR like protein (sporulation protein)	194	191
METTOv1_200009	protein of unknown function	194	217
METTOv1_300021	exported protein of unknown function	194	218
METTOv1_380008	exported protein of unknown function	194	173
METTOv1_480013	exported protein of unknown function	194	184
METTOv1_690014	ErfK/YbiS/YcfS/YnhG family protein	194	211
METTOv1_820015	Polysaccharide export protein	194	183
METTOv1_10030	conserved protein of unknown function	193	211
METTOv1_130015	Organic solvent tolerance protein	193	182
METTOv1_200066	exported protein of unknown function	193	170
METTOv1_300057	histidine triad (HIT) protein	193	160
METTOv1_830019	putative membrane protein	193	223
METTOv1_1020001	putative Excinuclease ABC, C subunit, N-terminal	193	252
METTOv1_60006	glutamine amidotransferase of anthranilate synthase	192	150
METTOv1_60029	conserved protein of unknown function	192	177
METTOv1_240055	protein of unknown function	192	195
METTOv1_10147	conserved protein of unknown function	191	211
METTOv1_40101	ornithine carbamoyltransferase	191	208
METTOv1_60005	protein of unknown function	191	176
METTOv1_80008	conserved protein of unknown function	191	271
METTOv1_190052	conserved protein of unknown function	191	175
METTOv1_240005	von Willebrand factor type A	191	201
METTOv1_270059	Glyoxalase/bleomycin resistance protein/dioxygenase	191	225
METTOv1_370050	2-oxoglutarate dehydrogenase, E2 subunit, dihydrolipoamide succinyltransferase (fragment)	191	181
METTOv1_380021	acetyl-CoA carboxylase carboxyltransferase subunit alpha	191	192
METTOv1_590007	protein of unknown function	191	171
METTOv1_30028	conserved protein of unknown function	190	190
METTOv1_90066	conserved protein of unknown function (DUF444)	190	187
METTOv1_510001	protein of unknown function	190	159

METTOv1_510008	conserved protein of unknown function	190	175
METTOv1_510016	conserved protein of unknown function; Transglutaminase-like domain, putative cysteine protease.	190	177
METTOv1_70082	Sec-independent protein translocase, TatC subunit	189	187
METTOv1_70089	alkyl hydroperoxide reductase/ Thiol specific antioxidant/ Mal allergen	189	224
METTOv1_280021	protein of unknown function	189	194
METTOv1_460015	oxidodeductase	189	197
METTOv1_10031	protein of unknown function	188	172
METTOv1_70074	aspartate kinase	188	154
METTOv1_100073	conserved protein of unknown function	188	191
METTOv1_120029	protein of unknown function	188	180
METTOv1_350054	protein of unknown function	188	213
METTOv1_370011	conserved membrane protein of unknown function	188	201
METTOv1_660017	protein of unknown function	188	184
METTOv1_1090001	Sporulation domain protein (fragment)	188	161
METTOv1_10058	MaoC domain protein dehydratase	187	127
METTOv1_60013	Protein meaA	187	162
METTOv1_180055	Phosphoadenosine phosphosulfate reductase (PAPS reductase, thioredoxin dependent) (PAdoPS reductase) (3'-phosphoadenylylsulfate	187	172
METTOv1_180058	conserved protein of unknown function	187	171
METTOv1_10035	exported protein of unknown function	186	193
METTOv1_10135	squalene-hopene cyclase	186	173
METTOv1_10207	exported protein of unknown function	186	194
METTOv1_170083	putative restriction endonuclease protein	186	223
METTOv1_210050	signal recognition particle protein	186	174
METTOv1_620027	transposase (fragment)	186	161
METTOv1_700011	alanyl-tRNA synthetase	186	183
METTOv1_40008	protein of unknown function	185	264
METTOv1_140015	putative Response regulator receiver protein	185	180
METTOv1_830007	conserved protein of unknown function	185	188
METTOv1_70006	protein of unknown function	184	199
METTOv1_110057	protein of unknown function	184	174
METTOv1_540001	protein of unknown function	184	185
METTOv1_720014	tRNA pseudouridine synthase B	184	166
METTOv1_1040005	Prevent-host-death family protein	184	230
METTOv1_10005	guanylate kinase	183	142
METTOv1_10077	putative pilus assembly protein; putative membrane protein	183	180
METTOv1_20046	peptidase M16 domain protein	183	185
METTOv1_180054	sulfate adenylyltransferase subunit 2	183	184
METTOv1_340037	protein of unknown function	183	151
METTOv1_810008	O-succinylhomoserine sulfhydrylase	183	163
METTOv1_80056	maf protein	182	181
METTOv1_120042	chemotaxis-specific methylesterase	182	176
METTOv1_180040	General secretion protein D	182	176
METTOv1_1190003	protein of unknown function	182	185
METTOv1_70092	protein of unknown function	181	209
METTOv1_190071	exported protein of unknown function	181	130
METTOv1_200033	putative nitroreductase family protein; putative Oxygen-insensitive NADPH nitroreductase (NfsA-like)	181	175
METTOv1_240043	protein of unknown function	181	244

METTOv1_340039	pyruvate dehydrogenase (acetyl-transferring) E1 component, alpha subunit	181	175
METTOv1_350012	DEAD/DEAH box helicase domain protein	181	159
METTOv1_830003	tryptophan synthase subunit alpha	181	161
METTOv1_20111	response regulator receiver protein	180	239
METTOv1_80073	ABC-2 type transporter	180	204
METTOv1_250019	conserved exported protein of unknown function	180	195
METTOv1_300044	Heat shock protein 70-binding protein (fragment)	180	137
METTOv1_350014	protein of unknown function	180	210
METTOv1_420030	oligoendopeptidase, pepF/M3 family	180	192
METTOv1_140037	isocitrate dehydrogenase	179	178
METTOv1_150020	Aspartyl/glutamyl-tRNA(Asn/Gln) amidotransferase subunit B	179	143
METTOv1_440026	Phosphoesterase PA-phosphatase related	179	174
METTOv1_40065	protein of unknown function	178	131
METTOv1_40100	acetylornithine and succinylornithine aminotransferase	178	178
METTOv1_80099	protein of unknown function	178	228
METTOv1_270065	protein of unknown function	178	202
METTOv1_350052	protein of unknown function	178	189
METTOv1_10068	RDD domain containing protein	177	139
METTOv1_330002	putative replication protein B	177	257
METTOv1_360037	protein of unknown function DUF55	177	156
METTOv1_80093	carbamoyl-phosphate synthase, large subunit	176	172
METTOv1_250002	conserved protein of unknown function	176	199
METTOv1_310032	Regulatory protein, FmdB family	176	209
METTOv1_350027	protein of unknown function	176	145
METTOv1_480029	Ribosomal RNA large subunit methyltransferase H	176	179
METTOv1_560022	alkyl hydroperoxide reductase/ Thiol specific antioxidant/ Mal allergen	176	169
METTOv1_640020	phosphoribosylaminoimidazole carboxylase, catalytic subunit	176	155
METTOv1_820010	two component transcriptional regulator, winged helix family	176	151
METTOv1_10029	protein of unknown function	174	192
METTOv1_10055	glycosyl transferase family 2	174	157
METTOv1_20138	acetylglutamate kinase	174	163
METTOv1_190053	protein of unknown function	174	158
METTOv1_220048	conserved protein of unknown function	174	178
METTOv1_220068	phosphate ABC transporter, periplasmic phosphate-binding protein	174	187
METTOv1_50117	4-hydroxy-3-methylbut-2-en-1-yl diphosphate synthase	173	161
METTOv1_140076	protein of unknown function	173	157
METTOv1_140079	protein of unknown function	173	136
METTOv1_170081	phosphoribosyl-ATP diphosphatase	173	148
METTOv1_210008	orotidine 5'-phosphate decarboxylase	173	154
METTOv1_570026	arginyl-tRNA synthetase	173	163
METTOv1_620017	Phosphoribulokinase	173	164
METTOv1_880008	putative RNA polymerase, sigma-24 subunit, ECF subfamily	173	163
METTOv1_140074	LuxR family transcriptional regulator	172	214
METTOv1_230014	protein of unknown function	172	195
METTOv1_480032	Alcohol dehydrogenase zinc-binding domain protein	172	150
METTOv1_530013	Biopolymer transport protein ExbD/TolR	172	136
METTOv1_560001	H4MPT-linked C1 transfer pathway protein	172	167
METTOv1_570011	protein of unknown function	172	156
METTOv1_1120004	D-alanine--D-alanine ligase	172	173
METTOv1_10006	YicC domain protein	171	165

METTOv1_10122	acriflavin resistance protein	171	179
METTOv1_10193	putative RNA polymerase, sigma-24 subunit, ECF subfamily	171	191
METTOv1_60116	response regulator receiver protein	171	164
METTOv1_80023	conserved protein of unknown function	171	153
METTOv1_110066	Alkylhydroperoxidase like protein, AhpD family	171	144
METTOv1_190003	Transcriptional regulator/antitoxin, MazE (fragment)	171	176
METTOv1_220069	protein of unknown function	171	187
METTOv1_250027	Adenylosuccinate synthetase	171	169
METTOv1_1330001	protein of unknown function	171	184
METTOv1_30086	fragment of conserved protein of unknown function (part 2)	170	189
METTOv1_90026	HNH endonuclease	170	119
METTOv1_180080	transcription activator effector binding	170	156
METTOv1_300035	ErfK/YbiS/YcfS/YnhG family protein	170	204
METTOv1_370048	protein of unknown function	170	227
METTOv1_730008	conserved protein of unknown function	170	175
METTOv1_50011	HemY domain protein	169	174
METTOv1_130009	Biotin synthase	169	153
METTOv1_370040	protein of unknown function	169	174
METTOv1_530023	Recombination protein recR	169	146
METTOv1_830020	protein of unknown function	169	133
METTOv1_10089	protein of unknown function	168	151
METTOv1_40054	RNA polymerase, sigma 54 subunit, RpoN	168	164
METTOv1_190002	acetolactate synthase, small subunit	168	160
METTOv1_210001	protein of unknown function	168	126
METTOv1_270006	protein of unknown function	168	151
METTOv1_340051	conserved exported protein of unknown function	168	219
METTOv1_400029	protein of unknown function DUF971	168	160
METTOv1_510018	protein of unknown function DUF404	168	155
METTOv1_60011	conserved protein of unknown function	167	147
METTOv1_60086	protein of unknown function	167	163
METTOv1_210052	16S rRNA-processing protein RimM	167	177
METTOv1_30135	transcriptional regulator, XRE family (fragment)	166	169
METTOv1_80069	protein of unknown function	166	153
METTOv1_80098	conserved protein of unknown function	166	198
METTOv1_160001	Coenzyme PQQ synthesis protein E (modular protein)	166	161
METTOv1_200002	Activator of Hsp90 ATPase 1 family protein	166	189
METTOv1_450006	conserved protein of unknown function	166	156
METTOv1_140004	MazG family protein	165	153
METTOv1_140020	Glutathione S-transferase domain protein	165	160
METTOv1_280027	putative transcriptional regulator, CopG family	165	135
METTOv1_300026	conserved protein of unknown function	165	172
METTOv1_760001	exported protein of unknown function	165	197
METTOv1_830013	exported protein of unknown function	165	118
METTOv1_1340003	tol-pal system protein YbgF (fragment)	165	149
METTOv1_40020	Ribonuclease 3	164	175
METTOv1_50027	parB-like partition protein	164	160
METTOv1_240021	PQQ-dependent dehydrogenase, methanol/ethanol family	164	109
METTOv1_830018	exported protein of unknown function	164	135
METTOv1_10056	Ceramide glucosyltransferase	163	156
METTOv1_130054	thioredoxin	163	161
METTOv1_280059	fatty acid hydroxylase	163	157
METTOv1_670018	conserved protein of unknown function	163	147
METTOv1_780008	squalene synthase HpnC	163	142

METTOv1_10014	conserved protein of unknown function	162	158
METTOv1_40085	aconitate hydratase	162	145
METTOv1_60087	Aspartyl-tRNA synthetase	162	152
METTOv1_70095	TonB-dependent receptor	162	152
METTOv1_140035	conserved exported protein of unknown function	162	152
METTOv1_180044	exported protein of unknown function	162	124
METTOv1_200011	lipoprotein releasing system, transmembrane protein, LolC/E family	162	181
METTOv1_300012	pyrroline-5-carboxylate reductase	162	152
METTOv1_380033	GTP-binding protein lepA	162	149
METTOv1_390032	Plasmid stabilization system	162	162
METTOv1_450008	protein of unknown function	162	123
METTOv1_510014	Glyoxalase family protein	162	183
METTOv1_10141	ABC-2 type transporter	161	148
METTOv1_10219	GDP-mannose 4,6-dehydratase	161	153
METTOv1_110033	two component transcriptional regulator, winged helix family	161	153
METTOv1_230046	conserved protein of unknown function	161	167
METTOv1_480012	exported protein of unknown function	161	143
METTOv1_730019	putative type I restriction enzyme specificity protein	161	188
METTOv1_60128	putative heme exporter D (Cytochrome C-type biogenesis protein) transmembrane	160	177
METTOv1_70066	Predicted transcriptional regulator containing the CopG/Arc/MetJ DNA-binding domain	160	130
METTOv1_250010	protein of unknown function DUF465	160	155
METTOv1_340041	pyruvate dehydrogenase subunit beta	160	158
METTOv1_410032	putative Flagellar motor switch protein FliM	160	187
METTOv1_520025	protein of unknown function DUF540	160	137
METTOv1_720009	protein of unknown function	160	146
METTOv1_1630001	IstB domain protein ATP-binding protein	160	157
METTOv1_60136	conserved protein of unknown function	159	161
METTOv1_210061	protein of unknown function	159	154
METTOv1_230045	putative Response regulator receiver protein	159	182
METTOv1_440034	membrane protein of unknown function	159	183
METTOv1_520008	Succinyl-diaminopimelate desuccinylase	159	154
METTOv1_860001	Sel1 domain protein repeat-containing protein (fragment)	159	146
METTOv1_210022	exported protein of unknown function	158	168
METTOv1_380037	Divalent-cation tolerance protein cutA	158	152
METTOv1_460004	Phage integrase	158	171
METTOv1_640015	glycyl-tRNA synthetase subunit alpha	158	136
METTOv1_720005	Phospho-N-acetylmuramoyl-pentapeptide-transferas e	158	146
METTOv1_20047	peptidase M16 domain protein	157	142
METTOv1_300028	conserved protein of unknown function	157	263
METTOv1_340054	conserved protein of unknown function	157	231
METTOv1_380045	membrane protein of uknown function UCP014873	157	184
METTOv1_400028	molybdenum cofactor biosynthesis protein A	157	150
METTOv1_620021	protein of unknown function	157	163
METTOv1_770011	RNA binding S1 domain protein	157	160
METTOv1_1230001	putative metal dependent phosphohydrolase	157	124
METTOv1_30084	conserved protein of unknown function	156	174
METTOv1_90088	putative bifunctional anaerobic fatty acid oxidation complex protein (fadJ/fadB-like): enoyl-CoA hydratase/epimerase/isomerase ;	156	158
METTOv1_110087	molybdopterin converting factor, subunit 1	156	137

METTOv1_140008	conserved exported protein of unknown function	156	181
METTOv1_190050	conserved protein of unknown function	156	171
METTOv1_600009	leucyl/phenylalanyl-tRNA/protein transferase	156	142
METTOv1_670010	protein of unknown function	156	139
METTOv1_1130001	6,7-dimethyl-8-ribityllumazine synthase	156	146
METTOv1_10004	conserved exported protein of unknown function	155	117
METTOv1_10057	short-chain dehydrogenase/reductase SDR	155	153
METTOv1_30063	polyhydroxyalkanoate depolymerase, intracellular	155	151
METTOv1_180053	Sulfate adenylyltransferase subunit 1 (Sulfate adenylate transferase) (SAT) (ATP-sulfurylase large subunit)	155	124
METTOv1_190055	ATP/cobalamin adenosyltransferase	155	163
METTOv1_340025	replicative DNA helicase	155	137
METTOv1_370006	UDP-glucose 4-epimerase	155	153
METTOv1_540008	putative HlyD family secretion protein	155	140
METTOv1_60008	Indole-3-glycerol phosphate synthase	154	127
METTOv1_220016	cytochrome c class I	154	193
METTOv1_510017	protein of unknown function DUF403	154	159
METTOv1_1020004	exported protein of unknown function	154	129
METTOv1_10075	conserved protein of unknown function; TPR domain protein	153	115
METTOv1_10129	protein of unknown function DUF29	153	173
METTOv1_50017	DNA polymerase III, epsilon subunit	153	163
METTOv1_430012	protein of unknown function	153	106
METTOv1_600006	flagellar MS-ring protein	153	142
METTOv1_750001	transposase	153	195
METTOv1_190051	GTP-binding protein EngA	152	131
METTOv1_260042	protein of unknown function	152	206
METTOv1_390017	protein of unknown function	152	149
METTOv1_520030	GTP-dependent nucleic acid-binding protein EngD	152	145
METTOv1_560006	membrane protein of unknown function	152	157
METTOv1_700014	exported protein of unknown function	152	177
METTOv1_720013	conserved protein of unknown function	152	119
METTOv1_820005	aminopeptidase N	152	161
METTOv1_40044	conserved protein of unknown function	151	163
METTOv1_50026	Cobyrinic acid ac-diamide synthase	151	174
METTOv1_140048	FeS assembly protein SufD	151	119
METTOv1_190056	putative Thiol-disulfide isomerase and thioredoxins	151	151
METTOv1_210067	anthranilate synthase component I	151	124
METTOv1_230056	protein of unknown function	151	262
METTOv1_310019	putative nitrate transporter component, nrtA	151	154
METTOv1_420008	Peptidoglycan-binding domain 1 protein	151	142
METTOv1_90030	protein of unknown function DUF45	150	148
METTOv1_610002	GtrA family protein (fragment)	150	159
METTOv1_20039	phospho-2-dehydro-3-deoxyheptonate aldolase	149	131
METTOv1_60095	flagellar L-ring protein	149	159
METTOv1_90037	phenylalanyl-tRNA synthetase, beta subunit	149	125
METTOv1_90051	conserved protein of unknown function	149	170
METTOv1_130052	conserved protein of unknown function	149	124
METTOv1_700017	transferase hexapeptide repeat containing protein	149	142
METTOv1_1410002	protein of unknown function	149	243
METTOv1_30065	protein of unknown function	148	158
METTOv1_70084	Seryl-tRNA synthetase	148	142
METTOv1_90077	exported protein of unknown function	148	151
METTOv1_110036	NAD dependent epimerase/dehydratase family protein	148	131

METTOv1_150059	protein of unknown function	148	142
METTOv1_340027	conserved membrane protein of unknown function	148	155
METTOv1_390030	signal transduction histidine kinase, nitrogen specific, NtrB	148	152
METTOv1_520015	transcriptional regulator, BadM/Rrf2 family	148	116
METTOv1_550004	FAD linked oxidase domain protein	148	135
METTOv1_550021	conserved protein of unknown function; putative membrane protein	148	142
METTOv1_1030009	conserved exported protein of unknown function	148	181
METTOv1_20149	Chemotaxis protein cheA	147	138
METTOv1_70064	DNA topoisomerase I	147	143
METTOv1_140009	methionyl-tRNA synthetase	147	171
METTOv1_210003	glucan biosynthesis protein D	147	143
METTOv1_280041	conserved exported protein of unknown function	147	137
METTOv1_350044	Cell wall hydrolase SleB	147	142
METTOv1_620018	transketolase	147	123
METTOv1_170080	pantothenate kinase	146	157
METTOv1_690011	protein of unknown function	146	127
METTOv1_1040002	transposase (fragment)	146	182
METTOv1_1040006	PilT protein domain protein	146	114
METTOv1_70088	exported protein of unknown function	145	162
METTOv1_110044	Dihydroxy-acid dehydratase	145	137
METTOv1_120036	putative Pyruvate carboxyltransferase	145	140
METTOv1_210045	protein of unknown function	145	150
METTOv1_420009	putative sensor histidine kinase with a PAS domain	145	141
METTOv1_630015	Prolyl-tRNA synthetase	145	136
METTOv1_680001	protein of unknown function	145	137
METTOv1_1380002	transposase, IS66 family	145	150
METTOv1_10062	Methylmalonyl-CoA mutase small subunit (MCM-beta)	144	144
METTOv1_20133	putative ferredoxin protein, FixX	144	142
METTOv1_70071	Tyrosyl-tRNA synthetase	144	143
METTOv1_150065	conserved protein of unknown function	144	167
METTOv1_240007	von Willebrand factor type A	144	141
METTOv1_250031	protein of unknown function DUF1044	144	115
METTOv1_300020	ribonuclease D	144	163
METTOv1_340032	2'-5' RNA ligase	144	129
METTOv1_370021	integral membrane sensor signal transduction histidine kinase	144	137
METTOv1_550013	protein of unknown function DUF983	144	146
METTOv1_860009	protein of unknown function	144	147
METTOv1_20015	lytic murein transglycosylase	143	144
METTOv1_120035	Haloacid dehalogenase domain protein hydrolase	143	114
METTOv1_150012	Inositol-phosphate phosphatase	143	142
METTOv1_190001	acetolactate synthase, large subunit, biosynthetic type	143	126
METTOv1_190073	exported protein of unknown function	143	134
METTOv1_220034	Periplasmic protein-like protein	143	131
METTOv1_220064	phosphate uptake regulator, PhoU	143	146
METTOv1_390008	putative multidrug efflux system, outer membrane subunit (efflux pump component) (TolC family)	143	134
METTOv1_660016	exported protein of unknown function	143	160
METTOv1_10071	Acyltransferase 3	142	126
METTOv1_50059	putative Apolipoprotein A1/A4/E	142	145
METTOv1_80019	protein of unknown function	142	170
METTOv1_190072	exported protein of unknown function	142	130
METTOv1_230005	Transcriptional regulator, Fis family	142	192

METTOv1_270064	Patatin	142	120
METTOv1_380019	Undecaprenyl-diphosphatase	142	130
METTOv1_540007	OmpA/MotB domain protein	142	129
METTOv1_10189	conserved membrane protein of unknown function	141	155
METTOv1_60069	putative secretion protein, RND family efflux protein	141	128
METTOv1_160074	exported protein of unknown function	141	150
METTOv1_220037	Transcriptional regulator, MerR family	141	137
METTOv1_230057	putative transcriptional regulator, XRE family	141	158
METTOv1_280024	protein of unknown function	141	140
METTOv1_340024	Glutathione S-transferase domain	141	127
METTOv1_400014	phosphoenolpyruvate carboxylase	141	139
METTOv1_420017	conserved exported protein of unknown function	141	128
METTOv1_470001	conserved membrane protein of unknown function	141	117
METTOv1_620024	protein of unknown function	141	244
METTOv1_650015	conserved protein of unknown function	141	120
METTOv1_100040	protein of unknown function	140	100
METTOv1_160065	protein of unknown function DUF1052	140	106
METTOv1_210033	protein of unknown function	140	124
METTOv1_220018	conserved exported protein of unknown function	140	145
METTOv1_230039	conserved protein of unknown function	140	178
METTOv1_340019	conserved protein of unknown function	140	183
METTOv1_550025	PhoH family protein	140	126
METTOv1_610027	septum site-determining protein MinC	140	144
METTOv1_620016	PfkB domain protein	140	144
METTOv1_20068	protein of unknown function DUF1178	139	137
METTOv1_130053	peptidase S16 lon domain protein	139	149
METTOv1_210036	protein of unknown function	139	142
METTOv1_220014	protein of unknown function	139	128
METTOv1_450024	Two-component response regulator	139	165
METTOv1_10044	4-hydroxy-3-methylbut-2-enyl diphosphate reductase	138	140
METTOv1_50064	conserved protein of unknown function	138	128
METTOv1_150064	putative Haemophilus-specific protein, uncharacterized	138	120
METTOv1_180056	conserved protein of unknown function	138	136
METTOv1_200032	homoserine dehydrogenase	138	104
METTOv1_350015	putative sugar transferase family protein	138	124
METTOv1_370029	protein of unknown function	138	152
METTOv1_400010	conserved protein of unknown function	138	132
METTOv1_910003	Prevent-host-death protein	138	145
METTOv1_1060003	Cytidylate kinase	138	151
METTOv1_20056	protein of unknown function	137	130
METTOv1_40130	pyridoxamine 5'-phosphate oxidase	137	122
METTOv1_60111	putative Type IV pilus assembly PilZ	137	108
METTOv1_90059	Purine nucleotide phosphorylase	137	127
METTOv1_140033	conserved protein of unknown function	137	119
METTOv1_160031	exported protein of unknown function	137	142
METTOv1_220010	HpcH/HpaI aldolase	137	135
METTOv1_240008	MxaA	137	127
METTOv1_270010	protein of unknown function	137	124
METTOv1_400005	conserved protein of unknown function	137	111
METTOv1_480015	conserved exported protein of unknown function	137	149
METTOv1_590003	phosphoribosylformylglycinamidine synthase, purS	137	121
METTOv1_680002	conserved protein of unknown function	137	141
METTOv1_730003	conserved protein of unknown function	137	138

METTOv1_770004	Cell division transporter substrate-binding protein FtsY (Signal recognition particle receptor)	137	114
METTOv1_820004	Chorismate synthase	137	135
METTOv1_830008	conserved protein of unknown function	137	124
METTOv1_860006	exported protein of unknown function	137	144
METTOv1_1070004	Transcriptional regulator, XRE family	137	178
METTOv1_50020	uroporphyrinogen decarboxylase	136	125
METTOv1_60045	conserved membrane protein of unknown function	136	135
METTOv1_80103	conserved protein of unknown function	136	120
METTOv1_110046	outer membrane protein B	136	134
METTOv1_110072	DNA gyrase, B subunit	136	134
METTOv1_160054	protein of unknown function	136	156
METTOv1_190035	protein of unknown function	136	160
METTOv1_230016	protein of unknown function	136	181
METTOv1_310006	conserved protein of unknown function	136	169
METTOv1_570022	conserved exported protein of unknown function	136	149
METTOv1_680013	2,3-bisphosphoglycerate-dependent phosphoglycerate mutase	136	135
METTOv1_770016	helicase domain protein	136	130
METTOv1_1610001	protein of unknown function	136	139
METTOv1_20156	protein of unknown function	135	60
METTOv1_60033	Transcriptional regulator, MerR family	135	109
METTOv1_60137	protein of unknown function	135	132
METTOv1_150040	exported protein of unknown function	135	102
METTOv1_170075	putative Capsule polysaccharide export protein-like	135	125
METTOv1_180061	conserved protein of unknown function	135	129
METTOv1_220015	conserved protein of unknown function	135	98
METTOv1_220062	putative outer-membrane immunogenic protein precursor	135	134
METTOv1_280035	protein of unknown function	135	176
METTOv1_320004	putative phage repressor	135	110
METTOv1_330003	putative replication protein A	135	177
METTOv1_480014	conserved exported protein of unknown function	135	97
METTOv1_590027	cytochrome C biogenesis protein (modular protein)	135	154
METTOv1_810005	exported protein of unknown function	135	110
METTOv1_970008	protein of unknown function	135	127
METTOv1_60121	CheD	134	106
METTOv1_100067	putative C repressor	134	114
METTOv1_110054	Propeptide PepSY amd peptidase M4	134	119
METTOv1_240042	protein of unknown function	134	143
METTOv1_260035	protein of unknown function	134	159
METTOv1_280057	putative Sensor protein	134	118
METTOv1_540004	protein of unknown function	134	151
METTOv1_830014	protein of unknown function	134	116
METTOv1_80097	thioredoxin reductase	133	120
METTOv1_100076	radical SAM domain protein	133	131
METTOv1_210035	isoleucyl-tRNA synthetase	133	121
METTOv1_210049	protein of unknown function	133	234
METTOv1_240038	multi-sensor signal transduction histidine kinase	133	121
METTOv1_530030	Prevent-host-death family protein	133	153
METTOv1_40019	GTP-binding protein Era	132	108
METTOv1_40093	Glyoxalase/bleomycin resistance protein/dioxygenase	132	142
METTOv1_40131	protein of unknown function	132	125
METTOv1_70093	conserved protein of unknown function	132	111
METTOv1_110037	putative membrane protein	132	139

METTOv1_110047	protein of unknown function	132	148
METTOv1_10032	4-hydroxybenzoate polyprenyl transferase	131	114
METTOv1_40004	ErfK/YbiS/YcfS/YnhG family protein	131	115
METTOv1_60089	conserved protein of unknown function	131	130
METTOv1_70068	Tetracenomycin polyketide synthesis 8-O-methyl transferase tcmO	131	123
METTOv1_160007	Phosphatidylserine decarboxylase proenzyme	131	121
METTOv1_250033	Extracellular ligand-binding receptor	131	123
METTOv1_280005	protein of unknown function	131	131
METTOv1_280031	carbamoyl-phosphate synthase, small subunit	131	121
METTOv1_770005	putative intracellular septation protein (ispA/ispZ family), putative membrane protein	131	144
METTOv1_90044	3-oxoacyl-(acyl-carrier-protein) synthase III	130	122
METTOv1_120034	conserved protein of unknown function	130	165
METTOv1_140007	hydrolase, TatD family	130	136
METTOv1_280046	protein of unknown function	130	117
METTOv1_810001	acetylornithine deacetylase (ArgE)	130	120
METTOv1_20034	glucan biosynthesis protein D	129	124
METTOv1_50009	putative citrate lyase beta chain	129	125
METTOv1_110039	Xanthine phosphoribosyltransferase	129	120
METTOv1_110081	3-oxoacyl-(acyl-carrier-protein) synthase III	129	120
METTOv1_160032	protein of unknown function	129	198
METTOv1_280020	lipopolysaccharide transport periplasmic protein LptA	129	146
METTOv1_400022	methionine-R-sulfoxide reductase	129	94
METTOv1_540005	integrase family protein	129	132
METTOv1_640013	ChaC family protein	129	122
METTOv1_830010	protein of unknown function	129	115
METTOv1_10155	homoserine O-acetyltransferase	128	118
METTOv1_10183	putative cation efflux system protein (CzcA/CusA-like)	128	119
METTOv1_40011	alpha-L-glutamate ligase, RimK family	128	111
METTOv1_40057	protein of unknown function	128	124
METTOv1_40084	Transcriptional regulator, LysR family	128	107
METTOv1_50092	LemA family protein	128	149
METTOv1_80100	glycyl-tRNA synthetase, beta subunit	128	136
METTOv1_160056	protein of unknown function	128	108
METTOv1_370015	Glutathione S-transferase domain protein	128	116
METTOv1_450002	conserved protein of unknown function	128	142
METTOv1_530026	DedA family	128	131
METTOv1_1420003	Integrase, catalytic region	128	146
METTOv1_70061	Glycosyl transferase family 2	127	123
METTOv1_120039	protein of unknown function	127	134
METTOv1_130021	protein of unknown function	127	155
METTOv1_200021	transposase	127	120
METTOv1_300019	exported protein of unknown function	127	151
METTOv1_720008	Peptidoglycan glycosyltransferase	127	121
METTOv1_10037	conserved protein of unknown function containing two histidine-rich domains; putative signal peptide	126	125
METTOv1_20110	protein of unknown function	126	160
METTOv1_40102	Hsp33-like chaperonin	126	123
METTOv1_90019	Zinc finger, SWIM-type (modular protein)	126	132
METTOv1_640009	conserved protein of unknown function	126	118
METTOv1_960005	PilT protein domain protein	126	112
METTOv1_970015	hopanoid-associated sugar epimerase	126	120

METTOv1_130004	conserved protein of unknown function	125	189
METTOv1_180033	1-deoxy-D-xylulose 5-phosphate reductoisomerase	125	93
METTOv1_190014	protein of unknown function DUF1058	125	120
METTOv1_240020	protein of unknown function	125	90
METTOv1_270013	protein of unknown function	125	115
METTOv1_450022	Serine-type D-Ala-D-Ala carboxypeptidase	125	111
METTOv1_590010	Transcriptional regulator protein	125	90
METTOv1_590013	exodeoxyribonuclease VII, small subunit	125	142
METTOv1_780016	protein of unknown function	125	149
METTOv1_20120	protein of unknown function DUF683	124	224
METTOv1_20182	Putative plasmid maintenance system antidote protein, XRE family (fragment)	124	161
METTOv1_80020	Leucyl-tRNA synthetase	124	113
METTOv1_110074	conserved protein of unknown function	124	111
METTOv1_150048	protein of unknown function	124	145
METTOv1_240006	protein of unknown function	124	132
METTOv1_270008	protein of unknown function	124	161
METTOv1_310049	tRNA pseudouridine synthase A	124	125
METTOv1_10036	conserved protein of unknown function	123	127
METTOv1_10120	putative outer membrane efflux protein	123	126
METTOv1_60040	transglutaminase family protein cysteine peptidase BTLCP	123	131
METTOv1_150061	Pyridoxal-5'-phosphate-dependent protein beta subunit	123	105
METTOv1_170082	Imidazole glycerol phosphate synthase subunit hisF	123	107
METTOv1_200042	acetoacetate decarboxylase	123	114
METTOv1_220065	phosphate ABC transporter, ATPase subunit	123	103
METTOv1_620006	putative methyl-accepting chemotaxis sensory transducer	123	108
METTOv1_780007	Hopene-associated glycosyltransferase HpnB	123	119
METTOv1_1030008	conserved exported protein of unknown function	123	128
METTOv1_10170	MOSC domain containing protein	122	112
METTOv1_60125	Methyl-accepting chemotaxis sensory transducer	122	128
METTOv1_140081	membrane protein of unknown function	122	111
METTOv1_150037	PTSINtr with GAF domain, PtsP	122	108
METTOv1_270003	exported protein of unknown function	122	91
METTOv1_340047	protein of unknown function	122	115
METTOv1_380050	antitoxin of the YoeB-YefM toxin-antitoxin system	122	135
METTOv1_600005	protein of unknown function	122	122
METTOv1_600013	exported protein of unknown function	122	117
METTOv1_610005	Beta-ketoacyl synthase	122	105
METTOv1_780001	N-acetylmuramoyl-L-alanine amidase (fragment)	122	123
METTOv1_1230002	protein of unknown function	122	135
METTOv1_50100	protein of unknown function	121	98
METTOv1_60102	type III secretion system inner membrane R protein	121	146
METTOv1_80067	conserved exported protein of unknown function	121	101
METTOv1_110071	protein of unknown function	121	140
METTOv1_160050	protein of unknown function DUF330	121	100
METTOv1_390007	efflux transporter, RND family, MFP subunit	121	104
METTOv1_400034	fragment of protein of unknown function (part 1)	121	90
METTOv1_590004	phosphoribosylformylglycinamidine synthase I	121	98
METTOv1_720001	UDP-N-acetylmuramate--L-alanine ligase	121	119
METTOv1_720004	UDP-N-acetylmuramoyl-L-alanyl-D-glutamate synthetase	121	118
METTOv1_140070	exodeoxyribonuclease III Xth	120	109
METTOv1_150021	Chorismate mutase	120	112
METTOv1_220025	short-chain dehydrogenase/reductase SDR	120	98

METTOv1_310003	protein of unknown function	120	172
METTOv1_460011	Cation efflux system protein	120	105
METTOv1_550005	exported protein of unknown function	120	125
METTOv1_590005	putative Mg2+ transporter protein, CorA-like	120	100
METTOv1_640022	protein of unknown function	120	111
METTOv1_20096	nitrogenase cofactor biosynthesis protein NifB	119	141
METTOv1_50095	aldo/keto reductase	119	121
METTOv1_80014	Cytochrome c, class I	119	124
METTOv1_80018	poly(R)-hydroxyalkanoic acid synthase, class I	119	124
METTOv1_110048	Acetyltransferase (Isoleucine patch superfamily)-like protein	119	148
METTOv1_120031	nucleotide sugar epimerase; putative Capsular polysaccharide biosynthesis protein	119	113
METTOv1_170088	Imidazoleglycerol-phosphate dehydratase	119	112
METTOv1_960001	RNA-binding S4 domain protein (fragment)	119	103
METTOv1_980014	Uncharacterized HTH-type transcriptional regulator y4mF (modular protein)	119	153
METTOv1_1090002	putative sugar hydrolase/Beta-N-acetylhexosaminidase	119	108
METTOv1_40098	NAD(P)(+) transhydrogenase (AB-specific)	118	125
METTOv1_80012	protein of unknown function DUF1332	118	113
METTOv1_180081	Integral membrane protein TerC	118	104
METTOv1_190007	protein of unknown function	118	127
METTOv1_200015	conserved membrane protein of unknown function	118	104
METTOv1_280032	Argininosuccinate lyase	118	113
METTOv1_370030	protein of unknown function	118	96
METTOv1_400004	conserved protein of unknown function	118	146
METTOv1_420010	exported protein of unknown function	118	77
METTOv1_700016	transglutaminase family protein cysteine peptidase BTLCP	118	94
METTOv1_940007	conserved protein of unknown function	118	114
METTOv1_20035	glucosyltransferase MdoH	117	108
METTOv1_20105	DegT/DnrJ/EryC1/StrS aminotransferase	117	148
METTOv1_40032	Rv0623-like transcription factor	117	96
METTOv1_40082	penicillin-insensitive murein endopeptidase	117	109
METTOv1_80092	protein of unknown function	117	128
METTOv1_110086	molybdopterin biosynthesis MoaE protein	117	90
METTOv1_130020	protein of unknown function	117	124
METTOv1_300040	protein of unknown function	117	110
METTOv1_350039	protein of unknown function	117	113
METTOv1_410004	protein-(glutamine-N5) methyltransferase, release factor-specific	117	113
METTOv1_640021	conserved protein of unknown function	117	68
METTOv1_680011	molybdenum cofactor synthesis domain protein	117	99
METTOv1_720006	UDP-N-acetylmuramoylalanyl-D-glutamyl-2, 6-diaminopimelate/D-alanyl-D-alanyl ligase	117	111
METTOv1_780005	K potassium transporter	117	104
METTOv1_980010	conserved protein of unknown function	117	142
METTOv1_1090003	chromosome segregation and condensation protein ScpA	117	120
METTOv1_1170004	transposase (fragment)	117	86
METTOv1_1350001	conserved protein of unknown function	117	119
METTOv1_20084	conserved protein of unknown function	116	82
METTOv1_110001	protein of unknown function zinc metallopeptidase putative	116	122
METTOv1_120041	protein of unknown function	116	126
METTOv1_130043	conserved exported protein of unknown function	116	112
METTOv1_190060	NMT1/THI5 like domain protein	116	108

METTOv1_200022	Acetoacetyl-coenzyme A synthetase	116	114
METTOv1_420001	MltA-interacting MipA family protein	116	91
METTOv1_630007	conserved protein of unknown function	116	117
METTOv1_730009	conserved protein of unknown function	116	143
METTOv1_810018	protein of unknown function	116	104
METTOv1_1730001	protein of unknown function	116	117
METTOv1_20087	histidinol-phosphate aminotransferase	115	100
METTOv1_20146	Chemotaxis protein methyltransferase	115	102
METTOv1_30136	conserved exported protein of unknown function	115	88
METTOv1_50057	protein of unknown function	115	149
METTOv1_60119	chemotaxis-specific methylesterase	115	113
METTOv1_150051	protein of unknown function	115	129
METTOv1_350036	Prephenate dehydratase	115	132
METTOv1_370045	Cysteinyl-tRNA synthetase	115	122
METTOv1_630014	exported protein of unknown function	115	91
METTOv1_40006	conserved protein of unknown function	114	116
METTOv1_50034	arginine biosynthesis bifunctional protein ArgJ	114	115
METTOv1_100066	protein of unknown function	114	101
METTOv1_140065	dTMP kinase	114	113
METTOv1_210041	arsenate reductase	114	107
METTOv1_280067	conserved protein of unknown function	114	119
METTOv1_450023	cobalamin synthesis protein P47K	114	104
METTOv1_490006	Cobyrinic acid a,c-diamide synthase	114	99
METTOv1_700015	exported protein of unknown function	114	138
METTOv1_1000008	PilT protein domain protein	114	105
METTOv1_1240006	protein of unknown function	114	93
METTOv1_60004	sodium:dicarboxylate symporter	113	123
METTOv1_90018	protein of unknown function	113	119
METTOv1_90070	aspartate/glutamate/uridylate kinase	113	110
METTOv1_130036	thiamine biosynthesis protein ThiC	113	108
METTOv1_560024	cytochrome B561	113	124
METTOv1_720003	cell cycle protein	113	88
METTOv1_30012	conserved protein of unknown function	112	100
METTOv1_30154	ribonucleotide-diphosphate reductase subunit alpha	112	105
METTOv1_40103	Histidyl-tRNA synthetase	112	115
METTOv1_50005	protein of unknown function	112	82
METTOv1_130011	Site-specific recombinase, phage integrase family (fragment)	112	95
METTOv1_150034	conserved protein of unknown function	112	112
METTOv1_190061	Creatininase	112	97
METTOv1_200062	exported protein of unknown function	112	112
METTOv1_340033	phospholipid/glycerol acyltransferase	112	109
METTOv1_430002	cobalt chelatase, pCobT subunit	112	93
METTOv1_540019	protein of unknown function	112	137
METTOv1_20134	valyl-tRNA synthetase	111	98
METTOv1_30016	protein of unknown function	111	109
METTOv1_40080	conserved exported protein of unknown function	111	107
METTOv1_50088	exported protein of unknown function	111	108
METTOv1_60002	Putative Copper resistance protein, copC family	111	99
METTOv1_110063	uridylate kinase	111	128
METTOv1_210030	Ubiquinone biosynthesis hydroxylase, UbiH/UbiF/VisC/COQ6 family	111	95
METTOv1_210040	Phosphoglucosamine mutase	111	104
METTOv1_210048	protein of unknown function	111	163

METTOv1_340015	protein of unknown function	111	160
METTOv1_400006	conserved exported protein of unknown function	111	101
METTOv1_420019	protein of unknown function	111	127
METTOv1_510024	transcriptional regulator, ModE family	111	96
METTOv1_570010	conserved protein of unknown function	111	126
METTOv1_1640001	transposase	111	104
METTOv1_10028	inositol monophosphatase	110	101
METTOv1_110006	conserved protein of unknown function	110	111
METTOv1_120009	protein of unknown function	110	129
METTOv1_130055	tRNA (guanine-N(7)-)-methyltransferase (fragment)	110	110
METTOv1_150010	inosine-5'-monophosphate dehydrogenase	110	91
METTOv1_200001	membrane protein of unknown function	110	135
METTOv1_390014	phosphoribosylaminoimidazolecarboxamide formyltransferase/IMP cyclohydrolase	110	99
METTOv1_480031	protein of unknown function	110	107
METTOv1_620015	exported protein of unknown function	110	91
METTOv1_620020	exported protein of unknown function	110	110
METTOv1_630009	protein of unknown function	110	81
METTOv1_640024	exported protein of unknown function	110	107
METTOv1_1480002	transposase	110	92
METTOv1_90090	acyl-CoA dehydrogenase domain protein	109	102
METTOv1_110070	protein of unknown function	109	157
METTOv1_190004	Transcriptional modulator of MazE/toxin, MazF	109	109
METTOv1_190010	3-dehydroquinate synthase	109	104
METTOv1_220055	Glyoxalase/bleomycin resistance protein/dioxygenase	109	87
METTOv1_280039	Phosphoribosylformylglycinamidine synthase 2	109	99
METTOv1_320019	protein of unknown function	109	110
METTOv1_430004	protein of unknown function	109	111
METTOv1_590029	UDP-3-O-acyl N-acetylglucosamine deacetylase	109	90
METTOv1_640014	protein of unknown function	109	99
METTOv1_710009	Polyketide biosynthesis protein pksE [Includes: Malonyl CoA-acyl carrier protein transacylase] (fragment)	109	81
METTOv1_30059	protein of unknown function	108	99
METTOv1_40033	ABC transporter related	108	103
METTOv1_90052	Gamma-glutamyl phosphate reductase	108	95
METTOv1_110002	metallophosphoesterase	108	94
METTOv1_150049	3-deoxy-7-phosphoheptulonate synthase	108	112
METTOv1_160069	protein of unknown function DUF498	108	94
METTOv1_360006	protein of unknown function	108	112
METTOv1_430025	two component transcriptional regulator, LuxR family	108	137
METTOv1_460006	protein of unknown function	108	118
METTOv1_1060002	3-phosphoshikimate 1-carboxyvinyltransferase	108	108
METTOv1_10190	conserved exported protein of unknown function	107	139
METTOv1_20065	Nitrilase/cyanide hydratase and apolipoprotein N-acyltransferase	107	89
METTOv1_30158	PilT protein domain protein	107	94
METTOv1_70085	5'-nucleotidase surE	107	106
METTOv1_80025	NADP-dependent malic enzyme	107	106
METTOv1_120033	protein of unknown function	107	111
METTOv1_190041	conserved protein of unknown function	107	99
METTOv1_220042	protein of unknown function	107	117
METTOv1_240034	Peptidyl-tRNA hydrolase	107	104
METTOv1_250032	Transcriptional regulator	107	99

METTOv1_350051	cell divisionFtsK/SpoIIIE	107	111
METTOv1_420002	lysyl-tRNA synthetase	107	107
METTOv1_420014	extracellular solute-binding protein family 5	107	99
METTOv1_530021	DNA polymerase III, subunits gamma and tau	107	99
METTOv1_550023	CBS domain containing protein	107	117
METTOv1_640019	phosphoribosylaminoimidazole carboxylase, ATPase subunit	107	82
METTOv1_30085	protein of unknown function	106	132
METTOv1_60020	nitrogen-fixing NifU domain protein	106	106
METTOv1_80016	putative PLP-dependent aminotransferase, putative aspartate aminotransferase.	106	88
METTOv1_80039	Mn2+/Fe2+ transporter, NRAMP family	106	101
METTOv1_90003	conserved protein of unknown function	106	74
METTOv1_130030	Short-chain dehydrogenase/reductase SDR	106	116
METTOv1_140014	exported protein of unknown function	106	100
METTOv1_270012	putative Lipopolysaccharide biosynthesis protein	106	111
METTOv1_310012	hydrogenase 4 subunit F	106	108
METTOv1_310027	40-residue YVTN family beta-propeller repeat protein	106	100
METTOv1_600020	protein of unknown function DUF155	106	109
METTOv1_710008	Methyltransferase type 11	106	101
METTOv1_10166	conserved protein of unknown function	105	88
METTOv1_60133	exported protein of unknown function	105	103
METTOv1_180079	Diaminopimelate epimerase	105	98
METTOv1_350049	conserved exported protein of unknown function	105	76
METTOv1_440041	putative Predicted Na+-dependent transporter	105	93
METTOv1_500008	efflux transporter, RND family, MFP subunit	105	102
METTOv1_540010	non-ribosomal peptide synthetase	105	96
METTOv1_800021	transcriptional regulator, AraC family	105	110
METTOv1_1230004	conserved protein of unknown function	105	98
METTOv1_1320002	Transcriptional repressor nrdR	105	105
METTOv1_20027	Methyltransferase, UbiE/COQ5 family	104	108
METTOv1_20102	NifZ family protein	104	68
METTOv1_20112	nitrogenase reductase	104	106
METTOv1_50021	protein of unknown function	104	110
METTOv1_50098	serine acetyltransferase	104	123
METTOv1_60127	periplasmic protein thiol/disulphide oxidoreductase DsbE	104	105
METTOv1_90075	putative Exopolysaccharide production protein exoZ	104	120
METTOv1_110035	exported protein of unknown function	104	127
METTOv1_130002	phosphoenolpyruvate carboxylase	104	89
METTOv1_160014	conserved protein of unknown function	104	104
METTOv1_180025	lipolytic protein G-D-S-L family	104	105
METTOv1_230006	conserved protein of unknown function	104	95
METTOv1_380049	toxin of the YoeB-YefM toxin-antitoxin system	104	83
METTOv1_460005	Sodium/hydrogen exchanger	104	96
METTOv1_510023	NADPH-dependent 7-cyano-7-deazaguanine reductase	104	115
METTOv1_10157	putative Transcriptional activator	103	114
METTOv1_20176	protein of unknown function	103	122
METTOv1_30109	Camphor resistance protein CrcB	103	87
METTOv1_60009	molybdenum cofactor biosynthesis protein C	103	68
METTOv1_80054	protein of unknown function	103	123
METTOv1_210042	DinB family protein	103	96
METTOv1_360038	Transcriptional regulator, XRE family	103	106
METTOv1_620026	protein of unknown function	103	147
METTOv1_1640002	conserved protein of unknown function	103	116

METTOv1_20044	putative H/ACA ribonucleoprotein complex subunit 1	102	104
METTOv1_60041	conserved protein of unknown function	102	115
METTOv1_90025	Exopolysaccharide biosynthesis polyprenyl glycosylphosphotransferase	102	91
METTOv1_100075	putative 6-pyruvoyltetrahydropterin synthase	102	130
METTOv1_170063	exported protein of unknown function	102	103
METTOv1_200012	ABC transporter related	102	121
METTOv1_200061	excinuclease ABC, A subunit	102	101
METTOv1_220040	UBA/THIF-type NAD/FAD binding protein	102	73
METTOv1_220061	exported protein of unknown function	102	102
METTOv1_260045	conserved protein of unknown function	102	124
METTOv1_310026	protein of unknown function	102	79
METTOv1_350029	Acylphosphatase (modular protein)	102	97
METTOv1_400027	Extracellular solute-binding protein family 3	102	96
METTOv1_430022	putative TPR repeat-containing protein	102	93
METTOv1_740007	conserved protein of unknown function	102	136
METTOv1_770013	conserved membrane protein of unknown function	102	85
METTOv1_1500002	conserved protein of unknown function	102	87
METTOv1_10164	DNA repair protein recO	101	100
METTOv1_50050	transglutaminase domain protein	101	96
METTOv1_80044	conserved protein of unknown function; putative exported protein	101	78
METTOv1_100061	Phosphoglycerate mutase (modular protein)	101	99
METTOv1_240046	glycine oxidase ThiO	101	102
METTOv1_260043	protein of unknown function	101	145
METTOv1_340042	pyruvate dehydrogenase complex dihydrolipoamide acetyltransferase (fragment)	101	106
METTOv1_350041	conserved protein of unknown function	101	87
METTOv1_390027	Methyl-accepting chemotaxis sensory transducer (modular protein)	101	106
METTOv1_400038	dihydrolipoamide dehydrogenase	101	81
METTOv1_440035	aspartate carbamoyltransferase catalytic subunit	101	98
METTOv1_540013	protein of unknown function	101	99
METTOv1_10156	methionine biosynthesis protein MetW	100	92
METTOv1_20011	conserved protein of unknown function	100	80
METTOv1_40078	conserved protein of unknown function	100	101
METTOv1_50061	protein of unknown function	100	114
METTOv1_80074	globin family protein (fragment)	100	82
METTOv1_90060	MaoC domain protein dehydratase	100	82
METTOv1_100058	cobalamin biosynthesis protein CobW	100	85
METTOv1_110050	protein of unknown function	100	93
METTOv1_130039	conserved exported protein of unknown function	100	111
METTOv1_140045	aminotransferase class V	100	97
METTOv1_150013	GCN5-related N-acetyltransferase	100	80
METTOv1_150016	delta-aminolevulinic acid dehydratase	100	79
METTOv1_230013	conserved protein of unknown function	100	97
METTOv1_360002	methionyl-tRNA formyltransferase	100	96
METTOv1_440032	transcriptional regulator, AsnC family	100	106
METTOv1_540014	Cytochrome d ubiquinol oxidase subunit 1 (Cytochrome d ubiquinol oxidase subunit I) (Cytochrome bd-I oxidase subunit I)	100	101
METTOv1_40005	conserved protein of unknown function; putative exported protein	99	95
METTOv1_120005	protein of unknown function	99	75

METTOv1_130064	protein of unknown function	99	101
METTOv1_140071	exported protein of unknown function	99	103
METTOv1_190043	conserved protein of unknown function; putative membrane protein	99	111
METTOv1_280007	putative nitrogen regulatory protein (NtrR-like), putative virulence associated protein (vap)	99	89
METTOv1_340031	multifunctional: acyl-CoA thioesterase I; protease I; lysophospholipaseL(I)	99	93
METTOv1_340040	protein of unknown function	99	112
METTOv1_520011	DNA topoisomerase IV subunit B	99	97
METTOv1_680012	Dihydrodipicolinate reductase	99	76
METTOv1_830015	membrane protein of unknown function	99	108
METTOv1_1050010	ADP-ribose pyrophosphatase	99	119
METTOv1_100025	conserved exported protein of unknown function	98	115
METTOv1_400023	amino acid permease-associated region	98	88
METTOv1_500007	putative HTH-type transcriptional repressor acnR	98	90
METTOv1_540006	acriflavin resistance protein	98	92
METTOv1_550020	TonB-dependent receptor	98	96
METTOv1_810016	conserved membrane protein of unknown function	98	91
METTOv1_1080003	protein of unknown function DUF1007	98	82
METTOv1_1120005	protein of unknown function	98	86
METTOv1_10128	tryptophan synthase subunit beta	97	91
METTOv1_20005	putative Response regulator receiver domain protein (CheY-like)	97	92
METTOv1_30080	protein of unknown function	97	113
METTOv1_40117	trehalose-6-phosphate phosphatase, biosynthetic	97	88
METTOv1_60114	CheW protein	97	111
METTOv1_90045	MiaB-like tRNA modifying enzyme YliG	97	91
METTOv1_120002	Radical SAM domain protein	97	96
METTOv1_300006	Ribosomal RNA large subunit methyltransferase N	97	86
METTOv1_1030007	protein of unknown function	97	90
METTOv1_1060008	Peptidase M23	97	103
METTOv1_20183	Plasmid maintenance system killer	96	114
METTOv1_30127	biotin/acetyl-CoA-carboxylase ligase	96	94
METTOv1_40134	putative HspC2 heat shock protein	96	115
METTOv1_70065	conserved protein of unknown function	96	87
METTOv1_70075	3-demethylubiquinone-9 3-methyltransferase	96	86
METTOv1_100070	fatty acid hydroxylase	96	82
METTOv1_170085	1-(5-phosphoribosyl)-5-[(5-phosphoribosylamino)m ethylideneamino] imidazole-4-carboxamide isomerase	96	89
METTOv1_180068	5'-methylthioadenosine phosphorylase	96	87
METTOv1_190011	protein of unknown function DUF21	96	103
METTOv1_230038	protein of unknown function	96	126
METTOv1_270011	Undecaprenyl-phosphate alpha N-acetylglucosaminyltransferase	96	103
METTOv1_300029	integral membrane sensor signal transduction histidine kinase	96	82
METTOv1_320002	membrane protein of unknown function	96	86
METTOv1_430009	conserved protein of unknown function; putative signal peptide	96	72
METTOv1_430010	exported protein of unknown function	96	77
METTOv1_460001	conserved protein of unknown function	96	142
METTOv1_550030	protein of unknown function	96	99
METTOv1_560008	protein of unknown function	96	102
METTOv1_610004	ribosomal L11 methyltransferase	96	83
METTOv1_620005	conserved exported protein of unknown function	96	96
METTOv1_830005	putative Virulence-associated protein	96	64

METTOv1_10045	Homoserine kinase	95	97
METTOv1_20086	protein of unknown function	95	82
METTOv1_40087	conserved exported protein of unknown function	95	90
METTOv1_70025	protein of unknown function	95	112
METTOv1_80013	short-chain dehydrogenase/reductase SDR	95	84
METTOv1_130029	nickel and cobalt resistance	95	84
METTOv1_170077	Glycosyltransferase protein (modular protein)	95	78
METTOv1_190029	conserved protein of unknown function	95	91
METTOv1_270017	putative Polysaccharide biosynthesis protein	95	101
METTOv1_710006	PilT protein domain protein	95	112
METTOv1_960004	Metabolite transport protein	95	98
METTOv1_10119	transcriptional regulatory protein	94	95
METTOv1_30106	DNA repair protein RadC	94	67
METTOv1_60034	cytochrome oxidase assembly	94	87
METTOv1_60078	exported protein of unknown function	94	85
METTOv1_70005	protein of unknown function	94	112
METTOv1_240051	protein of unknown function	94	86
METTOv1_250023	protein of unknown function	94	102
METTOv1_270062	transcriptional regulator	94	85
METTOv1_340036	cell division ATP-binding protein FtsE	94	95
METTOv1_440045	beta-ribofuranosylaminobenzene 5'-phosphate synthase family	94	80
METTOv1_640008	protein of unknown function	94	110
METTOv1_820007	magnesium and cobalt transport protein CorA	94	83
METTOv1_1020007	protein of unknown function DUF192	94	71
METTOv1_1490001	fragment of putative transposase (part 2)	94	87
METTOv1_10049	conserved protein of unknown function	93	86
METTOv1_10169	FAD dependent oxidoreductase	93	80
METTOv1_20153	Extensin family protein	93	83
METTOv1_20167	protein of unknown function	93	162
METTOv1_50003	exported protein of unknown function	93	79
METTOv1_60003	conserved protein of unknown function	93	91
METTOv1_60117	CheA signal transduction histidine kinase	93	87
METTOv1_100056	cob(I)alamin adenosyltransferase	93	105
METTOv1_110064	4Fe-4S ferredoxin iron-sulfur binding domain protein	93	76
METTOv1_190033	conserved protein of unknown function	93	106
METTOv1_240037	protein of unknown function UPF0079	93	96
METTOv1_280069	N-acetylmuramoyl-L-alanine amidase (fragment)	93	74
METTOv1_360032	conserved protein of unknown function	93	99
METTOv1_410035	flagellum-specific ATP synthase	93	83
METTOv1_500023	protein of unknown function	93	91
METTOv1_550017	threonine synthase	93	83
METTOv1_550026	(dimethylallyl)adenosine tRNA methylthiotransferase	93	100
METTOv1_910004	PilT protein-like	93	115
METTOv1_930008	conserved protein of unknown function	93	119
METTOv1_10142	heat-inducible transcription repressor	92	91
METTOv1_60122	conserved protein of unknown function	92	66
METTOv1_130026	conserved protein of unknown function	92	84
METTOv1_150035	protein of unknown function	92	97
METTOv1_170006	CRISPR-associated protein, Cas2 family	92	125
METTOv1_180041	protein of unknown function	92	89
METTOv1_210024	Lysine--tRNA ligase	92	101
METTOv1_390025	cyclase/dehydrase (modular protein)	92	81
METTOv1_410022	protein of unknown function	92	105

METTOv1_530015	Protein tolB precursor	92	101
METTOv1_780004	conserved protein of unknown function	92	94
METTOv1_40022	Holo-[acyl-carrier-protein] synthase	91	101
METTOv1_40052	ABC transporter related	91	94
METTOv1_50110	integral membrane sensor signal transduction histidine kinase	91	86
METTOv1_80102	exported protein of unknown function	91	87
METTOv1_310010	hydrogenase-3 subunit G	91	69
METTOv1_340017	exported protein of unknown function	91	83
METTOv1_350018	Lipopolysaccharide biosynthesis protein	91	91
METTOv1_380042	Endoribonuclease L-PSP	91	81
METTOv1_390015	protein of unknown function	91	94
METTOv1_620007	protein of unknown function	91	52
METTOv1_820011	fragment of integral membrane sensor signal transduction histidine kinase (part 1)	91	97
METTOv1_830009	conserved membrane protein of unknown function	91	54
METTOv1_40063	metallophosphoesterase	90	73
METTOv1_60032	putative Methyltransferase, FkbM family protein	90	104
METTOv1_60094	putative Flagellar basal body-associated protein FliL	90	93
METTOv1_140006	beta-lactamase domain protein	90	75
METTOv1_190028	GCN5-related N-acetyltransferase	90	92
METTOv1_210004	membrane protein of unknown function	90	87
METTOv1_220038	conserved protein of unknown function	90	80
METTOv1_300018	Ppx/GppA phosphatase	90	76
METTOv1_420015	binding-protein-dependent transport systems inner membrane component	90	106
METTOv1_490018	putative tonB-dependent receptor protein	90	82
METTOv1_620014	2-polyprenylphenol 6-hydroxylase	90	98
METTOv1_630003	fosmidomycin resistance protein	90	86
METTOv1_660018	protein of unknown function	90	87
METTOv1_10026	Three-deoxy-D-manno-octulosonic-acid transferase domain protein	89	81
METTOv1_20058	endonuclease III	89	66
METTOv1_130044	conserved exported protein of unknown function	89	108
METTOv1_160006	CDP-diacylglycerol/serine O-phosphatidyltransferase	89	79
METTOv1_180030	UDP-3-O-(3-hydroxymyristoyl) glucosamine N-acyltransferase LpxD	89	64
METTOv1_180045	type II secretion system protein E	89	78
METTOv1_230037	protein of unknown function	89	150
METTOv1_350016	putative glycosyl transferase, group 1	89	88
METTOv1_420023	NLP/P60 protein	89	92
METTOv1_690015	Scaffold protein Nfu/NifU	89	90
METTOv1_930007	conserved protein of unknown function	89	93
METTOv1_30079	protein of unknown function	88	115
METTOv1_40023	pyridoxine 5'-phosphate synthase	88	72
METTOv1_50008	Carbonate dehydratase	88	81
METTOv1_120032	exported protein of unknown function	88	84
METTOv1_150028	protein of unknown function DUF205	88	96
METTOv1_160068	putative phytoene synthase (terpenoid synthase)	88	76
METTOv1_200006	ATPase associated with various cellular activities AAA_3	88	83
METTOv1_240036	nucleotidyl transferase	88	88
METTOv1_280053	Enoyl-CoA hydratase/isomerase	88	78
METTOv1_310015	hydrogenase 4 subunit B	88	82
METTOv1_350013	protein of unknown function DUF423	88	71

METTOv1_550029	Glucose-6-phosphate isomerase	88	84
METTOv1_690022	conserved protein of unknown function; putative proteasome-type protease domain	88	76
METTOv1_720007	UDP-N-acetylmuramyl-tripeptide synthetase	88	80
METTOv1_780017	protein of unknown function	88	107
METTOv1_20101	NifZ family protein	87	99
METTOv1_90004	conserved protein of unknown function	87	109
METTOv1_90032	Phosphate butyryltransferase	87	81
METTOv1_170086	imidazole glycerol phosphate synthase, glutamine amidotransferase subunit	87	79
METTOv1_340030	ABC transporter related	87	101
METTOv1_340038	putative integron gene cassette protein	87	86
METTOv1_590025	Cytochrome c-type biogenesis protein ccmE	87	84
METTOv1_810017	conserved protein of unknown function	87	139
METTOv1_880005	exported protein of unknown function	87	83
METTOv1_10040	glutamate/cysteine ligase	86	64
METTOv1_40007	GCN5-related N-acetyltransferase	86	74
METTOv1_40037	Glycine cleavage system H protein	86	85
METTOv1_60014	protein of unknown function	86	118
METTOv1_80101	protein of unknown function	86	103
METTOv1_170064	protein of unknown function	86	132
METTOv1_260036	conserved protein of unknown function	86	90
METTOv1_340008	Methylase involved in ubiquinone/menaquinone biosynthesis	86	105
METTOv1_360039	Phage-related protein	86	65
METTOv1_530027	exported protein of unknown function	86	62
METTOv1_10176	putative transcriptional regulator, ModE family	85	73
METTOv1_20026	cytochrome c biogenesis protein transmembrane region	85	81
METTOv1_20109	ferredoxin, 2Fe-2S (AaFd4)	85	91
METTOv1_40031	PilT protein domain protein	85	85
METTOv1_140053	Phosphoribosylformylglycinamidine cyclo-ligase	85	70
METTOv1_180039	exported protein of unknown function	85	93
METTOv1_190074	exported protein of unknown function	85	86
METTOv1_300041	putative TadE family protein	85	58
METTOv1_340011	membrane protein of unknown function	85	155
METTOv1_340028	conserved protein of unknown function	85	99
METTOv1_630010	protein of unknown function DUF339	85	71
METTOv1_650004	membrane protein of unknown function	85	72
METTOv1_10208	excinuclease ABC, B subunit	84	81
METTOv1_40128	protein of unknown function	84	110
METTOv1_70056	Tetratricopeptide TPR_2 repeat protein	84	85
METTOv1_130023	peptidase M24	84	75
METTOv1_130031	heavy metal translocating P-type ATPase	84	88
METTOv1_140034	multi-sensor hybrid histidine kinase	84	77
METTOv1_180067	protein of unknown function	84	94
METTOv1_250001	conserved protein of unknown function	84	75
METTOv1_260022	conserved exported protein of unknown function	84	143
METTOv1_280045	protein of unknown function DUF710	84	88
METTOv1_280050	Class I peptide chain release factor	84	90
METTOv1_310001	glutathione synthetase	84	83
METTOv1_310011	hydrogenlyase component	84	86
METTOv1_340022	conserved protein of unknown function	84	73
METTOv1_380047	conserved protein of unknown function	84	66

METTOv1_420016	binding-protein-dependent transport systems inner membrane component	84	88
METTOv1_420034	conserved protein of unknown function	84	80
METTOv1_570008	Oxygen-independent coproporphyrinogen-III oxidase	84	62
METTOv1_620001	sulfate ABC transporter, ATPase subunit	84	64
METTOv1_660010	putative acetylase	84	82
METTOv1_690017	GCN5-related N-acetyltransferase	84	90
METTOv1_10158	UvrD/REP helicase	83	69
METTOv1_20108	protein of unknown function	83	79
METTOv1_40043	putative Glycosyl transferase family 2	83	81
METTOv1_50072	Methyltransferase FkbM family	83	65
METTOv1_110004	conserved protein of unknown function	83	87
METTOv1_170004	CRISPR-associated endonuclease, Csn1 family	83	93
METTOv1_180043	putative Fimbrial assembly family protein	83	69
METTOv1_190017	ATPase, RecF-like protein	83	80
METTOv1_200010	short-chain dehydrogenase/reductase SDR	83	96
METTOv1_280009	Sodium/hydrogen exchanger	83	79
METTOv1_280028	Plasmid stabilization system (fragment)	83	113
METTOv1_360019	B12-dependent methionine synthase	83	73
METTOv1_400025	protein of unknown function DUF185	83	68
METTOv1_460025	putative Two-component transcriptional regulator	83	105
METTOv1_520003	DNA topoisomerase IV subunit A	83	76
METTOv1_520020	putative sugar kinase	83	78
METTOv1_750005	protein of unknown function	83	122
METTOv1_780009	squalene synthase HpnD	83	74
METTOv1_20075	conserved membrane protein of unknown function	82	101
METTOv1_20118	nitrogen fixation protein NifX	82	65
METTOv1_60016	conserved protein of unknown function	82	98
METTOv1_60093	flagellar biosynthetic protein FliP	82	78
METTOv1_90040	exported protein of unknown function	82	67
METTOv1_130045	Thioredoxin family protein (modular protein)	82	74
METTOv1_200036	exported protein of unknown function	82	50
METTOv1_210031	protein of unknown function	82	74
METTOv1_340021	Glutathione S-transferase domain protein	82	55
METTOv1_600027	exported protein of unknown function	82	62
METTOv1_620004	sulfate ABC transporter, periplasmic sulfate-binding protein	82	59
METTOv1_690016	peptidase M22 glycoprotease	82	78
METTOv1_970006	protein of unknown function	82	91
METTOv1_10139	protein of unknown function	81	80
METTOv1_30015	conserved protein of unknown function	81	121
METTOv1_30042	conserved protein of unknown function	81	90
METTOv1_150066	membrane protein of unknown function	81	71
METTOv1_240016	conserved exported protein of unknown function	81	98
METTOv1_300054	tRNA synthetase class II (G H P and S)	81	71
METTOv1_310005	protein of unknown function	81	65
METTOv1_310051	ErfK/YbiS/YcfS/YnhG family protein	81	81
METTOv1_340020	conserved protein of unknown function	81	70
METTOv1_380015	conserved membrane protein of unknown function	81	73
METTOv1_380026	Tetratricopeptide TPR_2 repeat protein (modular protein)	81	73
METTOv1_420021	ABC transporter related	81	77
METTOv1_470012	conserved protein of unknown function	81	115
METTOv1_710005	conserved protein of unknown function	81	66
METTOv1_730014	protein of unknown function	81	79

METTOv1_860008	glycosyl transferase group 1	81	86
METTOv1_970014	exported protein of unknown function	81	58
METTOv1_1040007	Integrase family protein	81	64
METTOv1_1290002	Ferritin Dps family protein	81	74
METTOv1_10202	adenylosuccinate lyase	80	95
METTOv1_20139	ribosome biogenesis GTP-binding protein YsxC	80	82
METTOv1_20177	protein of unknown function	80	91
METTOv1_30058	40-residue YVTN family beta-propeller repeat protein	80	65
METTOv1_100078	Cytochrome c-552	80	102
METTOv1_110010	protein of unknown function UPF0153	80	70
METTOv1_140003	Quaternary ammonium compound-resistance protein qacE	80	75
METTOv1_140041	protein of unknown function	80	71
METTOv1_180002	conserved protein of unknown function	80	73
METTOv1_180072	conserved exported protein of unknown function	80	68
METTOv1_220008	protein of unknown function	80	54
METTOv1_350004	glutamyl-tRNA synthetase	80	65
METTOv1_410018	putative Histidine kinase	80	81
METTOv1_440040	nicotinate-nucleotide pyrophosphorylase	80	61
METTOv1_570001	protein of unknown function	80	90
METTOv1_670026	conserved protein of unknown function	80	84
METTOv1_810013	conserved membrane protein of unknown function	80	85
METTOv1_930001	protein of unknown function	80	62
METTOv1_950002	protein of unknown function	80	94
METTOv1_1190004	putative Helicase, Snf2 family	80	81
METTOv1_30083	protein of unknown function	79	64
METTOv1_30153	exported protein of unknown function	79	79
METTOv1_40030	Lytic transglycosylase catalytic	79	71
METTOv1_90046	membrane protein of unknown function	79	65
METTOv1_120010	protein of unknown function	79	68
METTOv1_120030	protein of unknown function	79	86
METTOv1_180004	protein of unknown function	79	52
METTOv1_240061	protein of unknown function	79	53
METTOv1_310052	putative RhsD protein	79	87
METTOv1_420022	Leucyl aminopeptidase	79	84
METTOv1_440047	dihydropteroate synthase	79	80
METTOv1_540015	Cytochrome d ubiquinol oxidase subunit 2 (Cytochrome d ubiquinol oxidase subunit II) (Cytochrome bd-I oxidase subunit II)	79	51
METTOv1_560021	conserved hypothetical rhodanese sulfurtransferase protein	79	76
METTOv1_620012	protein of unknown function	79	101
METTOv1_650011	ErfK/YbiS/YcfS/YnhG family protein	79	88
METTOv1_730012	conserved exported protein of unknown function	79	71
METTOv1_730013	putative C-N oxidoreductase	79	75
METTOv1_1040001	transposase (fragment)	79	93
METTOv1_1080004	indolepyruvate ferredoxin oxidoreductase	79	77
METTOv1_1170003	putative RNA polymerase sigma-E factor	79	94
METTOv1_10047	conserved exported protein of unknown function	78	90
METTOv1_40077	Transcriptional regulator, TetR family	78	93
METTOv1_50035	GTPase obg	78	74
METTOv1_60139	penicillin-binding protein, 1A family	78	68
METTOv1_80108	alanine racemase domain protein	78	71
METTOv1_130025	TonB family protein	78	67
METTOv1_160064	extracellular solute-binding protein family 1	78	76

METTOv1_190026	glucan biosynthesis protein G	78	67
METTOv1_230019	protein of unknown function	78	74
METTOv1_300036	protein of unknown function DUF459	78	64
METTOv1_360033	conserved protein of unknown function	78	61
METTOv1_370043	protein of unknown function	78	99
METTOv1_380005	FAD linked oxidase domain protein	78	68
METTOv1_420007	protein of unknown function DUF1491	78	85
METTOv1_1070005	conserved membrane protein of unknown function	78	70
METTOv1_10012	malonyl CoA-acyl carrier protein transacylase	77	67
METTOv1_10160	conserved protein of unknown function	77	98
METTOv1_20088	Arogenate dehydrogenase	77	70
METTOv1_60024	ABC transporter related	77	75
METTOv1_140013	protein of unknown function DUF1153	77	103
METTOv1_140064	AAA ATPase	77	81
METTOv1_200045	Rhodanese domain protein	77	64
METTOv1_230058	conserved protein of unknown function	77	70
METTOv1_260046	conserved protein of unknown function	77	82
METTOv1_290007	(2Fe-2S)-binding domain protein	77	66
METTOv1_300017	polyphosphate kinase	77	75
METTOv1_350045	protein of unknown function UPF0047	77	72
METTOv1_410038	conserved protein of unknown function	77	80
METTOv1_490027	putative hydroxamate-type ferrisiderophore receptor	77	83
METTOv1_530011	tol-pal system-associated acyl-CoA thioesterase	77	56
METTOv1_530028	exported protein of unknown function	77	88
METTOv1_600003	putative MotC protein	77	62
METTOv1_600012	protein of unknown function	77	67
METTOv1_680018	protein of unknown function	77	75
METTOv1_690012	protein of unknown function	77	81
METTOv1_840008	monooxygenase FAD-binding	77	66
METTOv1_10215	exported protein of unknown function	76	72
METTOv1_40066	protein of unknown function	76	52
METTOv1_40129	protein of unknown function	76	77
METTOv1_100005	protein of unknown function	76	72
METTOv1_110040	molybdopterin binding domain protein	76	65
METTOv1_110080	integration host factor, alpha subunit (modular protein)	76	83
METTOv1_150052	protein of unknown function	76	51
METTOv1_150053	conserved exported protein of unknown function	76	83
METTOv1_190046	ferredoxin	76	66
METTOv1_220049	transposase (fragment)	76	88
METTOv1_230074	Fatty acid desaturase	76	95
METTOv1_280055	conserved exported protein of unknown function	76	80
METTOv1_310009	exported protein of unknown function	76	56
METTOv1_330023	SpoVT/AbrB-like protein	76	55
METTOv1_340007	putative hydrolase, alpha/beta hydrolase fold family protein	76	76
METTOv1_350002	protein of unknown function	76	71
METTOv1_370023	Polyprenyl synthetase	76	52
METTOv1_420040	Electron-transferring-flavoprotein dehydrogenase	76	57
METTOv1_520029	component of potassium efflux complex with KefB	76	61
METTOv1_590020	protein of unknown function	76	80
METTOv1_600028	exported protein of unknown function	76	82
METTOv1_670024	Rubrerythrin	76	85
METTOv1_830022	putative restriction endonuclease	76	75
METTOv1_880001	exported protein of unknown function	76	80

METTOv1_30110	pseudouridine synthase, RluA family	75	58
METTOv1_30150	protein of unknown function	75	106
METTOv1_40091	Lipoprotein signal peptidase	75	74
METTOv1_50023	putative tRNA modification GTPase (trmE)	75	74
METTOv1_50049	short-chain dehydrogenase/reductase SDR	75	68
METTOv1_60027	putative signal transduction protein with CBS domains	75	84
METTOv1_120001	protein of unknown function	75	78
METTOv1_140010	conserved protein of unknown function	75	56
METTOv1_140063	conserved protein of unknown function	75	95
METTOv1_150055	N-formylglutamate amidohydrolase	75	79
METTOv1_250029	conserved protein of unknown function; putative deoxyribonuclease	75	57
METTOv1_300038	putative Ribonuclease T2 family protein	75	67
METTOv1_350022	conserved membrane protein of unknown function	75	68
METTOv1_390006	acriflavin resistance protein	75	70
METTOv1_530009	protein of unknown function	75	102
METTOv1_570009	protein of unknown function	75	66
METTOv1_680021	tRNA nucleotidyltransferase/poly(A) polymerase family protein	75	65
METTOv1_840004	two component transcriptional regulator, LuxR family	75	85
METTOv1_910005	Resolvase	75	81
METTOv1_20113	nitrogenase molybdenum-iron protein alpha chain	74	77
METTOv1_70032	Nicotinate-nucleotide--dimethylbenzimidazole phosphoribosyltransferase	74	62
METTOv1_90049	nicotinate (nicotinamide) nucleotide adenylyltransferase	74	105
METTOv1_90087	lipoate-protein ligase B	74	64
METTOv1_110032	exported protein of unknown function	74	61
METTOv1_160025	conserved protein of unknown function	74	75
METTOv1_190030	conserved protein of unknown function	74	58
METTOv1_260037	protein of unknown function	74	62
METTOv1_260049	protein of unknown function	74	95
METTOv1_300005	ABC transporter related	74	64
METTOv1_300043	conserved protein of unknown function	74	65
METTOv1_320003	HNH endonuclease (modular protein)	74	65
METTOv1_390035	two component, sigma54 specific, transcriptional regulator, Fis family	74	62
METTOv1_520019	Ppx/GppA phosphatase	74	81
METTOv1_640002	methyltransferase type 11	74	65
METTOv1_670003	Sensor protein	74	66
METTOv1_720010	S-adenosyl-L-methionine-dependent methyltransferase mraW	74	65
METTOv1_770014	conserved membrane protein of unknown function	74	64
METTOv1_830001	FolC bifunctional protein	74	62
METTOv1_830004	plasmid maintenance system killer	74	66
METTOv1_830023	Putative chromosome segregation SMC protein (fragment)	74	129
METTOv1_840002	protein of unknown function	74	121
METTOv1_10162	protein of unknown function	73	115
METTOv1_20106	ferredoxin	73	78
METTOv1_20143	Methyltransferase type 12 (fragment)	73	65
METTOv1_40012	triphosphoribosyl-dephospho-CoA protein	73	72
METTOv1_50015	FxsA cytoplasmic membrane protein	73	36
METTOv1_50036	GCN5-related N-acetyltransferase	73	75
METTOv1_50058	conserved protein of unknown function; putative Hpt domain	73	34
METTOv1_60118	MCP methyltransferase, CheR-type	73	62
METTOv1_130001	exported protein of unknown function	73	61

METTOv1_140026	queuine tRNA-ribosyltransferase	73	62
METTOv1_160047	Mammalian cell entry related domain protein	73	65
METTOv1_170012	conserved exported protein of unknown function	73	60
METTOv1_190077	HicA protein	73	56
METTOv1_190078	HicB protein	73	82
METTOv1_210016	putrescine transport protein (ABC superfamily, atp_bind)	73	71
METTOv1_210026	DNA mismatch endonuclease Vsr	73	82
METTOv1_290002	protein of unknown function	73	62
METTOv1_300016	chromosomal replication initiator DnaA	73	60
METTOv1_370035	conserved protein of unknown function	73	92
METTOv1_370037	putative ABC transporter, periplasmic amino acid-binding protein	73	88
METTOv1_380036	conserved protein of unknown function; putative Rieske [2Fe-2S] domain	73	106
METTOv1_390024	conserved protein of unknown function	73	66
METTOv1_410039	Lytic transglycosylase catalytic	73	44
METTOv1_520006	conserved protein of unknown function	73	80
METTOv1_540016	Cyd operon protein YbgT	73	106
METTOv1_650024	Peptidyl-dipeptidase Dcp	73	64
METTOv1_10180	Phosphoglycerate mutase (modular protein)	72	65
METTOv1_20074	conserved protein of unknown function	72	98
METTOv1_60010	molybdenum cofactor synthesis domain protein	72	50
METTOv1_70055	conserved exported protein of unknown function	72	58
METTOv1_110005	conserved protein of unknown function	72	95
METTOv1_110038	conserved protein of unknown function; putative radical SAM domain	72	58
METTOv1_130034	protein of unknown function	72	125
METTOv1_140072	conserved exported protein of unknown function	72	85
METTOv1_150067	protein of unknown function	72	80
METTOv1_230042	conserved protein of unknown function	72	83
METTOv1_290012	protein of unknown function	72	80
METTOv1_300056	membrane protein of unknown function	72	60
METTOv1_310013	protein of unknown function	72	100
METTOv1_340009	beta-lactamase domain protein	72	58
METTOv1_430006	putative Peptidase C14 caspase catalytic subunit p20	72	77
METTOv1_490028	FecR protein (fragment)	72	55
METTOv1_640010	putative Capsule polysaccharide export protein-like protein	72	65
METTOv1_910013	protein of unknown function	72	77
METTOv1_30039	putative Tetratricopeptide TPR_2	71	71
METTOv1_70029	L-threonine-O-3-phosphate decarboxylase	71	78
METTOv1_80072	conserved exported protein of unknown function	71	62
METTOv1_80105	cyclic nucleotide-binding protein	71	55
METTOv1_120004	protein of unknown function	71	82
METTOv1_130005	Addiction module antidote protein	71	61
METTOv1_130066	protein of unknown function DUF721	71	64
METTOv1_140032	conserved protein of unknown function	71	66
METTOv1_140069	protein of unknown function	71	65
METTOv1_160037	exported protein of unknown function	71	80
METTOv1_170074	protein of unknown function	71	68
METTOv1_180042	exported protein of unknown function	71	66
METTOv1_190049	PilT protein domain protein (fragment)	71	65
METTOv1_210070	Peptidase M50	71	67
METTOv1_270016	GumH protein	71	70

METTOv1_280054	putative acyl-CoA dehydrogenase	71	68
METTOv1_350042	protein of unknown function	71	92
METTOv1_380034	exported protein of unknown function	71	64
METTOv1_520010	Signal transduction histidine kinase (fragment)	71	64
METTOv1_730016	protein of unknown function	71	67
METTOv1_810012	protein of unknown function	71	73
METTOv1_830012	protein of unknown function	71	57
METTOv1_930006	protein of unknown function	71	58
METTOv1_10163	protein of unknown function	70	109
METTOv1_20014	protein of unknown function	70	40
METTOv1_30056	exported protein of unknown function	70	70
METTOv1_70024	Prophage CP4-57 regulatory (modular protein)	70	92
METTOv1_70050	conserved exported protein of unknown function	70	60
METTOv1_90061	TRAP transporter solute receptor, TAXI family	70	72
METTOv1_100068	DNA repair protein RecN	70	74
METTOv1_130014	permease YjgP/YjgQ family protein	70	81
METTOv1_130060	exported protein of unknown function	70	71
METTOv1_140025	DNA modification methylase	70	68
METTOv1_220067	phosphate ABC transporter, inner membrane subunit PstC	70	68
METTOv1_400024	Prolipoprotein diacylglyceryl transferase	70	69
METTOv1_430024	Sensor protein fixL (modular protein)	70	66
METTOv1_610006	protein of unknown function	70	86
METTOv1_670013	KDP operon transcriptional regulatory protein kdpE	70	76
METTOv1_1020003	Putative metallophosphoesterase ykuE	70	67
METTOv1_10182	putative Cobalt-zinc-cadmium resistance protein czcC	69	87
METTOv1_20119	nitrogen fixation protein	69	90
METTOv1_20132	FAD dependent oxidoreductase	69	81
METTOv1_50007	HAD-superfamily hydrolase, subfamily IIA	69	72
METTOv1_70058	conserved exported protein of unknown function	69	70
METTOv1_130048	cyclic nucleotide-binding protein	69	61
METTOv1_200007	protein of unknown function	69	76
METTOv1_230012	putative integrase protein	69	70
METTOv1_270009	Endoglucanase (fragment)	69	62
METTOv1_310014	hydrogenlyase component	69	58
METTOv1_430029	RNA polymerase sigma factor	69	48
METTOv1_450016	protein of unknown function	69	53
METTOv1_510005	integrase family protein	69	61
METTOv1_670004	protein of unknown function	69	78
METTOv1_690019	FecR protein	69	51
METTOv1_840010	Ste24 endopeptidase	69	69
METTOv1_50089	protein of unknown function	68	94
METTOv1_100037	protein of unknown function DUF815	68	76
METTOv1_110049	putative DegT/DnrJ/EryC1/StrS aminotransferase	68	67
METTOv1_110062	Oligopeptidase B	68	59
METTOv1_150033	DNA ligase	68	63
METTOv1_150069	protein of unknown function DUF125 transmembrane	68	68
METTOv1_180028	Acyl-[acyl-carrier-protein]--UDP-N-acetylglucosa mine O-acyltransferase	68	67
METTOv1_200031	fructose 1,6-bisphosphatase II	68	56
METTOv1_200052	protein of unknown function	68	54
METTOv1_210020	folate-binding protein YgfZ	68	61
METTOv1_220041	conserved protein of unknown function	68	100
METTOv1_220046	transcriptional regulator, XRE family	68	68

METTOv1_390028	2C-methyl-D-erythritol 2,4-cyclodiphosphate synthase	68	48
METTOv1_470013	conserved protein of unknown function	68	86
METTOv1_490019	FecR protein	68	54
METTOv1_590014	cation diffusion facilitator family transporter	68	71
METTOv1_800014	TonB-dependent receptor	68	61
METTOv1_890010	two-component response regulator	68	49
METTOv1_980002	conserved protein of unknown function	68	74
METTOv1_1120007	UDP-N-acetylenolpyruvoylglucosamine reductase	68	55
METTOv1_1240003	conserved protein of unknown function	68	102
METTOv1_1470002	conserved protein of unknown function	68	67
METTOv1_20159	protein of unknown function	67	63
METTOv1_20185	protein of unknown function	67	81
METTOv1_130061	exported protein of unknown function	67	69
METTOv1_140002	GTP-binding proten HflX	67	75
METTOv1_140068	single-stranded-DNA-specific exonuclease RecJ	67	61
METTOv1_150070	protein of unknown function	67	41
METTOv1_180069	conserved exported protein of unknown function	67	56
METTOv1_230018	conserved protein of unknown function	67	67
METTOv1_390010	Hopanoid biosynthesis associated RND transporter like protein HpnN	67	58
METTOv1_400039	Usg family protein	67	76
METTOv1_460026	Sensor protein	67	67
METTOv1_530014	TolA protein	67	62
METTOv1_690006	Sensor protein (modular protein)	67	72
METTOv1_730018	Type I restriction-modification system, M subunit	67	64
METTOv1_1130005	conserved exported protein of unknown function	67	61
METTOv1_1520001	Putative replication protein C (fragment)	67	48
METTOv1_10042	conserved protein of unknown function	66	56
METTOv1_20040	putative AsmA family protein	66	59
METTOv1_20045	NAD(+) kinase	66	69
METTOv1_30128	beta-lactamase domain protein	66	55
METTOv1_50013	MltA domain protein	66	68
METTOv1_50025	methyltransferase GidB	66	70
METTOv1_60026	Patatin	66	61
METTOv1_60036	N-acetyl-gamma-glutamyl-phosphate reductase	66	55
METTOv1_70067	conserved protein of unknown function; putative membrane protein	66	66
METTOv1_100035	protein of unknown function	66	69
METTOv1_150062	molybdopterin-guanine dinucleotide biosynthesis protein A	66	62
METTOv1_180005	Protein-tyrosine phosphatase, low molecular weight	66	64
METTOv1_300030	two component transcriptional regulator, winged helix family	66	63
METTOv1_350028	3'(2'),5'-bisphosphate nucleotidase	66	64
METTOv1_370044	conserved protein of unknown function	66	54
METTOv1_380027	Putative NADH dehydrogenase/NAD(P)H nitroreductase RutE (Pyrimidine utilization protein E)	66	62
METTOv1_390011	conserved protein of unknown function	66	58
METTOv1_510004	Alpha/beta hydrolase fold	66	59
METTOv1_550018	GCN5-related N-acetyltransferase	66	69
METTOv1_610021	exported protein of unknown function	66	103
METTOv1_1100007	conserved protein of unknown function	66	56
METTOv1_10131	protein of unknown function	65	77
METTOv1_60077	protein of unknown function	65	49
METTOv1_70057	protein of unknown function	65	63

METTOv1_110009	penicillin-binding protein, 1A family	65	65
METTOv1_160030	protein of unknown function	65	61
METTOv1_160036	amine oxidase	65	63
METTOv1_180050	putative Prepilin peptidase	65	59
METTOv1_180076	conserved protein of unknown function	65	85
METTOv1_210019	dihydroorotase	65	47
METTOv1_220019	protein of unknown function	65	58
METTOv1_260021	putative transcriptional regulator, XRE family	65	75
METTOv1_300049	exported protein of unknown function	65	61
METTOv1_300055	conserved exported protein of unknown function	65	43
METTOv1_370039	ABC transporter related	65	58
METTOv1_380007	protein of unknown function DUF224 cysteine-rich region domain protein	65	65
METTOv1_380048	3-oxoacyl-[acyl-carrier-protein] reductase	65	63
METTOv1_410044	protein of unknown function	65	48
METTOv1_440043	glycosyl transferase family 2	65	62
METTOv1_520026	GSCFA	65	81
METTOv1_730015	putative RNA polymerase sigma factor fecI	65	67
METTOv1_830016	pentapeptide repeat protein	65	56
METTOv1_10059	conserved protein of unknown function	64	76
METTOv1_10070	conserved exported protein of unknown function	64	59
METTOv1_10090	conserved protein of unknown function	64	43
METTOv1_40003	3-methyl-2-oxobutanoate hydroxymethyltransferase	64	63
METTOv1_50024	tRNA uridine 5-carboxymethylaminomethyl modification enzyme mnmG	64	71
METTOv1_50075	Electron transport protein SCO1/SenC (modular protein)	64	64
METTOv1_70094	dihydroorotase, multifunctional complex type	64	66
METTOv1_100055	Periplasmic binding protein	64	79
METTOv1_170013	protein of unknown function	64	58
METTOv1_180070	protein of unknown function	64	43
METTOv1_340052	conserved membrane protein of unknown function	64	32
METTOv1_360048	Cell division protein ftsH homolog (ATP-dependent zinc-metallo protease)	64	63
METTOv1_410029	flagellar biosynthesis protein FlhB	64	61
METTOv1_410040	protein of unknown function	64	65
METTOv1_520004	protein of unknown function	64	57
METTOv1_550028	conserved protein of unknown function	64	60
METTOv1_590022	4-hydroxythreonine-4-phosphate dehydrogenase	64	51
METTOv1_610018	Glyoxalase/bleomycin resistance protein/dioxygenase	64	64
METTOv1_980004	protein of unknown function	64	62
METTOv1_40089	WD-40 repeat protein	63	62
METTOv1_50087	ErfK/YbiS/YcfS/YnhG family protein	63	56
METTOv1_60047	putative Amine oxidase	63	69
METTOv1_90028	conserved protein of unknown function; Putative ATPase	63	59
METTOv1_130067	exported protein of unknown function	63	69
METTOv1_160024	conserved protein of unknown function	63	49
METTOv1_180075	serine acetyltransferase	63	65
METTOv1_220066	phosphate ABC transporter, inner membrane subunit PstA	63	63
METTOv1_240049	thiamine-phosphate pyrophosphorylase	63	65
METTOv1_260023	protein of unknown function	63	75
METTOv1_280066	conserved protein of unknown function	63	72
METTOv1_300042	conserved exported protein of unknown function	63	56
METTOv1_350007	Thioredoxin domain protein	63	72

METTOv1_400035	fragment of protein of unknown function (part 2)	63	31
METTOv1_590008	exported protein of unknown function	63	60
METTOv1_650005	conserved protein of unknown function	63	51
METTOv1_770012	conserved membrane protein of unknown function	63	70
METTOv1_820012	fragment of integral membrane sensor signal transduction histidine kinase (part 2)	63	55
METTOv1_860010	permease YjgP/YjgQ family protein	63	52
METTOv1_1170002	putative Protein fecR	63	68
METTOv1_30009	protein of unknown function	62	68
METTOv1_30111	ATP12 ATPase	62	45
METTOv1_40097	putative NAD(P) transhydrogenase subunit alpha part 2 transmembrane protein	62	60
METTOv1_80068	diaminopimelate decarboxylase	62	58
METTOv1_100036	conserved protein of unknown function	62	53
METTOv1_100065	protein of unknown function	62	52
METTOv1_180034	phosphatidate cytidylyltransferase	62	60
METTOv1_190057	Peptidoglycan-binding domain 1 protein	62	60
METTOv1_240018	conserved protein of unknown function; putative signal peptide	62	79
METTOv1_250035	UPF0102 protein Msil_0293	62	56
METTOv1_280040	protein of unknown function	62	57
METTOv1_320049	protein of unknown function	62	53
METTOv1_350020	Polysaccharide deacetylase	62	62
METTOv1_350030	pantoate--beta-alanine ligase	62	63
METTOv1_420005	conserved protein of unknown function with SCP/PR1 domains; putative signal peptide	62	54
METTOv1_600002	protein of unknown function	62	60
METTOv1_610024	Serine O-acetyltransferase	62	39
METTOv1_790004	Polysaccharide export protein (modular protein)	62	64
METTOv1_910014	membrane protein of unknown function	62	84
METTOv1_980003	AAA ATPase, central region	62	63
METTOv1_1140002	conserved protein of unknown function	62	60
METTOv1_1500001	putative C-N oxidoreductase (fragment)	62	55
METTOv1_10132	protein of unknown function	61	57
METTOv1_10177	conserved protein of unknown function	61	62
METTOv1_20002	protein of unknown function DUF900 hydrolase family protein	61	52
METTOv1_20054	glutamyl-tRNA synthetase	61	46
METTOv1_20063	Spore coat polysaccharide biosynthesis protein F CMP-KDO synthetase-like protein	61	43
METTOv1_20129	nitrogen fixation protein NifW	61	50
METTOv1_20136	protein of unknown function	61	65
METTOv1_30040	conserved exported protein of unknown function	61	65
METTOv1_90069	protein of unknown function	61	59
METTOv1_90072	dihydropteroate synthase DHPS	61	57
METTOv1_130024	conserved exported protein of unknown function	61	46
METTOv1_160045	protein of unknown function DUF140	61	66
METTOv1_200003	protein of unknown function DUF1355	61	65
METTOv1_240023	extracellular solute-binding protein family 3	61	56
METTOv1_240056	HAD-superfamily hydrolase, subfamily IA, variant 1	61	61
METTOv1_330004	Urease accessory protein ureG	61	46
METTOv1_380003	protein of unknown function	61	53
METTOv1_440029	type I secretion outer membrane protein, TolC family	61	47
METTOv1_490021	TonB-dependent siderophore receptor	61	43
METTOv1_500009	transporter, hydrophobe/amphiphile efflux-1 (HAE1) family	61	61

METTOv1_540022	Probable O-sialoglycoprotein endopeptidase	61	48
METTOv1_980012	protein of unknown function	61	64
METTOv1_10185	two component transcriptional regulator, winged helix family	60	50
METTOv1_70007	conserved protein of unknown function	60	48
METTOv1_70033	exported protein of unknown function	60	47
METTOv1_80010	conserved protein of unknown function	60	61
METTOv1_80022	conserved protein of unknown function	60	65
METTOv1_120059	transcriptional regulator of oxidative stress, regulates intracellular hydrogen peroxide (LysR family)	60	68
METTOv1_140038	conserved protein of unknown function	60	78
METTOv1_140055	protein of unknown function	60	54
METTOv1_150036	diguanylate cyclase/phosphodiesterase	60	56
METTOv1_150075	ABC transporter related	60	61
METTOv1_160067	integral membrane sensor signal transduction histidine kinase	60	64
METTOv1_170087	protein of unknown function	60	69
METTOv1_180046	putative General secretion pathway protein F	60	56
METTOv1_220003	protein of unknown function DUF218	60	58
METTOv1_230047	UvrD/REP helicase	60	57
METTOv1_240052	ribose 5-phosphate isomerase	60	42
METTOv1_250038	conserved protein of unknown function	60	56
METTOv1_280029	putative chaperone protein (yegD)	60	55
METTOv1_330022	PilT protein domain protein	60	46
METTOv1_370003	lytic murein transglycosylase	60	57
METTOv1_370041	putative alpha-isopropylmalate/homocitrate synthase family transferase	60	49
METTOv1_380030	rare lipoprotein A	60	65
METTOv1_440042	YdjC family protein	60	60
METTOv1_440048	2-amino-4-hydroxy-6- hydroxymethyldihydropteridine pyrophosphokinase	60	59
METTOv1_720002	UDP-N-acetylglucosamine--N-acetylmuramyl- (pentapeptide) pyrophosphoryl-undecaprenol N-acetylglucosamine transferase	60	53
METTOv1_790005	Protein-tyrosine-phosphatase (fragment)	60	68
METTOv1_860007	glycosyl transferase group 1	60	58
METTOv1_10083	membrane protein of unknown function	59	43
METTOv1_20081	Prevent-host-death family protein	59	45
METTOv1_30103	ferredoxin-dependent glutamate synthase	59	59
METTOv1_40009	conserved protein of unknown function	59	81
METTOv1_50032	Putative major facilitator family transporter	59	64
METTOv1_60043	conserved exported protein of unknown function	59	51
METTOv1_70070	conserved protein of unknown function	59	51
METTOv1_110007	conserved protein of unknown function	59	62
METTOv1_140023	peptidase M15D vanX D-ala-D-ala dipeptidase	59	46
METTOv1_160057	Mannosidase	59	46
METTOv1_180016	ATP synthase C chain (Lipid-binding protein)	59	86
METTOv1_190075	protein of unknown function	59	39
METTOv1_210009	2-haloacid dehalogenase	59	63
METTOv1_220054	CMP/dCMP deaminase zinc-binding	59	65
METTOv1_300053	ATP phosphoribosyltransferase	59	55
METTOv1_330001	Putative replication protein C (fragment)	59	46
METTOv1_330010	Urease subunit gamma	59	60
METTOv1_350043	protein of unknown function	59	122
METTOv1_390016	conserved protein of unknown function; putative molybdenum ABC transporter (ATP-binding protein) (fragment)	59	71

METTOv1_430019	exsB protein	59	46
METTOv1_440039	L-aspartate oxidase	59	56
METTOv1_450019	exported protein of unknown function	59	49
METTOv1_560005	protein of unknown function	59	38
METTOv1_750004	protein of unknown function	59	70
METTOv1_800013	conserved protein of unknown function; putative prolyl 4-hydroxylase, iron-regulated	59	65
METTOv1_1320003	riboflavin biosynthesis protein RibD	59	51
METTOv1_1390001	putative C-N oxidoreductase (fragment)	59	51
METTOv1_20001	exported protein of unknown function	58	42
METTOv1_20094	transcriptional regulator, NifA, Fis Family	58	55
METTOv1_90058	Chemoreceptor mcpA	58	58
METTOv1_130072	DNA primase	58	59
METTOv1_160033	Helix-hairpin-helix motif protein	58	51
METTOv1_180073	putative sulfurtransferase (Rhodanese)	58	55
METTOv1_210046	conserved protein of unknown function	58	53
METTOv1_260053	protein of unknown function	58	57
METTOv1_300001	Peptidase M23 (fragment)	58	55
METTOv1_300025	conserved protein of unknown function	58	53
METTOv1_340023	Methyltransferase type 12	58	44
METTOv1_390026	CinA domain protein	58	54
METTOv1_400009	ABC transporter related	58	45
METTOv1_470003	Peroxidase protein	58	69
METTOv1_640018	surface antigen (D15)	58	60
METTOv1_710015	Polyketide biosynthesis protein pksE [Includes: Malonyl CoA-acyl carrier protein transacylase] (fragment)	58	49
METTOv1_860003	conserved membrane protein of unknown function	58	43
METTOv1_10138	conserved protein of unknown function; putative Rieske [2Fe-2S] domain.	57	54
METTOv1_10220	GDP-6-deoxy-D-lyxo-4-hexulose reductase	57	57
METTOv1_20048	membrane protein of unknown function	57	52
METTOv1_30008	protein of unknown function	57	55
METTOv1_40024	(p)ppGpp synthetase I, SpoT/RelA	57	63
METTOv1_50002	exported protein of unknown function	57	64
METTOv1_60126	Diguanylate cyclase with PAS/PAC sensor	57	56
METTOv1_80106	conserved protein of unknown function	57	60
METTOv1_100034	putative Histidine kinase	57	40
METTOv1_120014	precorrin-6x reductase	57	62
METTOv1_170007	protein of unknown function	57	65
METTOv1_170009	protein of unknown function	57	73
METTOv1_210043	6-phosphogluconate dehydrogenase NAD-binding	57	54
METTOv1_210068	conserved membrane protein of unknown function	57	37
METTOv1_250054	peptide chain release factor 3	57	55
METTOv1_280019	conserved protein of unknown function	57	67
METTOv1_340048	nucleotide-binding protein-like protein	57	49
METTOv1_360028	conserved exported protein of unknown function	57	67
METTOv1_390022	Lipoyl synthase	57	61
METTOv1_390029	TIM-barrel protein, nifR3 family	57	53
METTOv1_650025	exported protein of unknown function	57	67
METTOv1_700023	protein of unknown function	57	69
METTOv1_780014	protein of unknown function	57	51
METTOv1_960002	putative UDP-N-acetylglucosamine 1-carboxyvinyltransferase	57	61
METTOv1_970005	cystathionine beta-lyase	57	49

METTOv1_1050001	Putative replication protein C (fragment)	57	57
METTOv1_1190005	protein of unknown function	57	61
METTOv1_1320004	riboflavin synthase, alpha subunit	57	49
METTOv1_1370003	putative C-N oxidoreductase (fragment)	57	42
METTOv1_10127	Sodium/hydrogen exchanger	56	53
METTOv1_20031	Ribonuclease H	56	47
METTOv1_20099	tetratricopeptide TPR_2	56	58
METTOv1_60023	ribosomal RNA methyltransferase RrmJ/FtsJ	56	47
METTOv1_60138	protein of unknown function	56	33
METTOv1_120012	precorrin-2 C20-methyltransferase	56	65
METTOv1_120060	catalase/peroxidase HPI	56	56
METTOv1_130042	Cytochrome c biogenesis protein transmembrane region	56	47
METTOv1_140028	S-adenosylmethionine:tRNA ribosyltransferase-isomerase	56	36
METTOv1_190063	exported protein of unknown function	56	58
METTOv1_200026	GMP synthase, large subunit	56	55
METTOv1_230035	protein of unknown function	56	53
METTOv1_240062	protein of unknown function	56	72
METTOv1_260020	protein of unknown function	56	85
METTOv1_260047	conserved protein of unknown function	56	53
METTOv1_260054	protein of unknown function	56	61
METTOv1_280017	fragment of Erythromycin esterase (part 2)	56	57
METTOv1_300015	protein of unknown function UPF0118	56	56
METTOv1_330021	protein of unknown function	56	55
METTOv1_340012	protein of unknown function	56	112
METTOv1_360005	RNA methyltransferase, TrmH family, group 3	56	64
METTOv1_360018	5,10-methylenetetrahydrofolate reductase	56	49
METTOv1_780003	Nitrogen fixation regulation protein fixK	56	62
METTOv1_790003	Capsular exopolysaccharide family	56	54
METTOv1_1090004	chromosome segregation and condensation protein, ScpB	56	58
METTOv1_1140003	protein of unknown function	56	60
METTOv1_1240004	protein of unknown function	56	67
METTOv1_10212	Luciferase-like monooxygenase	55	60
METTOv1_20033	UDP-N-acetylglucosamine pyrophosphorylase	55	43
METTOv1_20178	conserved protein of unknown function	55	35
METTOv1_40116	Glycoside hydrolase 15-related	55	57
METTOv1_50051	protein of unknown function DUF404	55	55
METTOv1_50094	conserved protein of unknown function	55	36
METTOv1_90068	protein of unknown function	55	45
METTOv1_110083	protein of unknown function DUF177	55	59
METTOv1_120003	protein of unknown function	55	86
METTOv1_130028	putative CHAD domain containing protein	55	50
METTOv1_130040	protein of unknown function	55	50
METTOv1_140019	membrane protein of unknown function	55	58
METTOv1_150045	exported protein of unknown function	55	43
METTOv1_150073	GCN5-related N-acetyltransferase	55	56
METTOv1_180049	exported protein of unknown function	55	47
METTOv1_200024	HPr kinase	55	54
METTOv1_200063	Glycosyl transferase group 1	55	62
METTOv1_220063	two component transcriptional regulator PhoB, winged helix family	55	63
METTOv1_220071	integral membrane protein-like protein	55	57
METTOv1_250015	conserved protein of unknown function	55	51
METTOv1_250048	dihydrolipoamide dehydrogenase	55	54

METTOv1_280006	protein of unknown function	55	76
METTOv1_420036	putative methylated-DNA--protein-cysteine methyltransferase (fragment)	55	52
METTOv1_440024	putative Metallophosphoesterase	55	60
METTOv1_480034	conserved protein of unknown function	55	37
METTOv1_520007	protein of unknown function	55	60
METTOv1_540020	NAD(P)H-dependent glycerol-3-phosphate dehydrogenase	55	44
METTOv1_590012	efflux transporter, RND family, MFP subunit	55	43
METTOv1_600001	protein of unknown function	55	47
METTOv1_680022	conserved protein of unknown function	55	55
METTOv1_710012	putative ABC-1	55	54
METTOv1_810006	conserved exported protein of unknown function	55	51
METTOv1_1000007	conserved protein of unknown function	55	66
METTOv1_1100008	transposase (fragment)	55	80
METTOv1_1460001	conserved protein of unknown function	55	107
METTOv1_10144	Short-chain dehydrogenase/reductase SDR	54	62
METTOv1_50055	riboflavin biosynthesis protein RibF	54	67
METTOv1_60076	Alkyl hydroperoxide reductase subunit F (Alkyl hydroperoxide reductase F52A protein)	54	54
METTOv1_100002	3,4-dihydroxy-2-butanone 4-phosphate synthase	54	50
METTOv1_150029	lipid A ABC exporter family, fused ATPase and inner membrane subunits	54	38
METTOv1_150054	histidinol-phosphate phosphatase	54	49
METTOv1_250045	Alpha/beta hydrolase	54	56
METTOv1_290033	conserved protein of unknown function	54	64
METTOv1_390005	acriflavin resistance protein	54	49
METTOv1_430016	conserved protein of unknown function	54	43
METTOv1_480002	conserved protein of unknown function	54	55
METTOv1_480003	metallophosphoesterase	54	51
METTOv1_520017	sulfate ABC transporter, periplasmic sulfate-binding protein	54	41
METTOv1_530025	protein of unknown function	54	42
METTOv1_620011	Thymidylate synthase thyX (fragment)	54	49
METTOv1_680020	protein of unknown function	54	57
METTOv1_780013	Enoyl-CoA hydratase/isomerase	54	46
METTOv1_810003	putative TonB-dependent receptor	54	57
METTOv1_830021	protein of unknown function	54	90
METTOv1_1240002	protein of unknown function	54	42
METTOv1_1520002	putative DNA-invertase y4cG (fragment)	54	51
METTOv1_10046	ribonuclease H	53	38
METTOv1_10200	DNA-binding response regulator	53	46
METTOv1_20079	TIM-barrel protein, yjbN family	53	48
METTOv1_20130	Electron transfer flavoprotein alpha/beta-subunit	53	55
METTOv1_30050	protein of unknown function	53	44
METTOv1_30139	conserved protein of unknown function	53	51
METTOv1_90080	pseudouridine synthase, RluA family	53	45
METTOv1_110073	conserved protein of unknown function	53	69
METTOv1_120013	precorrin-3B C17-methyltransferase	53	52
METTOv1_190064	conserved membrane protein of unknown function	53	41
METTOv1_220026	conserved protein of unknown function	53	34
METTOv1_240050	glutathione-disulfide reductase	53	47
METTOv1_330008	Urease subunit alpha	53	59
METTOv1_600014	Aspartyl protease-like protein (fragment)	53	54
METTOv1_610016	Arsenical resistance operon trans-acting repressor arsD	53	30

METTOv1_630013	Proton/sodium-glutamate symport protein	53	48
METTOv1_670007	urea amidolyase related protein	53	57
METTOv1_670017	protein of unknown function	53	55
METTOv1_840016	ABC-type branched-chain amino acid transport systems, periplasmic component (fragment)	53	42
METTOv1_880006	exported protein of unknown function	53	51
METTOv1_980009	conserved protein of unknown function	53	76
METTOv1_1030003	conserved protein of unknown function	53	55
METTOv1_1190006	protein of unknown function	53	75
METTOv1_20043	exported protein of unknown function	52	61
METTOv1_20080	conserved protein of unknown function	52	50
METTOv1_20137	putative HAD-superfamily hydrolase; Pyrimidine 5-nucleotidase	52	54
METTOv1_40050	exported protein of unknown function	52	46
METTOv1_50073	DNA-cytosine methyltransferase	52	42
METTOv1_60079	conserved protein of unknown function	52	52
METTOv1_70052	PpiC-type peptidyl-prolyl cis-trans isomerase	52	52
METTOv1_120008	putative accessory protein to ABC-type macrolide transport protein MacB	52	45
METTOv1_130046	BNR/Asp-box repeat domain protein	52	46
METTOv1_140054	phosphoribosylglycinamide formyltransferase	52	46
METTOv1_160046	ABC transporter related	52	39
METTOv1_190066	Porphobilinogen deaminase	52	62
METTOv1_190079	Shikimate dehydrogenase	52	51
METTOv1_220013	extracellular solute-binding protein family 5	52	55
METTOv1_250039	tRNA-specific 2-thiouridylase MnmA	52	50
METTOv1_260050	protein of unknown function	52	40
METTOv1_280063	putative spermidine synthase	52	44
METTOv1_540025	conserved exported protein of unknown function	52	49
METTOv1_570025	putative transcriptional regulator, CopG family	52	36
METTOv1_630016	transcriptional regulator, LysR family	52	39
METTOv1_670005	LamB/YcsF family protein	52	47
METTOv1_680006	Putative enoyl-CoA hydratase	52	49
METTOv1_800008	putative transcriptional regulator, XRE family (modular protein)	52	48
METTOv1_900002	putative tetracycline-efflux transporter	52	46
METTOv1_970003	tRNA/rRNA methyltransferase (SpoU)	52	62
METTOv1_1290001	PII uridylyl-transferase	52	49
METTOv1_1550002	protein of unknown function	52	52
METTOv1_20020	conserved exported protein of unknown function	51	54
METTOv1_20059	protein of unknown function	51	68
METTOv1_20114	nitrogenase molybdenum-iron protein beta chain	51	53
METTOv1_20124	iron-sulfur cluster assembly accessory protein	51	64
METTOv1_30060	Methylthioribose-1-phosphate isomerase	51	52
METTOv1_40079	putative peptidase	51	46
METTOv1_60044	Holliday junction ATP-dependent DNA helicase ruvB	51	50
METTOv1_70062	ribonuclease R	51	56
METTOv1_80107	protein of unknown function	51	57
METTOv1_130058	apolipoprotein N-acyltransferase	51	44
METTOv1_220044	metallophosphoesterase	51	41
METTOv1_240066	conserved protein of unknown function	51	35
METTOv1_400041	two component transcriptional regulator, LuxR family	51	38
METTOv1_450020	protein of unknown function	51	47
METTOv1_480028	protein of unknown function	51	46
METTOv1_510020	conserved exported protein of unknown function	51	51

METTOv1_620022	Ribulose-phosphate 3-epimerase	51	53
METTOv1_640012	exported protein of unknown function	51	36
METTOv1_730004	putative sigma-70 factor, ECF subfamily	51	46
METTOv1_1210001	protein of unknown function	51	50
METTOv1_10011	conserved exported protein of unknown function	50	40
METTOv1_10197	conserved protein of unknown function	50	29
METTOv1_60012	PilT protein domain protein	50	52
METTOv1_60070	RND divalent metal cation efflux transporter CnrA	50	44
METTOv1_110067	Transcriptional regulator, TetR family protein	50	58
METTOv1_170078	protein of unknown function	50	50
METTOv1_180021	membrane-bound ATP synthase, F1 sector, beta-subunit	50	41
METTOv1_180022	conserved exported protein of unknown function	50	46
METTOv1_200004	conserved protein of unknown function; putative membrane protein	50	56
METTOv1_200049	Ferredoxin-6	50	73
METTOv1_210011	NADPH-dependent FMN reductase	50	51
METTOv1_210060	2'-deoxycytidine 5'-triphosphate deaminase	50	51
METTOv1_240064	protein of unknown function	50	67
METTOv1_250044	PRC-barrel domain protein	50	57
METTOv1_260044	conserved protein of unknown function	50	54
METTOv1_350050	pyruvate phosphate dikinase	50	52
METTOv1_530008	conserved protein of unknown function	50	34
METTOv1_600015	GtrA family protein	50	32
METTOv1_650007	putative Acyltransferase 3	50	81
METTOv1_650023	conserved protein of unknown function; putative conserved domain typically associated with flavoprotein oxygenases, DIM6/NTAB fa	50	55
METTOv1_670021	putative phosphohistidine phosphatase, SixA	50	57
METTOv1_690008	Sensor protein	50	58
METTOv1_770009	protein of unknown function	50	56
METTOv1_810007	protein of unknown function	50	54
METTOv1_880010	putative TonB-dependent receptor protein	50	45
METTOv1_920010	membrane protein of unknown function	50	75
METTOv1_930011	L-fuculose-1-phosphate aldolase	50	58
METTOv1_1170001	putative TonB-dependent receptor protein	50	55
METTOv1_1580001	conserved protein of unknown function	50	62
METTOv1_50052	transglutaminase domain protein	49	41
METTOv1_60071	Outer membrane efflux protein bepC	49	42
METTOv1_60113	methyl-accepting chemotaxis sensory transducer (fragment)	49	45
METTOv1_70083	protein of unknown function	49	55
METTOv1_80053	Alanine racemase	49	54
METTOv1_100001	chromosome segregation protein SMC (fragment)	49	51
METTOv1_180015	ATP synthase subunit b 2	49	34
METTOv1_210053	alpha/beta hydrolase fold protein	49	40
METTOv1_250006	protein of unknown function	49	59
METTOv1_280058	putative Histidine kinase	49	57
METTOv1_310028	ABC transporter related	49	38
METTOv1_330005	putative Protein hupE	49	51
METTOv1_350011	Response regulator receiver protein	49	36
METTOv1_360001	conserved protein of unknown function	49	40
METTOv1_370024	protein of unknown function DUF1345	49	45
METTOv1_400001	fumarylacetoacetate (FAA) hydrolase	49	61
METTOv1_520016	putative L-sorbosone dehydrogenase	49	49

METTOv1_590024	cytochrome c-type biogenesis protein CcmI	49	47
METTOv1_670023	protein of unknown function DUF463 YcjX family protein	49	39
METTOv1_680016	conserved protein of unknown function; putative DJ-1/PfpI family protein	49	41
METTOv1_850010	protein of unknown function	49	43
METTOv1_1410001	protein of unknown function	49	60
METTOv1_10211	protein of unknown function	48	41
METTOv1_30019	protein of unknown function	48	60
METTOv1_40002	Ferrochelatase	48	53
METTOv1_40049	Transcriptional regulator, TetR family	48	34
METTOv1_50109	Putative HPr kinase/phosphorylase	48	43
METTOv1_70051	conserved exported protein of unknown function	48	42
METTOv1_80001	exported protein of unknown function	48	50
METTOv1_80080	magnesium transporter	48	48
METTOv1_90017	protein of unknown function	48	55
METTOv1_90063	putative transcriptional regulator, Fur family; zinc uptake regulator ZUR	48	39
METTOv1_120006	Carbamoyltransferase	48	53
METTOv1_130059	exported protein of unknown function	48	55
METTOv1_240033	exported protein of unknown function	48	42
METTOv1_240048	thiazole biosynthesis family protein	48	48
METTOv1_240065	protein of unknown function	48	40
METTOv1_300039	protein of unknown function DUF519	48	50
METTOv1_350025	conserved exported protein of unknown function	48	49
METTOv1_380004	protein of unknown function DUF336	48	41
METTOv1_380006	FAD linked oxidase domain protein	48	52
METTOv1_380029	protein of unknown function	48	44
METTOv1_400002	conserved membrane protein of unknown function	48	37
METTOv1_410041	protein of unknown function	48	55
METTOv1_530016	protein of unknown function	48	47
METTOv1_630011	transcription-repair coupling factor	48	45
METTOv1_640011	protein of unknown function	48	60
METTOv1_730005	putative FecR iron sensor protein	48	58
METTOv1_810004	protein of unknown function	48	42
METTOv1_1020002	protein of unknown function	48	49
METTOv1_1400003	transposase (fragment)	48	42
METTOv1_1450001	protein of unknown function	48	53
METTOv1_10025	Tetraacyldisaccharide 4'-kinase (Lipid A 4'-kinase)	47	56
METTOv1_10034	protein of unknown function DUF558	47	48
METTOv1_10118	protein of unknown function	47	55
METTOv1_10205	molybdate ABC transporter, inner membrane subunit	47	37
METTOv1_10213	protein of unknown function	47	36
METTOv1_20184	protein of unknown function	47	44
METTOv1_50106	Homocysteine S-methyltransferase	47	42
METTOv1_70059	Lytic transglycosylase, catalytic	47	47
METTOv1_80077	Predicted Zn-dependent protease, modulator of DNA gyrase, TldD protein	47	53
METTOv1_100072	conserved protein of unknown function	47	55
METTOv1_110008	protein of unknown function DUF1214	47	48
METTOv1_150039	Regulator of chromosome condensation RCC1 (fragment)	47	43
METTOv1_160042	DNA polymerase I	47	44
METTOv1_170005	CRISPR-associated protein, Cas1 family	47	54
METTOv1_170010	protein of unknown function	47	76

METTOv1_190047	glucosamine/fructose-6-phosphate aminotransferase, isomerizing	47	42
METTOv1_210012	exodeoxyribonuclease III	47	40
METTOv1_210038	protein of unknown function	47	24
METTOv1_270060	Uncharacterized amino acid permease yfnA	47	36
METTOv1_290003	Peptidase S41	47	53
METTOv1_320051	protein of unknown function	47	50
METTOv1_330009	Urease subunit beta	47	50
METTOv1_330018	exported protein of unknown function	47	35
METTOv1_510022	conserved protein of unknown function	47	42
METTOv1_550014	Surfeit locus 1 family protein (fragment)	47	42
METTOv1_690018	putative TonB-dependent receptor	47	43
METTOv1_800022	conserved membrane protein of unknown function	47	77
METTOv1_860004	putative MscS Mechanosensitive ion channel	47	47
METTOv1_860005	lysine 2,3-aminomutase YodO family protein	47	41
METTOv1_910006	putative Resolvase, N-terminal:Recombinase (fragment)	47	42
METTOv1_1110004	conserved membrane protein of unknown function	47	61
METTOv1_10095	conserved protein of unknown function	46	48
METTOv1_20121	ferredoxin III, nif-specific	46	49
METTOv1_30108	putative Type IV pilus assembly PilZ	46	56
METTOv1_40088	Transcriptional regulator protein (fragment)	46	44
METTOv1_40115	Trehalose-6-phosphate synthase	46	44
METTOv1_50031	Glutamate-ammonia-ligase adenylyltransferase	46	40
METTOv1_50093	putative phosphoesterase familiy protein; putative signal peptide	46	49
METTOv1_80002	putative two component response transcriptional regulator (OmpR family)	46	51
METTOv1_90043	putative TonB-dependent receptor protein	46	47
METTOv1_110079	Major facilitator superfamily MFS_1	46	40
METTOv1_140024	GCN5-related N-acetyltransferase	46	53
METTOv1_160048	Transcriptional regulator, AraC family	46	39
METTOv1_180024	uracil-DNA-glycosylase	46	42
METTOv1_190069	Transcriptional regulator, MarR family	46	43
METTOv1_220043	glycosyl transferase group 1	46	48
METTOv1_280044	DNA-3-methyladenine glycosylase I	46	36
METTOv1_290022	Fatty acid desaturase	46	49
METTOv1_310041	LysR transcriptional regulator-like protein	46	30
METTOv1_310055	protein of unknown function	46	46
METTOv1_360052	protein of unknown function	46	41
METTOv1_420006	conserved protein of unknown function	46	45
METTOv1_420037	SNARE associated Golgi protein	46	32
METTOv1_440031	transcriptional regulator, BadM/Rrf2 family	46	46
METTOv1_450021	protein of unknown function	46	94
METTOv1_510013	Glyoxalase family protein	46	47
METTOv1_550027	putative Lyso-ornithine lipid acyltransferase	46	37
METTOv1_620002	sulfate ABC transporter, inner membrane subunit CysW	46	46
METTOv1_680023	conserved protein of unknown function	46	60
METTOv1_700008	putative Lipoprotein	46	54
METTOv1_710018	protein of unknown function	46	50
METTOv1_720012	glutamate racemase	46	47
METTOv1_820002	Oxidoreductase, aldo/keto reductase family	46	40
METTOv1_880007	conserved exported protein of unknown function	46	60
METTOv1_1040010	site-specific DNA recombinase; e14 prophage (modular protein)	46	41

METTOv1_1150001	protein of unknown function	46	53
METTOv1_10192	conserved protein of unknown function	45	39
METTOv1_50004	protein of unknown function	45	55
METTOv1_60046	membrane protein of unknown function	45	47
METTOv1_60090	RNA polymerase, sigma-24 subunit, ECF subfamily (modular protein)	45	43
METTOv1_80011	D-amino-acid dehydrogenase	45	45
METTOv1_80017	exported protein of unknown function	45	46
METTOv1_100018	putative Predicted glycosyltransferase	45	43
METTOv1_100081	Sensor protein (modular protein)	45	37
METTOv1_180026	OpgC protein	45	32
METTOv1_190062	integrase family protein	45	39
METTOv1_230004	protein of unknown function	45	49
METTOv1_250052	putative methylamine utilization protein MauG	45	41
METTOv1_260028	protein of unknown function	45	65
METTOv1_290006	aldehyde oxidase and xanthine dehydrogenase molybdopterin binding	45	38
METTOv1_360016	carbon starvation protein CstA	45	41
METTOv1_400026	protein of unknown function DUF152	45	50
METTOv1_410042	exported protein of unknown function	45	35
METTOv1_410043	protein of unknown function	45	31
METTOv1_420024	conserved protein of unknown function	45	51
METTOv1_450001	putative Phenylalanine racemase (ATP-hydrolyzing)	45	46
METTOv1_550022	protein of unknown function	45	50
METTOv1_650006	putative 3-oxoacyl-[acyl-carrier-protein] reductase	45	46
METTOv1_710007	putative cytochrome P450 110	45	31
METTOv1_770008	exported protein of unknown function	45	13
METTOv1_1450002	MotA/TolQ/ExbB proton channel	45	59
METTOv1_20029	exported protein of unknown function	44	43
METTOv1_30023	Plasmid stability protein, putative (fragment)	44	19
METTOv1_60017	protein of unknown function	44	73
METTOv1_110034	integral membrane sensor signal transduction histidine kinase	44	40
METTOv1_120011	Precorrin-8X methylmutase	44	45
METTOv1_150038	conserved protein of unknown function, putative regulator of chromosome condensation	44	38
METTOv1_160029	exported protein of unknown function	44	41
METTOv1_260055	putative integrase protein	44	55
METTOv1_380010	putative thiosulfate sulfurtransferase; Rhodanese-like domain/ankyrin repeat domain protein	44	49
METTOv1_420012	Succinate-semialdehyde dehydrogenase [NADP+] (SSDH)	44	46
METTOv1_430015	protein of unknown function	44	38
METTOv1_430021	Peptidase U62 modulator of DNA gyrase	44	47
METTOv1_470014	conserved protein of unknown function	44	51
METTOv1_570024	fragment of protein of unknown function (part 1)	44	44
METTOv1_590026	cytochrome c assembly protein	44	40
METTOv1_600026	peptidase M48 Ste24p	44	46
METTOv1_690007	MCP methyltransferase, CheR-type	44	48
METTOv1_1390002	DNA mismatch repair protein mutL (fragment)	44	42
METTOv1_10126	exported protein of unknown function	43	42
METTOv1_20095	protein of unknown function	43	48
METTOv1_30011	conserved protein of unknown function	43	45
METTOv1_30021	protein of unknown function	43	38
METTOv1_50048	putative transcriptional regulator, arsR family	43	49

METTOv1_50062	protein of unknown function	43	42
METTOv1_60028	conserved protein of unknown function; putative membrane protein, Rhomboid-like family	43	40
METTOv1_70040	Putative phytochelatin synthase (fragment)	43	33
METTOv1_70044	protein of unknown function	43	31
METTOv1_90027	conserved membrane protein of unknown function	43	63
METTOv1_100003	exported protein of unknown function	43	37
METTOv1_120040	conserved exported protein of unknown function	43	41
METTOv1_170079	protein of unknown function	43	53
METTOv1_180009	Amidase	43	38
METTOv1_200017	multicopper oxidase type 3	43	36
METTOv1_230003	protein of unknown function	43	30
METTOv1_240045	conserved protein of unknown function	43	18
METTOv1_280001	putative pyridoxal-dependent decarboxylase	43	44
METTOv1_280051	protein of unknown function DUF159	43	32
METTOv1_300031	transcriptional regulator, MarR family	43	42
METTOv1_330024	protein of unknown function	43	41
METTOv1_360004	4-diphosphocytidyl-2-C-methyl-D-erythritol kinase	43	37
METTOv1_370008	protein of unknown function DUF482	43	34
METTOv1_370031	protein of unknown function	43	39
METTOv1_430030	conserved membrane protein of unknown function	43	32
METTOv1_450017	protein of unknown function	43	92
METTOv1_460021	protein of unknown function	43	50
METTOv1_460024	TonB-dependent siderophore receptor	43	44
METTOv1_540021	Phosphatidylserine/phosphatidylglycerophosphate/ cardiolipin synthase-like protein (fragment)	43	41
METTOv1_610017	arsenate reductase	43	41
METTOv1_700022	protein of unknown function	43	42
METTOv1_730011	putative transaminase	43	28
METTOv1_910001	protein of unknown function	43	19
METTOv1_1550001	protein of unknown function	43	46
METTOv1_30020	protein of unknown function	42	60
METTOv1_30151	putative phage related integrase	42	37
METTOv1_50012	Smr protein/MutS2	42	45
METTOv1_70047	MotA/TolQ/ExbB proton channel	42	43
METTOv1_80095	protein of unknown function	42	44
METTOv1_90039	exported protein of unknown function	42	34
METTOv1_150027	DNA protecting protein DprA	42	51
METTOv1_180011	Putative cyclase (fragment)	42	38
METTOv1_210032	Diguanylate cyclase/phosphodiesterase with PAS/PAC and GAF sensor(S) (fragment)	42	38
METTOv1_220050	transposase	42	41
METTOv1_300022	conserved protein of unknown function	42	25
METTOv1_380022	Holliday junction ATP-dependent DNA helicase ruvA	42	44
METTOv1_390012	sun protein	42	43
METTOv1_460007	protein of unknown function	42	60
METTOv1_490004	protein of unknown function	42	28
METTOv1_620008	exported protein of unknown function	42	39
METTOv1_640003	hydroxyacylglutathione hydrolase	42	40
METTOv1_650009	protein of unknown function	42	35
METTOv1_700007	NUDIX hydrolase	42	24
METTOv1_740004	membrane protein of unknown function	42	43
METTOv1_930003	Mg chelatase, subunit ChlI	42	50

METTOv1_970009	protein of unknown function	42	20
METTOv1_1450003	TonB family protein (fragment)	42	51
METTOv1_10130	Phosphoribosylanthranilate isomerase	41	30
METTOv1_20036	thiamine monophosphate synthase	41	46
METTOv1_30013	conserved protein of unknown function	41	46
METTOv1_30014	putative CRISPR-associatedhD domain protein	41	37
METTOv1_40042	O-methyltransferase family 2	41	35
METTOv1_70039	NADH:flavin oxidoreductase/NADH oxidase	41	47
METTOv1_70060	conserved exported protein of unknown function	41	26
METTOv1_70078	gamma-glutamyl kinase	41	40
METTOv1_90033	acetate kinase	41	28
METTOv1_90073	protein of unknown function DUF447	41	32
METTOv1_90086	membrane protein of unknown function	41	43
METTOv1_110069	protein of unknown function	41	40
METTOv1_160013	conserved protein of unknown function	41	47
METTOv1_180078	MiaB-like tRNA modifying enzyme	41	43
METTOv1_190059	ABC transporter related	41	53
METTOv1_200028	Fmu (Sun) domain protein	41	41
METTOv1_250003	Type I site-specific deoxyribonuclease protein R	41	44
METTOv1_250034	Uroporphyrin-III C/tetrapyrrole (Corrin/Porphyrin) methyltransferase	41	41
METTOv1_320060	protein of unknown function	41	19
METTOv1_340029	putative ABC transporter, permease protein	41	38
METTOv1_380028	conserved membrane protein of unknown function	41	35
METTOv1_460020	two component transcriptional regulator, winged helix family	41	55
METTOv1_530007	Nucleotide-sugar dehydrogenase	41	43
METTOv1_570023	fragment of protein of unknown function (part 2)	41	42
METTOv1_800007	conserved protein of unknown function	41	40
METTOv1_10154	putative Histidine kinase	40	46
METTOv1_20038	Alpha/beta hydrolase fold-3 domain protein	40	39
METTOv1_20154	protein of unknown function	40	32
METTOv1_40036	glycine cleavage system T protein	40	42
METTOv1_40109	conserved membrane protein of unknown function	40	56
METTOv1_50056	protein of unknown function	40	38
METTOv1_70045	protein of unknown function	40	26
METTOv1_70046	Polypeptide-transport-associated domain protein, ShlB-type	40	25
METTOv1_120019	precorrin 6A synthase	40	61
METTOv1_130051	acyl-CoA thioesterase II	40	34
METTOv1_160011	glycosyl transferase family 2	40	42
METTOv1_210014	DNA polymerase III chi subunit HolC	40	33
METTOv1_320057	protein of unknown function	40	34
METTOv1_340004	exported protein of unknown function	40	45
METTOv1_370018	conserved exported protein of unknown function	40	27
METTOv1_410011	quinone oxidoreductase, YhdH/YhfP family	40	35
METTOv1_410019	putative OmpA/MotB domain protein	40	40
METTOv1_420038	conserved protein of unknown function	40	39
METTOv1_430017	conserved protein of unknown function; GCN5-related N-acetyltransferase	40	41
METTOv1_490013	CheW protein	40	33
METTOv1_490014	Methyl-accepting chemotaxis sensory transducer	40	37
METTOv1_650010	Glutamine-dependent NAD(+) synthetase	40	36
METTOv1_680009	phosphopantothenoylcysteine decarboxylase/phosphopantothenate/cysteine ligase	40	37

METTOv1_790012	conserved protein of unknown function	40	41
METTOv1_840012	dihydroorotate dehydrogenase	40	39
METTOv1_930002	Putative Sensor histidine kinase with PAS and Response regulator receiver domains (fragment)	40	45
METTOv1_930012	Glutathione S-transferase domain protein	40	47
METTOv1_970010	protein of unknown function	40	47
METTOv1_980007	protein of unknown function	40	47
METTOv1_1030010	Resolvase domain protein (fragment)	40	52
METTOv1_1080001	Long-chain-fatty-acid--CoA ligase	40	41
METTOv1_1250001	protein of unknown function	40	26
METTOv1_1530001	protein of unknown function	40	41
METTOv1_1670001	protein of unknown function	40	32
METTOv1_10074	conserved membrane protein of unknown function	39	33
METTOv1_20131	Electron transfer flavoprotein alpha subunit	39	38
METTOv1_70031	Cobyric acid synthase	39	34
METTOv1_80078	protein of unknown function	39	55
METTOv1_100038	protein of unknown function	39	25
METTOv1_150005	conserved exported protein of unknown function	39	32
METTOv1_150009	2-dehydropantoate 2-reductase	39	45
METTOv1_150024	Putative Holliday junction resolvase	39	20
METTOv1_190036	protein of unknown function	39	39
METTOv1_200051	protein of unknown function	39	36
METTOv1_250007	protein of unknown function	39	44
METTOv1_310042	putative two-component response regulator, with HTH luxR family	39	30
METTOv1_320066	protein of unknown function	39	44
METTOv1_340006	protein of unknown function DUF1499	39	32
METTOv1_390034	multi-sensor signal transduction histidine kinase	39	32
METTOv1_400007	periplasmic solute binding protein	39	32
METTOv1_400036	N-acetylmuramyl-L-alanine amidase, negative regulator of AmpC, AmpD	39	51
METTOv1_420025	conserved protein of unknown function	39	50
METTOv1_490015	exported protein of unknown function	39	35
METTOv1_560012	molybdenum ABC transporter, periplasmic molybdate-binding protein	39	49
METTOv1_590009	conserved exported protein of unknown function	39	19
METTOv1_620003	sulfate ABC transporter, inner membrane subunit CysT	39	28
METTOv1_660008	protein of unknown function	39	50
METTOv1_680014	2OG-Fe(II) oxygenase	39	36
METTOv1_710016	Zinc-containing alcohol dehydrogenase superfamily	39	37
METTOv1_980008	conserved protein of unknown function	39	27
METTOv1_1100001	protein of unknown function	39	41
METTOv1_1130006	conserved protein of unknown function	39	22
METTOv1_1400001	DNA-invertase (fragment)	39	51
METTOv1_10168	Glycosyl transferase family 2	38	37
METTOv1_20077	protein of unknown function	38	25
METTOv1_30027	conserved protein of unknown function	38	41
METTOv1_40015	histidine biosynthesis protein	38	45
METTOv1_60007	Anthranilate phosphoribosyltransferase	38	34
METTOv1_100054	protein of unknown function	38	37
METTOv1_140078	conserved protein of unknown function; putative membrane protein	38	34
METTOv1_150026	protein of unknown function	38	17

METTOv1_210015	Methyltransferase type 11	38	37
METTOv1_230007	protein of unknown function	38	37
METTOv1_230027	conserved protein of unknown function	38	57
METTOv1_270061	putative SAM-dependent methyltransferse	38	41
METTOv1_280064	heme exporter protein CcmA	38	42
METTOv1_320050	membrane protein of unknown function	38	23
METTOv1_360029	putative TonB dependent receptor	38	38
METTOv1_370042	GCN5-related N-acetyltransferase	38	45
METTOv1_410008	AAA ATPase central domain protein	38	32
METTOv1_590021	dimethyladenosine transferase	38	37
METTOv1_680017	uroporphyrin-III C-methyltransferase	38	41
METTOv1_900003	protein of unknown function	38	50
METTOv1_1050009	Nicotinamide phosphoribosyltransferase	38	35
METTOv1_1140001	Cytosine-specific methyltransferase (fragment)	38	39
METTOv1_10203	protein of unknown function	37	50
METTOv1_20037	Sel1 domain protein repeat-containing protein	37	34
METTOv1_20117	nitrogenase molybdenum-iron cofactor biosynthesis protein NifN	37	34
METTOv1_40127	protein of unknown function	37	32
METTOv1_40133	conserved protein of unknown function	37	47
METTOv1_60049	conserved exported protein of unknown function	37	40
METTOv1_120015	precorrin-6y C5,15-methyltransferase (decarboxylating), CbiE subunit	37	27
METTOv1_160018	protein of unknown function	37	22
METTOv1_190008	putative enzyme	37	37
METTOv1_190067	uroporphyrinogen III synthase HEM4	37	27
METTOv1_200005	protein of unknown function DUF58	37	39
METTOv1_200027	protein of unknown function	37	43
METTOv1_230071	conserved protein of unknown function	37	30
METTOv1_310029	ABC-2 type transporter	37	33
METTOv1_320056	conserved protein of unknown function	37	28
METTOv1_340035	protein of unknown function DUF214	37	38
METTOv1_350019	putative acyl-CoA N-acyltransferase related protein	37	31
METTOv1_380043	glycerophosphoryl diester phosphodiesterase	37	30
METTOv1_440044	integral membrane protein MviN	37	28
METTOv1_450025	putative Sensor protein	37	40
METTOv1_470011	Phage terminase GpA (fragment)	37	46
METTOv1_500004	NIPSNAP family containing protein	37	45
METTOv1_550019	UDP-hexose transferase	37	36
METTOv1_610028	Aldehyde Dehydrogenase	37	37
METTOv1_790014	UDP-N-acetylglucosamine 2-epimerase	37	30
METTOv1_10143	L-lactate dehydrogenase, FMN-linked	36	41
METTOv1_20003	putative oxidoreductase protein	36	22
METTOv1_20053	protein of unknown function	36	34
METTOv1_30031	putative Sensor protein	36	31
METTOv1_30142	protein of unknown function	36	45
METTOv1_70003	protein of unknown function	36	28
METTOv1_70030	cobalamin biosynthesis protein CobD	36	34
METTOv1_70048	Transport protein ExbD	36	36
METTOv1_70090	conserved protein of unknown function	36	34
METTOv1_80042	D-3-phosphoglycerate dehydrogenase	36	32
METTOv1_90084	Putative two-component sensor histidine kinase with a Hamp domain	36	40

METTOv1_130035	protein of unknown function	36	60
METTOv1_140016	Sensor protein (fragment)	36	34
METTOv1_150025	putative Secreted protein	36	40
METTOv1_180082	conserved exported protein of unknown function	36	46
METTOv1_210037	multi-sensor signal transduction histidine kinase	36	26
METTOv1_220060	exported protein of unknown function	36	30
METTOv1_240001	putative Signal transduction histidine kinase, glucose-6-phosphate specific	36	41
METTOv1_240069	putative transcriptional regulator, XRE family (modular protein)	36	14
METTOv1_250042	major facilitator superfamily MFS_1	36	43
METTOv1_290036	Integrase catalytic region (fragment)	36	33
METTOv1_350032	protein of unknown function	36	29
METTOv1_350035	DNA replication and repair protein RecF	36	34
METTOv1_360015	conserved protein of unknown function	36	35
METTOv1_360053	Glycosyl transferase family 28	36	41
METTOv1_380046	carbohydrate kinase, YjeF related protein	36	38
METTOv1_390021	DNA polymerase III, alpha subunit	36	34
METTOv1_410002	protein of unknown function	36	27
METTOv1_530010	conserved protein of unknown function	36	38
METTOv1_560009	TonB family protein	36	50
METTOv1_670011	CHRD domain containing protein	36	35
METTOv1_920002	protein of unknown function	36	43
METTOv1_980015	HipA protein	36	26
METTOv1_1110007	putative Copper resistance protein, copD family. (fragment)	36	29
METTOv1_1120006	conserved membrane protein of unknown function	36	27
METTOv1_1150007	adenosylmethionine-8-amino-7-oxononanoate aminotransferase	36	31
METTOv1_1240001	protein of unknown function	36	64
METTOv1_1460002	conserved protein of unknown function	36	56
METTOv1_30088	Phage integrase	35	35
METTOv1_50018	dephospho-CoA kinase	35	32
METTOv1_50074	conserved exported protein of unknown function	35	34
METTOv1_120054	putative Diguanylate cyclase	35	24
METTOv1_150004	Ser/Thr protein phosphatase family protein	35	32
METTOv1_150042	Regulator of chromosome condensation RCC1 (fragment)	35	32
METTOv1_160005	Alpha,alpha-trehalose-phosphate synthase (UDP-forming) precursor	35	35
METTOv1_220002	protein of unknown function	35	34
METTOv1_230075	ABC phosphate/phosphonate transporter, periplasmic ligand binding protein	35	46
METTOv1_230078	HTH-type transcriptional repressor nsrR	35	33
METTOv1_240040	conserved protein of unknown function	35	42
METTOv1_360017	transcriptional regulator, ArsR family	35	30
METTOv1_370017	conserved membrane protein of unknown function	35	24
METTOv1_390013	Heparinase II/III family protein	35	28
METTOv1_430008	GumN family protein	35	32
METTOv1_450004	conserved protein of unknown function	35	36
METTOv1_450012	efflux transporter, RND family, MFP subunit	35	25
METTOv1_490024	putative uncharacterized iron-regulated membrane protein	35	33
METTOv1_500005	Enoyl-[acyl-carrier protein] reductase	35	31
METTOv1_520028	potassium:proton antiporter	35	26
METTOv1_610012	protein of unknown function	35	24
METTOv1_610019	regulatory protein ArsR	35	36

METTOv1_640016	protein of unknown function DUF490	35	29
METTOv1_680007	transcriptional regulator, BadM/Rrf2 family	35	30
METTOv1_680008	Deoxyuridine 5'-triphosphate nucleotidohydrolase	35	29
METTOv1_700012	Putative voltage-dependent potassium channel with a cyclic nucleotide-binding domain	35	30
METTOv1_750006	protein of unknown function	35	40
METTOv1_780011	2-dehydro-3-deoxyphosphooctonate aldolase	35	34
METTOv1_830006	conserved protein of unknown function	35	42
METTOv1_970004	cationic amino acid ABC transporter, periplasmic binding protein	35	34
METTOv1_1000003	sigma 54-dependent transcriptional regulator of rtcBA expression	35	39
METTOv1_1130003	thiamine-monophosphate kinase	35	29
METTOv1_1470001	conserved protein of unknown function	35	37
METTOv1_10098	membrane protein of unknown function	34	43
METTOv1_20066	phosphoribosyltransferase	34	28
METTOv1_30032	putative Chemotaxis protein methyltransferase	34	34
METTOv1_50096	bifunctional protein : 2-acylglycerophosphoethanolamine acyltransferase; Acyl-ACP synthetase	34	31
METTOv1_90020	putative helicase, superfamily II	34	33
METTOv1_150044	conserved exported protein of unknown function	34	26
METTOv1_150046	Peptide chain release factor 1	34	25
METTOv1_170008	protein of unknown function	34	32
METTOv1_190058	binding-protein-dependent transport systems inner membrane component	34	32
METTOv1_240070	conserved protein of unknown function	34	39
METTOv1_300004	protein of unknown function	34	30
METTOv1_340001	TonB-dependent receptor	34	27
METTOv1_430026	protein of unknown function	34	27
METTOv1_470017	exported protein of unknown function	34	35
METTOv1_470029	protein of unknown function	34	14
METTOv1_520022	deoxyguanosinetriphosphate triphosphohydrolase	34	28
METTOv1_530017	glutathione S-transferase	34	28
METTOv1_560013	protein of unknown function	34	28
METTOv1_610022	transcriptional regulator, XRE family	34	30
METTOv1_660013	protein of unknown function	34	37
METTOv1_710013	Phosphopantetheinyl transferase protein	34	29
METTOv1_780010	squalene-associated FAD-dependent desaturase	34	35
METTOv1_900011	putative ABC transporter, ATP-binding protein	34	29
METTOv1_950012	putative Short-chain dehydrogenase/reductase SDR	34	35
METTOv1_1080002	high-affinity nickel-transporter	34	37
METTOv1_1300001	major facilitator superfamily MFS_1	34	30
METTOv1_20010	protein of unknown function DUF72	33	34
METTOv1_20083	Stomatin family protein	33	35
METTOv1_30003	protein of unknown function	33	50
METTOv1_40040	putative glucose/sorbosone dehydrogenase	33	36
METTOv1_50097	Transcriptional regulator, TetR family	33	24
METTOv1_50116	AAA ATPase central domain protein	33	36
METTOv1_80026	exported protein of unknown function	33	52
METTOv1_90024	protein of unknown function	33	25
METTOv1_100039	protein of unknown function	33	26
METTOv1_130027	phosphoribosylamine/glycine ligase (modular protein)	33	30
METTOv1_180077	conserved protein of unknown function	33	37
METTOv1_220006	protein of unknown function	33	53

METTOv1_220020	pseudouridine synthase	33	33
METTOv1_230002	protein of unknown function	33	30
METTOv1_230041	protein of unknown function	33	35
METTOv1_230053	protein of unknown function	33	41
METTOv1_240024	conserved protein of unknown function; putative Rhodanese-like domain protein	33	23
METTOv1_260025	protein of unknown function	33	20
METTOv1_340034	conserved protein of unknown function	33	35
METTOv1_370020	protein of unknown function	33	33
METTOv1_460003	conserved protein of unknown function	33	56
METTOv1_560014	Tetratricopeptide TPR_2 repeat protein	33	31
METTOv1_570012	AMP-dependent synthetase and ligase	33	31
METTOv1_620009	Formamidopyrimidine-DNA glycosylase	33	29
METTOv1_620010	conserved membrane protein of unknown function	33	28
METTOv1_630005	Alpha/beta hydrolase fold	33	30
METTOv1_740016	DMT superfamily permease	33	39
METTOv1_790013	putative Glycosyl transferase group 1	33	34
METTOv1_1010001	protein of unknown function	33	49
METTOv1_1150002	conserved protein of unknown function	33	21
METTOv1_1150006	dethiobiotin synthetase	33	31
METTOv1_10072	conserved membrane protein of unknown function	32	31
METTOv1_10096	conserved protein of unknown function	32	30
METTOv1_10161	conserved protein of unknown function	32	27
METTOv1_10181	Thioesterase	32	29
METTOv1_20028	Radical SAM domain protein	32	18
METTOv1_20123	protein of unknown function	32	37
METTOv1_20125	Fe-S cluster assembly protein NifU	32	36
METTOv1_20175	protein of unknown function	32	19
METTOv1_30077	protein of unknown function	32	26
METTOv1_50019	Maf family protein	32	32
METTOv1_80075	DNA binding domain protein, excisionase family	32	32
METTOv1_130006	Glycine amidinotransferase	32	33
METTOv1_130007	Quaternary ammonium compound-resistance protein sugE (modular protein)	32	30
METTOv1_140060	exported protein of unknown function	32	30
METTOv1_150041	conserved exported protein of unknown function	32	26
METTOv1_150068	metallophosphoesterase	32	42
METTOv1_160044	Mandelate racemase/muconate lactonizing protein	32	37
METTOv1_180019	putative H(+)-transporting ATP synthase, gene 1 protein	32	28
METTOv1_190025	DNA-directed DNA polymerase	32	35
METTOv1_290005	conserved protein of unknown function	32	32
METTOv1_350008	cytochrome c biogenesis protein transmembrane region	32	17
METTOv1_420013	conserved protein of unknown function	32	33
METTOv1_480001	UvrD/REP helicase	32	45
METTOv1_530019	putative Histidine kinase	32	27
METTOv1_610013	protein of unknown function	32	14
METTOv1_660007	putative Sensor protein	32	37
METTOv1_660012	protein of unknown function	32	27
METTOv1_690001	putative efflux protein	32	36
METTOv1_840011	protein of unknown function DUF952	32	26
METTOv1_950003	Putative transposase; putative IS66 family element, orf2	32	39
METTOv1_1370001	chromosome segregation protein SMC (fragment)	32	38
METTOv1_10038	protein of unknown function	31	60

METTOv1_20008	putative transcriptional regulator, XRE family	31	18
METTOv1_20052	Ceramide glucosyltransferase	31	32
METTOv1_20165	protein of unknown function	31	24
METTOv1_30033	putative Histidine kinase	31	30
METTOv1_40038	fragment of Glycine dehydrogenase [decarboxylating] (part 1)	31	33
METTOv1_40039	fragment of Glycine dehydrogenase [decarboxylating] (part 2)	31	31
METTOv1_40069	protein of unknown function	31	47
METTOv1_50028	MgtC/SapB transporter	31	28
METTOv1_50065	Patatin	31	36
METTOv1_70004	protein of unknown function	31	9
METTOv1_90005	putative ABC multidrug transport system, fused ATPase and permease domain	31	34
METTOv1_100057	Putative cobaltochelatase cobN subunit	31	24
METTOv1_110089	excinuclease ABC, C subunit	31	27
METTOv1_150008	conserved protein of unknown function	31	27
METTOv1_160020	Transcriptional regulatory protein basR	31	27
METTOv1_160043	membrane protein of unknown function	31	29
METTOv1_180012	protein of unknown function	31	37
METTOv1_210059	Cytochrome c3 hydrogenase alpha (Or beta) chain	31	26
METTOv1_220053	conserved protein of unknown function	31	28
METTOv1_230032	Mercuric resistance operon regulatory protein	31	39
METTOv1_230059	protein of unknown function	31	24
METTOv1_240025	putative Regulatory protein, TetR	31	30
METTOv1_290004	protein of unknown function DUF182	31	25
METTOv1_300008	Methyltransferase type 11	31	22
METTOv1_350001	protein of unknown function	31	40
METTOv1_360035	conserved membrane protein of unknown function	31	30
METTOv1_530005	Glycosyl transferase group 1	31	29
METTOv1_590028	Outer membrane protein	31	23
METTOv1_650020	conserved membrane protein of unknown function	31	24
METTOv1_710014	putative Polyketide biosynthesis acyltransferase homolog pksD	31	33
METTOv1_730010	conserved membrane protein of unknown function	31	29
METTOv1_790009	membrane protein of unknown function	31	27
METTOv1_920007	FAD dependent oxidoreductase	31	29
METTOv1_1000009	Resolvase domain protein (fragment)	31	33
METTOv1_1210002	putative TonB-dependent receptor protein	31	32
METTOv1_10167	conserved exported protein of unknown function	30	31
METTOv1_10186	Putative Two-component sensor histidine kinase	30	23
METTOv1_20009	conserved exported protein of unknown function	30	30
METTOv1_30010	protein of unknown function	30	42
METTOv1_30107	Gcn5-related n-acetyltransferase protein	30	37
METTOv1_60067	conserved protein of unknown function	30	38
METTOv1_120018	cobyrinic acid a,c-diamide synthase	30	28
METTOv1_130047	Poly-beta-hydroxybutyrate polymerase domain protein	30	30
METTOv1_130073	conserved protein of unknown function	30	45
METTOv1_140017	Uncharacterized glycosyltransferase ykcC	30	32
METTOv1_140027	protein of unknown function	30	18
METTOv1_140039	sodium-proton antiporter	30	25
METTOv1_140058	Triose-phosphate isomerase	30	28
METTOv1_220007	protein of unknown function	30	75
METTOv1_250004	protein of unknown function	30	33
METTOv1_250014	conserved protein of unknown function	30	21

METTOv1_260026	conserved protein of unknown function; putative membrane protein	30	18
METTOv1_350010	conserved protein of unknown function	30	29
METTOv1_350021	conserved protein of unknown function	30	40
METTOv1_350031	AIG2 family protein	30	21
METTOv1_350055	Na+ dependent nucleoside transporter domain protein	30	20
METTOv1_370014	Cytochrome P450	30	29
METTOv1_380014	putative TonB-dependent receptor protein	30	26
METTOv1_410015	methyltransferase small	30	27
METTOv1_430018	polysaccharide deacetylase	30	23
METTOv1_470025	protein of unknown function	30	36
METTOv1_560007	Chloride channel core (modular protein)	30	27
METTOv1_800016	protein of unknown function	30	34
METTOv1_890009	Sensor protein	30	35
METTOv1_970011	protein of unknown function	30	25
METTOv1_980006	UBA/THIF-type NAD/FAD binding protein	30	30
METTOv1_980013	protein of unknown function	30	26
METTOv1_1060007	protein of unknown function	30	29
METTOv1_1230003	conserved protein of unknown function; putative nicotinic acid mononucleotide (NMN) biosynthesis protein	30	27
METTOv1_20004	protein of unknown function	29	22
METTOv1_20057	Putative chitinase (modular protein)	29	32
METTOv1_40067	exported protein of unknown function	29	22
METTOv1_70049	conserved protein of unknown function	29	24
METTOv1_90022	Undecaprenyl-phosphate glucose phosphotransferase (fragment)	29	40
METTOv1_120007	putative macrolide ABC transporter, fusion of ATP-binding (N-terminal) and membrane (C-terminal) domains	29	29
METTOv1_150001	acid phosphatase	29	17
METTOv1_170002	protein of unknown function	29	32
METTOv1_170089	XorII very short patch repair endonuclease	29	22
METTOv1_180023	exported protein of unknown function	29	27
METTOv1_210039	Radical SAM domain protein	29	27
METTOv1_220047	putative Major facilitator superfamily MFS_1	29	23
METTOv1_270020	hydrogenase expression/formation protein HypE	29	44
METTOv1_290021	ABC phosphate/phosphonate transporter, periplasmic ligand binding protein	29	29
METTOv1_290041	protein of unknown function	29	43
METTOv1_380044	conserved protein of unknown function	29	24
METTOv1_400037	putative Oxidoreductase, short-chain dehydrogenase/reductase family	29	21
METTOv1_480006	putative oxidoreductase	29	15
METTOv1_790015	Glycosyl transferase, group 1	29	26
METTOv1_1110005	membrane protein involved in the export of O-antigen and teichoic acid-like protein	29	27
METTOv1_1150004	Rieske (2Fe-2S) domain protein	29	19
METTOv1_20069	integrase family protein	28	24
METTOv1_20073	Conjugal transfer protein traD (modular protein)	28	42
METTOv1_20126	cysteine desulfurase NifS	28	31
METTOv1_20158	Phage integrase	28	22
METTOv1_20180	protein of unknown function	28	23
METTOv1_30041	putative Chase2 sensor protein	28	26
METTOv1_40001	Uracil DNA glycosylase (fragment)	28	28

METTOv1_40135	alpha-2-macroglobulin domain protein	28	31
METTOv1_60132	Phosphoesterase	28	23
METTOv1_70038	phosphoenolpyruvate synthase	28	26
METTOv1_80066	Transcriptional regulator, ArsR family	28	27
METTOv1_100010	sulfate adenylyltransferase subunit 2	28	32
METTOv1_100060	iron-dicitrate transporter subunit ; ATP-binding component of ABC superfamily; KpLE2 phage-like element	28	23
METTOv1_140061	conserved protein of unknown function	28	27
METTOv1_160010	glycosyl transferase group 1	28	24
METTOv1_160034	ATP-dependent helicase	28	29
METTOv1_160049	SAM-dependent methyltransferase	28	30
METTOv1_170027	conserved exported protein of unknown function	28	30
METTOv1_190044	conserved protein of unknown function	28	30
METTOv1_210034	Radical SAM	28	39
METTOv1_230031	protein of unknown function	28	26
METTOv1_240039	protein of unknown function	28	38
METTOv1_250040	Phosphatidylethanolamine N-methyltransferase	28	28
METTOv1_300002	protein of unknown function	28	58
METTOv1_320065	protein of unknown function	28	19
METTOv1_330025	protein of unknown function	28	27
METTOv1_480016	protein of unknown function	28	9
METTOv1_540012	conserved protein of unknown function	28	30
METTOv1_550015	Surfeit locus 1 family protein (fragment)	28	27
METTOv1_590002	protein of unknown function	28	17
METTOv1_610001	conserved membrane protein of unknown function	28	27
METTOv1_660014	protein of unknown function DUF6 transmembrane	28	21
METTOv1_790011	protein of unknown function	28	25
METTOv1_820014	two component transcriptional regulator, LuxR family	28	19
METTOv1_830017	ROK family protein	28	20
METTOv1_860002	protein of unknown function	28	23
METTOv1_980005	protein of unknown function	28	27
METTOv1_1060004	glutamyl-tRNA synthetase class Ic	28	23
METTOv1_20115	protein of unknown function	27	19
METTOv1_20152	Chromate transporter	27	20
METTOv1_40090	phosphomethylpyrimidine kinase	27	25
METTOv1_60019	Crossover junction endodeoxyribonuclease ruvC	27	29
METTOv1_70028	Adenosylcobinamide kinase	27	19
METTOv1_80076	ABC transporter molybdenum-binding protein	27	25
METTOv1_80089	Aminoglycoside phosphotransferase	27	26
METTOv1_90079	Transcriptional regulator protein	27	17
METTOv1_90081	HI0933 family protein	27	33
METTOv1_90082	protein of unknown function	27	31
METTOv1_130070	3-deoxy-manno-octulosonate cytidylyltransferase	27	20
METTOv1_160019	putative Sensor protein	27	27
METTOv1_180008	putative transcriptional regulatory protein, related to MarR family	27	28
METTOv1_190068	protein of unknown function	27	25
METTOv1_330011	Urease accessory protein UreD	27	29
METTOv1_330016	urea ABC transporter, urea binding protein	27	29
METTOv1_460027	protein of unknown function	27	32
METTOv1_470030	Peptidase S8 and S53 subtilisin kexin sedolisin	27	26
METTOv1_490007	protein of unknown function	27	26
METTOv1_490025	conserved exported protein of unknown function	27	16

METTOv1_520013	protein of unknown function	27	40
METTOv1_530020	exported protein of unknown function	27	31
METTOv1_630006	Major facilitator superfamily MFS_1	27	24
METTOv1_670016	protein of unknown function	27	19
METTOv1_680019	ATP-dependent DNA helicase RecQ	27	27
METTOv1_740008	putative TonB family protein	27	22
METTOv1_850001	periplasmic binding protein (modular protein)	27	42
METTOv1_880011	exported protein of unknown function	27	31
METTOv1_950004	fragment of putative transposase (part 1)	27	36
METTOv1_950011	protein of unknown function	27	33
METTOv1_960003	double-strand break repair helicase AddA	27	25
METTOv1_970013	Glycosyl transferase group 1	27	27
METTOv1_990009	putative transcriptional regulatory protein, TetR family.	27	36
METTOv1_1240005	transposase	27	34
METTOv1_1460003	protein of unknown function	27	43
METTOv1_10018	DNA-binding response regulator	26	24
METTOv1_30104	putative transmembrane protein	26	26
METTOv1_70036	putative TonB-dependent receptor protein	26	26
METTOv1_100007	Luciferase family protein	26	23
METTOv1_110003	exported protein of unknown function	26	41
METTOv1_110045	protein of unknown function	26	25
METTOv1_120017	precorrin-4 C11-methyltransferase	26	26
METTOv1_130032	Isoprenylcysteine carboxyl methyltransferase	26	26
METTOv1_150043	exported protein of unknown function	26	36
METTOv1_150047	conserved exported protein of unknown function	26	25
METTOv1_150063	conserved exported protein of unknown function	26	18
METTOv1_180010	Haloacetate dehalogenase (fragment)	26	17
METTOv1_190009	NUDIX hydrolase	26	32
METTOv1_210065	exported protein of unknown function	26	22
METTOv1_220070	histidine kinase	26	27
METTOv1_230040	Cation transporter MgtC/SapB (fragment)	26	13
METTOv1_240044	protein of unknown function	26	42
METTOv1_270024	hydrogenase accessory protein HypB	26	25
METTOv1_280018	alanine dehydrogenase oxidoreductase protein	26	34
METTOv1_290040	putative transcriptional regulator	26	20
METTOv1_330007	UreE urease accessory domain protein	26	29
METTOv1_400008	ABC-3 protein	26	21
METTOv1_450011	putative TonB-dependent receptor protein	26	26
METTOv1_520031	methyltransferase	26	29
METTOv1_610007	pyridoxamine 5'-phosphate oxidase-related FMN-binding	26	20
METTOv1_620025	protein of unknown function	26	23
METTOv1_670022	protein of unknown function	26	22
METTOv1_700018	putative Polysaccharide biosynthesis protein	26	24
METTOv1_790008	protein of unknown function	26	18
METTOv1_810011	conserved protein of unknown function	26	25
METTOv1_1010003	transposase (fragment)	26	43
METTOv1_1070009	putative transposase	26	26
METTOv1_1150003	putative aldo/keto reductase, NAD(P)-binding	26	25
METTOv1_10145	protein of unknown function	25	23
METTOv1_40062	glycosyl transferase family 51	25	26
METTOv1_70053	conserved protein of unknown function	25	20
METTOv1_80065	putative peroxiredoxin 2	25	24
METTOv1_90083	two component transcriptional regulator, LuxR family	25	16

METTOv1_90085	protein of unknown function	25	27
METTOv1_110078	DNA repair protein RadA	25	22
METTOv1_120016	Putative CobE protein	25	13
METTOv1_130037	Putative component of multidrug efflux system; putative signal peptide	25	21
METTOv1_130038	acriflavin resistance protein	25	27
METTOv1_130041	protein of unknown function	25	28
METTOv1_140018	protein of unknown function	25	21
METTOv1_140022	ATP-dependent DNA helicase RecG	25	21
METTOv1_150003	Cytochrome-c peroxidase	25	22
METTOv1_190065	DNA polymerase III, delta subunit	25	23
METTOv1_280065	heme exporter protein CcmB	25	32
METTOv1_290039	acriflavin resistance protein	25	26
METTOv1_330006	Urease accessory protein UreF	25	29
METTOv1_330012	urea ABC transporter, ATP-binding protein UrtE	25	29
METTOv1_340053	UPF0060 membrane protein Msil_1658	25	29
METTOv1_370013	putative Beta-(1-->2)glucan export ATP-binding protein chvA	25	20
METTOv1_470033	protein of unknown function	25	24
METTOv1_490009	Response regulator receiver domain protein (CheY-like)	25	38
METTOv1_610010	Transcriptional regulator, LysR family	25	24
METTOv1_630012	Diguanylate cyclase	25	24
METTOv1_640017	protein of unknown function	25	31
METTOv1_670006	Allophanate hydrolase subunit 1	25	17
METTOv1_710002	Polyketide biosynthesis 3-hydroxy-3-methylglutaryl-ACP synthase pksG	25	24
METTOv1_760013	protein of unknown function	25	26
METTOv1_800015	protein of unknown function	25	28
METTOv1_880009	Anti-FecI sigma factor, FecR	25	18
METTOv1_970012	protein of unknown function	25	16
METTOv1_980016	protein of unknown function	25	40
METTOv1_1150005	7-keto-8-aminopelargonic acid synthetase	25	23
METTOv1_10153	NTE family protein rssA	24	30
METTOv1_20070	putative Prophage CP4-57 regulatory protein	24	31
METTOv1_20100	LRV FeS4 cluster domain protein	24	21
METTOv1_30066	Phage terminase-like protein	24	22
METTOv1_30092	exported protein of unknown function	24	16
METTOv1_30131	conserved protein of unknown function	24	29
METTOv1_30143	protein of unknown function	24	20
METTOv1_50091	protein of unknown function DUF477	24	28
METTOv1_60015	protein of unknown function	24	47
METTOv1_70023	protein of unknown function	24	6
METTOv1_80084	Potassium-transporting ATPase C chain	24	29
METTOv1_90023	protein of unknown function	24	22
METTOv1_110085	conserved protein of unknown function	24	22
METTOv1_150007	conserved protein of unknown function	24	38
METTOv1_180017	ATP synthase A chain (ATPase protein 6)	24	18
METTOv1_180027	Lipid-A-disaccharide synthase	24	19
METTOv1_180047	putative General secretion pathway protein H	24	23
METTOv1_220023	protein of unknown function	24	26
METTOv1_240019	Putative Metallo-beta-lactamase family protein	24	19
METTOv1_240026	Glycosyltransferase 28 domain protein	24	22
METTOv1_240053	conserved protein of unknown function	24	18
METTOv1_290038	efflux transporter, RND family, MFP subunit	24	28

METTOv1_320048	conserved protein of unknown function	24	38
METTOv1_360007	Hydrogenase maturation protease	24	18
METTOv1_370046	conserved protein of unknown function	24	16
METTOv1_450007	putative Phenylalanine racemase (ATP-hydrolyzing)	24	18
METTOv1_460010	copper-transporting P-type ATPase (modular protein)	24	24
METTOv1_470002	protein of unknown function	24	29
METTOv1_480020	two-component response regulator	24	21
METTOv1_490008	CheA Signal Transduction Histidine Kinases (STHK)	24	25
METTOv1_520023	exported protein of unknown function	24	28
METTOv1_540024	conserved membrane protein of unknown function	24	23
METTOv1_570017	putative PepSY-associated TM helix	24	21
METTOv1_580002	putative Alkanal monooxygenase (FMN-linked)	24	22
METTOv1_660015	putative manganese transport protein	24	25
METTOv1_680004	protein of unknown function	24	27
METTOv1_690013	Glutathione-dependent formaldehyde-activating GFA	24	21
METTOv1_700005	conserved protein of unknown function	24	23
METTOv1_710017	putative Zinc-binding dehydrogenase	24	31
METTOv1_790010	Cyclopropane-fatty-acyl-phospholipid	24	17
METTOv1_900010	putative ABC transporter, permease protein	24	18
METTOv1_1010007	putative Molybdenum ABC transporter, periplasmic molybdate-binding protein	24	22
METTOv1_1050006	transposase (fragment)	24	15
METTOv1_10117	conserved protein of unknown function	23	22
METTOv1_50086	oxidoreductase FAD/NAD(P)-binding domain protein	23	19
METTOv1_60073	protein of unknown function	23	18
METTOv1_80009	protein of unknown function	23	22
METTOv1_90021	protein of unknown function	23	20
METTOv1_90078	quinone oxidoreductase	23	24
METTOv1_130065	A/G-specific adenine glycosylase	23	24
METTOv1_150060	Aldose 1-epimerase	23	20
METTOv1_160004	HAD-superfamily hydrolase subfamily IIB	23	23
METTOv1_170028	protein of unknown function	23	42
METTOv1_180001	exodeoxyribonuclease VII, large subunit	23	20
METTOv1_220051	conserved protein of unknown function	23	22
METTOv1_220056	Ribosomal small subunit Rsm22	23	20
METTOv1_230034	conserved protein of unknown function	23	28
METTOv1_250005	protein of unknown function	23	21
METTOv1_250011	Amino acid permease-associated region (fragment)	23	21
METTOv1_260029	conserved protein of unknown function	23	17
METTOv1_270001	protein of unknown function	23	22
METTOv1_300014	CDP-alcohol phosphatidyltransferase	23	20
METTOv1_330026	putative RhiD protein	23	23
METTOv1_370019	exported protein of unknown function	23	20
METTOv1_370036	protein of unknown function	23	32
METTOv1_380012	two-component response regulator	23	19
METTOv1_480007	conserved protein of unknown function	23	14
METTOv1_490016	putative DNA-binding response regulator	23	24
METTOv1_500020	ABC transporter related	23	18
METTOv1_520012	protein of unknown function	23	32
METTOv1_530006	conserved protein of unknown function	23	23
METTOv1_540011	conserved protein of unknown function	23	22
METTOv1_560018	protein of unknown function DUF1501	23	24
METTOv1_560020	Carboxymethylenebutenolidase	23	21

METTOv1_580001	protein of unknown function	23	22
METTOv1_600017	phosphoserine phosphatase SerB	23	18
METTOv1_600025	RNA polymerase sigma-32 factor	23	31
METTOv1_650012	conserved protein of unknown function	23	39
METTOv1_800004	conjugation TrbI family protein	23	31
METTOv1_820003	conserved protein of unknown function	23	23
METTOv1_910007	Resolvase, N-terminal domain (fragment)	23	30
METTOv1_980001	putative plasmid stabilization protein (fragment) (fragment)	23	46
METTOv1_1100006	conserved protein of unknown function	23	17
METTOv1_1350002	DNA mismatch repair protein mutL (fragment)	23	20
METTOv1_1370002	exported protein of unknown function	23	11
METTOv1_10097	protein of unknown function	22	13
METTOv1_10201	putative Sensor protein	22	27
METTOv1_10216	DNA mismatch repair protein MutS	22	27
METTOv1_20007	conserved membrane protein of unknown function	22	24
METTOv1_20072	putative conjugual transfert protein, traC	22	97
METTOv1_20151	Chromate transporter	22	28
METTOv1_30076	protein of unknown function	22	14
METTOv1_30078	protein of unknown function	22	17
METTOv1_50001	fragment of protein of unknown function (part 1)	22	17
METTOv1_50102	conserved membrane protein of unknown function	22	16
METTOv1_120020	protein of unknown function	22	45
METTOv1_150006	exported protein of unknown function	22	33
METTOv1_170001	protein of unknown function	22	27
METTOv1_210058	Cyclic nucleotide-binding protein, hydrogenase accessory protein, HoxI	22	25
METTOv1_230070	protein of unknown function	22	25
METTOv1_270018	putative sensor histidine kinase with a PAS domain	22	24
METTOv1_280010	conserved protein of unknown function	22	24
METTOv1_290034	Alcohol dehydrogenase zinc-binding domain protein	22	10
METTOv1_300003	conserved protein of unknown function	22	35
METTOv1_300007	protein of unknown function	22	21
METTOv1_310054	protein of unknown function	22	17
METTOv1_340003	putative ECF sigma factor protein	22	17
METTOv1_370016	iron-sulfur cluster binding protein	22	20
METTOv1_460008	protein of unknown function	22	48
METTOv1_460016	Acriflavin resistance protein	22	21
METTOv1_460022	putative transcriptional regulator, XRE family (modular protein)	22	37
METTOv1_480017	conserved exported protein of unknown function	22	8
METTOv1_530004	putative Glycosyl transferase group 1	22	16
METTOv1_580003	protein of unknown function	22	20
METTOv1_590011	acriflavin resistance protein	22	27
METTOv1_610023	protein of unknown function DUF336	22	15
METTOv1_640004	protein of unknown function DUF985	22	27
METTOv1_690004	protein of unknown function	22	29
METTOv1_700006	membrane protein of unknown function	22	20
METTOv1_890003	exported protein of unknown function	22	33
METTOv1_920008	Methyltransferase type 11	22	30
METTOv1_990008	putative membrane fusion protein (MFP) component of efflux pump, membrane anchor; UPF0194 family	22	16
METTOv1_10016	exported protein of unknown function	21	28
METTOv1_10209	HTH-type transcriptional regulator pecS	21	16
METTOv1_20060	4Fe-4S ferredoxin iron-sulfur binding domain protein	21	19

METTOv1_20078	protein of unknown function	21	20
METTOv1_20128	serine O-acetyltransferase	21	18
METTOv1_50090	protein of unknown function DUF477	21	14
METTOv1_60018	major facilitator superfamily MFS_1	21	26
METTOv1_60074	transcriptional regulator, LysR family	21	22
METTOv1_70041	gamma-glutamyltranspeptidase	21	27
METTOv1_90013	putative Cytochrome c, class I	21	34
METTOv1_100009	sulfate adenylyltransferase, large subunit	21	29
METTOv1_100050	multi-sensor hybrid histidine kinase	21	24
METTOv1_110090	Iron complex outermembrane recepter protein	21	19
METTOv1_130063	conserved membrane protein of unknown function	21	18
METTOv1_140059	exported protein of unknown function	21	20
METTOv1_160041	protein of unknown function	21	26
METTOv1_180014	ATP synthase subunit alpha (ATPase subunit alpha) (ATP synthase F1 sector subunit alpha)	21	15
METTOv1_180020	putative ATP synthase F1, epsilon subunit	21	21
METTOv1_200035	ATPase, P-type (Transporting), HAD superfamily, subfamily IC (modular protein)	21	19
METTOv1_240057	NAD-dependent epimerase/dehydratase	21	25
METTOv1_250043	Citrate transporter	21	19
METTOv1_280003	putative DNA-binding protein	21	17
METTOv1_340002	putative Anti-FecI sigma factor, FecR	21	18
METTOv1_350048	Fatty acid hydroxylase	21	22
METTOv1_370022	protein of unknown function	21	20
METTOv1_430014	GCN5-related N-acetyltransferase	21	22
METTOv1_470021	conserved protein of unknown function	21	16
METTOv1_490012	Chemotaxis response regulator protein-glutamate methylesterase 3	21	17
METTOv1_490022	putative FecR protein	21	16
METTOv1_490026	protein of unknown function	21	17
METTOv1_560019	protein of unknown function	21	18
METTOv1_620023	Pyruvate kinase	21	16
METTOv1_660006	DNA-binding response regulator	21	28
METTOv1_730001	transposase	21	8
METTOv1_740003	phosphoesterase PA-phosphatase related (fragment)	21	23
METTOv1_840001	double-strand break repair protein AddB (fragment)	21	15
METTOv1_890002	putative FecR, iron siderophore sensor protein	21	16
METTOv1_940001	protein of unknown function	21	25
METTOv1_1140005	protein of unknown function	21	29
METTOv1_1140006	protein of unknown function	21	22
METTOv1_1300002	protein of unknown function	21	15
METTOv1_1340002	tRNA(Ile)-lysidine synthetase	21	14
METTOv1_1420002	protein of unknown function	21	24
METTOv1_1580002	protein of unknown function	21	30
METTOv1_10023	Macrolide export ATP-binding/permease protein macB	20	14
METTOv1_10191	membrane protein of unknown function	20	23
METTOv1_10204	molybdate ABC transporter, ATPase subunit	20	19
METTOv1_30005	protein of unknown function	20	20
METTOv1_30006	protein of unknown function	20	51
METTOv1_30098	tonB-system energizer ExbB	20	19
METTOv1_30140	protein of unknown function	20	12
METTOv1_30144	protein of unknown function	20	7
METTOv1_40076	putative phage related integrase	20	19

METTOv1_50084	Methane monooxygenase	20	19
METTOv1_60042	putative Oxidoreductase FAD/NAD(P)-binding protein	20	17
METTOv1_60072	conserved membrane protein of unknown function	20	15
METTOv1_120026	transport system permease protein	20	29
METTOv1_150071	conserved protein of unknown function	20	23
METTOv1_180013	H+transporting two-sector ATPase gamma subunit	20	21
METTOv1_190040	protein of unknown function	20	17
METTOv1_190042	GCN5-related N-acetyltransferase (modular protein)	20	17
METTOv1_200050	Enolase-phosphatase E1	20	21
METTOv1_240017	putative aminotransferase	20	25
METTOv1_460017	putative membrane fusion protein MtrC	20	21
METTOv1_470023	protein of unknown function	20	18
METTOv1_470034	protein of unknown function	20	19
METTOv1_480005	D-threo-aldose 1-dehydrogenase protein	20	23
METTOv1_480018	conserved exported protein of unknown function	20	17
METTOv1_490011	PBS lyase HEAT-like repeat protein	20	17
METTOv1_510028	protein of unknown function	20	35
METTOv1_570002	putative outer membrane hemin/siderophore receptor protein	20	22
METTOv1_600019	Major facilitator superfamily MFS_1	20	19
METTOv1_610009	putative transcriptional regulator, LysR family	20	11
METTOv1_610015	Arsenical pump-driving ATPase (Arsenite-translocating ATPase) (Arsenical resistance ATPase) (Arsenite-transporting ATPase)	20	18
METTOv1_660001	protein of unknown function	20	27
METTOv1_690003	putative ABC transporter (fused ATP-binding and permease components)	20	18
METTOv1_710004	putative polyketide biosynthesis enoyl-CoA isomerase pksI	20	20
METTOv1_730002	transposase	20	13
METTOv1_740010	MotA/TolQ/ExbB proton channel	20	21
METTOv1_800019	conserved protein of unknown function	20	23
METTOv1_990004	conserved protein of unknown function	20	24
METTOv1_1020010	transposase	20	12
METTOv1_1070006	protein of unknown function	20	15
METTOv1_10146	DNA-binding transcriptional dual regulator, Fe-S center for redox-sensing	19	15
METTOv1_10210	Cyanate permease	19	20
METTOv1_30072	protein of unknown function	19	23
METTOv1_30093	molybdenum ABC transporter, periplasmic molybdate-binding protein	19	13
METTOv1_30099	Biopolymer transport protein ExbD/TolR	19	34
METTOv1_30161	putative FecR	19	24
METTOv1_40121	Enoyl-CoA hydratase/isomerase	19	14
METTOv1_50083	monooxygenase component MmoB/DmpM	19	14
METTOv1_50085	Methane monooxygenase component D	19	13
METTOv1_80021	sn-glycerol-3-phosphate dehydrogenase FAD/NAD(P)-binding (aerobic)	19	19
METTOv1_100082	elongation factor G (EF-G)	19	19
METTOv1_180018	conserved exported protein of unknown function	19	17
METTOv1_180074	Transcriptional regulator, GntR family with aminotransferase domain	19	18
METTOv1_200037	Lipolytic protein G-D-S-L family	19	28
METTOv1_210018	putrescine transport protein (ABC superfamily, membrane)	19	19
METTOv1_220009	protein of unknown function	19	20

METTOv1_230028	conserved protein of unknown function; putative membrane protein	19	30
METTOv1_230033	protein of unknown function	19	24
METTOv1_230077	Hydroxylamine reductase	19	16
METTOv1_240071	protein of unknown function	19	18
METTOv1_250009	conserved protein of unknown function	19	19
METTOv1_250012	Methyltransferase type 11	19	21
METTOv1_270005	protein of unknown function	19	19
METTOv1_270019	two component sigma-54-dependent hydrogenase transcriptional regulator, Fis family	19	18
METTOv1_270051	UPF0337 protein bsl1473	19	15
METTOv1_320052	Phage integrase	19	11
METTOv1_320054	putative Portal protein, HK97 family	19	16
METTOv1_360049	putative TPR domain protein	19	20
METTOv1_390003	putative nitroreductase protein	19	20
METTOv1_420027	Manganese containing catalase superfamily	19	17
METTOv1_420028	protein of unknown function	19	8
METTOv1_450010	putative FecR	19	20
METTOv1_690021	Sua5/YciO/YrdC/YwlC family protein	19	13
METTOv1_790006	Sugar transferase	19	14
METTOv1_1040008	transposase	19	17
METTOv1_1060001	conserved protein of unknown function	19	21
METTOv1_1210004	putative RNA polymerase sigma factor fecI	19	14
METTOv1_20006	protein of unknown function	18	19
METTOv1_20169	protein of unknown function	18	21
METTOv1_30002	conserved protein of unknown function	18	24
METTOv1_30004	protein of unknown function	18	20
METTOv1_30049	aldo/keto reductase	18	17
METTOv1_50115	nitroreductase	18	14
METTOv1_60081	protein of unknown function	18	10
METTOv1_60084	exported protein of unknown function	18	26
METTOv1_70054	putative Filamentous haemagglutinin family outer membrane protein	18	18
METTOv1_90015	PQQ-dependent dehydrogenase, methanol/ethanol family	18	17
METTOv1_90016	DNA topoisomerase III	18	22
METTOv1_100083	protein of unknown function	18	21
METTOv1_150074	putative ATPase (yhcM) AFG1 family	18	13
METTOv1_170003	conserved protein of unknown function	18	17
METTOv1_170068	conserved protein of unknown function	18	23
METTOv1_200041	Amino acid/peptide transporter	18	9
METTOv1_210017	putrescine transport protein (ABC superfamily, membrane)	18	15
METTOv1_220001	Glycosyl transferase group 1	18	18
METTOv1_220027	conserved protein of unknown function	18	9
METTOv1_220045	protein of unknown function	18	17
METTOv1_230026	protein of unknown function	18	26
METTOv1_240027	conserved membrane protein of unknown function	18	27
METTOv1_250053	protein of unknown function	18	18
METTOv1_270002	RNA polymerase, sigma-24 subunit, ECF subfamily	18	23
METTOv1_290032	Glycosyl transferase group 1	18	25
METTOv1_290037	conserved exported protein of unknown function	18	20
METTOv1_350005	conserved protein of unknown function	18	21
METTOv1_430020	Sensor protein kdpD	18	20
METTOv1_450018	protein of unknown function	18	29

METTOv1_460018	putative Outer membrane efflux protein	18	14
METTOv1_470006	transposase (fragment)	18	21
METTOv1_470026	protein of unknown function	18	24
METTOv1_470035	protein of unknown function	18	23
METTOv1_560025	protein of unknown function	18	14
METTOv1_580005	Peroxisomal 2,4-dienoyl-CoA reductase SPS19	18	17
METTOv1_610008	protein of unknown function DUF1348	18	11
METTOv1_610029	putative transcriptional regulatory protein, Fis family	18	12
METTOv1_630004	Transcriptional regulator, LysR family	18	20
METTOv1_670014	exported protein of unknown function	18	16
METTOv1_690002	putative ABC transporter (ATP-binding protein)	18	22
METTOv1_740009	Biopolymer transport protein ExbD/TolR	18	7
METTOv1_790002	exported protein of unknown function	18	10
METTOv1_800009	protein of unknown function	18	17
METTOv1_950010	Glycosyl transferase family 28 (fragment)	18	9
METTOv1_1030006	protein of unknown function	18	8
METTOv1_1100004	conserved protein of unknown function	18	20
METTOv1_10019	putative TonB-dependent siderophore receptor	17	19
METTOv1_10073	P-type ATPase, Mg2+ ATPase transport protein	17	13
METTOv1_10174	conserved protein of unknown function	17	10
METTOv1_20016	protein of unknown function	17	21
METTOv1_20122	NifQ family protein	17	12
METTOv1_30029	putative LysR-type regulator	17	25
METTOv1_30073	Single-strand binding protein/Primosomal replication protein n	17	28
METTOv1_30102	protein of unknown function	17	19
METTOv1_50063	Citrate (Pro-3S)-lyase	17	15
METTOv1_50104	membrane protein of unknown function	17	11
METTOv1_60001	conserved protein of unknown function	17	26
METTOv1_80079	polysaccharide deacetylase	17	19
METTOv1_80087	conserved protein of unknown function	17	22
METTOv1_90006	ABC transporter related	17	14
METTOv1_100051	DEAD/H associated domain protein	17	15
METTOv1_150011	exported protein of unknown function	17	20
METTOv1_200008	Polynucleotide adenylyltransferase region	17	18
METTOv1_210071	tRNA (guanine-N1)-methyltransferase	17	23
METTOv1_240067	protein of unknown function	17	20
METTOv1_320020	conserved protein of unknown function	17	9
METTOv1_380011	putative Sensor protein	17	15
METTOv1_460023	FecR protein (fragment)	17	18
METTOv1_480009	Transcriptional regulator	17	12
METTOv1_510012	Activator of Hsp90 ATPase-like (modular protein)	17	22
METTOv1_510027	TonB-dependent receptor	17	12
METTOv1_570016	putative TonB-dependent receptor protein	17	16
METTOv1_600018	tRNA dimethylallyltransferase	17	9
METTOv1_610014	arsenical-resistance protein	17	9
METTOv1_780015	protein of unknown function	17	21
METTOv1_800003	putative conjugal transfer protein trbG	17	8
METTOv1_800012	conserved protein of unknown function	17	23
METTOv1_820001	exported protein of unknown function	17	6
METTOv1_820008	conserved protein of unknown function	17	17
METTOv1_990007	putative transporter fused subunits of ABC superfamily: ATP-binding components	17	15
METTOv1_10105	conserved exported protein of unknown function	16	18

METTOv1_10110	protein of unknown function	16	13
METTOv1_10172	MarR family transcriptional regulatory protein	16	13
METTOv1_20166	protein of unknown function	16	12
METTOv1_30025	putative TonB-dependent siderophore receptor	16	22
METTOv1_30097	Polypeptide-transport-associated domain protein ShlB-type	16	16
METTOv1_30132	exported protein of unknown function	16	21
METTOv1_30133	protein of unknown function	16	9
METTOv1_70079	Putative intradiol ring-cleavage dioxygenase (modular protein)	16	20
METTOv1_70086	Protein-L-isoaspartate(D-aspartate) O-methyltransferase	16	11
METTOv1_80007	TonB-dependent receptor	16	17
METTOv1_80058	putative acyltransferase	16	17
METTOv1_100049	putative Sensor protein	16	12
METTOv1_160063	protein of unknown function	16	14
METTOv1_170020	putative phage major tail tube protein	16	22
METTOv1_210010	Transcriptional regulator, LysR family	16	11
METTOv1_210062	Dihydrofolate reductase	16	22
METTOv1_230054	transposase (fragment)	16	18
METTOv1_250018	putative Ribosomal protein L11 methylase-like protein	16	18
METTOv1_260034	protein of unknown function	16	6
METTOv1_320062	protein of unknown function	16	16
METTOv1_320068	extracellular solute-binding protein family 5	16	15
METTOv1_390002	protein of unknown function	16	12
METTOv1_450009	RNA polymerase, sigma-24 subunit, ECF subfamily	16	18
METTOv1_460002	FMN-dependent NADH-azoreductase	16	17
METTOv1_470008	protein of unknown function	16	16
METTOv1_470022	conserved protein of unknown function	16	32
METTOv1_470027	protein of unknown function	16	20
METTOv1_500006	putative Transcriptional regulator, TetR family	16	15
METTOv1_550002	hemolysin A	16	14
METTOv1_560010	Polypeptide-transport-associated domain protein ShlB-type	16	12
METTOv1_560017	protein of unknown function DUF1549	16	17
METTOv1_670025	Anaerobic glycerol-3-phosphate dehydrogenase subunit C	16	11
METTOv1_710019	conserved exported protein of unknown function	16	22
METTOv1_750003	protein of unknown function	16	15
METTOv1_800006	transcriptional regulatory protein (fragment)	16	18
METTOv1_850008	protein of unknown function	16	7
METTOv1_910010	conserved protein of unknown function	16	14
METTOv1_920009	conserved membrane protein of unknown function	16	19
METTOv1_990005	putative transporter subunit: permease component of ABC superfamily	16	15
METTOv1_1020011	RNA polymerase, sigma-24 subunit, ECF subfamily	16	13
METTOv1_1460005	protein of unknown function	16	10
METTOv1_10116	conserved protein of unknown function	15	14
METTOv1_20030	protein of unknown function	15	13
METTOv1_20116	nitrogenase MoFe cofactor biosynthesis protein NifE	15	18
METTOv1_20160	protein of unknown function	15	26
METTOv1_20161	protein of unknown function	15	11
METTOv1_30053	putative TonB-dependent receptor protein	15	18
METTOv1_30094	conserved exported protein of unknown function	15	19
METTOv1_40072	conserved protein of unknown function	15	14
METTOv1_40075	protein of unknown function	15	10
METTOv1_40136	fragment of protein of unknown function (part 2)	15	21
METTOv1_50103	protein of unknown function	15	11

METTOv1_70002	putative TonB-dependent receptor protein	15	16
METTOv1_70035	putative Protein fecR	15	14
METTOv1_80096	conserved protein of unknown function	15	20
METTOv1_90041	putative RNA polymerase sigma factor fecI	15	18
METTOv1_140040	anhydro-N-acetylmuramic acid kinase	15	12
METTOv1_170047	conserved protein of unknown function	15	14
METTOv1_170049	conserved protein of unknown function	15	23
METTOv1_180007	conserved membrane protein of unknown function	15	23
METTOv1_260056	protein of unknown function	15	11
METTOv1_270038	NADH ubiquinone oxidoreductase 20 kDa subunit	15	12
METTOv1_320064	protein of unknown function	15	16
METTOv1_330019	integral membrane sensor hybrid histidine kinase	15	9
METTOv1_400040	putative ATP-dependent DNA helicase recQ	15	14
METTOv1_440036	exported protein of unknown function	15	18
METTOv1_470028	protein of unknown function	15	15
METTOv1_470032	conserved protein of unknown function	15	13
METTOv1_480019	putative ATPase, histidine kinase-, DNA gyrase B-, and HSP90-like domain protein	15	10
METTOv1_480023	putative Acetylornithine transaminase	15	15
METTOv1_500014	conserved protein of unknown function	15	18
METTOv1_530018	putative Acyltransferase 3	15	10
METTOv1_650008	protein of unknown function	15	17
METTOv1_650021	exported protein of unknown function	15	17
METTOv1_710001	putative Beta-ketoacyl-acyl-carrier-protein synthase II	15	10
METTOv1_710003	putative Enoyl-CoA hydratase/isomerase	15	9
METTOv1_770007	protein of unknown function	15	15
METTOv1_790007	membrane protein of unknown function	15	10
METTOv1_810010	putative Acyltransferase family protein	15	17
METTOv1_840003	PAS/PAC sensor signal transduction histidine kinase (fragment)	15	14
METTOv1_880004	Cytochrome P450	15	13
METTOv1_920001	protein of unknown function	15	20
METTOv1_920006	NADH-ubiquinone oxidoreductase 39-40 kDa subunit-like protein	15	13
METTOv1_990010	protein of unknown function	15	26
METTOv1_1140008	protein of unknown function	15	10
METTOv1_1250002	putative transcriptional regulator, GntR family protein	15	9
METTOv1_1660001	conjugal transfer protein trbL (fragment)	15	16
METTOv1_30075	conserved protein of unknown function	14	10
METTOv1_40035	conserved protein of unknown function	14	3
METTOv1_40105	putative Nitrous oxidase accessory protein	14	8
METTOv1_40126	ABC transporter ATP-binding protein	14	11
METTOv1_50066	putative Pyrrolo-quinoline quinone	14	13
METTOv1_50112	conserved exported protein of unknown function	14	13
METTOv1_60025	protein of unknown function	14	18
METTOv1_110091	Anti-sigma factor, FecR-like protein (fragment)	14	13
METTOv1_120056	exported protein of unknown function	14	15
METTOv1_150077	protein of unknown function	14	16
METTOv1_160035	conserved protein of unknown function	14	13
METTOv1_190031	protein of unknown function	14	10
METTOv1_190045	Putative Glutathionylspermidine synthase family protein	14	9
METTOv1_210056	Sulfhydrogenase delta subunit	14	8
METTOv1_230048	protein of unknown function	14	11
METTOv1_320055	Prohead protease	14	14

METTOv1_340013	DNA polymerase III, alpha subunit	14	12
METTOv1_370025	protein of unknown function	14	6
METTOv1_430001	conserved protein of unknown function	14	15
METTOv1_470031	protein of unknown function	14	14
METTOv1_480022	putative arylsulfatase	14	12
METTOv1_490010	Chemotaxis protein methyltransferase 2	14	21
METTOv1_500019	transport system permease protein	14	11
METTOv1_500024	cysteine desulfurase, SufS subfamily	14	12
METTOv1_530003	Glycosyl transferase family 2	14	7
METTOv1_550003	putative RNA methyltransferase	14	15
METTOv1_570014	two-component response regulator	14	15
METTOv1_650022	transcriptional regulator, AraC family	14	17
METTOv1_660009	Amino acid adenylation domain protein	14	14
METTOv1_730017	protein of unknown function	14	10
METTOv1_900004	putative Sensor protein	14	10
METTOv1_900005	two-component response regulator	14	19
METTOv1_930014	protein of unknown function	14	23
METTOv1_990003	Thioesterase	14	9
METTOv1_1070002	conserved protein of unknown function	14	16
METTOv1_1320001	protein of unknown function	14	5
METTOv1_1460004	protein of unknown function	14	14
METTOv1_1680001	exported protein of unknown function	14	35
METTOv1_30054	putative Protein fecR	13	11
METTOv1_30067	putative Phage terminase, small subunit, , P27	13	14
METTOv1_30141	protein of unknown function	13	11
METTOv1_30156	primosome assembly protein PriA	13	10
METTOv1_30159	protein of unknown function	13	22
METTOv1_40016	exported protein of unknown function	13	15
METTOv1_40068	protein of unknown function	13	12
METTOv1_40070	conserved protein of unknown function	13	12
METTOv1_40110	Cytochrome c oxidase, subunit I	13	12
METTOv1_50054	protein of unknown function	13	7
METTOv1_50082	Methane monooxygenase	13	9
METTOv1_70034	putative RNA polymerase, sigma-24 subunit, ECF subfamily	13	16
METTOv1_80060	Sulfide-quinone reductase	13	8
METTOv1_120058	protein of unknown function	13	11
METTOv1_130068	Non-hemolytic phospholipase C precursor	13	14
METTOv1_130069	Phosphoglycerate mutase	13	13
METTOv1_150072	putative Uncharacterized N-acetyltransferase yuaI	13	11
METTOv1_160021	protein of unknown function	13	14
METTOv1_170014	putative Phage late control D family protein	13	12
METTOv1_170029	protein of unknown function	13	18
METTOv1_170058	protein of unknown function	13	27
METTOv1_180048	putative General secretion pathway protein I	13	12
METTOv1_210027	Serine/threonine protein kinase	13	10
METTOv1_210055	conserved protein of unknown function	13	14
METTOv1_210057	Oxidoreductase FAD/NAD(P)-binding domain protein	13	11
METTOv1_220021	Membrane bound O-acyl transferase MBOAT family protein	13	13
METTOv1_230017	protein of unknown function	13	16
METTOv1_230043	Na/Pi-cotransporter II-related protein	13	13
METTOv1_240075	putative conjugal transfer protein trbG	13	10
METTOv1_250047	exported protein of unknown function	13	8
METTOv1_260048	protein of unknown function	13	9

METTOv1_270025	Probable hydrogenase nickel incorporation protein hypA	13	22
METTOv1_290031	conserved protein of unknown function	13	12
METTOv1_290042	protein of unknown function	13	13
METTOv1_300050	putative O-antigen polymerase	13	13
METTOv1_300052	putative ABC transporter related	13	14
METTOv1_330020	two component transcriptional regulator, LuxR family	13	9
METTOv1_380013	exported protein of unknown function	13	17
METTOv1_460019	Sensor protein	13	14
METTOv1_470019	Peptidase U35, phage prohead HK97	13	16
METTOv1_470037	conserved exported protein of unknown function	13	20
METTOv1_500003	protein of unknown function	13	19
METTOv1_570015	exported protein of unknown function	13	17
METTOv1_690005	protein of unknown function	13	25
METTOv1_700019	putative esterase/lipase/thioesterase	13	14
METTOv1_750012	conserved protein of unknown function	13	12
METTOv1_770006	putative Transcriptional regulator, LysR family	13	12
METTOv1_800002	conjugal transfer protein TrbF	13	8
METTOv1_890004	putative RNA polymerase ECF-type sigma factor, FecI	13	17
METTOv1_900001	Dimodular nonribosomal peptide synthase (fragment)	13	15
METTOv1_910008	conserved protein of unknown function	13	12
METTOv1_920011	protein of unknown function	13	10
METTOv1_940006	protein of unknown function	13	10
METTOv1_1000005	conserved protein of unknown function	13	13
METTOv1_1000006	RNA 3'-terminal phosphate cyclase	13	14
METTOv1_1070008	DotH	13	13
METTOv1_10106	conserved exported protein of unknown function	12	9
METTOv1_10150	Capsular exopolysaccharide family	12	11
METTOv1_20062	conserved protein of unknown function	12	12
METTOv1_20127	pyruvate carboxyltransferase	12	7
METTOv1_20171	protein of unknown function	12	8
METTOv1_30017	CRISPR-associated protein, Cas1 family / CRISPR-associated exonuclease, Cas4 family	12	8
METTOv1_30034	putative Phospholipase D	12	13
METTOv1_40018	Glycine betaine/L-proline ABC transporter, permease and substrate-binding protein	12	13
METTOv1_40047	conserved protein of unknown function	12	8
METTOv1_40071	protein of unknown function	12	9
METTOv1_40119	isovaleryl-CoA dehydrogenase	12	13
METTOv1_50101	protein of unknown function	12	8
METTOv1_60080	putative NADPH-dependent FMN reductase	12	9
METTOv1_70014	protein of unknown function	12	23
METTOv1_70037	protein of unknown function	12	21
METTOv1_70042	protein of unknown function	12	11
METTOv1_70043	RNA polymerase, sigma-24 subunit, ECF subfamily	12	13
METTOv1_80003	putative two component sensor histidine kinase; putative osmolarity sensor	12	14
METTOv1_80030	phosphate transporter	12	11
METTOv1_100017	putative Polysaccharide biosynthesis protein	12	11
METTOv1_100019	protein of unknown function	12	13
METTOv1_100033	protein of unknown function	12	13
METTOv1_100047	ornithine aminotransferase	12	13
METTOv1_100048	conserved protein of unknown function, putative amidinotransferase	12	3

METTOv1_160023	Protein of unknown function DUF1810	12	11
METTOv1_170018	protein of unknown function	12	22
METTOv1_190032	protein of unknown function	12	8
METTOv1_210054	Peptidase M52, hydrogen uptake protein	12	16
METTOv1_230025	conserved protein of unknown function	12	9
METTOv1_230073	conserved protein of unknown function	12	12
METTOv1_240074	conjugation TrbI family protein	12	10
METTOv1_270033	Hydrogenase maturation protein hupD	12	11
METTOv1_270052	Peptidase S10 serine carboxypeptidase	12	8
METTOv1_280002	putative 31 kDa outer-membrane immunogenic protein	12	14
METTOv1_290001	protein TolR (fragment)	12	20
METTOv1_290013	beta-lactamase domain protein	12	13
METTOv1_320058	exported protein of unknown function	12	11
METTOv1_320059	protein of unknown function	12	14
METTOv1_370026	Glycosyl transferase family 2 (fragment)	12	7
METTOv1_430013	heat shock protein DnaJ domain protein	12	11
METTOv1_460009	protein of unknown function	12	10
METTOv1_470016	protein of unknown function	12	10
METTOv1_470039	putative transposase	12	11
METTOv1_530001	protein of unknown function	12	7
METTOv1_560004	Acetyl-coenzyme A synthetase	12	14
METTOv1_560011	filamentous hemeagglutinin family outer membrane protein	12	12
METTOv1_560016	protein of unknown function	12	14
METTOv1_580006	putative Beta-ketoacyl-acyl-carrier-protein synthase II	12	11
METTOv1_650019	protein of unknown function	12	11
METTOv1_700021	putative ExoQ-like protein	12	12
METTOv1_750011	conserved protein of unknown function	12	10
METTOv1_800005	conserved protein of unknown function	12	20
METTOv1_800020	protein of unknown function	12	18
METTOv1_850009	transposase (fragment)	12	6
METTOv1_890001	exported protein of unknown function	12	9
METTOv1_920012	protein of unknown function	12	9
METTOv1_950006	putative transposase	12	15
METTOv1_960007	double-strand break repair protein AddB (fragment)	12	7
METTOv1_1000004	TROVE domain protein	12	9
METTOv1_1010004	Filamentous hemeagglutinin family outer membrane protein	12	12
METTOv1_1140004	protein of unknown function	12	12
METTOv1_1200001	protein of unknown function	12	13
METTOv1_1400002	transposase	12	10
METTOv1_10043	TonB-dependent receptor	11	10
METTOv1_10092	exported protein of unknown function	11	12
METTOv1_10115	conserved membrane protein of unknown function	11	13
METTOv1_10179	conserved protein of unknown function	11	7
METTOv1_10217	fragment of Penicillin amidase (part 1)	11	9
METTOv1_20170	protein of unknown function	11	19
METTOv1_30045	Maleylacetoacetate isomerase, MAAI (Glutathione S- transferase zeta 1, GSTZ1-1)	11	8
METTOv1_30052	conserved membrane protein of unknown function	11	7
METTOv1_30160	exported protein of unknown function	11	7
METTOv1_40048	Pyridine nucleotide-disulfide oxidoreductase	11	10
METTOv1_40094	Patatin	11	8
METTOv1_50080	chaperonin Cpn60/TCP-1	11	10

METTOv1_50113	Glycerol kinase (ATP:glycerol 3-phosphotransferase) (Glycerokinase) (GK)	11	13
METTOv1_70009	protein of unknown function	11	14
METTOv1_70013	protein of unknown function	11	28
METTOv1_70022	protein of unknown function	11	9
METTOv1_90009	Integral membrane protein TerC family (fragment)	11	5
METTOv1_90047	membrane protein of unknown function	11	12
METTOv1_100059	Uncharacterized ABC transporter permease protein yvrB	11	14
METTOv1_160022	putative TonB-dependent receptor protein	11	13
METTOv1_170050	protein of unknown function	11	14
METTOv1_190021	putative pyruvate dehydrogenase E1 component (Alpha subunit) oxidoreductase protein	11	7
METTOv1_230055	protein of unknown function	11	17
METTOv1_240068	protein of unknown function	11	13
METTOv1_250013	exported protein of unknown function	11	13
METTOv1_260016	putative replication protein A	11	9
METTOv1_260024	protein of unknown function	11	13
METTOv1_300027	Phospholipase/Carboxylesterase	11	13
METTOv1_320030	conserved protein of unknown function	11	7
METTOv1_320069	HTH-type transcriptional regulator DarR	11	13
METTOv1_330017	protein of unknown function	11	7
METTOv1_340050	protein of unknown function	11	5
METTOv1_350046	putative two-component sensor histidine kinase	11	12
METTOv1_380020	conserved exported protein of unknown function	11	12
METTOv1_400032	conserved membrane protein of unknown function	11	6
METTOv1_450026	conserved protein of unknown function	11	14
METTOv1_470018	putative bacteriophage-related protein	11	12
METTOv1_470038	transposase (fragment)	11	14
METTOv1_500018	protein of unknown function	11	9
METTOv1_500025	transcriptional regulator, BadM/Rrf2 family	11	12
METTOv1_510019	conserved exported protein of unknown function	11	9
METTOv1_660021	putative fumarate reductase/succinate dehydrogenase, flavoprotein subunit (fragment)	11	1
METTOv1_690023	conserved protein of unknown function	11	11
METTOv1_770002	protein of unknown function	11	12
METTOv1_900008	conserved membrane protein of unknown function	11	15
METTOv1_950001	Glycosyl transferase family 28 (fragment)	11	7
METTOv1_1010005	Tetratricopeptide TPR_2 repeat protein	11	14
METTOv1_1050008	transposase (fragment)	11	23
METTOv1_1260002	membrane protein of unknown function	11	13
METTOv1_10017	putative Sensor protein	10	13
METTOv1_10020	Twin-arginine translocation pathway signal	10	4
METTOv1_10021	Secretion protein HlyD (fragment)	10	5
METTOv1_20181	Integrase family protein (fragment)	10	9
METTOv1_30024	Nitrogenase	10	7
METTOv1_30046	4-hydroxyphenylpyruvate dioxygenase	10	9
METTOv1_30068	putative HNH nuclease / phage PHI-105 holin-like protein	10	6
METTOv1_40045	conserved protein of unknown function	10	16
METTOv1_40104	conserved exported protein of unknown function	10	1
METTOv1_40123	hydroxymethylglutaryl-CoA lyase	10	6
METTOv1_50077	membrane protein of unknown function	10	8
METTOv1_70001	Putative FecR (fragment)	10	12
METTOv1_80061	conserved exported protein of unknown function	10	20

METTOv1_80085	K+-transporting ATPase, B subunit	10	10
METTOv1_90042	putative FecR	10	9
METTOv1_100006	Twin-arginine translocation pathway signal (fragment)	10	14
METTOv1_100020	protein of unknown function	10	13
METTOv1_100032	Methyltransferase type 11	10	9
METTOv1_120027	Periplasmic binding protein	10	12
METTOv1_160040	protein of unknown function	10	14
METTOv1_170015	protein of unknown function	10	2
METTOv1_170039	Phage protein GP20	10	6
METTOv1_170040	putative phage protein GP19	10	5
METTOv1_170048	Cytosine-specific methyltransferase	10	15
METTOv1_170061	protein of unknown function	10	17
METTOv1_210029	putative outer membrane protein B; OmpB	10	14
METTOv1_230010	protein of unknown function	10	18
METTOv1_230020	putative ardC antirestriction protein	10	10
METTOv1_230072	Putative Conjugal transfer protein traG	10	9
METTOv1_240072	transcriptional regulatory protein (fragment)	10	7
METTOv1_260019	protein of unknown function	10	0
METTOv1_260051	protein of unknown function	10	12
METTOv1_270039	protein of unknown function	10	13
METTOv1_290016	GTP cyclohydrolase-2	10	6
METTOv1_290029	ABC transporter related	10	11
METTOv1_300023	putative regulatory protein (nitrile hydratase activator like)	10	7
METTOv1_300051	conserved exported protein of unknown function	10	12
METTOv1_320026	protein of unknown function	10	3
METTOv1_320063	protein of unknown function	10	9
METTOv1_360014	protein of unknown function	10	9
METTOv1_360051	conserved protein of unknown function, putative adenylate cyclase	10	10
METTOv1_420003	conserved protein of unknown function, putative MbtH-like protein	10	20
METTOv1_470020	GpW	10	27
METTOv1_500026	TonB-dependent receptor	10	9
METTOv1_500027	putative 31 kDa outer-membrane immunogenic protein	10	10
METTOv1_500028	TonB family protein (fragment)	10	11
METTOv1_510029	protein of unknown function	10	0
METTOv1_530002	Alcohol dehydrogenase zinc-binding domain protein	10	9
METTOv1_650014	methyltransferase	10	13
METTOv1_660020	protein of unknown function	10	2
METTOv1_800018	protein of unknown function	10	3
METTOv1_820013	exported protein of unknown function	10	4
METTOv1_890006	TonB-dependent receptor	10	9
METTOv1_900012	protein of unknown function	10	6
METTOv1_920003	UbiA prenyltransferase family protein	10	15
METTOv1_1000002	protein of unknown function	10	15
METTOv1_1020012	protein of unknown function	10	10
METTOv1_1060006	exported protein of unknown function	10	11
METTOv1_1140007	protein of unknown function	10	8
METTOv1_1210003	putative FecR	10	13
METTOv1_1260003	protein of unknown function	10	10
METTOv1_10022	Macrolide export ATP-binding/permease protein macB (fragment)	9	4
METTOv1_20071	putative Conjugal transfer protein, traA	9	9

METTOv1_20157	protein of unknown function	9	9
METTOv1_30001	protein of unknown function	9	11
METTOv1_30022	Plasmid stability protein stbB	9	12
METTOv1_30038	cytochrome o ubiquinol oxidase, subunit II	9	11
METTOv1_30062	exported protein of unknown function	9	16
METTOv1_30095	protein of unknown function	9	10
METTOv1_30100	protein of unknown function	9	11
METTOv1_30101	TonB family protein	9	10
METTOv1_30145	protein of unknown function	9	8
METTOv1_30149	conserved protein of unknown function	9	14
METTOv1_30155	exported protein of unknown function	9	14
METTOv1_30162	putative RNA polymerase sigma factor fecI	9	5
METTOv1_40046	Nitrogen-fixing NifU domain protein	9	8
METTOv1_50081	Methane monooxygenase	9	8
METTOv1_60039	bicupin, oxalate decarboxylase family	9	7
METTOv1_70016	protein of unknown function	9	10
METTOv1_70017	protein of unknown function	9	3
METTOv1_80005	conserved exported protein of unknown function	9	11
METTOv1_80088	exported protein of unknown function	9	13
METTOv1_90010	exported protein of unknown function	9	14
METTOv1_90012	ATPase, P-type (Transporting), HAD superfamily, subfamily IC	9	9
METTOv1_100042	protein of unknown function	9	2
METTOv1_110011	protein of unknown function	9	7
METTOv1_120025	Hemin import ATP-binding protein hmuV	9	9
METTOv1_120055	conserved exported protein of unknown function	9	11
METTOv1_150019	protein of unknown function	9	13
METTOv1_160062	protein of unknown function	9	5
METTOv1_170016	Phage tail assembly protein	9	8
METTOv1_170038	protein of unknown function	9	8
METTOv1_170059	protein of unknown function	9	13
METTOv1_190018	putative dihydrolipoamide acetyltransferase (Component e2 of pyruvate dehydrogenase complex) protein	9	12
METTOv1_190024	diguanylate cyclase	9	8
METTOv1_190070	4Fe-4S binding domain protein	9	8
METTOv1_200038	Sulfate transporter	9	13
METTOv1_210023	conserved protein of unknown function	9	9
METTOv1_220028	formate dehydrogenase, alpha subunit	9	7
METTOv1_230001	protein of unknown function	9	3
METTOv1_230021	conserved protein of unknown function	9	2
METTOv1_230069	transposase	9	8
METTOv1_250037	protein of unknown function	9	13
METTOv1_260001	conjugal transfer protein trbL (fragment)	9	9
METTOv1_270028	Probable rubredoxin hupI	9	3
METTOv1_270036	Uptake hydrogenase small subunit precursor	9	7
METTOv1_290010	protein of unknown function DUF938	9	17
METTOv1_310043	putative two-component sensor histidine kinase	9	7
METTOv1_320027	conserved protein of unknown function	9	1
METTOv1_320053	conserved protein of unknown function	9	10
METTOv1_330015	Putative Branched-chain amino acid ABC transporter (permease protein)	9	7
METTOv1_420004	JmjC domain protein	9	7
METTOv1_480004	protein of unknown function	9	4
METTOv1_480027	conserved protein of unknown function	9	5

METTOv1_490003	conserved protein of unknown function	9	8
METTOv1_500010	exported protein of unknown function	9	8
METTOv1_510011	transcriptional regulator, ArsR family	9	15
METTOv1_510025	Extracellular solute-binding protein family 1	9	11
METTOv1_550001	conserved exported protein of unknown function	9	10
METTOv1_660022	TonB-dependent receptor (fragment)	9	8
METTOv1_750013	Acyl transferase	9	7
METTOv1_790001	protein of unknown function	9	6
METTOv1_810009	major facilitator superfamily MFS_1	9	11
METTOv1_910012	VWA containing CoxE family protein	9	10
METTOv1_920004	FAD linked oxidase domain protein	9	8
METTOv1_990006	putative transporter subunit: permease component of ABC superfamily	9	7
METTOv1_1010002	protein of unknown function	9	11
METTOv1_1040003	Hemolysin-type calcium-binding region (fragment)	9	11
METTOv1_1060005	conserved membrane protein of unknown function	9	8
METTOv1_1070007	conserved protein of unknown function	9	11
METTOv1_1100005	putative plasmid stabilization protein	9	12
METTOv1_1130007	histone deacetylase superfamily (fragment)	9	11
METTOv1_10103	conserved membrane protein of unknown function	8	6
METTOv1_20082	Cobalamin synthase	8	7
METTOv1_20168	protein of unknown function	8	4
METTOv1_30007	protein of unknown function	8	6
METTOv1_30026	protein of unknown function	8	4
METTOv1_30055	putative RNA polymerase sigma factor fecI	8	8
METTOv1_30069	conserved protein of unknown function	8	2
METTOv1_30134	protein of unknown function	8	11
METTOv1_40073	protein of unknown function	8	5
METTOv1_50053	protein of unknown function	8	7
METTOv1_50079	protein of unknown function	8	8
METTOv1_60066	putative Transcriptional antitermination protein	8	10
METTOv1_70010	protein of unknown function	8	9
METTOv1_80004	protein of unknown function	8	7
METTOv1_80006	conserved protein of unknown function	8	18
METTOv1_80062	putative membrane protein; permease family	8	8
METTOv1_80064	putative enzyme with metallo-hydrolase/oxidoreductase domain (ycbL-like)	8	12
METTOv1_90014	putative methanol oxidation protein	8	5
METTOv1_100008	protein of unknown function	8	5
METTOv1_100014	membrane protein of unknown function	8	15
METTOv1_130062	Multimeric flavodoxin WrbA	8	24
METTOv1_160012	putative enzyme	8	9
METTOv1_160039	PRC-barrel domain protein	8	10
METTOv1_170019	conserved protein of unknown function	8	8
METTOv1_170026	Adenine specific DNA methyltransferase, D12 class	8	7
METTOv1_170046	conserved protein of unknown function	8	10
METTOv1_170054	protein of unknown function	8	9
METTOv1_170057	protein of unknown function	8	7
METTOv1_170060	protein of unknown function	8	12
METTOv1_170065	exported protein of unknown function	8	9
METTOv1_170072	Phage Gp37Gp68 (modular protein)	8	8
METTOv1_190038	conserved protein of unknown function	8	7
METTOv1_200040	Putative regulatory protein, LysR family	8	7

METTOv1_220072	conserved protein of unknown function	8	6
METTOv1_230008	protein of unknown function	8	1
METTOv1_230049	conserved protein of unknown function	8	8
METTOv1_230052	protein of unknown function	8	8
METTOv1_230062	putative replication protein A	8	7
METTOv1_250016	protein of unknown function	8	14
METTOv1_260005	conjugal transfer protein trbC	8	11
METTOv1_260012	putative conjugal transfer protein precursor	8	11
METTOv1_260052	protein of unknown function	8	7
METTOv1_270030	Hydrogenase expression/formation protein hupG	8	7
METTOv1_270031	Hydrogenase expression/formation protein HupF	8	4
METTOv1_270032	conserved exported protein of unknown function	8	18
METTOv1_290024	putative sulfite oxidase (soxC)	8	9
METTOv1_290030	Bifunctional deaminase-reductase domain protein	8	9
METTOv1_290035	Glycosyl transferase family 2	8	9
METTOv1_340014	protein of unknown function	8	8
METTOv1_350009	ComEC/Rec2-related protein	8	5
METTOv1_420026	protein of unknown function	8	15
METTOv1_420041	protein of unknown function	8	0
METTOv1_450013	acriflavin resistance protein	8	5
METTOv1_450014	acriflavin resistance protein	8	5
METTOv1_470024	Site-specific recombinase, phage integrase family (fragment)	8	8
METTOv1_490017	putative Histidine kinase	8	6
METTOv1_500015	protein of unknown function	8	11
METTOv1_510026	conserved protein of unknown function; putative thiol oxidoreductase	8	7
METTOv1_560015	protein of unknown function	8	5
METTOv1_570013	putative Sensor protein	8	4
METTOv1_590001	protein of unknown function	8	11
METTOv1_610011	conserved membrane protein of unknown function	8	6
METTOv1_610030	site-specific DNA recombinase; e14 prophage (fragment)	8	11
METTOv1_660002	acriflavin resistance protein	8	8
METTOv1_900007	putative TonB-dependent receptor protein	8	5
METTOv1_1200002	protein of unknown function	8	0
METTOv1_10091	protein of unknown function	7	10
METTOv1_10107	conserved protein of unknown function	7	5
METTOv1_10123	conserved protein of unknown function; putative membrane protein	7	8
METTOv1_10148	Protein-tyrosine-phosphatase	7	7
METTOv1_10152	conserved protein of unknown function	7	2
METTOv1_10175	conserved protein of unknown function	7	7
METTOv1_10198	TonB-dependent receptor	7	8
METTOv1_20019	conserved protein of unknown function	7	9
METTOv1_20061	conserved protein of unknown function	7	9
METTOv1_20089	Sugar transferase	7	5
METTOv1_30036	cytochrome o ubiquinol oxidase, subunit III	7	1
METTOv1_30047	homogentisate 1,2-dioxygenase	7	3
METTOv1_30048	Fumarylacetoacetate hydrolase family protein	7	7
METTOv1_30096	filamentous hemeagglutinin family outer membrane protein	7	9
METTOv1_40122	Carbamoyl-phosphate synthase L chain ATP-binding	7	6
METTOv1_50105	AMP-dependent synthetase and ligase	7	8
METTOv1_60053	membrane protein of unknown function	7	7

METTOv1_80059	conserved protein of unknown function, putative transmembrane protein precursor	7	5
METTOv1_80086	potassium-transporting ATPase, A subunit	7	6
METTOv1_90001	protein of unknown function	7	7
METTOv1_90002	conserved protein of unknown function	7	6
METTOv1_100031	protein of unknown function	7	12
METTOv1_100046	Aldehyde dehydrogenase family 7 member A1 homolog	7	9
METTOv1_120022	protein of unknown function	7	19
METTOv1_120023	conserved protein of unknown function	7	16
METTOv1_120024	conserved protein of unknown function	7	6
METTOv1_120053	Outer membrane efflux protein	7	11
METTOv1_130033	conserved protein of unknown function	7	13
METTOv1_170011	Phospholipase C/P1 nuclease domain-containing protein	7	6
METTOv1_170021	Putative major tail sheath protein FI (modular protein)	7	6
METTOv1_170045	conserved protein of unknown function	7	6
METTOv1_170052	conserved protein of unknown function	7	7
METTOv1_190037	protein of unknown function	7	6
METTOv1_190039	protein of unknown function	7	8
METTOv1_210013	protein of unknown function	7	5
METTOv1_230009	Integrase family protein	7	2
METTOv1_230024	conserved protein of unknown function	7	6
METTOv1_230050	protein of unknown function	7	5
METTOv1_230051	putative UBA/THIF-type NAD/FAD binding protein	7	6
METTOv1_240073	conserved protein of unknown function	7	3
METTOv1_260032	protein of unknown function	7	6
METTOv1_260033	conserved protein of unknown function	7	10
METTOv1_270021	hydrogenase expression/formation protein HypD	7	9
METTOv1_270029	hydrogenase expression/formation protein hupH	7	7
METTOv1_270037	uptake hydrogenase accessory protein HupV	7	4
METTOv1_320047	exported protein of unknown function	7	0
METTOv1_320072	fragment of putative transposase (part 2)	7	5
METTOv1_470036	conserved protein of unknown function	7	8
METTOv1_480008	protein of unknown function	7	7
METTOv1_500013	molybdate ABC transporter, ATPase subunit	7	7
METTOv1_500021	conserved protein of unknown function	7	11
METTOv1_540023	protein of unknown function	7	9
METTOv1_580004	putative RhiD protein	7	7
METTOv1_600016	putative Cytochrome B561	7	9
METTOv1_610020	exported protein of unknown function	7	5
METTOv1_650013	protein of unknown function DUF323	7	5
METTOv1_710021	conserved protein of unknown function; putative signal peptide	7	8
METTOv1_770003	putative Response regulator/sensor histidine kinase	7	6
METTOv1_800010	protein of unknown function	7	10
METTOv1_800011	protein of unknown function	7	21
METTOv1_890005	conserved exported protein of unknown function	7	10
METTOv1_900009	Deoxyribodipyrimidine photo-lyase	7	6
METTOv1_910009	exported protein of unknown function	7	4
METTOv1_920013	protein of unknown function	7	13
METTOv1_950005	fragment of Putative Transposase IS66 family (part 1)	7	7
METTOv1_950013	conserved protein of unknown function	7	6
METTOv1_1010006	protein of unknown function	7	19
METTOv1_1050007	protein of unknown function	7	3
METTOv1_1070001	protein of unknown function	7	10

METTOv1_1140009	protein of unknown function	7	7
METTOv1_1360001	peptide synthetase (fragment)	7	5
METTOv1_10218	fragment of Penicillin amidase (part 2)	6	11
METTOv1_20090	O-antigen polymerase	6	5
METTOv1_20174	protein of unknown function	6	16
METTOv1_30091	protein of unknown function	6	6
METTOv1_40114	protein of unknown function	6	3
METTOv1_50114	protein of unknown function	6	4
METTOv1_60050	conserved protein of unknown function	6	5
METTOv1_60083	Amylo-alpha-16-glucosidase	6	6
METTOv1_60085	Mannose-1-phosphate guanyltransferase (modular protein)	6	10
METTOv1_70015	protein of unknown function	6	8
METTOv1_80063	protein of unknown function DUF395 YeeE/YedE	6	7
METTOv1_90011	protein of unknown function	6	9
METTOv1_100013	Glycosyl transferase family 2	6	7
METTOv1_100015	protein of unknown function	6	2
METTOv1_100021	conserved protein of unknown function	6	6
METTOv1_100024	putative O-antigen polymerase	6	11
METTOv1_110031	conserved protein of unknown function	6	7
METTOv1_170017	protein of unknown function	6	7
METTOv1_170023	exported protein of unknown function	6	4
METTOv1_170030	conserved protein of unknown function	6	6
METTOv1_170031	protein of unknown function	6	0
METTOv1_170053	conserved protein of unknown function	6	5
METTOv1_170067	protein of unknown function	6	9
METTOv1_190019	2-oxoisovalerate dehydrogenase beta subunit	6	6
METTOv1_200039	Carbonic anhydrase	6	2
METTOv1_210028	putative outer membrane protein B; OmpB	6	10
METTOv1_230011	Integrase family protein	6	7
METTOv1_240059	Pyridoxal-dependent decarboxylase	6	7
METTOv1_240060	Regulatory protein ada	6	2
METTOv1_250017	Methyltransferase type 11	6	8
METTOv1_260014	conserved protein of unknown function	6	0
METTOv1_270034	Ni/Fe-hydrogenase, 1 b-type cytochrome subunit	6	2
METTOv1_270048	Membrane protein	6	4
METTOv1_280011	Pyrazinamidase/nicotinamidase, PncA protein	6	11
METTOv1_280015	fragment of Erythromycin esterase (part 2)	6	1
METTOv1_320011	protein of unknown function	6	0
METTOv1_320029	protein of unknown function	6	9
METTOv1_320033	conserved protein of unknown function	6	8
METTOv1_350047	two-component response transcriptional regulator (OmpR family)	6	8
METTOv1_400033	conserved membrane protein of unknown function	6	7
METTOv1_470007	transposase (fragment)	6	4
METTOv1_470009	protein of unknown function	6	5
METTOv1_480010	protein of unknown function	6	10
METTOv1_500022	transcriptional regulator, XRE family (fragment)	6	4
METTOv1_560003	Acyltransferase 3	6	7
METTOv1_660019	ABC-type nitrate/sulfonate/bicarbonate transport system ATPase component (fragment)	6	3
METTOv1_710020	conserved protein of unknown function; putative exported protein	6	4
METTOv1_710022	putative sulfur/thiosulfate oxidation protein (SoxB)	6	5

METTOv1_770001	protein of unknown function	6	30
METTOv1_850003	putative TonB-dependent siderophore receptor	6	4
METTOv1_850005	transport system permease protein	6	5
METTOv1_850007	putative TonB family protein	6	11
METTOv1_920005	Acetoacetyl-CoA reductase	6	7
METTOv1_950009	fragment of Putative Transposase IS66 family (part 2)	6	9
METTOv1_990002	Polyketide synthase	6	8
METTOv1_1250005	conserved protein of unknown function	6	0
METTOv1_1540001	protein of unknown function	6	7
METTOv1_1720002	putative conjugal transfer protein trbJ (fragment)	6	14
METTOv1_10013	putative pyoverdine chromophore synthetase	5	5
METTOv1_10111	N-acetylmuramic acid 6-phosphate etherase	5	3
METTOv1_10112	Glutamine--fructose-6-phosphate transaminase (Isomerizing)	5	7
METTOv1_10199	exported protein of unknown function	5	6
METTOv1_10221	protein of unknown function	5	5
METTOv1_20024	Phenylalanine-4-hydroxylase	5	5
METTOv1_20179	protein of unknown function	5	2
METTOv1_30018	CRISPR-associated protein, Cas2 family	5	13
METTOv1_30030	Pirin domain protein	5	8
METTOv1_40041	Transcriptional regulatory protein (fragment)	5	6
METTOv1_40074	protein of unknown function	5	4
METTOv1_40125	acetyl-CoA acetyltransferase with thiolase domain	5	6
METTOv1_60082	glycosyl transferase group 1	5	8
METTOv1_70011	conserved protein of unknown function	5	5
METTOv1_100022	protein of unknown function	5	9
METTOv1_100071	Putative sulfate transporter with a STAS domain (C-terminal)	5	5
METTOv1_110012	conserved exported protein of unknown function	5	5
METTOv1_120057	protein of unknown function	5	4
METTOv1_160038	Diaminobutyrate--2-oxoglutarate aminotransferase	5	5
METTOv1_170022	protein of unknown function	5	10
METTOv1_170043	GpW	5	0
METTOv1_170069	DNA polymerase III subunit beta	5	2
METTOv1_170070	Single-stranded DNA-binding protein	5	7
METTOv1_170071	ProP effector (fragment)	5	5
METTOv1_230022	protein of unknown function	5	8
METTOv1_230023	putative methylase/helicase	5	3
METTOv1_230030	Mercuric ion transport protein, MerT	5	7
METTOv1_230076	Methane monooxygenase component C	5	8
METTOv1_230079	conjugal transfer protein trbB	5	7
METTOv1_230080	putative conjugal transfer protein; TrbC	5	2
METTOv1_240058	putative colanic acid biosynthesis glycosyl transferase	5	5
METTOv1_260004	conjugal transfer protein	5	0
METTOv1_260027	conserved protein of unknown function	5	9
METTOv1_260030	conserved protein of unknown function	5	7
METTOv1_260031	putative methylase/helicase	5	5
METTOv1_270022	hydrogenase assembly chaperone hypC/hupF	5	6
METTOv1_270023	(NiFe) hydrogenase maturation protein HypF	5	5
METTOv1_270026	putative Hydrogenase expression/formation protein hupK	5	9
METTOv1_270035	Uptake hydrogenase large subunit precursor	5	11
METTOv1_270049	CBS domain-containing protein yhcV	5	1
METTOv1_280012	conserved protein of unknown function	5	3
METTOv1_290015	protein of unknown function	5	6
METTOv1_290017	conserved membrane protein of unknown function	5	4

METTOv1_290018	efflux transporter, RND family, MFP subunit	5	5
METTOv1_320031	putative head-tail adaptor protein	5	0
METTOv1_320032	conserved protein of unknown function	5	5
METTOv1_320034	conserved protein of unknown function	5	5
METTOv1_320035	protein of unknown function	5	4
METTOv1_320039	conserved protein of unknown function	5	9
METTOv1_320043	exported protein of unknown function	5	4
METTOv1_320067	protein of unknown function	5	5
METTOv1_320071	putative TonB-dependent siderophore receptor	5	4
METTOv1_330014	Putative branched-chain amino acid ABC transporter (permease protein)	5	7
METTOv1_470015	exported protein of unknown function	5	6
METTOv1_480021	protein of unknown function DUF323	5	6
METTOv1_490002	protein of unknown function	5	3
METTOv1_500016	putative TonB-dependent siderophore receptor	5	7
METTOv1_700020	UDP-hexose transferase	5	6
METTOv1_750002	protein of unknown function	5	4
METTOv1_780006	conserved membrane protein of unknown function	5	4
METTOv1_890008	protein of unknown function	5	6
METTOv1_940005	Penicillin acylase II	5	5
METTOv1_950014	putative transcriptional regulator, XRE family	5	0
METTOv1_1100002	protein of unknown function	5	5
METTOv1_1140012	protein of unknown function	5	3
METTOv1_1160001	Polyketide synthase (fragment)	5	4
METTOv1_10093	protein of unknown function	4	0
METTOv1_10113	N-acetylglucosamine-6-phosphate deacetylase	4	3
METTOv1_20017	Amine oxidase	4	5
METTOv1_20025	Tryptophanase	4	5
METTOv1_20162	protein of unknown function	4	4
METTOv1_30070	Phage major capsid protein, HK97 (modular protein)	4	4
METTOv1_40017	putative Glycine betaine/carnitine/choline ABC transporter (ATP-binding protein)	4	5
METTOv1_40112	conserved membrane protein of unknown function	4	3
METTOv1_40124	enoyl-CoA hydratase/isomerase	4	2
METTOv1_50078	GAF modulated sigma54 specific transcriptional regulator, Fis family	4	3
METTOv1_70012	protein of unknown function	4	4
METTOv1_70018	protein of unknown function	4	3
METTOv1_70019	protein of unknown function	4	4
METTOv1_70020	conserved protein of unknown function	4	5
METTOv1_100023	putative Bacterial sugar transferase family protein	4	1
METTOv1_100026	conserved protein of unknown function	4	4
METTOv1_100030	protein of unknown function	4	2
METTOv1_110016	Phage major capsid protein, HK97 family	4	2
METTOv1_120021	protein of unknown function	4	3
METTOv1_120052	conserved protein of unknown function	4	6
METTOv1_170025	putative Peptidoglycan-binding domain 1 protein	4	3
METTOv1_170041	Peptidase S49 (fragment)	4	4
METTOv1_170044	Putative phage terminase large subunit	4	5
METTOv1_170051	conserved protein of unknown function	4	3
METTOv1_190020	exported protein of unknown function	4	10
METTOv1_190022	Radical SAM domain protein (fragment)	4	4

METTOv1_220022	putative AlgJ protein, required for O-acetylation of alginate in Pseudomonas aeruginosa	4	6
METTOv1_230029	protein of unknown function	4	7
METTOv1_230066	putative conjugal transfer protein precursor	4	0
METTOv1_230083	putative conjugal transfer protein trbJ	4	4
METTOv1_250046	alginate O-acetyltransferase	4	3
METTOv1_260003	conjugal transfer protein trbE	4	3
METTOv1_260009	conserved protein of unknown function	4	3
METTOv1_260010	Putative soluble lytic murein transglycosylase precursor	4	5
METTOv1_260011	conserved protein of unknown function	4	4
METTOv1_270027	hydrogenase expression/formation protein hupJ	4	1
METTOv1_270042	exported protein of unknown function	4	8
METTOv1_280016	Cold-shock DNA-binding domain protein	4	5
METTOv1_290020	acriflavin resistance protein	4	4
METTOv1_320006	protein of unknown function	4	1
METTOv1_320010	protein of unknown function	4	4
METTOv1_320017	putative methyltransferase	4	4
METTOv1_320018	protein of unknown function	4	4
METTOv1_320025	Phage major capsid protein	4	4
METTOv1_320036	conserved protein of unknown function	4	10
METTOv1_320037	protein of unknown function	4	5
METTOv1_330013	urea ABC transporter, ATP-binding protein UrtD	4	5
METTOv1_360008	conserved protein of unknown function	4	3
METTOv1_360009	conserved protein of unknown function	4	3
METTOv1_360012	conserved protein of unknown function	4	4
METTOv1_480024	putative Asparagine synthase	4	3
METTOv1_500011	Molybdenum ABC transporter, periplasmic molybdate-binding protein	4	5
METTOv1_660005	RND efflux system, outer membrane lipoprotein, NodT family (fragment)	4	3
METTOv1_680010	Sensor protein	4	6
METTOv1_770015	protein of unknown function	4	7
METTOv1_800001	conjugal transfer protein; TrbL (modular protein)	4	5
METTOv1_850002	protein of unknown function	4	5
METTOv1_910011	conserved protein of unknown function	4	6
METTOv1_1040009	protein of unknown function	4	0
METTOv1_1100003	conserved protein of unknown function	4	0
METTOv1_1140011	protein of unknown function	4	6
METTOv1_1720001	conserved protein of unknown function; putative trbK parecursor	4	4
METTOv1_10104	conserved exported protein of unknown function	3	3
METTOv1_10149	Polysaccharide export protein (modular protein)	3	5
METTOv1_20021	LmbE family protein	3	4
METTOv1_20022	protein of unknown function	3	1
METTOv1_20023	protein of unknown function	3	3
METTOv1_20172	HNH endonuclease (modular protein)	3	4
METTOv1_20173	protein of unknown function	3	5
METTOv1_30089	protein of unknown function	3	3
METTOv1_30146	protein of unknown function	3	6
METTOv1_30148	protein of unknown function	3	4
METTOv1_40113	conserved membrane protein of unknown function	3	5
METTOv1_40118	bifunctional proline dehydrogenase/pyrroline-5-carboxylate dehydrogenase	3	4

METTOv1_40120	Propionyl-CoA carboxylase	3	4
METTOv1_60062	O-antigen export system permease protein	3	2
METTOv1_60063	putative GDP-mannose-dependent alpha-(1-6)-phosphatidylinositol monomannoside mannosyltransferase	3	1
METTOv1_70021	protein of unknown function	3	1
METTOv1_90007	Putative ABC transporter (permease protein)	3	3
METTOv1_100012	protein of unknown function	3	4
METTOv1_100016	protein of unknown function	3	0
METTOv1_100027	protein of unknown function	3	3
METTOv1_100028	Capsular exopolysaccharide family	3	3
METTOv1_100041	transcriptional regulator, MarR family	3	3
METTOv1_110018	putative Phage head-tail adaptor	3	2
METTOv1_110025	conserved protein of unknown function	3	2
METTOv1_120028	protein of unknown function	3	8
METTOv1_120051	AcrB/AcrD/AcrF family protein	3	4
METTOv1_170024	membrane protein of unknown function	3	6
METTOv1_170032	conserved protein of unknown function	3	2
METTOv1_170042	Phage portal protein, lambda family	3	2
METTOv1_170055	putative DNA primase	3	2
METTOv1_170056	protein of unknown function	3	6
METTOv1_230060	conserved protein of unknown function	3	2
METTOv1_230063	putative plasmid partition protein	3	3
METTOv1_240077	conjugal transfer protein; TrbL (modular protein)	3	3
METTOv1_260006	conjugal transfer protein trbB	3	1
METTOv1_260013	conserved protein of unknown function	3	0
METTOv1_260015	putative plasmid partition protein	3	3
METTOv1_260018	conserved protein of unknown function	3	0
METTOv1_270040	NADH dehydrogenase I, D subunit	3	3
METTOv1_270041	protein of unknown function DUF1328	3	0
METTOv1_270043	protein of unknown function	3	6
METTOv1_270044	ribonuclease BN	3	4
METTOv1_270050	protein of unknown function	3	0
METTOv1_280013	Nicotinate phosphoribosyltransferase pncB2	3	3
METTOv1_280014	Ribose-phosphate pyrophosphokinase (modular protein)	3	1
METTOv1_290009	Putative Arylsulfatase	3	2
METTOv1_290023	putative cytochrome c region protein	3	3
METTOv1_290027	protein of unknown function	3	4
METTOv1_320021	protein of unknown function	3	3
METTOv1_320022	Phage terminase-like protein large subunit	3	2
METTOv1_320038	conserved protein of unknown function	3	3
METTOv1_320042	protein of unknown function	3	2
METTOv1_320044	conserved protein of unknown function	3	3
METTOv1_360011	protein of unknown function	3	4
METTOv1_360050	conserved protein of unknown function	3	2
METTOv1_410014	conserved exported protein of unknown function	3	0
METTOv1_480026	conserved protein of unknown function	3	6
METTOv1_660003	acriflavin resistance protein	3	3
METTOv1_710023	putative sulfur oxidation protein (soxZ)	3	2
METTOv1_750007	putative type I polyketide synthase component	3	2
METTOv1_750010	Catalytic domain of component of various dehydrogenase complexes	3	1
METTOv1_750014	Polyketide synthase (fragment)	3	2
METTOv1_850006	ABC transporter related	3	5

METTOv1_850011	membrane protein of unknown function	3	6
METTOv1_890007	protein of unknown function	3	7
METTOv1_940002	putative transcriptional regulator (fragment)	3	3
METTOv1_940004	putative ABC transporter ATP-binding protein yojI	3	3
METTOv1_950007	Putative transposase; putative IS66 family element, orf2	3	2
METTOv1_950015	conserved membrane protein of unknown function	3	1
METTOv1_1250007	protein of unknown function	3	4
METTOv1_10109	putative membrane protein	2	1
METTOv1_20018	conserved protein of unknown function	2	5
METTOv1_20144	protein of unknown function	2	0
METTOv1_20163	protein of unknown function	2	3
METTOv1_20164	protein of unknown function	2	2
METTOv1_30035	Cytochrome O ubiquinol oxidase protein subunit IV (modular protein)	2	6
METTOv1_30037	cytochrome o ubiquinol oxidase, subunit I	2	2
METTOv1_30071	Phage portal protein, HK97 family	2	3
METTOv1_30147	putative Single-strand binding protein/Primosomal replication protein n	2	2
METTOv1_60051	Glycosyltransferase	2	4
METTOv1_60057	putative Glycosyl transferase group 1	2	1
METTOv1_60059	protein of unknown function	2	0
METTOv1_60060	Polysaccharide ABC transporter, ATPase component	2	2
METTOv1_60064	Asparagine synthase (Glutamine-hydrolyzing)	2	2
METTOv1_90008	ABC transporter substrate-binding protein	2	3
METTOv1_100011	Glycosyl transferase group 1	2	2
METTOv1_100029	exported protein of unknown function	2	1
METTOv1_110013	conserved protein of unknown function	2	3
METTOv1_110017	conserved protein of unknown function	2	1
METTOv1_110021	Gene transfer agent	2	4
METTOv1_110022	conserved protein of unknown function	2	4
METTOv1_110023	conserved protein of unknown function	2	2
METTOv1_110024	conserved protein of unknown function	2	0
METTOv1_110027	conserved exported protein of unknown function	2	3
METTOv1_110028	conserved protein of unknown function	2	2
METTOv1_110029	protein of unknown function	2	2
METTOv1_110030	Endolysin	2	0
METTOv1_170037	protein of unknown function	2	2
METTOv1_170073	protein of unknown function	2	10
METTOv1_190023	Glutamate dehydrogenase	2	2
METTOv1_230081	conjugal transfer protein	2	0
METTOv1_230082	conjugal transfer protein trbE	2	3
METTOv1_230084	protein of unknown function	2	0
METTOv1_240076	conjugal transfer protein TrbF	2	4
METTOv1_260007	conserved protein of unknown function	2	0
METTOv1_260008	Putative Conjugal transfer protein traG	2	3
METTOv1_270047	fragment of ATP-dependent DNA ligase (part 2)	2	1
METTOv1_290011	protein of unknown function	2	4
METTOv1_290019	acriflavin resistance protein	2	3
METTOv1_290025	fragment of ABC-type nitrate/sulfonate/bicarbonate transport systems periplasmic components-like protein (part 2)	2	3
METTOv1_290026	fragment of ABC-type nitrate/sulfonate/bicarbonate transport systems periplasmic components-like protein (part 1)	2	3

METTOv1_290028	binding-protein-dependent transport systems inner membrane component	2	1
METTOv1_300024	protein of unknown function	2	0
METTOv1_320005	protein of unknown function	2	2
METTOv1_320008	exported protein of unknown function	2	6
METTOv1_320009	conserved protein of unknown function	2	7
METTOv1_320012	protein of unknown function	2	0
METTOv1_320013	conserved protein of unknown function	2	2
METTOv1_320014	protein of unknown function	2	1
METTOv1_320015	protein of unknown function	2	0
METTOv1_320023	Phage portal protein, HK97 family	2	1
METTOv1_320041	conserved protein of unknown function	2	3
METTOv1_320046	Lysozyme (modular protein)	2	2
METTOv1_320073	fragment of putative transposase (part 1)	2	3
METTOv1_360013	conserved protein of unknown function	2	2
METTOv1_470010	Bacteriophage tail assembly protein (fragment)	2	3
METTOv1_470040	Putative transposase; putative IS66 family element, orf2	2	0
METTOv1_480025	protein of unknown function	2	5
METTOv1_490001	transposase (fragment)	2	3
METTOv1_500012	Molybdenum ABC transporter (permease protein)	2	4
METTOv1_500017	Nitrogenase	2	2
METTOv1_710024	Putative sulfur oxidation protein (soxY)	2	2
METTOv1_750008	putative FkbH like protein	2	1
METTOv1_750009	putative Acyl carrier protein ACP	2	6
METTOv1_850004	Nitrogenase	2	2
METTOv1_900006	exported protein of unknown function	2	3
METTOv1_910002	conserved protein of unknown function	2	2
METTOv1_940003	protein of unknown function	2	4
METTOv1_990001	protein of unknown function	2	2
METTOv1_1000001	conserved protein of unknown function	2	2
METTOv1_1140010	protein of unknown function	2	1
METTOv1_10094	protein of unknown function	1	0
METTOv1_10108	membrane protein of unknown function	1	3
METTOv1_10114	putative Beta-hexosaminidase	1	2
METTOv1_10151	protein of unknown function	1	2
METTOv1_30074	protein of unknown function	1	19
METTOv1_40111	Quinol oxidase subunit II	1	4
METTOv1_60052	putative Glycosyl transferase, group 1 family protein	1	1
METTOv1_60054	Glycosyl transferase, group 1	1	1
METTOv1_60055	conserved protein of unknown function	1	0
METTOv1_60056	conserved protein of unknown function	1	1
METTOv1_60058	putative Glycosyl transferase group 1	1	1
METTOv1_60061	protein of unknown function	1	0
METTOv1_60065	protein of unknown function	1	1
METTOv1_110014	conserved protein of unknown function	1	0
METTOv1_110019	conserved protein of unknown function	1	3
METTOv1_110020	conserved protein of unknown function	1	6
METTOv1_110026	putative phage cell wall peptidase, NlpC/P60	1	3
METTOv1_170034	Baseplate assembly protein J	1	0
METTOv1_170036	protein of unknown function	1	4
METTOv1_170066	conserved protein of unknown function	1	3
METTOv1_230061	conserved protein of unknown function	1	0
METTOv1_230064	conserved protein of unknown function	1	3

METTOv1_230065	conserved protein of unknown function	1	1
METTOv1_230067	conserved protein of unknown function	1	2
METTOv1_230068	Putative soluble lytic murein transglycosylase precursor	1	1
METTOv1_260002	putative conjugal transfer protein trbJ	1	2
METTOv1_270045	putative DNA repair protein ACP_3505	1	2
METTOv1_290008	conserved protein of unknown function; Tetratricopeptide repeat	1	2
METTOv1_290014	Acylphosphatase	1	1
METTOv1_320007	conserved protein of unknown function	1	7
METTOv1_320016	protein of unknown function	1	0
METTOv1_320024	ATP-dependent Clp protease proteolytic subunit	1	2
METTOv1_320028	protein of unknown function	1	0
METTOv1_320040	NLP/P60 protein	1	2
METTOv1_320045	protein of unknown function	1	2
METTOv1_320070	conserved protein of unknown function; putative signal peptide; putative DJ-1/PfpI family protein	1	2
METTOv1_360010	conserved protein of unknown function	1	3
METTOv1_660004	efflux transporter, RND family, MFP subunit	1	1
METTOv1_1250004	protein of unknown function	1	2
METTOv1_1250006	transposase (fragment)	1	1
METTOv1_tRNA1	His tRNA	0	0
METTOv1_tRNA2	Phe tRNA	0	0
METTOv1_tRNA48	Glu tRNA	0	0
METTOv1_tRNA3	Ala tRNA	0	0
METTOv1_tRNA4	Val tRNA	0	0
METTOv1_30090	protein of unknown function	0	0
METTOv1_tRNA5	Lys tRNA	0	0
METTOv1_tRNA6	Lys tRNA	0	0
METTOv1_tRNA47	Ser tRNA	0	0
METTOv1_tRNA7	Leu tRNA	0	0
METTOv1_tRNA46	Thr tRNA	0	0
METTOv1_tRNA8	Arg tRNA	0	0
METTOv1_tRNA45	Pro tRNA	0	0
METTOv1_tRNA9	Arg tRNA	0	0
METTOv1_tRNA44	Ser tRNA	0	0
METTOv1_110015	protein of unknown function	0	0
METTOv1_tRNA43	Leu tRNA	0	0
METTOv1_tRNA42	Gly tRNA	0	0
METTOv1_tRNA41	Trp tRNA	0	0
METTOv1_170033	protein of unknown function	0	0
METTOv1_170035	GPW/gp25 (fragment)	0	0
METTOv1_tRNA40	Met tRNA	0	0
METTOv1_tRNA10	Glu tRNA	0	0
METTOv1_tRNA11	Leu tRNA	0	0
METTOv1_tRNA39	Pro tRNA	0	0
METTOv1_tRNA12	Val tRNA	0	0
METTOv1_tRNA38	Gly tRNA	0	0
METTOv1_tRNA37	Val tRNA	0	0
METTOv1_tRNA13	Thr tRNA	0	0
METTOv1_tRNA36	Gln tRNA	0	0
METTOv1_260017	conserved protein of unknown function	0	1
METTOv1_tRNA35	Pseudo tRNA	0	0
METTOv1_tRNA34	Arg tRNA	0	0

METTOv1_270046	fragment of ATP-dependent DNA ligase (part 1)	0	1
METTOv1_320001	protein of unknown function	0	4
METTOv1_tRNA14	Gly tRNA	0	0
METTOv1_tRNA33	Cys tRNA	0	0
METTOv1_tRNA15	Ser tRNA	0	0
METTOv1_tRNA16	Asn tRNA	0	0
METTOv1_tRNA32	Gly tRNA	0	0
METTOv1_tRNA31	Tyr tRNA	0	0
METTOv1_tRNA30	Thr tRNA	0	0
METTOv1_tRNA29	Asp tRNA	0	0
METTOv1_tRNA17	Arg tRNA	0	0
METTOv1_tRNA18	Gln tRNA	0	0
METTOv1_tRNA19	Leu tRNA	0	0
METTOv1_tRNA20	Met tRNA	0	0
METTOv1_tRNA28	Ser tRNA	0	0
METTOv1_tRNA21	Gly tRNA	0	0
METTOv1_tRNA27	Leu tRNA	0	0
METTOv1_tRNA26	Ala tRNA	0	0
METTOv1_tRNA22	Pro tRNA	0	0
METTOv1_tRNA23	Arg tRNA	0	0
METTOv1_16s_rRNA_	ribosomal RNA 16s_rRNA	0	0
METTOv1_tRNA24	Ile tRNA	0	0
METTOv1_tRNA25	Ala tRNA	0	0
METTOv1_23s_rRNA_	ribosomal RNA 23s_rRNA	0	0
METTOv1_5s_rRNA_1	ribosomal RNA 5s_rRNA	0	0

Table S3. Summary of putative transcription site mapping. [Note that the published sequences need references added.]

Strain	Gene	Putative TS*	-35	-10
M. trichosporium OB3b	*pmoC*	-273	**TTG**T**CA**	AATGGTT G
	pmoC	-324	**TTG**TGC	TCCTACC CTA
	mxaF	-156	CA**GACA**	**TATA**TG
	fae1	-214	**TT**CGAT	**TATA**AT
	pqqA	-90	**TTG**CAC	GC**TAAT**
Methylocystis sp. M[1]	*pmoC*		**TTG**T**CA**	GA**AT**GG**T** TG
M.extorquens AM1[2]	*mxaF*		AA**GACA**	**TA**G**A**A**A**
	mxaW		**TTG**GCA	ACCCAT
E. coli [3]	σ70		**TTGACA**	**TATAAT**
	σ32		TCTCNCCCTTGAA	
	σ54		CTGGNA	TTGCA
	σ28		CTAAA	CCGATAT

1 Holmes *et al.*, 1995 (see Ref)
2. Zhang, M., and Lidstrom, M.E. (2003) Microbiol 149:1033-40.
3. Harley, C.B., and Reynolds, R.P. 1987, Nucleic Acids Res. 11:2343-61

Table S4. Summary of RNA-seq (Illumina) reads

	Reads Mapped		% of Reads Mapped	
	Replicate 1	Replicate 2	Replicate 1	Replicate 2
Coding sequences	$3.652*10^6$	$1.546*10^6$	13.3	5.44
Noncoding sequences	$2.935*10^5$	$1.396.*10^5$	1.07	0.49
rRNA	$2.359*10^7$	$2.671*10^7$	85.6	94.0
tRNA	$7.937*10^3$	$4.095*10^3$	0.029	0.0014

Table S5. Genes removed from reference scaffold before alignment

Locus tag	Comment
METTOv1_1600001	chimeric sequence/ METTOv1_870001 and METTOv1_870003
METTOv1_820016	protein of unknown function
METTOv1_150030	protein of unknown function
METTOv1_300034	protein of unknown function/gltD operon
METTOv1_1650001	chimeric sequence/part of METATv1_30343 and METATv1_30340
METTOv1_140042	chimeric protein/part of METTOv1_140043
METTOv1_1700001	chimeric sequence/METTOv1_870001 and METTOv1_870003
METTOv1_10052	chimeric protein/part of METTOv1_10051
METTOv1_200054	chimeric protein/part of METTOv1_200053
METTOv1_600024	exported protein of unknown function
METTOv1_370034	protein of unknown function
METTOv1_200053	protein of unknown function
METTOv1_720016	protein of unknown function
METTOv1_450003	protein of unknown function
METTOv1_590015	protein of unknown function
METTOv1_360021	protein of unknown function
METTOv1_800023	protein of unknown function//part of transposase IS3/IS911
METTOv1_760015	protein of unknown function
METTOv1_270007	protein of unknown function
METTOv1_210063	protein of unknown function
METTOv1_10194	protein of unknown function
METTOv1_200055	protein of unknown function
METTOv1_450005	protein of unknown function
METTOv1_1280001	protein of unknown function
METTOv1_1050002	protein of unknown function
METTOv1_160016	protein of unknown function
METTOv1_200057	protein of unknown function
METTOv1_260041	protein of unknown function

METTOv1_600022	exported protein of unknown function
METTOv1_870007	protein of unknown function
METTOv1_260040	protein of unknown function
METTOv1_90064	protein of unknown function
METTOv1_30137	exported protein of unknown function
METTOv1_590023	protein of unknown function
METTOv1_250030	protein of unknown function
METTOv1_360046	protein of unknown function
METTOv1_830011	protein of unknown function
